# Whole-Genome Analyses of Korean Native and Holstein Cattle Breeds by Massively Parallel Sequencing

**DOI:** 10.1371/journal.pone.0101127

**Published:** 2014-07-03

**Authors:** Jung-Woo Choi, Xiaoping Liao, Paul Stothard, Won-Hyong Chung, Heoyn-Jeong Jeon, Stephen P. Miller, So-Young Choi, Jeong-Koo Lee, Bokyoung Yang, Kyung-Tai Lee, Kwang-Jin Han, Hyeong-Cheol Kim, Dongkee Jeong, Jae-Don Oh, Namshin Kim, Tae-Hun Kim, Hak-Kyo Lee, Sung-Jin Lee

**Affiliations:** 1 Centre for Genetic Improvement of Livestock, Animal & Poultry Science, University of Guelph, Guelph, Ontario, Canada; 2 Department of Agricultural, Food and Nutritional Science, University of Alberta, Edmonton, Canada; 3 Korean Bioinformation Center, Korea Research Institute of Bioscience and Biotechnology, Daejeon, Republic of Korea; 4 Division of Animal Genomics and Bioinformatics, National Institute of Animal Science, Rural Development Administration, Suwon, Republic of Korea; 5 College of Animal Life Sciences, Kangwon National University, Chuncheon, Republic of Korea; 6 Theragen BiO Institute, TheragenEtex, Suwon, Republic of Korea; 7 Dairy Cattle Improvement Center, National Agricultural Cooperative Federation, Goyang-Si, Republic of Korea; 8 Hanwoo Experiment Station, National Institute of Animal Science, Rural Development Administration, Gangwon-do, Republic of Korea; 9 Department of Biotechnology, Jeju National University, Jeju, Republic of Korea; 10 Department of Biotechnology, Hankyong National University, Anseong, Republic of Korea; Wageningen UR Livestock Research, Netherlands

## Abstract

A main goal of cattle genomics is to identify DNA differences that account for variations in economically important traits. In this study, we performed whole-genome analyses of three important cattle breeds in Korea—Hanwoo, Jeju Heugu, and Korean Holstein—using the Illumina HiSeq 2000 sequencing platform. We achieved 25.5-, 29.6-, and 29.5-fold coverage of the Hanwoo, Jeju Heugu, and Korean Holstein genomes, respectively, and identified a total of 10.4 million single nucleotide polymorphisms (SNPs), of which 54.12% were found to be novel. We also detected 1,063,267 insertions–deletions (InDels) across the genomes (78.92% novel). Annotations of the datasets identified a total of 31,503 nonsynonymous SNPs and 859 frameshift InDels that could affect phenotypic variations in traits of interest. Furthermore, genome-wide copy number variation regions (CNVRs) were detected by comparing the Hanwoo, Jeju Heugu, and previously published Chikso genomes against that of Korean Holstein. A total of 992, 284, and 1881 CNVRs, respectively, were detected throughout the genome. Moreover, 53, 65, 45, and 82 putative regions of homozygosity (ROH) were identified in Hanwoo, Jeju Heugu, Chikso, and Korean Holstein respectively. The results of this study provide a valuable foundation for further investigations to dissect the molecular mechanisms underlying variation in economically important traits in cattle and to develop genetic markers for use in cattle breeding.

## Introduction

**Figure 1:**
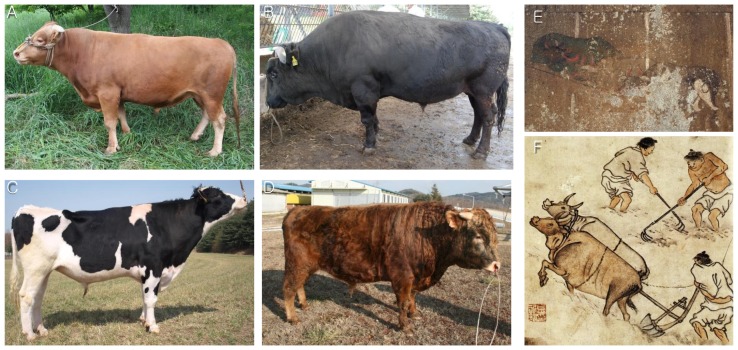
Korean cattle breeds used in this study. (A) Hanwoo. (B) Jeju Heugu. (C) Korean Holstein. (D) Chikso. (E) Mural painting in the Anak tomb no. 3 (A.D. 357) in the Goguryeo age: a stable illustrating three cattle in black, yellow, and brindle colors (courtesy of Dr. Ho-Tae Jeon). F: Eighteenth century painting by Hong-Do Kim (1745–1806), Joseon Dynasty, depicting farmers ploughing a rice field using Korean native cattle (at the National Museum of Korea).

Native cattle have been raised across the Korea peninsula since 2000 B.C [1]. There are currently four Korean native cattle breeds registered with the Food and Agricultural Organization: Hanwoo (Korean brown cattle), Jeju Heugu (Jeju black cattle), Chikso (Korean brindle cattle), and Heugu (Korean black cattle) [2]. Each breed has its own characteristics, particularly in hair color (Fig. 1A–D) [3], [4], and historical records indicate that they were mainly used as pack and draft animals (Fig. 1E–F). Since the 1960s, Korean native cattle have been mainly used for beef because of increasing meat consumption coupled with the growth of the Korean economy in the recent decades.

A selective breeding program for Hanwoo was initiated in 1979, and it has contributed to significant increases in economically important traits, such as carcass yield and marbling scores [1], [5]. Unlike Hanwoo, the other three native cattle breeds are threatened with extinction because of policies to unify cattle coat colors at the beginning of the 20^th^ century. However, those cattle breeds are currently being revaluated to conserve local genetic resources and to pioneer new niche markets to meet demands for safe meats from Korean native breeds in Korea. Holstein has been the most widely used dairy breed in Korea since its introduction there in 1885. Since the initiation of the official dairy herd improvement program in 1979, Holsteins have been intensively selected for Korean environments. As a result, milk traits have dramatically improved in the past 30 years; for example, yields of 4,681 kg of milk per cow per lactation can be achieved [6].

Thanks to the international bovine genome sequencing and HapMap projects, substantial numbers of single nucleotide polymorphisms (SNPs) are known throughout the cattle genome [7], [8]. They have contributed to the development of SNP marker panels, which are widely used to detect signatures of selection and for genome-wide association studies in cattle [9]–[15]. Recent advances in massively parallel sequencing technologies (a.k.a. next generation sequencing, NGS) have further catalogued large amounts of genetic variation. Several recent studies successfully applied NGS technologies in cattle while showing that many SNPs and insertions–deletions (InDels) remain to be detected, especially in diverse cattle breeds or multiple individuals [16]–[20]. In addition to SNPs, copy number variation (CNV) has received much interest as another genetic parameter that could account for trait variation in cattle. Genome-wide CNVs in cattle were initially accessed via SNP genotyping platforms [21]–[24]. However, the size of each putative CNV tended to be overestimated because of its low density in the SNP array. More recently, NGS has been successfully applied in cattle as an approach to detect CNVs with improved resolution [17], [25], [26].

Despite these recent achievements, there is a drastic shortage of whole-genome sequencing (WGS) investigations for Asian cattle breeds and of their comparisons with European-origin cattle breeds that are widely used for beef or dairy [18], [26]. Among the few examples, Kuchinoshima-Ushi and Chikso were recently resequenced using the Illumina Genome Analyzer II and HiSeq 2000 sequencers, revealing approximately 6.30 and 5.97 million SNPs, respectively. As with previous reports on European *Bos taurus* cattle, these studies clearly demonstrated that there remain substantial numbers of SNPs to be discovered (87% and 45% novel SNPs for Kuchinoshima-Ushi and Chikso, respectively). In this article, we present WGS analyses of three *Bos taurus* cattle breeds, two Korean native breeds (Hanwoo and Jeju Heugu) and a Korean Holstein, using the Illumina HiSeq 2000 sequencing platform. In addition, a recently sequenced Chikso genome was included for comparison [20]. The representative animals for each breed in this study were influential sires to be used for artificial insemination to enhance the genetic potential of economically important traits in their populations.

## Materials and Methods

### Ethics statement

For sampling cattle breeds in this study, the study protocol and standard operating procedures were reviewed and approved by the National Institute of Animal Science's Institutional Animal Care and Use Committee.

### Sampling and DNA extraction

We sequenced three *Bos taurus* bull genomes from two Korean native cattle breeds, Hanwoo and Jeju Heugu, and a Korean Holstein as a representative dairy breed. These bulls were accessed at the Hanwoo Experiment Station, National Institute of Animal Science, Rural Development Administration, Pyongchang; the Jeju Provincial Livestock Research Institute, Jeju; and the Dairy Cattle Improvement Center, National Agricultural Cooperative Federation, Goyang respectively. Each bull was an influential sire for artificial insemination, with extensive phenotypic records and proper pedigree information. In particular, the Korean Holstein (named Eugene, code 208HO10170) had been ranked in the first percentile of elite sires in the international bull evaluation service database. Genomic DNA from each animal was isolated from the EDTA-blood, using the PAXgene Blood DNA Kit (PreAnalytiX GmbH, Hombrechtikon, Switzerland). The quality and quantity of the DNA were evaluated by the Qubit fluorometer (Invitrogen, Carlsbad, CA, USA) and Infinite F200 microplate reader (TECAN), according to the manufacturer's instruction. The status of the DNA was visually checked by 0.8% agarose gel electrophoresis.

### Library preparation for massively parallel sequencing

Purified genomic DNA was randomly sheared to yield DNA fragments of 400∼500 bp in size, and the average fragment size was determined using an Agilent Bioanalyzer 2100 (Agilent Technologies, Palo Alto, CA, USA). The fragments were ligated with index adapters using the Illumina TrueSeq End Repair-Kit and the AMPure XP Beads purification kit (Beckman Coulter Genomics, Danvers, MA, USA). After size selection of the ligation products using a 2% agarose gel, successfully ligated fragments were enriched via PCR with adapter-specific primer sets. The DNA was further isolated using AMPure XP Beads, and the resulting libraries were assessed on a 2100 Bioanalyzer (Agilent Technologies, Santa Clara, CA) and then sequenced by 100 bp paired-end sequencing using the Illumina HiSeq 2000 platform. Further image analysis and base calling were performed with the Illumina pipeline using default settings. Additionally, previously published data for Chikso was included for comparison with the three newly sequenced genomes obtained in this study. The genomic data of Chikso was generated using the same library construction and sequencing procedures as used in the current study [20].

### Mapping short reads, identification of SNPs and InDels, and their annotation

For each sample, sequence reads were removed if they failed the Illumina chastity filter or if the average Phred quality score was less than 20. Next, reads were trimmed to 90 bp to omit the error-prone ends. The remaining reads were mapped against the bovine genome assembly UMD 3.1 [27] including unassembled contigs using BWA ver. 0.5.9 [28]. BWA option ‘−q 20’ was applied to enable trimming of low-quality bases at the 3′-end. After mapping, local realignment was performed using GATK ver. 2.4 [29] and then duplicates were marked using Picard ver. 1.54 (http://picard.sourceforge.net). Variants were called using Samtools-0.1.18 mpileup [30]. All SNPs and InDels were identified as differences from the reference sequence. The resulting variant lists were filtered by removing the following: (1) SNPs and InDels with overall quality less than 20; (2) variants with very low (less than 10) or very high (more than the mean read depth plus three times the standard deviation) read depths; (3) variants with less than one forward or reverse alternative allele read; (4) variants within 5 bp of each other; (5) SNPs within 5 bp of an InDel; and (6) InDels within 10 bp of each other. After variant calling, functional annotation was performed using NGS-SNP [31]. The source databases for annotation included Ensembl release 68, Entrez Gene, NCBI and UniProt [32]–[34].

### Detecting Copy Number Variation Regions

Putative CNV regions (CNVRs) were detected for all 29 bovine autosomes and the X chromosome using the CNV-seq application, which compares two sets of mapped reads and reports genomic regions with significantly different read depths [35]. Three comparisons were made: Hanwoo versus Korean Holstein (HANvsHOL), Jeju Heugu versus Korean Holstein (JJHvsHOL), and Chikso versus Korean Holstein (CHSvsHOL). All mapped reads were converted to “best-hit” format files to be used as input files. Subsequently, a customized CNV-seq.pl script was run using the best-hit files and strict customized threshold values (*P* = 0.001 and log_2_ threshold = 0.7) with a window size of 5 to generate a list of CNVs. Additionally, we used a minimum-window-required setting of 10 to specify a CNVR by ten consecutive sliding windows showing a significant read depth difference.

### Detecting Regions of Homozygosity

A previously described method was followed to identify regions of homozygosity throughout all 29 bovine autosomes [19]. Each chromosome was divided into non-overlapping 400-kb bins and the ratio of homozygous SNPs per bin was calculated using genotype data derived from whole-genome resequencing in this study. A 0.95 ratio was imposed to determine the bins with high degree of homozygosity. Consecutive bins with high degrees of homozygosity were merged afterwards.

### Annotation of CNVRs and Gene Ontology analysis

NGS-SNP was used to assess the gene content of each CNVR by comparing its coordinates to the positions of genes in the Ensembl database (release 68) [32]. The Ensembl protein ID associated with each gene overlapping each variant was obtained using BioMart [36], and the set of protein IDs were analyzed in agriGO server to perform Gene Ontology (GO) analysis using the bovine genome locus background [37]. The singular enrichment analysis tool in agriGO was applied to identify enriched GO terms among the set of Ensembl protein IDs. The significance of term enrichments was assessed by Fisher's exact test [37], and the default multiple comparison correction (Benjamini–Yekutieli method) was applied.

### Validation of SNPs using BovineSNP50 array

Each sequenced animal was genotyped using the Illumina BovineSNP50 v2 BeadChip array (54,609 SNP probes), which includes markers spanning the entire reference sequence (UMD 3.1). Approximately 1% of the panel SNPs were excluded from further concordance analysis because their genomic coordinates could not be determined or because their alleles were incompatible with those detected by WGS. We calculated the concordance rate based on SNPs that were successfully genotyped by the chip array and that were not homozygous for the reference allele. Subsequently, we evaluated the genotype concordance by two measures: genotype concordance at variant sites, and non-reference sensitivity. Genotype concordance at variant sites is calculated by dividing the number of concordant non-reference genotypes by the total number of non-reference chip array genotypes. Non-reference sensitivity refers to the rate at which non-reference sites (heterozygous or homozygous non-reference) detected by the genotyping panel are recovered in the WGS-derived data and is calculated by dividing the number of non-reference genotypes identified by the chip array and classified as non-reference using WGS by the number of non-reference genotypes identified by the chip.

### Data access

All SNPs and InDels detected in this study have been submitted to dbSNP under the handle “AGL_CJW’ with the accession number: from 99630286 to 1026504451. The complete lists of CNVRs detected are provided in Tables S1–6.

## Results and Discussion

### Massively parallel sequencing and mapping

Genomic DNA from Hanwoo (HAN), Jeju Heugu (JJH), and Korean Holstein (HOL) were sequenced at a high depth of coverage using the Illumina HiSeq 2000 sequencing platform using 100-bp paired-end libraries. To the best of our knowledge, this study provides the first whole-genome sequence of Jeju Heugu. We applied rigorous custom filtering to the resulting sequence reads to detect high-confidence variants; including removing error prone regions at read ends and redundancy occurring in the library preparation step (see Materials and Methods for details). For HAN, JJH, and HOL, a total of 862,545,570, 1,017,728,088, and 992,361,054 initial reads were obtained, of which 98.66%, 98.48%, and 98.50%, respectively, were successfully mapped to the bovine reference assembly UMD 3.1. On average, 98.8%, 98.9%, and 98.9% of the reference genomes were covered by at least one read, and the coverages were 25.5-, 29.6-, and 29.5-fold, respectively (Table 1). Compared with previous sequencing studies in cattle [16]–[18], the depth of coverage is fairly high and more than sufficient to detect high-confidence variants, a conclusion further supported by the genotype concordance rates using the BovineSNP50 BeadChip array (see Section 3.2). The overall coverage differences among the three breeds are evenly distributed across the genome, with no distinct coverage bias on any particular chromosome (Fig. S1).

**Table 1:** Summary of sequencing results for Hanwoo, Jeju Heugu, and Korean Holstein cattle breeds.

Sample Name	Reads	Mapped Reads	Properly Paired	Fold_C^a^	Assembly_C^b^	Seq_C^c^.	Dup_R
Hanwoo	862,545,570	851,024,569 (98.66%)	825,218,262 (95.67%)	25.52	98.83%	25.82	0.108
Jeju Heugu	1,017,728,088	1,002,248,995 (98.48%)	972,481,046 (95.55%)	29.61	98.86%	29.95	0.121
Korean Holstein	992,361,054	977,489,671 (98.50%)	942,409,652 (94.97%)	29.48	98.89%	29.82	0.102

Abbreviations: Fold_C, fold coverage; Assembly_C, assembly coverage; Seq_C, sequence coverage; Dup_R, duplication ratio.

a Fold coverage was calculated as the average depth of coverage across the whole genome.

b Assembly coverage was calculated as the proportion of bases in the genome assembly that were covered by at least one read.

c Sequence coverage was computed as the average depth of coverage of the bases that were covered by least one read.

### SNPs

A total of 10,471,178 SNPs were identified throughout the genomes from all three breeds sequenced in this study (HAN: 6,469,804; JJH: 6,484,293; HOL: 5,814,990). Of these SNPs, 5,667,367 (54.12%) were found to be novel (Table 2) using dbSNP build 133 (HAN: 45.8%; JJH: 46.4%; HOL: 44.6%). The proportions of novel SNPs were lower than previous studies [16]–[18]; however, the values still suggest that further sequencing efforts are required to obtain more comprehensive sets of SNPs in cattle. SNP annotation showed that 67.2% of the SNPs were located in intergenic regions (HAN: 67.4%; JJH: 67.1%; HOL: 67.7%), while 25.5% (HAN: 25.5%; JJH: 25.5%; HOL: 25.0%) were located in genic regions, including introns, untranslated regions, and splice sites. All the SNPs detected in this study have been submitted to the dbSNP database under the handle AGL_CJW.

**Table 2:** Functional classification and novelty status of the detected single nucleotide polymorphisms (SNPs) and insertion–deletions (InDels).

SNPs		InDels	
3 prime UTR variant	20,704	3 prime UTR variant	2,533
5 prime UTR variant	3,838	5 prime UTR variant	288
coding sequence variant	50	INTERGENIC	707,901
downstream gene variant^a^	309,055	coding sequence variant	86
initiator codon variant	76	downstream gene variant	33,854
intergenic variant	7,034,568	frameshift variant	859
intron variant	2,669,278	inframe deletion	231
mature miRNA variant	59	inframe insertion	150
missense variant	31,503	intron variant	279,622
nc transcript variant	15	mature miRNA variant	31
non coding exon variant	3,362	missense variant	29
splice acceptor variant	189	nc transcript variant	20
splice donor variant	175	non coding exon variant	213
splice region variant	6,504	splice acceptor variant	56
stop gained	328	splice donor variant	44
stop lost	20	splice region variant	667
stop retained variant	32	stop gained	1
synonymous variant	40,878	upstream gene variant	36,682
upstream gene variant	350,544		
fully known	4,803,811	fully known	224,125
novel	5,667,367	novel	839,142
**Total**	**10,471,178**	**Total**	**1,063,267**

Abbreviations: UTR, untranslated region; nc, non-coding.

a 'Downstream gene variant' indicates variants within 5 kb downstream of the 3' end of a transcript.

The homozygous-to-heterozygous ratios of the breeds were 1∶1.6 for HAN (2,476,336: 3,993,468), 1∶1.5 for JJH (2,560,306: 3,923,987), and 1∶1.6 for HOL (2,212,968: 3,602,022) (Table 3). The fact that the ratio of Jeju Heugu was only slightly higher than those of the other two breeds was somewhat surprising, because Jeju Heugu has been regarded as not only indigenous with a reduced population size but also isolated on Jeju Island, Korea. We further investigated this ratio in the Japanese native cattle breed Kuchinoshima-Ushi using its SNP set from dbSNP. Kuchinoshima-Ushi had a ratio of 1∶1.2, which corresponded to the report of 44.6% to 55.4% in the original paper [18], which was distinctly higher than in the animals we sequenced (Table 3). This result can be explained by the fact that Kuchinoshima-Ushi has long been isolated on a small Kuchinoshima Island and remains highly inbred as well as the lower sequencing depth applied in the original study [18]. The results may suggest that the native Jeju Heugu maintained genetic diversity comparable to cattle breeds with larger population sizes, such as Hanwoo. Interestingly, a recent publication reported that Jeju Heugu had genetic diversity comparable to that of Hanwoo based on mtDNA variation patterns [38].

**Table 3:** Homozygous-to-heterozygous and transition-to-transversion ratios for the single nucleotide polymorphism (SNP) datasets.

Breed	Coverage	No. SNPs	Hom:Het	TS:TV	Seq. Platform	References
**Hanwoo**	25.5×	6.1 M	1∶1.6	2.24∶1	HiSeq 2000	-
**Jeju Heugu**	29.6×	6.1 M	1∶1.5	2.24∶1	HiSeq 2000	-
**Korean Holstein**	29.5×	5.5 M	1∶1.6	2.23∶1	HiSeq 2000	-
**Chikso**	25.3×	5.9 M	1∶1.9	2.24∶1	HiSeq 2000	Choi et al. (2013)
**Kuchinoshima-Ushi**	15.8×	6.3 M	1∶1.2	1.63∶1	Genome Analyzer II	Kawahara-Miki et al. (2011)
**Black Angus**	9.9×	3.2 M	NA	2.24∶1	SOLiD 3	Stothard et al. (2011)
**Goldwyn**	16.5×	3.7 M	NA	2.23∶1	SOLiD 3	Stothard et al. (2011)

Abbreviations: Hom:Het, homozygous-to-heterozygous ratio; TS:TV, transition-to-transversion ratio. Previously sequenced cattle breeds are listed below the dotted line (SNP sets for those breeds were retrieved using dbSNP build 133).

To assess SNP quality, transition-to-transversion (TS/TV) ratios were computed as indicators of possible random sequence errors. The ratios all approximated the empirical human TS/TV ratio >2.1 (HAN: 2.24; JJH: 2.24; HOL: 2.23) (Table 3) [39]. For comparison, we also calculated the TS/TV ratios for the previously sequenced cattle breeds Black Angus [17], Goldwyn [17], Kuchinoshima-Ushi [18] and Chikso [20]. Black Angus and Goldwyn had values similar to the cattle breeds used in this study, but Kuchinoshima-Ushi had a distinctly lower TS/TV ratio (1.63∶1) (Table 3). Because the Kuchinoshima-Ushi study had reasonable depth coverage (∼15.8×) and acceptable quality controls for variant calling [18], we cannot conclude that the lower TS/TV value was due to technical errors in SNP calling. Such distinct values can derive from different coverages or from biological differences in the breeds, therefore further genome sequencing efforts should use a consistent sequencing platform and bioinformatics pipelines for direct comparisons.

The quality of the SNPs from this study was further assured by experimental validation using the commercial Bovine SNP array to genotype the same individuals. The Illumina BovineSNP50K BeadChip exhibited high concordance rates computed by both genotype concordance at variant sites, which measures the overall accuracy of variant genotype calls, and non-reference sensitivity, which evaluates the rate at which non-reference sites in the SNP panel were recovered in the WGS-derived SNPs (see Materials and Methods). The respective values were 99.8% and 99.7% for HAN, 99.9% and 99.6% for JJH, and 99.8% and 99.6% for HOL (Table 4), indicating that most of the SNPs were correctly called from WGS in this study.

**Table 4:** Concordance between single nucleotide polymorphism (SNP) genotypes derived from the BovineSNP50 BeadChip and genotypes from whole-genome sequencing (WGS).

Chip Genotype	No. of chip SNPs			WGS genotype					
	Hanwoo	Jeju Heugu	Korean Holstein	Hanwoo		Jeju Heugu		Korean Holstein	
				A/B	B/B	A/B	B/B	A/B	B/B
A/A	26,330	26,503	24,925	37 (0.3%)	2 (0%)	38 (0.3%)	2 (0%)	42 (0.3%)	1 (0%)
A/B	13,562	13,119	15,340	13,129 (99.2%)	22 (0.2%)	12,763 (99.2%)	7 (0.1%)	14,840 (99.2%)	20 (0.2%)
B/B	13,840	14,063	13,462	41 (0.3%)	13,367 (99.7%)	31 (0.2%)	13,578 (99.6%)	46 (0.3%)	13,022 (99.6%)
./.	143	190	143	27 (0.2%)	21 (0.2%)	34 (0.3%)	42 (0.3%)	32 (0.2%)	32 (0.2%)
**Total**	53,875	53,875	53,870	13,234	13,412	12,866	13,629	14,960	13,075

‘A’, reference allele; ‘B’, non-reference (alternative) allele; ‘.’, no call. Dark gray cells indicate the concordant non-reference genotypes. Light gray cells indicate the discordant non-reference genotypes.

**Figure 2:**
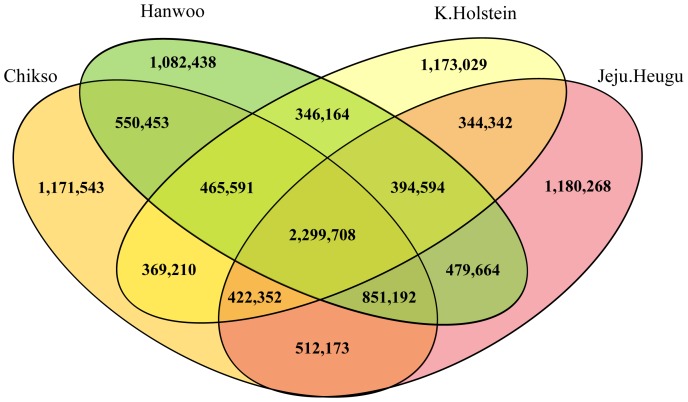
Venn diagram showing the overlap of all detected single nucleotide polymorphisms in the Hanwoo, Jeju Heugu, Chikso, and Korean Holstein genomes.

In addition, we systematically compared all the detected SNP sets, including SNPs from the recent WGS effort for the native Korean Chikso [20]. A total of 11,642,721 SNPs were compared among the four breeds (Fig. 2). The number of breed-specific SNPs (with no overlap with any other breed) was 1,082,438 (16.7% of the breed's SNPs) in Hanwoo, 1,180,268 (18.2%) in Jeju Heugu, 1,171,543 (17.6%) in Chikso, and 1,173,029 (20.2%) in Korean Holstein. The number of SNPs that were shared among all four breeds was 2,299,708, which accounted for approximately 35.5%, 35.5%, 34.6%, and 39.5%, respectively, of the SNPs in each breed. This result met our expectation that substantial numbers of SNPs would be shared, because all four breeds belonged to the same species, *Bos taurus*.

### InDels

**Figure 3:**
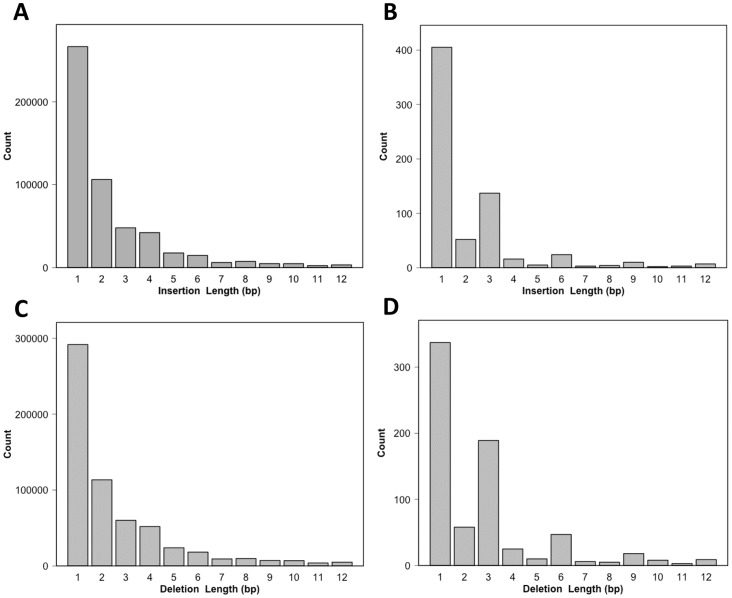
Length distribution of insertions–deletions (InDels) in this study. (A) Total insertion length distribution. (B) Distribution of insertions in coding regions. (C) Total deletion length distribution. (D) Distribution of deletions in coding regions.

InDels remain less explored than SNPs in cattle genomics. In this study, we also investigated InDel events and found a total of 1,063,267 InDels (697,048 in HAN; 702,965 in JJH; 631,332 in HOL), of which 568,069 were deletions. The InDels ranged in length from 31 (insertion) to –49 (deletion). Most InDels were short; approximately 76.5% were less than 4 bp (Fig. 3A, C). The distribution of read depths for all InDels is shown in Fig. S2A. Although the minimum required read depth was 10, approximately 98% of the InDels had at least 20 reads. Furthermore, approximately 98.8% of the InDels had at least five alternative allele reads (Fig. S2B). These results indicated that the InDels detected in this study are well supported by the sequencing data.

Of the InDels, 224,125 (21.09%) were found in the dbSNP database, while the remaining 78.91% were novel. Of the InDels, 707,901 (66.57%) and 279,622 (26.30%) were located in intergenic and intronic regions, respectively (Table 2), and 36,682 (3.45%) and 33,854 (3.18%) were located in the upstream and downstream 5-kb regions, respectively. Only 859 (0.08%) of the InDels were predicted to cause a translational frameshift. Following annotation, we investigated the length distributions of InDels in coding regions compared with all InDels. As shown in Fig. 3B, D, both insertions and deletions in coding regions were enriched for InDels with a 3n length, as has been observed for human data [40]. Such polymorphisms are expected to be more easily tolerated than those inducing frameshifts.

### Copy Number Variation Regions

**Figure 4:**
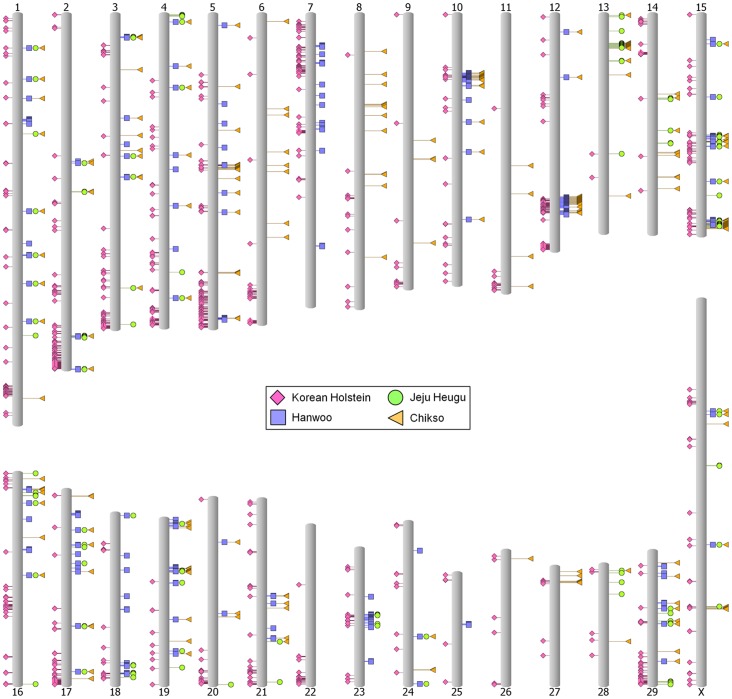
Distribution of copy number variation regions (CNVRs) on the chromosomes. Pink diamonds on the left of each chromosome indicate CNVR gains in Korean Holstein relative to Hanwoo, Jeju Heugu, and Chikso. Blue squares, green circles, and yellow triangles on the right represent CNVR gains in Hanwoo, Jeju Heugu, and Chikso, respectively.

CNVRs were identified across all 29 bovine autosomes and the X chromosome using CNV-seq with the same strict criteria that achieved a high CNVR validation rate (∼82%) in a recent study [26]. For a more extensive CNVR profile of Korean native cattle, we incorporated the recently-sequenced Chikso (CHS) genome in this study. We generated three sets of whole-genome CNVR lists: HANvsHOL, JJHvsHOL, and CHSvsHOL (Tables S1–3). In those comparisons, we identified 992 (16,116,675 bp), 284 (4,748,962 bp), and 1881 (30,802,172 bp) putative CNVRs, respectively, which included 0.61%, 0.18%, and 1.16% of the UMD 3.1 reference genome assembly (Table 5). The detected CNVRs were not evenly distributed throughout the genome (Fig. 4). In particular, a high density of the CNVRs were observed around the telomere regions, and this may be due to the nature of telomeric regions which is highly repetitive. However, we cannot pinpoint an exact reason that would explain the interesting chromosomal distribution of the CNVRs. The median sizes of CNVRs were 13,780, 9,156, and 13,626 bp, with ranges of 7,905–56,253, 5,390–27,428, and 6,324–55,949 bp, respectively (Table 5).

**Table 5:** Distributions and characteristics of putative copy number variation regions (CNVRs) in genome comparisons of Hanwoo, Jeju Heugu, and Chikso with Korean Holstein.

Chr	CNVR length	No. CNVR	Mean length	Median length	Max length	Min length
**1**	423497/234307/1107406	25/19/88	16940/12332/12584	13931/11641/13184	113033/31543/113459	7961/7509/7989
**2**	1430545/182608/3942260	117/15/228	12227/12174/17291	15172/11716/27713	30345/26664/116045	8669/8080/8661
**3**	567832/189211/1419248	32/9/100	17745/21023/14192	15301/15321/18832	78201/58217/58208	8501/8427/8560
**4**	581154/348097/1995544	47/17/120	12365/20476/16630	13974/11781/19849	31236/144401/70295	8220/7601/8269
**5**	1325470/0/3360443	94/0/207	14101/0/16234	17281/0/22160	83809/0/143385	8641/0/8689
**6**	1348103/0/0	103/0/0	13088/0/0	16401/0/0	43201/0/0	8001/0/0
**7**	0/0/2321160	0/0/184	0/0/12615	0/0/18233	0/0/55969	0/0/8481
**8**	559030/0/0	38/0/0	14711/0/0	16857/0/0	34497/0/0	7841/0/0
**9**	333432/0/0	25/0/0	13337/0/0	13051/0/0	42100/0/0	8420/0/0
**10**	446760/0/724501	26/0/49	17183/0/14786	16644/0/16645	61320/0/51685	8760/0/8761
**11**	406054/0/0	32/0/0	12689/0/0	12277/0/0	67529/0/0	8769/0/0
**12**	3331580/0/4110763	120/0/163	27763/0/25219	32328/0/30057	368180/0/270505	8980/0/8841
**13**	341933/313944/0	17/19/0	20114/16523/0	18459/14478/0	51861/36576/0	8789/7620/0
**14**	874982/675824/0	47/32/0	18617/21120/0	17243/18412/0	129924/72923/0	8020/7221/0
**15**	806281/627120/1244340	45/43/80	17917/14584/15554	18627/15276/19624	60317/37788/60656	8869/8040/8920
**16**	246119/225620/1890252	21/16/126	11720/14101/15002	11427/11670/20287	28129/37344/42337	8789/7780/8821
**17**	648440/126732/2228250	53/12/136	12235/10561/16384	14620/10753/21625	33540/13825/110721	8600/7681/8649
**18**	0/192080/646118	0/13/39	0/14775/16567	0/13328/18920	0/44688/103114	0/9408/9460
**19**	272376/195300/357334	17/8/25	16022/24412/14293	13104/12401/15328	31824/55025/74724	9360/7751/9580
**20**	331128/8060/797190	25/1/38	13245/8060/20979	14892/8060/20171	21900/8060/60513	8760/8060/8769
**21**	327646/222530/673727	22/13/51	14893/17118/13210	15769/19250/14128	34165/32340/30905	8761/8470/8829
**22**	52501/0/927690	5/0/62	10500/0/14963	10063/0/16298	14001/0/27311	8749/0/8809
**23**	0/416276/910656	0/24/49	0/17345/18585	0/15181/22950	0/35955/79866	0/7991/9180
**24**	97023/48611/339888	9/3/24	10780/16204/14162	10489/16465/14892	16607/23521/31536	8741/8625/8760
**25**	11064/0/101444	1/0/8	11064/0/12680	11064/0/12799	11064/0/17065	11064/0/9481
**26**	136598/39984/0	12/2/0	11383/19992/0	11531/19992/0	18627/25872/0	8869/14112/0
**27**	313880/0/0	16/0/0	19618/0/0	14629/0/0	95759/0/0	6651/0/0
**28**	139609/47905/0	11/5/0	12692/9581/0	11218/7739/0	27423/16951/0	8309/7369/0
**29**	226060/214520/876288	16/20/74	14129/10726/11842	10963/11881/14592	60481/23761/61440	7561/6601/7680
**X**	537578/440233/827670	16/13/30	33599/33864/27589	26081/29349/30493	98531/97383/98737	14489/13341/14521
**All**	**16116675/4748962/30802172**	**992/284/1881**	**14023/10499/11379**	**13780/9156/13626**	**56253/27428/55949**	**7905/5390/6324**

Comparisons are listed as Hanwoo vs. Holstein/Jeju Heugu vs. Holstein/Chikso vs. Holstein.

We observed distinctly more CNVR gains (more copy numbers) in Korean Holstein than in Hanwoo and Chikso, with 755 (73.4% of all CNVRs) and 1,639 (85.9%) gains in the Holstein in the HANvsHOL and CHSvsHOL comparisons, respectively, but not in the JJHvsHOL comparison (151 CNVRs; 52.7%). Such differences could reflect subtle variations in the preparation of the samples and libraries or different selection histories applied to each breed. Because Holsteins have had a longer and more intensive artificial selection history than Korean native cattle breeds, the greater abundance of CNVR gains in the Holstein may be caused partly by recent strong selection. This result is well coincided with a previous report showing the high copy number gains in Holstein [17]. In the JJHvsHOL genome comparison, there is no distinct abundance observed in CNVR gains as HANvsHOL and CHSvsHOL. It may suggest that Jeju Heugu is a genetically distinct breed from even Hanwoo or Chikso based on the CNVR profile. Also, we cannot rule out the possibility of unrecorded crosses with European-origin cattle before the systematic management of Jeju Heugu. To our knowledge, no genome-wide study has investigated the role of CNVRs in the selection dynamics of cattle; so further studies will be required, particularly at the population level. After annotating the CNVR lists, 574 (9,737,161 bp), 126 (2,358,382 bp), and 1,456 (26,870,724 bp) CNVRs were found to overlap with genes from the HANvsHOL, JJHvsHOL, and CHSvsHOL respectively (Tables S4–6). The abundance pattern of CNVR-gains in Holstein agreed well with these genic CNVR lists: 465 genic CNVRs (75.9% of all genic CNVRs) from HANvsHOL represented gains in Holstein. The number of Holstein genic CNVR-gains for CHSvsHOL and JJHvsHOL were 1346 (90.8% of genic CNVRs) and 70 (47.2% of genic CNVRs), respectively.

### Gene Ontology analysis of nonsynonymous SNPs and genic CNVs

We identified numerous nonsynonymous SNPs (nsSNPs), some of which may account for genetic variation in economically important traits in cattle. Including SNPs from Chikso, we extracted breed-specific nsSNP sets that did not overlap among breeds; we found 3,264, 3,563, 3,459, and 3,573 nsSNPs among 2,080, 2,209, 2,191, and 2,327 genes in Hanwoo, Jeju Heugu, Chikso, and Korean Holstein, respectively. GO enrichment analyses of the 100 genes containing the most nsSNPs for each breed were performed using agriGO [37]. Many of the significantly enriched terms were shared among all four sets of nsSNPs, including “developmental process”, “immune system process”, and “response to stimulus” (Table S7). Hanwoo had several breed-specific GO terms, such as “regulation of biological process”, “biological process”, “cellular process”, “metabolic process”, and “cellular component biogenesis” (Table S7). Interestingly, the GO term “growth” (GO:0040007), defined as the increase in size or mass of an entire organism, a part of an organism, or a cell, was enriched only in the gene sets from Hanwoo and Korean Holstein. For example, one of the genes, cationic amino acid transporter 3-like was present in the enriched sets from both of the two breeds. It is widely known that cationic amino acids are essential for the optimal growth of cattle and can be regulated by cationic amino acid transporter [41], [42]. Both of these breeds have undergone systematic artificial selection for increased growth rate, so our result suggests that nsSNPs in the gene sets associated with "growth" may be involved with this trait.

The GO enrichment analysis was also applied to the genes that overlapped with the genic CNVRs. Each CNVR in this work represented a gain of sequence dosage in one animal relative to the other. Some of the GO terms were commonly enriched in the genic CNVR gains, such as “immune system process”, “cellular component organization”, and “response to stimulus” (Tables S8–10). This result agreed well with those of previous studies showing that immunity and sensory response-related genes are overrepresented in cattle; presumably, the increased gene dosages confer better fitness or these genes have certain properties that cause them to be associated with CNVRs [17], [43]. Compared with Holstein, four GO terms were specifically enriched for gain of genic CNVRs in Hanwoo and Chikso: “regulation of biological process”, “biological adhesion”, “cellular process”, and “metabolic process”. These enriched terms may reflect the selection history of those breeds, but no evidence has yet been published to associate the roles of those CNVRs with any phenotypic characteristic in cattle.

### Genes of interest overlapping with SNPs and CNVRs

By identifying numerous genetic variants, we could instantly locate several promising candidates for further investigations into how the genes are associated with traits of interest in cattle. For example, several nsSNPs occurred in pigmentation-related genes, such as tyrosinase, tyrosinase-related protein 1, dopachrome tautomerase, and melanocortin 1 receptor (*MC1R*) (Table S11). Coat color and pattern in cattle is a main breed characteristic, and it depends on the relative presence of phenomelanin and eumelanin produced by melanocytes [44]. Each of the four breeds in this study has a unique coat color: brown in Hanwoo, black in Jeju Heugu, brindle (tiger-striped) in Chikso, and black and white in Korean Holstein (Fig. 1A–D) *MC1R* is known to have three functional alleles (E^+^, E^D^, and e) and is responsible for the dominant black phenotype [45]. Two nsSNPs in *MC1R* were detected only in Jeju Heugu and Korean Holstein, which both have black coloration (Table S11). The SNP in Jeju Heugu should produce the E^D^ leading to black colour from the Hereford (without black) used to construct the bovine reference sequence assembly. The SNP detected in Holstein should be corresponded with E^D^ locus as well. The brindle pattern in Chikso requires at least one wild-type *MC1R* in the absence of a dominant allele [44], [45], and is consistent with a lack of SNPs in *MC1R* in the Chikso sequence. Because coat color involves multiple genes and remains incompletely understood in cattle, the information provided here should be a useful resource for clarifying its underlying genetic mechanisms in cattle.

**Figure 5:**
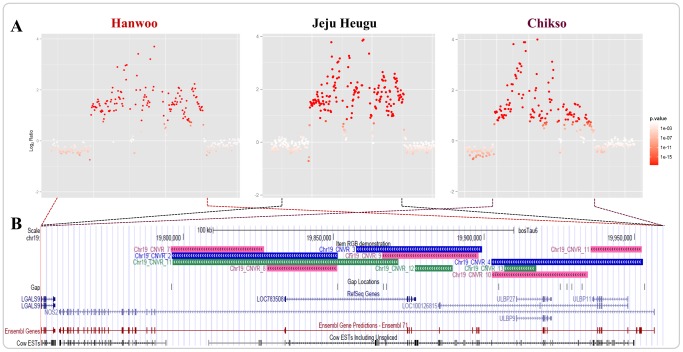
Copy number variation regions (CNVRs) overlapping the *NOS2* gene region. (A) Log_2_ ratio plot of the CNVRs overlapping the *NOS2* gene region for Hanwoo, Jeju Heugu, and Chikso versus Holstein, respectively. Each point presents the log_2_ ratio of the number of reads mapped in Korean Holstein versus the Korean native cattle breed. The color gradient indicates the log_10_ *p*-value obtained from CNV-seq. (B) *NOS2* gene regions in the UCSC Genome Browser. The colors pink, blue, and green indicate genic CNVR gains in Hanwoo, Jeju Heugu, and Chikso, respectively.

Some of the putative CNVRs were found to potentially affect economically important trait-related genes in either beef or dairy cattle. One example is the CNVRs overlapping with inducible nitric oxide synthase 2 (*NOS2*). *NOS2* acts as a mediator in several biological processes, such as growth, development, and involution of the mammary gland [46]. *NOS2* knockout mice showed a delay in involution along with increased levels of prolactin, which is required for alveoli differentiation in pregnancy and milk protein expression during lactation [47]–[49]. Interestingly, the CNVRs overlapping with *NOS2* consistently had fewer copies in Holstein than in the other three Korean native cattle breeds. The estimated differences in copy number were 2.9 (Chr19_CNVR_7-11), 3.6 (Chr19_CNVR_2-4), and 2.4 (Chr19_CNVR_11-13) fewer in Holstein than in Hanwoo, Jeju Heugu, and Chikso, respectively (Fig. 5 and Tables S4-6). While all three Korean native breeds have been widely used as beef cattle, the Korean Holstein bull was a highly influential dairy sire that confers impressive milk performance and is ranked among the top 1% in the international bull evaluation service database. Because such dramatic improvements in milk traits were partly accomplished by intensive selection on the Holstein, *NOS2* could be regarded as a potential candidate for milk production traits in dairy cattle.

### Regions of Homozygosity

**Figure 6:**
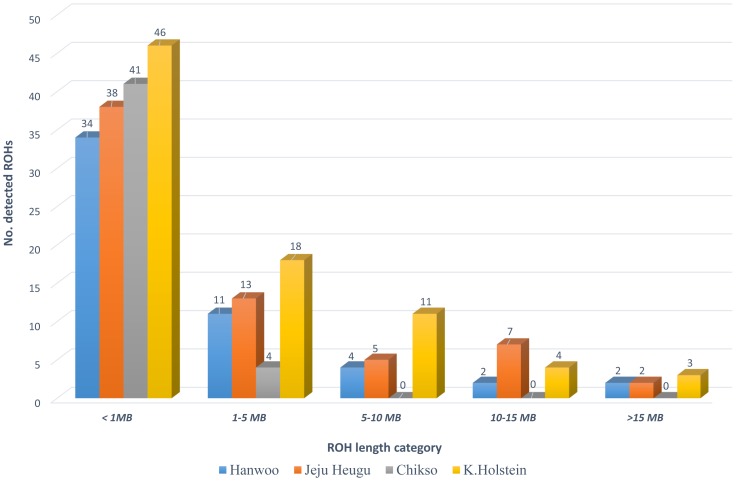
The size distribution of runs of homozygosity (ROHs). The total ROHs in each breed were plotted with respect to the five size categories (<1 MB, 1–5 MB, 5–10 MB, 10–15 MB, and >15 MB). Breeds from left to right in each size category are Hanwoo, Jeju Heugu, Chikso, and Korean Holstein, which are also highlighted with different colors corresponding to the legend in this figure.

A ROH is a continuous stretch of DNA of exhibiting significantly less heterozygosity than the rest of genome. In the present study, ROHs were identified across all 29 bovine autosomes using a previously described method [19]. We generated four sets of ROHs: Hanwoo, Jeju Heugu, Chikso, and Korean Holstein, including 53 (363 400-kb bins), 65 (615 400-kb bins), 45 (68 400-kb bins), and 82 (631 400-kb bins) putative ROHs respectively (Table S12). In addition, we systematically compared all the detected ROHs. A total of 1,395 400-kb bins were compared among the four breeds (Fig. 6). The number of breed-specific 400-kb bins was 248 in Hanwoo, 506 in Jeju Heugu, 54 in Chikso, and 526 in Korean Holstein (Figure S3). The total length of ROHs in Korean Holstein is longer than that of any of the other Korean native breeds in this study. In addition, the size distribution of the ROHs showed that most of the ROHs were included in the shorter size (<5 Mbps) category with ∼85%, ∼78%, 100%, and ∼78% of the total ROHs in Hanwoo, Jeju Heugu, Chikso, and Korean Holstein respectively. Longer ROHs were more frequently identified in Jeju Heugu and Korean Holstein than in Hanwoo and Jeju Heugu (Figure 6). This result may reflect the fact that Holstein has been artificially selected for a longer period of time than the Korean native breeds, and also supports the current concern of inbreeding depression caused by extensive artificial insemination using elite sires in the dairy industry [50]. Among the three native breeds, Jeju Heugu has the longest ROHs, which may be explained by its highly reduced population size isolated in Jeju Island, Korea.

## Conclusions

In this study, we presented extensive genome analyses of three influential cattle breeds in Korea (Hanwoo, Jeju Heugu, and Korean Holstein) using massively parallel sequencing, leading to the identification of numerous SNPs and InDels. Both the high-depth coverage and strict custom filtering applied here allowed us to detect reliable variants; in particular, SNPs were estimated to be highly accurate, as indicated by the approximately 99% concordance rate using the BovineSNP50 BeadChip. Including data from the Chikso breed, we showed that a substantial portion of SNPs overlapped among the cattle breeds and that, while a lower but still significant number of SNPs were breed specific and may contribute to the unique breed characteristics. In addition, compared with Korean Holstein, genome-wide CNVRs were identified in the three Korean native cattle breeds. The Holstein has much higher copy number gain abundance than Hanwoo or Chikso, which may reflect its stronger selection history than the Korean native breeds. However, a distinct difference in abundance was not observed between Jeju Heugu and Holstein. Because so few studies have attempted to associate CNVRs with characteristics of each breed, our results clearly highlight the need for further elucidation of how CNVRs relate to selection history in cattle. Furthermore, ROHs were identified in each of the breeds using genotypes derived from the whole-genome sequence data. Particularly long ROHs were more frequently detected in Jeju Heugu and Holstein possibly due to the reduced population size and the recent inbreeding in Jeju Heugu and Holstein respectively.

We annotated the potential functional role of each variant, which allowed us to identify several candidate variants of functional significance, such as nsSNPs in coat color-related genes as well as CNVRs overlapping with a gene potentially implicated in milk traits in cattle. Because only one animal of each breed was used in this whole-genome sequencing study, further studies are required to elucidate the detailed dynamics of each gene–trait combination. Nonetheless, the results from this study can be used as a valuable resource for further genetic investigations in cattle aimed at finding DNA polymorphisms that account for genetic variations in economically important traits and for the development of accurate genomic tools for cattle breeding.

## Supporting Information

Figure S1 **Distribution of mapped reads on the bovine reference assembly UMD3 for the Hanwoo, Jeju Heugu, and Korean Holstein samples.**
(PDF)

Figure S2 Histograms of InDel characteristics. (A) Read depth. (B) Number of alternative allele reads.
(PDF)

Figure S3 **Venn diagram showing the overlap of all detected ROH in the Hanwoo, Jeju Heugu, Chikso, and Korean Holstein genomes.**
(PDF)

Table S1 **CNVRs detected from HANvsHOL.**
(PDF)

Table S2 **CNVRs detected from JJHvsHOL.**
(PDF)

Table S3 **CNVRs detected from CHSvsHOL.**
(PDF)

Table S4 **Annotated genic-CNVR detected from HANvsHOL.**
(XLSX)

Table S5 **Annotated genic-CNVR detected from JJHvsHOL.**
(XLSX)

Table S6 **Annotated genic-CNVR detected from CHSvsHOL.**
(XLSX)

Table S7 **Gene Ontology terms enriched among the top 100 genes containing the highest number of nsSNPs in each of breed-specific nsSNPs. HAN, JJH, CHS, and HOL indicate Hanwoo, Jeju Heugu, Chikso, and Korean Holstein respectively.**
(PDF)

Table S8 **Gene Ontology terms enriched among the genic-CNVRs from HANvsHOL.**
(PDF)

Table S9 **Gene Ontology terms enriched among the genic-CNVRs from JJHvsHOL.**
(PDF)

Table S10 **Gene Ontology terms enriched among the genic-CNVRs from CHSvsHOL.**
(PDF)

Table S11 **The nsSNPs identified in Hanwoo, Jeju Heugu, Chikso, and Korean Holstein that overlap with pigmentation-related genes.** TYR, TYRP1, DCT, and MC1R indicate tyrosinase, tyrosinase-related protein 1, dopachrome tautomerase, and melanocortin 1 receptor respectively.
(PDF)

Table S12 **Regions of homozygosity (ROHs) detected from Hanwoo, Jeju Heugu, Chikso, and Korean Holstein in this study.**
(PDF)

## References

[1] Jo C, Cho SH, Chang J, Nam KC (2012) Keys to production and processing of Hanwoo beef: A perspective of tradition and science. Anim Frontiers 2: 32–38.

[2] Food and Agriculture Organization (2012) Domestic Animal Diversity Information Service (DAD-IS). Available: http://dad.fao.org/. Accessed 2013 April 21.

[3] National Institute of Animal Science (2012) The status of local livestock breeds in Korea, registered in DAD-IS. Available: http://www.nias.go.kr/. Accessed 2013 April 21.

[4] Choi TJ (2009) Establishment of phylogenomic characteristics for Korean traditional cattle breeds (Hanwoo, Korean brindle and black). Doctoral Thesis. Jeon-buk National University. Available: http://www.riss.kr/. Accessed 2013 April 21.

[5] National Institute of Animal Science (NIAS) (2011) Annual report for Hanwoo genetic evaluation. In: Annual report for livestock improvement in 2010. Available: http://www.nias.go.kr/. Accessed 2013 April 21.

[6] Ministry for Food, Agriculture Forestry and Fisheries (2013) 2012 Dairy Herd Improvement annual report in Korea, Republic of Korea. Available: http://rd.dcic.co.kr/. Accessed 2013 April 21.

[7] Bovine Genome S, Analysis C, Elsik CG, Tellam RL, Worley KC, et al (2009) The genome sequence of taurine cattle: a window to ruminant biology and evolution. Science 324: 522–528. 19390049 10.1126/science.1169588 PMC2943200

[8] Bovine HapMap C, Gibbs RA, Taylor JF, Van Tassell CP, Barendse W, et al (2009) Genome-wide survey of SNP variation uncovers the genetic structure of cattle breeds. Science 324: 528–532. 19390050 10.1126/science.1167936 PMC2735092

[9] Matukumalli LK, Lawley CT, Schnabel RD, Taylor JF, Allan MF, et al (2009) Development and characterization of a high density SNP genotyping assay for cattle. PLoS One 4: e5350. 19390634 10.1371/journal.pone.0005350 PMC2669730

[10] Barendse W, Harrison BE, Bunch RJ, Thomas MB, Turner LB (2009) Genome wide signatures of positive selection: the comparison of independent samples and the identification of regions associated to traits. BMC Genomics 10: 178. 19393047 10.1186/1471-2164-10-178 PMC2681478

[11] Qanbari S, Gianola D, Hayes B, Schenkel F, Miller S, et al (2011) Application of site and haplotype-frequency based approaches for detecting selection signatures in cattle. BMC Genomics 12: 318. 21679429 10.1186/1471-2164-12-318 PMC3146955

[12] Kolbehdari D, Wang Z, Grant JR, Murdoch B, Prasad A, et al (2009) A whole genome scan to map QTL for milk production traits and somatic cell score in Canadian Holstein bulls. J Anim Breed Genet 126: 216–227. 19646150 10.1111/j.1439-0388.2008.00793.x

[13] Sherman EL, Nkrumah JD, Moore SS (2010) Whole genome single nucleotide polymorphism associations with feed intake and feed efficiency in beef cattle. J Anim Sci 88: 16–22. 19749024 10.2527/jas.2008-1759

[14] Bolormaa S, Hayes BJ, Savin K, Hawken R, Barendse W, et al (2011) Genome-wide association studies for feedlot and growth traits in cattle. J Anim Sci 89: 1684–1697. 21239664 10.2527/jas.2010-3079

[15] Jiang L, Liu J, Sun D, Ma P, Ding X, et al (2010) Genome wide association studies for milk production traits in Chinese Holstein population. PLoS One 5: e13661. 21048968 10.1371/journal.pone.0013661 PMC2965099

[16] Eck SH, Benet-Pages A, Flisikowski K, Meitinger T, Fries R, et al (2009) Whole genome sequencing of a single Bos taurus animal for single nucleotide polymorphism discovery. Genome Biol 10: R82. 19660108 10.1186/gb-2009-10-8-r82 PMC2745763

[17] Stothard P, Choi JW, Basu U, Sumner-Thomson JM, Meng Y, et al (2011) Whole genome resequencing of black Angus and Holstein cattle for SNP and CNV discovery. BMC Genomics 12: 559. 22085807 10.1186/1471-2164-12-559 PMC3229636

[18] Kawahara-Miki R, Tsuda K, Shiwa Y, Arai-Kichise Y, Matsumoto T, et al (2011) Whole-genome resequencing shows numerous genes with nonsynonymous SNPs in the Japanese native cattle Kuchinoshima-Ushi. BMC Genomics 12: 103. 21310019 10.1186/1471-2164-12-103 PMC3048544

[19] Lee KT, Chung WH, Lee SY, Choi JW, Kim J, et al (2013) Whole-genome resequencing of Hanwoo (Korean cattle) and insight into regions of homozygosity. BMC Genomics 14: 519. 23899338 10.1186/1471-2164-14-519 PMC3750754

[20] Choi JW, Liao X, Park S, Jeon HJ, Chung WH, et al (2013) Massively parallel sequencing of Chikso (Korean brindle cattle) to discover genome-wide SNPs and InDels. Mol Cells 36: 203–211. 23912596 10.1007/s10059-013-2347-0 PMC3887973

[21] Bae JS, Cheong HS, Kim LH, NamGung S, Park TJ, et al (2010) Identification of copy number variations and common deletion polymorphisms in cattle. BMC Genomics 11: 232. 20377913 10.1186/1471-2164-11-232 PMC2859865

[22] Hou Y, Liu GE, Bickhart DM, Cardone MF, Wang K, et al (2011) Genomic characteristics of cattle copy number variations. BMC Genomics 12: 127. 21345189 10.1186/1471-2164-12-127 PMC3053260

[23] Jiang L, Jiang J, Wang J, Ding X, Liu J, et al (2012) Genome-wide identification of copy number variations in Chinese Holstein. PLoS One 7: e48732. 23144949 10.1371/journal.pone.0048732 PMC3492429

[24] Hou Y, Bickhart DM, Hvinden ML, Li C, Song J, et al (2012) Fine mapping of copy number variations on two cattle genome assemblies using high density SNP array. BMC Genomics 13: 376. 22866901 10.1186/1471-2164-13-376 PMC3583728

[25] Bickhart DM, Hou Y, Schroeder SG, Alkan C, Cardone MF, et al (2012) Copy number variation of individual cattle genomes using next-generation sequencing. Genome Res 22: 778–790. 22300768 10.1101/gr.133967.111 PMC3317159

[26] Choi JW, Lee KT, Liao X, Stothard P, An HS, et al (2013) Genome-wide copy number variation in Hanwoo, Black Angus, and Holstein cattle. Mamm Genome 24: 151–163. 23543395 10.1007/s00335-013-9449-z

[27] Zimin AV, Delcher AL, Florea L, Kelley DR, Schatz MC, et al (2009) A whole-genome assembly of the domestic cow, Bos taurus. Genome Biol 10: R42. 19393038 10.1186/gb-2009-10-4-r42 PMC2688933

[28] Li H, Durbin R (2009) Fast and accurate short read alignment with Burrows-Wheeler transform. Bioinformatics 25: 1754–1760. 19451168 10.1093/bioinformatics/btp324 PMC2705234

[29] McKenna A, Hanna M, Banks E, Sivachenko A, Cibulskis K, et al (2010) The Genome Analysis Toolkit: a MapReduce framework for analyzing next-generation DNA sequencing data. Genome Res 20: 1297–1303. 20644199 10.1101/gr.107524.110 PMC2928508

[30] Li H, Handsaker B, Wysoker A, Fennell T, Ruan J, et al (2009) The Sequence Alignment/Map format and SAMtools. Bioinformatics 25: 2078–2079. 19505943 10.1093/bioinformatics/btp352 PMC2723002

[31] Grant JR, Arantes AS, Liao X, Stothard P (2011) In-depth annotation of SNPs arising from resequencing projects using NGS-SNP. Bioinformatics 27: 2300–2301. 21697123 10.1093/bioinformatics/btr372 PMC3150039

[32] Flicek P, Amode MR, Barrell D, Beal K, Brent S, et al (2012) Ensembl 2012. Nucleic Acids Res 40: D84–90. 22086963 10.1093/nar/gkr991 PMC3245178

[33] Sayers EW, Barrett T, Benson DA, Bolton E, Bryant SH, et al (2012) Database resources of the National Center for Biotechnology Information. Nucleic Acids Res 40: D13–25. 22140104 10.1093/nar/gkr1184 PMC3245031

[34] UniProt C (2013) Update on activities at the Universal Protein Resource (UniProt) in 2013. Nucleic Acids Res 41: D43–47. 23161681 10.1093/nar/gks1068 PMC3531094

[35] Xie C, Tammi MT (2009) CNV-seq, a new method to detect copy number variation using high-throughput sequencing. BMC Bioinformatics 10: 80. 19267900 10.1186/1471-2105-10-80 PMC2667514

[36] Haider S, Ballester B, Smedley D, Zhang J, Rice P, et al (2009) BioMart Central Portal—unified access to biological data. Nucleic Acids Res 37: W23–27. 19420058 10.1093/nar/gkp265 PMC2703988

[37] Du Z, Zhou X, Ling Y, Zhang Z, Su Z (2010) agriGO: a GO analysis toolkit for the agricultural community. Nucleic Acids Res 38: W64–70. 20435677 10.1093/nar/gkq310 PMC2896167

[38] Dadi H, Lee SH, Jung KS, Choi JW, Ko MS, et al (2012) Effect of population reduction on mtDNA diversity and demographic history of Korean Cattle populations. Asian Austral J Anim 25: 1223–1228. 10.5713/ajas.2012.12122 PMC4092938 25049684

[39] Genomes Project C, Abecasis GR, Auton A, Brooks LD, DePristo MA, et al (2012) An integrated map of genetic variation from 1,092 human genomes. Nature 491: 56–65. 23128226 10.1038/nature11632 PMC3498066

[40] Fujimoto A, Nakagawa H, Hosono N, Nakano K, Abe T, et al (2010) Whole-genome sequencing and comprehensive variant analysis of a Japanese individual using massively parallel sequencing. Nat Genet 42: 931–936. 20972442 10.1038/ng.691

[41] Merchen NR, Titgemeyer EC (1992) Manipulation of amino acid supply to the growing ruminant. J Anim Sci 70: 3238–3247. 1429300 10.2527/1992.70103238x

[42] Liao SF, Vanzant ES, Harmon DL, McLeod KR, Boling JA, et al (2009) Ruminal and abomasal starch hydrolysate infusions selectively decrease the expression of cationic amino acid transporter mRNA by small intestinal epithelia of forage-fed beef steers. J Dairy Sci 92: 1124–1135. 19233805 10.3168/jds.2008-1521

[43] Liu GE, Hou Y, Zhu B, Cardone MF, Jiang L, et al (2010) Analysis of copy number variations among diverse cattle breeds. Genome Res 20: 693–703. 20212021 10.1101/gr.105403.110 PMC2860171

[44] Seo K, Mohanty TR, Choi T, Hwang I (2007) Biology of epidermal and hair pigmentation in cattle: a mini-review. Vet Dermatol 18: 392–400. 17991156 10.1111/j.1365-3164.2007.00634.x

[45] Klungland H, Vage DI, Gomez-Raya L, Adalsteinsson S, Lien S (1995) The role of melanocyte-stimulating hormone (MSH) receptor in bovine coat color determination. Mamm Genome 6: 636–639. 8535072 10.1007/BF00352371

[46] Iizuka T, Sasaki M, Oishi K, Uemura S, Koike M (1998) The presence of nitric oxide synthase in the mammary glands of lactating rats. Pediatr Res 44: 197–200. 9702914 10.1203/00006450-199808000-00010

[47] Zaragoza R, Miralles VJ, Rus AD, Garcia C, Carmena R, et al (2005) Weaning induces NOS-2 expression through NF-kappaB modulation in the lactating mammary gland: importance of GSH. Biochem J 391: 581–588. 15954866 10.1042/BJ20050507 PMC1276959

[48] Oakes SR, Rogers RL, Naylor MJ, Ormandy CJ (2008) Prolactin regulation of mammary gland development. J Mammary Gland Biol Neoplasia 13: 13–28. 18219564 10.1007/s10911-008-9069-5

[49] Zaragoza R, Bosch A, Garcia C, Sandoval J, Serna E, et al (2010) Nitric oxide triggers mammary gland involution after weaning: remodelling is delayed but not impaired in mice lacking inducible nitric oxide synthase. Biochem J 428: 451–462. 20345368 10.1042/BJ20091091

[50] Mc Parland S, Kearney JF, Rath M, Berry DP (2007) Inbreeding effects on milk production, calving performance, fertility, and conformation in Irish Holstein-Friesians. J Dairy Sci 90: 4411–4419. 17699061 10.3168/jds.2007-0227

