# Supplementary material for: Whole-Genome Analyses of Korean Native and Holstein Cattle Breeds by Massively Parallel Sequencing
Source: PLoS One. 2014 Jul 3;9(7):e101127. doi: 10.1371/journal.pone.0101127 (PMC4081042; doi:10.1371/journal.pone.0101127)

Supplementary Fig. S1. Distribution of mapped reads on the bovine reference assembly UMD3 for the Hanwoo, Jeju Heugu, and Korean Holstein samples.

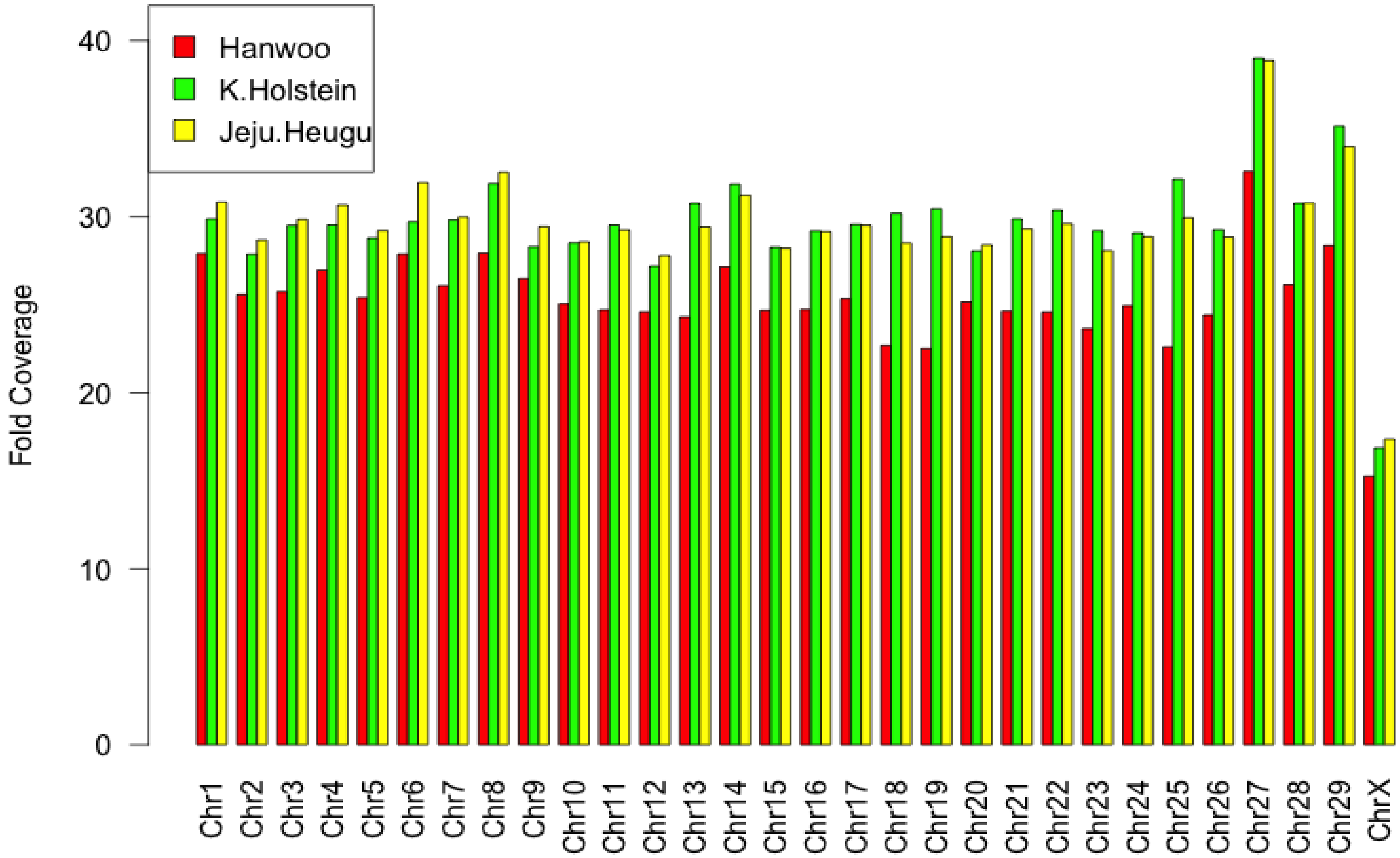

Supplement: Figure S1 — Distribution of mapped reads on the bovine reference assembly UMD3 for the Hanwoo, Jeju Heugu, and Korean Holstein samples. (PDF) [file pone.0101127.s001.pdf]
