# Supplementary material for: Whole-Genome Analyses of Korean Native and Holstein Cattle Breeds by Massively Parallel Sequencing
Source: PLoS One. 2014 Jul 3;9(7):e101127. doi: 10.1371/journal.pone.0101127 (PMC4081042; doi:10.1371/journal.pone.0101127)

Supplementary Fig. S2. Histograms of InDel characteristics. (A) Read depth. (B) Number of alternative allele reads.

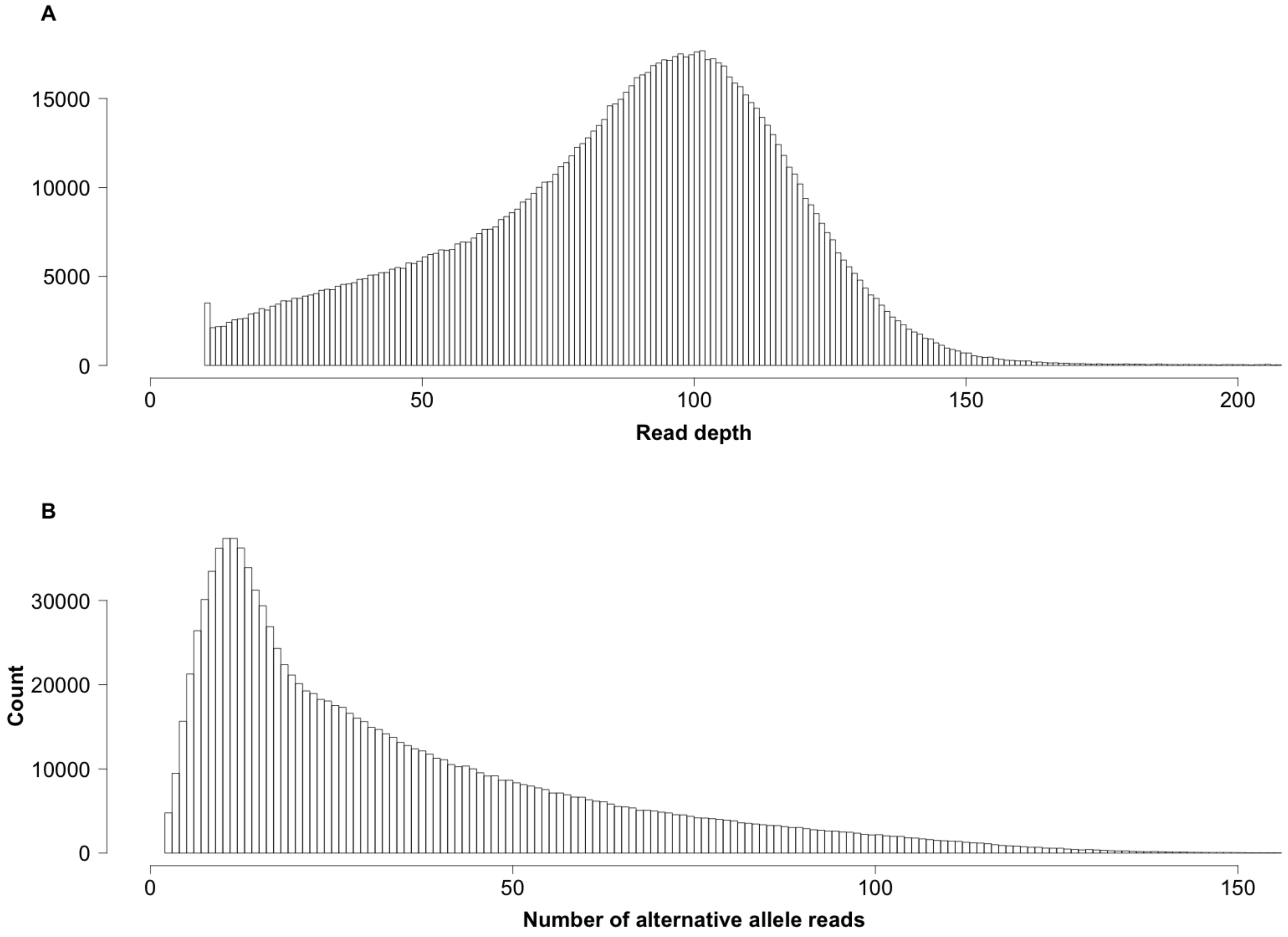

Supplement: Figure S2 — Histograms of InDel characteristics. (A) Read depth. (B) Number of alternative allele reads. (PDF) [file pone.0101127.s002.pdf]
