# Supplementary material for: Whole-Genome Analyses of Korean Native and Holstein Cattle Breeds by Massively Parallel Sequencing
Source: PLoS One. 2014 Jul 3;9(7):e101127. doi: 10.1371/journal.pone.0101127 (PMC4081042; doi:10.1371/journal.pone.0101127)

Supplementary Fig. S3. Venn diagram showing the overlap of all detected ROH in the Hanwoo, Jeju Heugu, Chikso, and Korean Holstein genomes.

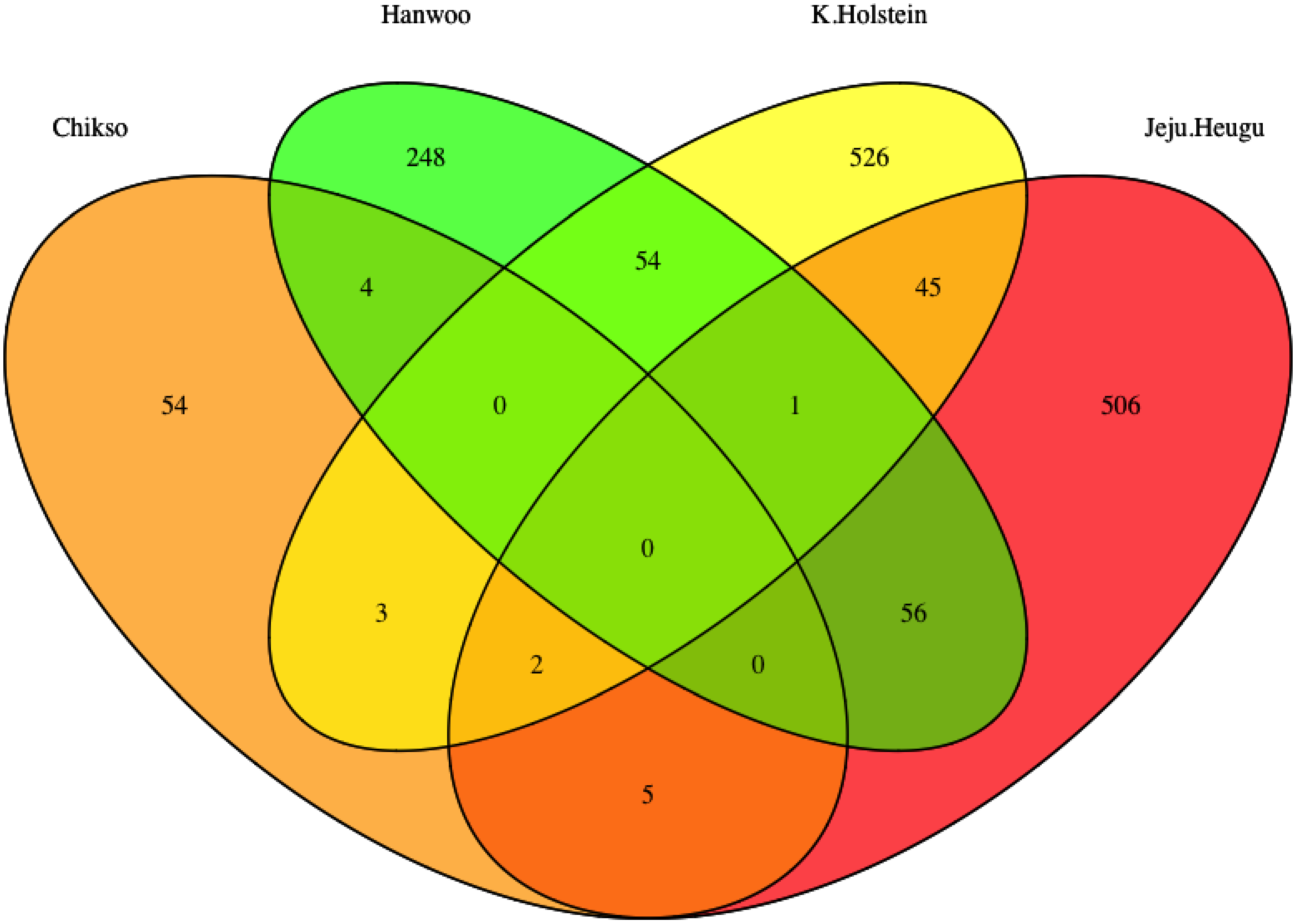

Supplement: Figure S3 — Venn diagram showing the overlap of all detected ROH in the Hanwoo, Jeju Heugu, Chikso, and Korean Holstein genomes. (PDF) [file pone.0101127.s003.pdf]
