# Supplementary material for: Whole-Genome Analyses of Korean Native and Holstein Cattle Breeds by Massively Parallel Sequencing
Source: PLoS One. 2014 Jul 3;9(7):e101127. doi: 10.1371/journal.pone.0101127 (PMC4081042; doi:10.1371/journal.pone.0101127)
Supplement: Table S1 — CNVRs detected from HANvsHOL. (PDF) [file pone.0101127.s004.pdf]

Supplementary Table S1. CNVRs detected from HANvsHOL

cnv_name	chr	start	end	size	log2	Ratio	pvalue	Hanwoo	Holstein
Chr1_CNVR_10	Chr1	1414891	1424443	9553	-1.43895	0.368836	0	Loss	Gain
Chr1_CNVR_11	Chr1	2309595	2319943	10349	-0.8086313	0.570923	8.57E-200	Loss	Gain
Chr1_CNVR_1	Chr1	12913111	12926643	13533	1.049404	2.06967	0	Gain	Loss
Chr1_CNVR_12	Chr1	12926643	12938583	11941	-1.679722	0.312143	0	Loss	Gain
Chr1_CNVR_13	Chr1	16020695	16028655	7961	-3.860569	0.0688419	0	Loss	Gain
Chr1_CNVR_2	Chr1	24985247	24997187	11941	1.027238	2.03812	0	Gain	Loss
Chr1_CNVR_14	Chr1	26473767	26506403	32637	-1.116228	0.461298	0	Loss	Gain
Chr1_CNVR_3	Chr1	32403171	32411131	7961	0.8564077	1.81052	1.84E-176	Gain	Loss
Chr1_CNVR_15	Chr1	42132679	42141435	8757	-1.170738	0.444194	0	Loss	Gain
Chr1_CNVR_4	Chr1	46184319	46193075	8757	1.742877	3.34702	0	Gain	Loss
Chr1_CNVR_16	Chr1	57647515	57659455	11941	-1.090717	0.469528	0	Loss	Gain
Chr1_CNVR_17	Chr1	68039295	68047255	7961	-0.8103049	0.570261	1.03E-154	Loss	Gain
Chr1_CNVR_18	Chr1	69204639	69222947	18309	-1.203864	0.434111	0	Loss	Gain
Chr1_CNVR_5	Chr1	76010439	76018399	7961	1.204884	2.30519	0	Gain	Loss
Chr1_CNVR_6	Chr1	93011407	93021755	10349	2.111294	4.32079	0	Gain	Loss
Chr1_CNVR_19	Chr1	93717459	93830491	113033	-5.674269	0.0195828	0	Loss	Gain
Chr1_CNVR_20	Chr1	97519951	97544627	24677	-1.046916	0.484002	0	Loss	Gain
Chr1_CNVR_7	Chr1	104072623	104082971	10349	2.175971	4.5189	0	Gain	Loss
Chr1_CNVR_8	Chr1	118624299	118636239	11941	1.132338	2.19214	0	Gain	Loss
Chr1_CNVR_21	Chr1	143523179	143537507	14329	-0.8190849	0.566801	4.82E-282	Loss	Gain
Chr1_CNVR_22	Chr1	145065031	145073787	8757	-0.8057543	0.572063	2.48E-168	Loss	Gain
Chr1_CNVR_23	Chr1	145823619	145835559	11941	-0.8341283	0.560922	6.49E-243	Loss	Gain
Chr1_CNVR_24	Chr1	146493851	146508179	14329	-0.8506287	0.554543	4.68E-301	Loss	Gain
Chr1_CNVR_25	Chr1	146696035	146704791	8757	-0.8217477	0.565756	3.76E-174	Loss	Gain
Chr1_CNVR_9	Chr1	148426539	148452011	25473	1.161778	2.23733	0	Gain	Loss
Chr10_CNVR_1	Chr10	22586347	22606494	20148	1.520303	2.86851	0	Gain	Loss
Chr10_CNVR_2	Chr10	22708111	22736142	28032	2.336524	5.05084	0	Gain	Loss
Chr10_CNVR_3	Chr10	23301163	23313426	12264	1.436241	2.70615	0	Gain	Loss
Chr10_CNVR_17	Chr10	23324815	23346714	21900	-1.018894	0.493495	0	Loss	Gain
Chr10_CNVR_18	Chr10	23369491	23430810	61320	-2.529629	0.173183	0	Loss	Gain
Chr10_CNVR_4	Chr10	23668207	23683098	14892	5.945921	61.6454	0	Gain	Loss
Chr10_CNVR_5	Chr10	23685727	23715510	29784	3.683192	12.8455	0	Gain	Loss
Chr10_CNVR_19	Chr10	23716387	23734782	18396	-1.918513	0.264527	0	Loss	Gain
Chr10_CNVR_6	Chr10	23888959	23897718	8760	0.9352845	1.91227	8.40E-212	Gain	Loss
Chr10_CNVR_7	Chr10	24006343	24015102	8760	1.0258	2.03609	1.46E-247	Gain	Loss
Chr10_CNVR_20	Chr10	24117595	24133362	15768	-0.9192562	0.528782	0	Loss	Gain
Chr10_CNVR_8	Chr10	24344479	24360246	15768	0.8846552	1.84632	0	Gain	Loss
Chr10_CNVR_9	Chr10	24785107	24807006	21900	1.533146	2.89416	0	Gain	Loss
Chr10_CNVR_21	Chr10	25011115	25021626	10512	-0.9996911	0.500107	4.89E-263	Loss	Gain
Chr10_CNVR_22	Chr10	25061047	25075938	14892	-1.036728	0.487432	0	Loss	Gain
Chr10_CNVR_10	Chr10	25103095	25116234	13140	1.801027	3.48468	0	Gain	Loss
Chr10_CNVR_11	Chr10	25156531	25173174	16644	2.56611	5.9221	0	Gain	Loss
Chr10_CNVR_12	Chr10	27364051	27372810	8760	2.201696	4.6002	0	Gain	Loss
Chr10_CNVR_13	Chr10	27634735	27660138	25404	1.291688	2.44814	0	Gain	Loss
Chr10_CNVR_23	Chr10	32527195	32537706	10512	-1.687531	0.310458	0	Loss	Gain
Chr10_CNVR_14	Chr10	41589415	41599050	9636	1.379538	2.60185	0	Gain	Loss
Chr10_CNVR_15	Chr10	53140351	53155242	14892	1.15841	2.23211	0	Gain	Loss
Chr10_CNVR_24	Chr10	55580887	55590522	9636	-3.606443	0.0821018	0	Loss	Gain
Chr10_CNVR_16	Chr10	79246903	79263546	16644	1.332628	2.51861	0	Gain	Loss
Chr10_CNVR_25	Chr10	81029563	81038322	8760	-0.8352986	0.560467	8.56E-163	Loss	Gain
Chr10_CNVR_26	Chr10	96710839	96720474	9636	-2.290766	0.204367	0	Loss	Gain
Chr11_CNVR_5	Chr11	36297716	36308238	10523	-6.82017	0.00884961	0	Loss	Gain

Chr11_CNVR_1	Chr11	58444596	58459504	14909	1.102812	2.14773	0	Gain	Loss
Chr11_CNVR_2	Chr11	69356230	69378154	21925	1.156385	2.22898	0	Gain	Loss
Chr11_CNVR_3	Chr11	93517580	93526350	8771	1.691107	3.22904	0	Gain	Loss
Chr11_CNVR_6	Chr11	99327706	99395234	67529	-1.521572	0.348306	0	Loss	Gain
Chr11_CNVR_7	Chr11	101203608	101212378	8771	-0.7931926	0.577066	8.71E-149	Loss	Gain
Chr11_CNVR_8	Chr11	103807422	103817068	9647	-0.9095568	0.532349	5.12E-206	Loss	Gain
Chr11_CNVR_9	Chr11	103891614	103900382	8769	-0.8515886	0.554174	6.50E-168	Loss	Gain
Chr11_CNVR_10	Chr11	104035442	104046842	11401	-0.9812349	0.506546	2.28E-275	Loss	Gain
Chr11_CNVR_11	Chr11	104061752	104071398	9647	-0.8151192	0.568362	2.81E-171	Loss	Gain
Chr11_CNVR_12	Chr11	104099462	104114370	14909	-0.8412499	0.55816	3.76E-278	Loss	Gain
Chr11_CNVR_13	Chr11	104311696	104320466	8771	-0.8286283	0.563064	2.56E-160	Loss	Gain
Chr11_CNVR_14	Chr11	104437984	104448508	10525	-0.9020836	0.535113	1.71E-221	Loss	Gain
Chr11_CNVR_15	Chr11	104457278	104466048	8771	-0.8252405	0.564388	3.32E-159	Loss	Gain
Chr11_CNVR_16	Chr11	104485342	104495866	10525	-0.9826502	0.506049	6.03E-255	Loss	Gain
Chr11_CNVR_17	Chr11	104522176	104530946	8771	-0.8598362	0.551015	1.15E-170	Loss	Gain
Chr11_CNVR_18	Chr11	104533578	104547608	14031	-0.8727144	0.546118	1.04E-278	Loss	Gain
Chr11_CNVR_19	Chr11	104593214	104606368	13155	-0.8552135	0.552784	1.23E-252	Loss	Gain
Chr11_CNVR_20	Chr11	104639694	104649340	9647	-0.9355099	0.522858	7.95E-216	Loss	Gain
Chr11_CNVR_21	Chr11	104707224	104716870	9647	-1.130859	0.456644	4.66E-292	Loss	Gain
Chr11_CNVR_22	Chr11	104725640	104735286	9647	-0.8389458	0.559052	6.92E-180	Loss	Gain
Chr11_CNVR_23	Chr11	104801062	104811586	10525	-0.8633759	0.549665	8.37E-206	Loss	Gain
Chr11_CNVR_24	Chr11	104851928	104861574	9647	-0.7935025	0.576942	1.43E-163	Loss	Gain
Chr11_CNVR_25	Chr11	104891394	104901040	9647	-0.8819829	0.542621	1.04E-195	Loss	Gain
Chr11_CNVR_4	Chr11	105125552	105134322	8771	2.031259	4.08761	0	Gain	Loss
Chr11_CNVR_26	Chr11	105682448	105694724	12277	-0.8357809	0.56028	6.51E-227	Loss	Gain
Chr11_CNVR_27	Chr11	106232326	106246358	14033	-0.8815878	0.54277	1.72E-283	Loss	Gain
Chr11_CNVR_28	Chr11	106313888	106322656	8769	-0.9844574	0.505416	1.92E-213	Loss	Gain
Chr11_CNVR_29	Chr11	106354230	106365630	11401	-0.8933305	0.53837	6.62E-236	Loss	Gain
Chr11_CNVR_30	Chr11	106540154	106552430	12277	-0.8621783	0.550121	3.43E-239	Loss	Gain
Chr11_CNVR_31	Chr11	106624346	106633114	8769	-0.8703248	0.547024	3.53E-174	Loss	Gain
Chr11_CNVR_32	Chr11	106650656	106660302	9647	-0.8845842	0.541644	1.12E-196	Loss	Gain
Chr12_CNVR_24	Chr12	936166	946043	9878	-4.122146	0.0574262	0	Loss	Gain
Chr12_CNVR_1	Chr12	6719286	6728265	8980	0.8250487	1.77159	7.08E-168	Gain	Loss
Chr12_CNVR_25	Chr12	9814692	9834447	19756	-0.9571148	0.515086	0	Loss	Gain
Chr12_CNVR_2	Chr12	24262614	24273389	10776	0.9204577	1.89272	3.50E-243	Gain	Loss
Chr12_CNVR_26	Chr12	32023130	32040191	17062	-1.191187	0.437942	0	Loss	Gain
Chr12_CNVR_27	Chr12	32041090	32056355	15266	-1.163466	0.446439	0	Loss	Gain
Chr12_CNVR_28	Chr12	32086888	32096765	9878	-1.021449	0.492621	2.49E-250	Loss	Gain
Chr12_CNVR_29	Chr12	32111134	32142563	31430	-0.9498852	0.517674	0	Loss	Gain
Chr12_CNVR_30	Chr12	32156034	32189259	33226	-1.021228	0.492697	0	Loss	Gain
Chr12_CNVR_31	Chr12	41376698	41388371	11674	-3.782475	0.0726711	0	Loss	Gain
Chr12_CNVR_3	Chr12	70296788	70310257	13470	3.596825	12.0991	0	Gain	Loss
Chr12_CNVR_4	Chr12	70641620	70653293	11674	3.734793	13.3133	0	Gain	Loss
Chr12_CNVR_5	Chr12	70817628	70826607	8980	2.561492	5.90318	0	Gain	Loss
Chr12_CNVR_6	Chr12	70827506	70910121	82616	3.957161	15.5319	0	Gain	Loss
Chr12_CNVR_7	Chr12	70911918	70977471	65554	3.970727	15.6786	0	Gain	Loss
Chr12_CNVR_8	Chr12	70978370	70999023	20654	3.323052	10.0078	0	Gain	Loss
Chr12_CNVR_9	Chr12	71000820	71009799	8980	3.504576	11.3497	0	Gain	Loss
Chr12_CNVR_10	Chr12	71041230	71149887	108658	4.944914	30.8012	0	Gain	Loss
Chr12_CNVR_11	Chr12	71167848	71214543	46696	2.919148	7.56399	0	Gain	Loss
Chr12_CNVR_32	Chr12	71259444	71283689	24246	-1.516125	0.349624	0	Loss	Gain
Chr12_CNVR_33	Chr12	71284588	71346549	61962	-1.76356	0.294521	0	Loss	Gain
Chr12_CNVR_34	Chr12	71691382	71706647	15266	-1.095321	0.468032	0	Loss	Gain
Chr12_CNVR_35	Chr12	71708444	71733587	25144	-1.444223	0.36749	0	Loss	Gain
Chr12_CNVR_36	Chr12	71734486	71783875	49390	-1.591141	0.331909	0	Loss	Gain
Chr12_CNVR_37	Chr12	71784774	71895227	110454	-2.729543	0.150774	0	Loss	Gain

Chr12_CNVR_38	Chr12	71915882	71927555	11674	-0.9887806	0.503904	3.10E-280	Loss	Gain
Chr12_CNVR_39	Chr12	72029030	72051479	22450	-4.517306	0.0436672	0	Loss	Gain
Chr12_CNVR_40	Chr12	72053276	72064051	10776	-1.623926	0.324451	0	Loss	Gain
Chr12_CNVR_12	Chr12	72174506	72187077	12572	1.000595	2.00083	0	Gain	Loss
Chr12_CNVR_41	Chr12	72205936	72234671	28736	-1.558274	0.339557	0	Loss	Gain
Chr12_CNVR_13	Chr12	72245448	72257121	11674	0.9341926	1.91082	4.93E-270	Gain	Loss
Chr12_CNVR_14	Chr12	72258918	72267897	8980	0.9560753	1.94003	1.44E-216	Gain	Loss
Chr12_CNVR_15	Chr12	72744736	72768981	24246	5.659184	50.5341	0	Gain	Loss
Chr12_CNVR_16	Chr12	72769880	72826453	56574	5.233499	37.6219	0	Gain	Loss
Chr12_CNVR_17	Chr12	72828250	72892007	63758	2.282084	4.8638	0	Gain	Loss
Chr12_CNVR_18	Chr12	72892906	72912661	19756	4.144003	17.6795	0	Gain	Loss
Chr12_CNVR_42	Chr12	72919846	72955765	35920	-2.12525	0.229211	0	Loss	Gain
Chr12_CNVR_43	Chr12	72956664	72967439	10776	-1.6855	0.310895	0	Loss	Gain
Chr12_CNVR_44	Chr12	72971032	72998869	27838	-1.813287	0.284542	0	Loss	Gain
Chr12_CNVR_45	Chr12	73017728	73028503	10776	-1.377312	0.384935	0	Loss	Gain
Chr12_CNVR_46	Chr12	73029402	73077893	48492	-1.94234	0.260194	0	Loss	Gain
Chr12_CNVR_47	Chr12	73078792	73102139	23348	-1.14406	0.452484	0	Loss	Gain
Chr12_CNVR_48	Chr12	73103936	73116507	12572	-0.9462144	0.518993	1.59E-280	Loss	Gain
Chr12_CNVR_49	Chr12	73125488	73192837	67350	-1.738212	0.299741	0	Loss	Gain
Chr12_CNVR_50	Chr12	73196430	73217083	20654	-1.242361	0.42268	0	Loss	Gain
Chr12_CNVR_51	Chr12	73218880	73229655	10776	-1.03014	0.489663	1.19E-276	Loss	Gain
Chr12_CNVR_52	Chr12	73239534	73276351	36818	-2.264837	0.208073	0	Loss	Gain
Chr12_CNVR_53	Chr12	73287128	73302393	15266	-1.822923	0.282648	0	Loss	Gain
Chr12_CNVR_54	Chr12	73329334	73347293	17960	-1.460513	0.363364	0	Loss	Gain
Chr12_CNVR_55	Chr12	73372438	73404765	32328	-1.592067	0.331696	0	Loss	Gain
Chr12_CNVR_56	Chr12	73489178	73504443	15266	-1.250647	0.42026	0	Loss	Gain
Chr12_CNVR_57	Chr12	73518812	73531383	12572	-1.817947	0.283624	0	Loss	Gain
Chr12_CNVR_58	Chr12	73549344	73571793	22450	-2.75	0.148651	0	Loss	Gain
Chr12_CNVR_59	Chr12	73572692	73635551	62860	-2.858039	0.137925	0	Loss	Gain
Chr12_CNVR_60	Chr12	73636450	73672369	35920	-2.353658	0.195649	0	Loss	Gain
Chr12_CNVR_61	Chr12	73675962	73711881	35920	-2.379681	0.192152	0	Loss	Gain
Chr12_CNVR_62	Chr12	73719066	73733433	14368	-1.131869	0.456324	0	Loss	Gain
Chr12_CNVR_63	Chr12	73737026	73746903	9878	-1.777604	0.291667	0	Loss	Gain
Chr12_CNVR_64	Chr12	73799886	73810661	10776	-0.9560052	0.515482	6.81E-245	Loss	Gain
Chr12_CNVR_65	Chr12	73811560	73825029	13470	-1.089087	0.470059	0	Loss	Gain
Chr12_CNVR_66	Chr12	73887890	73912135	24246	-2.866681	0.137102	0	Loss	Gain
Chr12_CNVR_67	Chr12	73930994	73957933	26940	-1.690993	0.309714	0	Loss	Gain
Chr12_CNVR_68	Chr12	74076470	74089939	13470	-1.090753	0.469516	0	Loss	Gain
Chr12_CNVR_69	Chr12	74200394	74250681	50288	-1.409991	0.376314	0	Loss	Gain
Chr12_CNVR_70	Chr12	74253376	74281213	27838	-1.446323	0.366955	0	Loss	Gain
Chr12_CNVR_71	Chr12	74295582	74321623	26042	-3.938987	0.0651999	0	Loss	Gain
Chr12_CNVR_72	Chr12	74364728	74379993	15266	-2.33923	0.197616	0	Loss	Gain
Chr12_CNVR_19	Chr12	74420404	74443751	23348	2.928581	7.61361	0	Gain	Loss
Chr12_CNVR_73	Chr12	74521878	74530857	8980	-0.938699	0.521703	2.41E-198	Loss	Gain
Chr12_CNVR_74	Chr12	74641312	74761643	120332	-1.90078	0.267799	0	Loss	Gain
Chr12_CNVR_75	Chr12	74767930	75136109	368180	-3.462519	0.0907148	0	Loss	Gain
Chr12_CNVR_76	Chr12	75137906	75217827	79922	-2.200989	0.217488	0	Loss	Gain
Chr12_CNVR_77	Chr12	75219624	75235787	16164	-1.548195	0.341938	0	Loss	Gain
Chr12_CNVR_78	Chr12	75445022	75575231	130210	-1.473351	0.360145	0	Loss	Gain
Chr12_CNVR_79	Chr12	75580620	75619233	38614	-1.124196	0.458758	0	Loss	Gain
Chr12_CNVR_20	Chr12	75885042	75903001	17960	2.269261	4.82076	0	Gain	Loss
Chr12_CNVR_80	Chr12	75946106	75957779	11674	-1.17991	0.441379	0	Loss	Gain
Chr12_CNVR_81	Chr12	76046682	76062845	16164	-1.423381	0.372838	0	Loss	Gain
Chr12_CNVR_82	Chr12	76063744	76074519	10776	-1.594141	0.331219	0	Loss	Gain
Chr12_CNVR_83	Chr12	76075418	76121215	45798	-1.573965	0.335884	0	Loss	Gain
Chr12_CNVR_84	Chr12	76170606	76182279	11674	-1.688715	0.310203	0	Loss	Gain

Chr12_CNVR_85	Chr12	76209220	76237955	28736	-1.518957	0.348938	0	Loss	Gain
Chr12_CNVR_86	Chr12	76238854	76273875	35022	-2.177196	0.221105	0	Loss	Gain
Chr12_CNVR_21	Chr12	76370860	76387921	17062	1.040631	2.05713	0	Gain	Loss
Chr12_CNVR_22	Chr12	76410372	76419351	8980	1.527952	2.88376	0	Gain	Loss
Chr12_CNVR_87	Chr12	76459762	76472333	12572	-0.8850035	0.541486	8.75E-251	Loss	Gain
Chr12_CNVR_88	Chr12	76482212	76500171	17960	-0.9456776	0.519186	0	Loss	Gain
Chr12_CNVR_23	Chr12	76501968	76513641	11674	1.11008	2.15858	0	Gain	Loss
Chr12_CNVR_89	Chr12	76530704	76553153	22450	-2.06701	0.238654	0	Loss	Gain
Chr12_CNVR_90	Chr12	76554052	76577399	23348	-0.9656009	0.512065	0	Loss	Gain
Chr12_CNVR_91	Chr12	76620504	76634871	14368	-0.9411902	0.520803	0	Loss	Gain
Chr12_CNVR_92	Chr12	76635770	76651035	15266	-1.382923	0.383441	0	Loss	Gain
Chr12_CNVR_93	Chr12	76652832	76664505	11674	-1.079787	0.473099	0	Loss	Gain
Chr12_CNVR_94	Chr12	76669894	76693241	23348	-1.130095	0.456886	0	Loss	Gain
Chr12_CNVR_95	Chr12	76694140	76709405	15266	-1.027458	0.490574	0	Loss	Gain
Chr12_CNVR_96	Chr12	89003924	89014699	10776	-0.8265605	0.563872	1.33E-191	Loss	Gain
Chr12_CNVR_97	Chr12	89335286	89345163	9878	-0.8410477	0.558238	4.04E-181	Loss	Gain
Chr12_CNVR_98	Chr12	89454720	89463699	8980	-0.8843271	0.54174	1.69E-179	Loss	Gain
Chr12_CNVR_99	Chr12	89660362	89671137	10776	-0.8936179	0.538263	8.29E-219	Loss	Gain
Chr12_CNVR_100	Chr12	89768122	89777101	8980	-0.8672383	0.548195	1.13E-173	Loss	Gain
Chr12_CNVR_101	Chr12	89905516	89920781	15266	-0.8318139	0.561822	1.60E-273	Loss	Gain
Chr12_CNVR_102	Chr12	89930660	89943231	12572	-0.8843645	0.541726	1.77E-250	Loss	Gain
Chr12_CNVR_103	Chr12	90136302	90146179	9878	-0.8737459	0.545728	2.88E-193	Loss	Gain
Chr12_CNVR_104	Chr12	90163242	90176711	13470	-0.8783272	0.543998	4.03E-265	Loss	Gain
Chr12_CNVR_105	Chr12	90229694	90241367	11674	-0.7869724	0.579559	1.62E-190	Loss	Gain
Chr12_CNVR_106	Chr12	90245858	90254837	8980	-0.835816	0.560266	4.31E-163	Loss	Gain
Chr12_CNVR_107	Chr12	90266512	90275491	8980	-0.8724395	0.546222	1.93E-175	Loss	Gain
Chr12_CNVR_108	Chr12	90361700	90391333	29634	-0.8702308	0.547059	0	Loss	Gain
Chr12_CNVR_109	Chr12	90392232	90404803	12572	-0.9953748	0.501606	6.40E-305	Loss	Gain
Chr12_CNVR_110	Chr12	90421866	90438029	16164	-0.891683	0.538985	0	Loss	Gain
Chr12_CNVR_111	Chr12	90539504	90551177	11674	-0.8525769	0.553795	1.05E-218	Loss	Gain
Chr12_CNVR_112	Chr12	90584404	90594281	9878	-0.8729098	0.546044	5.92E-193	Loss	Gain
Chr12_CNVR_113	Chr12	90634692	90643671	8980	-0.8442835	0.556987	6.32E-166	Loss	Gain
Chr12_CNVR_114	Chr12	90654448	90663427	8980	-0.9095214	0.532362	3.55E-188	Loss	Gain
Chr12_CNVR_115	Chr12	90746044	90756819	10776	-0.8944794	0.537941	3.66E-219	Loss	Gain
Chr12_CNVR_116	Chr12	90764902	90773881	8980	-0.9009735	0.535525	3.20E-185	Loss	Gain
Chr12_CNVR_117	Chr12	90792740	90803515	10776	-0.8945683	0.537908	3.36E-219	Loss	Gain
Chr12_CNVR_118	Chr12	90807108	90816087	8980	-0.8869432	0.540759	2.14E-180	Loss	Gain
Chr12_CNVR_119	Chr12	90924746	90940909	16164	-0.8542394	0.553157	5.25E-303	Loss	Gain
Chr12_CNVR_120	Chr12	90948094	90957073	8980	-0.8251149	0.564437	1.57E-159	Loss	Gain
Chr13_CNVR_1	Chr13	239529	263261	23733	2.390695	5.2441	0	Gain	Loss
Chr13_CNVR_2	Chr13	10995851	11004641	8791	1.787924	3.45318	0	Gain	Loss
Chr13_CNVR_3	Chr13	11009915	11030131	20217	1.101058	2.14512	0	Gain	Loss
Chr13_CNVR_4	Chr13	11037165	11047711	10547	1.096925	2.13898	0	Gain	Loss
Chr13_CNVR_5	Chr13	11066171	11081113	14943	1.328009	2.51056	0	Gain	Loss
Chr13_CNVR_6	Chr13	11090783	11103089	12307	1.204634	2.30479	0	Gain	Loss
Chr13_CNVR_7	Chr13	11113637	11138249	24613	1.148562	2.21693	0	Gain	Loss
Chr13_CNVR_8	Chr13	11176047	11184835	8789	0.7997144	1.74076	2.85E-169	Gain	Loss
Chr13_CNVR_9	Chr13	11340419	11365031	24613	1.149645	2.21859	0	Gain	Loss
Chr13_CNVR_10	Chr13	11367669	11384369	16701	0.9337732	1.91027	0	Gain	Loss
Chr13_CNVR_11	Chr13	11437989	11459963	21975	1.056078	2.07927	0	Gain	Loss
Chr13_CNVR_12	Chr13	11483697	11502155	18459	0.8761038	1.83541	0	Gain	Loss
Chr13_CNVR_13	Chr13	17837987	17863477	25491	1.056678	2.08014	0	Gain	Loss
Chr13_CNVR_14	Chr13	23349317	23360743	11427	1.043999	2.06194	0	Gain	Loss
Chr13_CNVR_16	Chr13	53932365	53984225	51861	-5.117468	0.0288064	0	Loss	Gain
Chr13_CNVR_17	Chr13	63029135	63066053	36919	-1.193735	0.43717	0	Loss	Gain
Chr13_CNVR_15	Chr13	70139367	70149913	10547	4.261599	19.1809	0	Gain	Loss

Chr14_CNVR_10	Chr14	1638086	1646105	8020	-0.8352489	0.560486	1.22E-162	Loss	Gain
Chr14_CNVR_11	Chr14	2137732	2147355	9624	-1.259619	0.417654	0	Loss	Gain
Chr14_CNVR_12	Chr14	2161792	2171415	9624	-0.8569804	0.552107	1.95E-203	Loss	Gain
Chr14_CNVR_13	Chr14	2505048	2513067	8020	-0.8358573	0.56025	7.65E-163	Loss	Gain
Chr14_CNVR_14	Chr14	2913266	2926097	12832	-0.9032635	0.534676	1.34E-295	Loss	Gain
Chr14_CNVR_15	Chr14	3559678	3567697	8020	-0.8090459	0.570759	4.93E-154	Loss	Gain
Chr14_CNVR_16	Chr14	3653512	3662333	8822	-0.90665	0.533422	3.65E-205	Loss	Gain
Chr14_CNVR_17	Chr14	3699226	3708047	8822	-0.85034	0.554654	3.24E-184	Loss	Gain
Chr14_CNVR_18	Chr14	11400030	11412059	12030	-0.9551136	0.515801	3.94E-304	Loss	Gain
Chr14_CNVR_19	Chr14	11417674	11434515	16842	-1.059657	0.479746	0	Loss	Gain
Chr14_CNVR_20	Chr14	14498156	14508581	10426	-0.9452049	0.519356	2.21E-259	Loss	Gain
Chr14_CNVR_21	Chr14	14541464	14561513	20050	-0.8782965	0.544009	0	Loss	Gain
Chr14_CNVR_22	Chr14	14566326	14576751	10426	-0.9872489	0.504439	1.60E-278	Loss	Gain
Chr14_CNVR_23	Chr14	14583970	14599207	15238	-0.9999236	0.500026	0	Loss	Gain
Chr14_CNVR_24	Chr14	14608030	14619257	11228	-1.795914	0.287989	0	Loss	Gain
Chr14_CNVR_25	Chr14	14620060	14641713	21654	-1.307519	0.404015	0	Loss	Gain
Chr14_CNVR_26	Chr14	14644120	14695447	51328	-1.106695	0.464357	0	Loss	Gain
Chr14_CNVR_27	Chr14	14696250	14718705	22456	-1.151278	0.450226	0	Loss	Gain
Chr14_CNVR_28	Chr14	14719508	14749181	29674	-1.063848	0.478354	0	Loss	Gain
Chr14_CNVR_29	Chr14	14749984	14764419	14436	-1.060174	0.479574	0	Loss	Gain
Chr14_CNVR_30	Chr14	14766024	14786875	20852	-1.054465	0.481476	0	Loss	Gain
Chr14_CNVR_31	Chr14	14787678	14801311	13634	-1.098439	0.467022	0	Loss	Gain
Chr14_CNVR_32	Chr14	14803718	14816549	12832	-1.075748	0.474425	0	Loss	Gain
Chr14_CNVR_33	Chr14	14817352	14845421	28070	-1.026061	0.491049	0	Loss	Gain
Chr14_CNVR_34	Chr14	14846224	14861461	15238	-1.105656	0.464691	0	Loss	Gain
Chr14_CNVR_35	Chr14	14867878	14887927	20050	-1.06911	0.476613	0	Loss	Gain
Chr14_CNVR_36	Chr14	14891136	14936849	45714	-1.238097	0.423931	0	Loss	Gain
Chr14_CNVR_37	Chr14	15015446	15033089	17644	-1.30111	0.405814	0	Loss	Gain
Chr14_CNVR_38	Chr14	15036298	15049129	12832	-1.055574	0.481106	0	Loss	Gain
Chr14_CNVR_39	Chr14	15057952	15187875	129924	-1.035325	0.487906	0	Loss	Gain
Chr14_CNVR_40	Chr14	15189480	15198301	8822	-0.9713166	0.51004	7.74E-230	Loss	Gain
Chr14_CNVR_41	Chr14	15234392	15242411	8020	-1.005339	0.498153	3.91E-221	Loss	Gain
Chr14_CNVR_42	Chr14	15278502	15287323	8822	-1.115998	0.461372	1.05E-286	Loss	Gain
Chr14_CNVR_43	Chr14	15288928	15316997	28070	-1.04273	0.485408	0	Loss	Gain
Chr14_CNVR_44	Chr14	15318602	15391583	72982	-1.084929	0.471415	0	Loss	Gain
Chr14_CNVR_45	Chr14	15395594	15407623	12030	-1.256188	0.418649	0	Loss	Gain
Chr14_CNVR_1	Chr14	30723418	30732239	8822	1.060396	2.0855	3.21E-292	Gain	Loss
Chr14_CNVR_2	Chr14	32270476	32292931	22456	2.329922	5.02778	0	Gain	Loss
Chr14_CNVR_46	Chr14	32534334	32543957	9624	-0.889442	0.539823	1.41E-216	Loss	Gain
Chr14_CNVR_3	Chr14	44857866	44867489	9624	2.016272	4.04537	0	Gain	Loss
Chr14_CNVR_4	Chr14	53283678	53291697	8020	0.8921434	1.85593	3.18E-198	Gain	Loss
Chr14_CNVR_5	Chr14	53549942	53558763	8822	1.157362	2.23049	0	Gain	Loss
Chr14_CNVR_6	Chr14	54400062	54408081	8020	0.8179983	1.76296	3.57E-170	Gain	Loss
Chr14_CNVR_7	Chr14	62711188	62720009	8822	0.9366753	1.91411	5.29E-237	Gain	Loss
Chr14_CNVR_8	Chr14	62810636	62819457	8822	1.167247	2.24583	0	Gain	Loss
Chr14_CNVR_9	Chr14	67336322	67344341	8020	0.9538126	1.93698	1.86E-222	Gain	Loss
Chr14_CNVR_47	Chr14	68126292	68135113	8822	-0.8651637	0.548984	1.12E-189	Loss	Gain
Chr15_CNVR_1	Chr15	11375333	11391297	15965	1.17994	2.26567	0	Gain	Loss
Chr15_CNVR_25	Chr15	20051967	20060835	8869	-3.213262	0.107823	0	Loss	Gain
Chr15_CNVR_26	Chr15	25560237	25577089	16853	-2.740377	0.149646	0	Loss	Gain
Chr15_CNVR_27	Chr15	30755395	30765151	9757	-0.7839502	0.580774	2.57E-160	Loss	Gain
Chr15_CNVR_28	Chr15	45721747	45731503	9757	-2.060392	0.239751	0	Loss	Gain
Chr15_CNVR_29	Chr15	46607859	46619389	11531	-1.052392	0.482168	0	Loss	Gain
Chr15_CNVR_2	Chr15	46827835	46838479	10645	1.09116	2.13045	0	Gain	Loss
Chr15_CNVR_3	Chr15	46894361	46908551	14191	1.096268	2.13801	0	Gain	Loss
Chr15_CNVR_4	Chr15	48453707	48465237	11531	1.099659	2.14304	0	Gain	Loss

Chr15_CNVR_5	Chr15	49090573	49103877	13305	1.082488	2.11768	0	Gain	Loss
Chr15_CNVR_6	Chr15	49111861	49124277	12417	0.9603916	1.94584	0	Gain	Loss
Chr15_CNVR_30	Chr15	50719105	50754583	35479	-4.214915	0.0538498	0	Loss	Gain
Chr15_CNVR_31	Chr15	50788291	50807803	19513	-2.047198	0.241954	0	Loss	Gain
Chr15_CNVR_7	Chr15	50811353	50830865	19513	1.066226	2.09395	0	Gain	Loss
Chr15_CNVR_32	Chr15	50831753	50844171	12419	-2.689361	0.155032	0	Loss	Gain
Chr15_CNVR_33	Chr15	52215473	52227003	11531	-1.348755	0.392631	0	Loss	Gain
Chr15_CNVR_34	Chr15	52229665	52248291	18627	-1.125354	0.45839	0	Loss	Gain
Chr15_CNVR_35	Chr15	52264259	52282885	18627	-1.881592	0.271384	0	Loss	Gain
Chr15_CNVR_36	Chr15	53044819	53053687	8869	-0.8223231	0.565531	2.00E-158	Loss	Gain
Chr15_CNVR_37	Chr15	57214605	57225249	10645	-2.117252	0.230486	0	Loss	Gain
Chr15_CNVR_8	Chr15	64543887	64566061	22175	1.302878	2.46721	0	Gain	Loss
Chr15_CNVR_38	Chr15	77189845	77199601	9757	-0.8326443	0.561499	8.17E-178	Loss	Gain
Chr15_CNVR_39	Chr15	78331415	78343831	12417	-2.86361	0.137394	0	Loss	Gain
Chr15_CNVR_9	Chr15	79531525	79543055	11531	3.111605	8.64344	0	Gain	Loss
Chr15_CNVR_10	Chr15	79606033	79617563	11531	1.220987	2.33106	0	Gain	Loss
Chr15_CNVR_11	Chr15	79816253	79833105	16853	1.209821	2.31309	0	Gain	Loss
Chr15_CNVR_12	Chr15	80490373	80508111	17739	0.8671335	1.82404	0	Gain	Loss
Chr15_CNVR_13	Chr15	80508999	80548913	39915	0.9321423	1.90811	0	Gain	Loss
Chr15_CNVR_14	Chr15	80569315	80588829	19515	0.9083332	1.87688	0	Gain	Loss
Chr15_CNVR_15	Chr15	80592377	80617213	24837	0.9313404	1.90705	0	Gain	Loss
Chr15_CNVR_16	Chr15	80656241	80675755	19515	0.8900025	1.85318	0	Gain	Loss
Chr15_CNVR_17	Chr15	80679303	80702365	23063	0.9106429	1.87988	0	Gain	Loss
Chr15_CNVR_18	Chr15	80709461	80736957	27497	0.8534458	1.80681	0	Gain	Loss
Chr15_CNVR_19	Chr15	80752037	80761793	9757	0.8437802	1.79475	3.51E-195	Gain	Loss
Chr15_CNVR_20	Chr15	80767117	80775985	8869	0.8167966	1.76149	9.98E-168	Gain	Loss
Chr15_CNVR_21	Chr15	80807031	80842511	35481	1.371266	2.58697	0	Gain	Loss
Chr15_CNVR_22	Chr15	81004833	81028781	23949	1.224192	2.33625	0	Gain	Loss
Chr15_CNVR_23	Chr15	81029669	81086435	56767	1.117565	2.1698	0	Gain	Loss
Chr15_CNVR_24	Chr15	81300203	81309073	8871	1.144421	2.21057	6.76E-297	Gain	Loss
Chr15_CNVR_40	Chr15	81743703	81752573	8871	-0.8407358	0.558359	1.59E-164	Loss	Gain
Chr15_CNVR_41	Chr15	81812003	81821759	9757	-0.9167624	0.529696	3.78E-209	Loss	Gain
Chr15_CNVR_42	Chr15	81822647	81840385	17739	-0.8495151	0.554971	0	Loss	Gain
Chr15_CNVR_43	Chr15	81869657	81929973	60317	-2.375839	0.192664	0	Loss	Gain
Chr15_CNVR_44	Chr15	82086973	82096729	9757	-0.8649248	0.549075	1.10E-189	Loss	Gain
Chr15_CNVR_45	Chr15	83368687	83378443	9757	-2.492004	0.177759	0	Loss	Gain
Chr16_CNVR_9	Chr16	93615	103283	9669	-0.7717087	0.585723	5.87E-156	Loss	Gain
Chr16_CNVR_1	Chr16	2107403	2122345	14943	1.001558	2.00216	0	Gain	Loss
Chr16_CNVR_2	Chr16	6028623	6037411	8789	0.8448272	1.79605	1.21E-180	Gain	Loss
Chr16_CNVR_3	Chr16	6084879	6093667	8789	0.9654443	1.95266	9.67E-228	Gain	Loss
Chr16_CNVR_4	Chr16	6100701	6110369	9669	0.9082018	1.8767	2.63E-225	Gain	Loss
Chr16_CNVR_5	Chr16	7408653	7419199	10547	0.9412061	1.92013	3.16E-261	Gain	Loss
Chr16_CNVR_6	Chr16	11524131	11533799	9669	3.207578	9.23798	0	Gain	Loss
Chr16_CNVR_7	Chr16	24811095	24820763	9669	0.8651249	1.8215	5.62E-207	Gain	Loss
Chr16_CNVR_8	Chr16	39449081	39477209	28129	2.853403	7.22703	0	Gain	Loss
Chr16_CNVR_10	Chr16	49636691	49645481	8791	-0.8893582	0.539854	1.01E-180	Loss	Gain
Chr16_CNVR_11	Chr16	49659545	49676245	16701	-0.8799694	0.543379	0	Loss	Gain
Chr16_CNVR_12	Chr16	50673911	50685337	11427	-0.8493351	0.55504	8.23E-217	Loss	Gain
Chr16_CNVR_13	Chr16	50694129	50707313	13185	-0.8772992	0.544386	6.62E-264	Loss	Gain
Chr16_CNVR_14	Chr16	51117807	51126595	8789	-0.8280997	0.563271	3.19E-160	Loss	Gain
Chr16_CNVR_15	Chr16	51147693	51156481	8789	-0.9202573	0.528415	2.75E-191	Loss	Gain
Chr16_CNVR_16	Chr16	51614441	51624989	10549	-0.8362895	0.560082	4.24E-195	Loss	Gain
Chr16_CNVR_17	Chr16	51668939	51680365	11427	-0.8893424	0.53986	2.65E-234	Loss	Gain
Chr16_CNVR_18	Chr16	70880363	70889153	8791	-0.8490524	0.555149	3.66E-167	Loss	Gain
Chr16_CNVR_19	Chr16	70958595	70977053	18459	-0.8310411	0.562123	0	Loss	Gain
Chr16_CNVR_20	Chr16	77057975	77067643	9669	-0.8173116	0.567498	3.81E-172	Loss	Gain

Chr16_CNVR_21	Chr16	81673605	81683273	9669	-0.8703478	0.547015	1.62E-191	Loss	Gain
Chr17_CNVR_1	Chr17	2165911	2175370	9460	0.8848413	1.84656	5.70E-214	Gain	Loss
Chr17_CNVR_2	Chr17	2231271	2264810	33540	0.9125037	1.88231	0	Gain	Loss
Chr17_CNVR_10	Chr17	14334911	14343510	8600	-0.8712072	0.546689	1.23E-174	Loss	Gain
Chr17_CNVR_3	Chr17	15323051	15332510	9460	2.673055	6.37778	0	Gain	Loss
Chr17_CNVR_4	Chr17	21082471	21097090	14620	4.011781	16.1312	0	Gain	Loss
Chr17_CNVR_11	Chr17	25029011	25045350	16340	-1.07575	0.474424	0	Loss	Gain
Chr17_CNVR_5	Chr17	31413651	31424830	11180	0.89044	1.85374	2.77E-255	Gain	Loss
Chr17_CNVR_12	Chr17	45696531	45705130	8600	-0.8358296	0.560261	8.26E-163	Loss	Gain
Chr17_CNVR_13	Chr17	45731791	45740390	8600	-0.7606846	0.590216	1.80E-138	Loss	Gain
Chr17_CNVR_14	Chr17	51377691	51396610	18920	-0.9496724	0.51775	0	Loss	Gain
Chr17_CNVR_6	Chr17	52439791	52450110	10320	6.850475	115.398	0	Gain	Loss
Chr17_CNVR_7	Chr17	52469031	52481070	12040	2.634575	6.20992	0	Gain	Loss
Chr17_CNVR_15	Chr17	53594771	53605950	11180	-0.8322395	0.561657	1.73E-209	Loss	Gain
Chr17_CNVR_16	Chr17	53695391	53705710	10320	-0.8494442	0.554999	2.17E-200	Loss	Gain
Chr17_CNVR_17	Chr17	62944691	62957590	12900	-0.8262365	0.563999	2.02E-238	Loss	Gain
Chr17_CNVR_18	Chr17	65865251	65873850	8600	-0.8779412	0.544143	6.50E-177	Loss	Gain
Chr17_CNVR_19	Chr17	69289771	69298370	8600	-0.7869045	0.579586	7.84E-147	Loss	Gain
Chr17_CNVR_8	Chr17	70099031	70120530	21500	1.745755	3.3537	0	Gain	Loss
Chr17_CNVR_20	Chr17	71476751	71485350	8600	-0.8527462	0.55373	1.94E-168	Loss	Gain
Chr17_CNVR_21	Chr17	71586831	71596290	9460	-0.8705558	0.546936	1.18E-191	Loss	Gain
Chr17_CNVR_9	Chr17	72846731	72863070	16340	1.446532	2.72552	0	Gain	Loss
Chr17_CNVR_22	Chr17	73148591	73157190	8600	-1.387247	0.382294	0	Loss	Gain
Chr17_CNVR_23	Chr17	73183851	73195030	11180	-0.870606	0.546917	3.86E-226	Loss	Gain
Chr17_CNVR_24	Chr17	73249211	73257810	8600	-0.9023558	0.535012	3.19E-185	Loss	Gain
Chr17_CNVR_25	Chr17	73415191	73423790	8600	-0.9121558	0.53139	1.39E-188	Loss	Gain
Chr17_CNVR_26	Chr17	73471091	73479690	8600	-0.9223385	0.527653	4.34E-192	Loss	Gain
Chr17_CNVR_27	Chr17	73515811	73524410	8600	-0.86435	0.549294	2.50E-172	Loss	Gain
Chr17_CNVR_28	Chr17	73681791	73690390	8600	-0.9294537	0.525057	1.51E-194	Loss	Gain
Chr17_CNVR_29	Chr17	73702431	73718770	16340	-0.8848006	0.541562	0	Loss	Gain
Chr17_CNVR_30	Chr17	73760051	73769510	9460	-1.085709	0.471161	1.03E-274	Loss	Gain
Chr17_CNVR_31	Chr17	73791011	73815090	24080	-0.9638337	0.512693	0	Loss	Gain
Chr17_CNVR_32	Chr17	73972471	73981930	9460	-0.8877686	0.540449	4.60E-198	Loss	Gain
Chr17_CNVR_33	Chr17	74087711	74097170	9460	-0.9166724	0.529729	6.09E-209	Loss	Gain
Chr17_CNVR_34	Chr17	74139311	74147910	8600	-0.9078661	0.532973	4.13E-187	Loss	Gain
Chr17_CNVR_35	Chr17	74158231	74170270	12040	-0.949976	0.517641	1.70E-281	Loss	Gain
Chr17_CNVR_36	Chr17	74189191	74200370	11180	-0.9167648	0.529696	1.38E-246	Loss	Gain
Chr17_CNVR_37	Chr17	74244231	74252830	8600	-0.8425686	0.55765	4.80E-165	Loss	Gain
Chr17_CNVR_38	Chr17	74290671	74306150	15480	-0.9407226	0.520972	0	Loss	Gain
Chr17_CNVR_39	Chr17	74342271	74353450	11180	-0.9523882	0.516776	1.23E-262	Loss	Gain
Chr17_CNVR_40	Chr17	74453211	74461810	8600	-0.9499516	0.51765	1.15E-201	Loss	Gain
Chr17_CNVR_41	Chr17	74464391	74475570	11180	-0.9611002	0.513665	1.36E-266	Loss	Gain
Chr17_CNVR_42	Chr17	74487611	74501370	13760	-0.828529	0.563103	2.21E-255	Loss	Gain
Chr17_CNVR_43	Chr17	74534911	74548670	13760	-0.9582142	0.514694	0	Loss	Gain
Chr17_CNVR_44	Chr17	74639831	74648430	8600	-0.9387343	0.52169	9.16E-198	Loss	Gain
Chr17_CNVR_45	Chr17	74673371	74684550	11180	-0.8653011	0.548932	8.10E-224	Loss	Gain
Chr17_CNVR_46	Chr17	74710351	74720670	10320	-0.8847703	0.541574	1.17E-214	Loss	Gain
Chr17_CNVR_47	Chr17	74756791	74780010	23220	-0.9242739	0.526946	0	Loss	Gain
Chr17_CNVR_48	Chr17	74788611	74803230	14620	-0.9534065	0.516412	0	Loss	Gain
Chr17_CNVR_49	Chr17	74839351	74851390	12040	-0.8429795	0.557491	2.01E-230	Loss	Gain
Chr17_CNVR_50	Chr17	74914171	74930510	16340	-0.9321421	0.52408	0	Loss	Gain
Chr17_CNVR_51	Chr17	74976091	74988990	12900	-1.182641	0.440544	0	Loss	Gain
Chr17_CNVR_52	Chr17	74990711	75002750	12040	-0.9083504	0.532794	2.41E-261	Loss	Gain
Chr17_CNVR_53	Chr17	75070691	75082730	12040	-0.9064922	0.533481	1.88E-260	Loss	Gain
Chr19_CNVR_1	Chr19	1206037	1216332	10296	1.724517	3.30469	0	Gain	Loss
Chr19_CNVR_2	Chr19	1866853	1877148	10296	1.291848	2.44841	0	Gain	Loss

Chr19_CNVR_3	Chr19	2885221	2896452	11232	0.9048387	1.87234	3.40E-261	Gain	Loss
Chr19_CNVR_4	Chr19	3422485	3439332	16848	1.546705	2.92149	0	Gain	Loss
Chr19_CNVR_5	Chr19	19073341	19085508	12168	1.092436	2.13234	0	Gain	Loss
Chr19_CNVR_6	Chr19	19122949	19132308	9360	0.859324	1.81419	7.13E-199	Gain	Loss
Chr19_CNVR_7	Chr19	19795933	19826820	30888	1.348395	2.54629	0	Gain	Loss
Chr19_CNVR_8	Chr19	19827757	19851156	23400	1.769425	3.40918	0	Gain	Loss
Chr19_CNVR_9	Chr19	19866133	19897956	31824	1.952064	3.86928	0	Gain	Loss
Chr19_CNVR_10	Chr19	19902637	19934460	31824	1.196291	2.2915	0	Gain	Loss
Chr19_CNVR_11	Chr19	19935397	19952244	16848	1.479361	2.78825	0	Gain	Loss
Chr19_CNVR_12	Chr19	38842597	38854764	12168	0.8947963	1.85935	3.12E-277	Gain	Loss
Chr19_CNVR_15	Chr19	43915717	43927884	12168	-1.306466	0.40431	0	Loss	Gain
Chr19_CNVR_13	Chr19	47246005	47255364	9360	0.9050257	1.87258	4.26E-218	Gain	Loss
Chr19_CNVR_16	Chr19	48867157	48877452	10296	-1.089147	0.470039	9.69E-273	Loss	Gain
Chr19_CNVR_14	Chr19	52132861	52145964	13104	1.791579	3.46194	0	Gain	Loss
Chr19_CNVR_17	Chr19	64048141	64058436	10296	-1.42695	0.371916	0	Loss	Gain
Chr2_CNVR_7	Chr2	55923	65459	9537	-0.8933301	0.53837	9.02E-201	Loss	Gain
Chr2_CNVR_8	Chr2	85401	115745	30345	-1.071068	0.475967	0	Loss	Gain
Chr2_CNVR_9	Chr2	128751	142621	13871	-1.262464	0.416831	0	Loss	Gain
Chr2_CNVR_10	Chr2	54595425	54610163	14739	-0.875724	0.54498	7.50E-299	Loss	Gain
Chr2_CNVR_11	Chr2	54612765	54629237	16473	-1.184065	0.44011	0	Loss	Gain
Chr2_CNVR_1	Chr2	56706569	56716973	10405	0.9366652	1.9141	1.11E-248	Gain	Loss
Chr2_CNVR_2	Chr2	57407973	57416641	8669	1.260106	2.39513	0	Gain	Loss
Chr2_CNVR_3	Chr2	68405001	68417137	12137	3.246733	9.49214	0	Gain	Loss
Chr2_CNVR_4	Chr2	68451819	68460487	8669	2.60889	6.10034	0	Gain	Loss
Chr2_CNVR_12	Chr2	79761833	79771369	9537	-2.2105	0.216059	0	Loss	Gain
Chr2_CNVR_13	Chr2	104934311	104952517	18207	-0.837435	0.559638	0	Loss	Gain
Chr2_CNVR_14	Chr2	106679583	106689119	9537	-0.8259656	0.564105	1.15E-175	Loss	Gain
Chr2_CNVR_15	Chr2	106706459	106715995	9537	-0.8381107	0.559376	4.01E-180	Loss	Gain
Chr2_CNVR_16	Chr2	106735937	106756745	20809	-0.9110572	0.531795	0	Loss	Gain
Chr2_CNVR_17	Chr2	108097127	108117067	19941	-0.8537731	0.553336	0	Loss	Gain
Chr2_CNVR_18	Chr2	108123137	108135275	12139	-0.919289	0.52877	1.51E-267	Loss	Gain
Chr2_CNVR_19	Chr2	108148281	108157817	9537	-0.8630722	0.549781	2.20E-189	Loss	Gain
Chr2_CNVR_20	Chr2	120178773	120196111	17339	-0.8556552	0.552614	0	Loss	Gain
Chr2_CNVR_21	Chr2	120900983	120910519	9537	-0.8208416	0.566112	8.60E-174	Loss	Gain
Chr2_CNVR_22	Chr2	120974679	120988549	13871	-0.9013505	0.535385	1.71E-295	Loss	Gain
Chr2_CNVR_23	Chr2	122527475	122543947	16473	-0.9077838	0.533003	0	Loss	Gain
Chr2_CNVR_24	Chr2	122544815	122555219	10405	-0.8190931	0.566798	1.04E-188	Loss	Gain
Chr2_CNVR_25	Chr2	122565623	122575159	9537	-0.8109083	0.570023	3.52E-170	Loss	Gain
Chr2_CNVR_26	Chr2	122609841	122618509	8669	-0.7883701	0.578998	1.61E-147	Loss	Gain
Chr2_CNVR_5	Chr2	124338639	124347307	8669	0.8277556	1.77492	1.18E-167	Gain	Loss
Chr2_CNVR_27	Chr2	126327537	126336205	8669	-0.7710511	0.58599	6.32E-142	Loss	Gain
Chr2_CNVR_28	Chr2	126535617	126544285	8669	-0.7544253	0.592782	1.28E-136	Loss	Gain
Chr2_CNVR_29	Chr2	128566131	128576533	10403	-0.79696	0.575561	5.68E-180	Loss	Gain
Chr2_CNVR_30	Chr2	129888305	129896975	8671	-0.8662388	0.548575	1.97E-173	Loss	Gain
Chr2_CNVR_31	Chr2	130518615	130527283	8669	-0.8551744	0.552798	1.12E-169	Loss	Gain
Chr2_CNVR_32	Chr2	130542023	130553293	11271	-0.8833723	0.542099	1.93E-232	Loss	Gain
Chr2_CNVR_33	Chr2	130568033	130578437	10405	-0.8112693	0.56988	1.32E-185	Loss	Gain
Chr2_CNVR_34	Chr2	130606181	130617451	11271	-0.9174209	0.529455	9.63E-248	Loss	Gain
Chr2_CNVR_35	Chr2	130636527	130652999	16473	-0.8917588	0.538957	0	Loss	Gain
Chr2_CNVR_36	Chr2	130653867	130665137	11271	-0.7880139	0.579141	5.21E-191	Loss	Gain
Chr2_CNVR_37	Chr2	130694615	130703285	8671	-0.8356035	0.560349	4.27E-163	Loss	Gain
Chr2_CNVR_38	Chr2	130770045	130779581	9537	-0.7745495	0.584571	3.75E-157	Loss	Gain
Chr2_CNVR_39	Chr2	130788251	130797787	9537	-0.8462058	0.556246	4.11E-183	Loss	Gain
Chr2_CNVR_40	Chr2	130851543	130863679	12137	-0.892429	0.538706	1.62E-254	Loss	Gain
Chr2_CNVR_41	Chr2	130864547	130874083	9537	-0.916761	0.529697	1.06E-209	Loss	Gain
Chr2_CNVR_42	Chr2	131490521	131500925	10405	-0.8392866	0.55892	8.79E-197	Loss	Gain

Chr2_CNVR_43	Chr2	131503527	131514797	11271	-0.8805384	0.543165	3.53E-231	Loss	Gain
Chr2_CNVR_44	Chr2	131736749	131753221	16473	-0.9579575	0.514785	0	Loss	Gain
Chr2_CNVR_45	Chr2	131779233	131791369	12137	-0.9158116	0.530046	7.52E-266	Loss	Gain
Chr2_CNVR_46	Chr2	131948297	131958701	10405	-0.8332576	0.56126	2.32E-194	Loss	Gain
Chr2_CNVR_47	Chr2	133284345	133296481	12137	-1.240442	0.423243	0	Loss	Gain
Chr2_CNVR_48	Chr2	133536641	133545311	8671	-0.7969337	0.575571	2.62E-150	Loss	Gain
Chr2_CNVR_49	Chr2	133580859	133590395	9537	-0.8431353	0.557431	5.61E-182	Loss	Gain
Chr2_CNVR_50	Chr2	133801077	133809745	8669	-0.8696501	0.54728	1.36E-174	Loss	Gain
Chr2_CNVR_51	Chr2	133849629	133868701	19073	-0.8756046	0.545025	0	Loss	Gain
Chr2_CNVR_52	Chr2	133873037	133886041	13005	-0.9680881	0.511183	0	Loss	Gain
Chr2_CNVR_53	Chr2	133903383	133918987	15605	-0.8678098	0.547978	0	Loss	Gain
Chr2_CNVR_54	Chr2	133941531	133950199	8669	-0.8886528	0.540118	4.25E-181	Loss	Gain
Chr2_CNVR_55	Chr2	133956269	133971875	15607	-0.7705231	0.586205	1.67E-253	Loss	Gain
Chr2_CNVR_56	Chr2	134189493	134200763	11271	-0.9124414	0.531285	1.72E-245	Loss	Gain
Chr2_CNVR_57	Chr2	134273591	134282261	8671	-0.8504962	0.554594	4.24E-168	Loss	Gain
Chr2_CNVR_58	Chr2	134301335	134311739	10405	-0.9111351	0.531767	3.17E-226	Loss	Gain
Chr2_CNVR_59	Chr2	134336015	134345551	9537	-0.829056	0.562897	8.53E-177	Loss	Gain
Chr2_CNVR_60	Chr2	134433987	134445257	11271	-0.8696078	0.547296	2.52E-226	Loss	Gain
Chr2_CNVR_61	Chr2	134480805	134490341	9537	-0.7941145	0.576697	4.00E-164	Loss	Gain
Chr2_CNVR_62	Chr2	134506815	134517217	10403	-0.8450281	0.5567	4.28E-199	Loss	Gain
Chr2_CNVR_63	Chr2	134538893	134547563	8671	-0.8894114	0.539834	2.33E-181	Loss	Gain
Chr2_CNVR_64	Chr2	134548431	134558833	10403	-0.9339199	0.523434	9.06E-236	Loss	Gain
Chr2_CNVR_65	Chr2	134567505	134595247	27743	-0.9246788	0.526798	0	Loss	Gain
Chr2_CNVR_66	Chr2	134613455	134622125	8671	-1.027262	0.49064	4.16E-230	Loss	Gain
Chr2_CNVR_67	Chr2	134635131	134645533	10403	-0.8479591	0.55557	2.80E-200	Loss	Gain
Chr2_CNVR_68	Chr2	134667209	134682815	15607	-0.854452	0.553075	2.70E-303	Loss	Gain
Chr2_CNVR_69	Chr2	134823269	134832805	9537	-0.816918	0.567653	2.31E-172	Loss	Gain
Chr2_CNVR_70	Chr2	134876157	134893495	17339	-0.8392846	0.558921	0	Loss	Gain
Chr2_CNVR_71	Chr2	134903901	134919505	15605	-0.880537	0.543165	0	Loss	Gain
Chr2_CNVR_72	Chr2	134956787	134965457	8671	-0.8568934	0.55214	2.93E-170	Loss	Gain
Chr2_CNVR_73	Chr2	134984531	134994935	10405	-0.8358133	0.560267	2.19E-195	Loss	Gain
Chr2_CNVR_74	Chr2	135002739	135013141	10403	-0.8648167	0.549116	4.02E-207	Loss	Gain
Chr2_CNVR_75	Chr2	135036551	135046087	9537	-0.8079266	0.571202	4.23E-169	Loss	Gain
Chr2_CNVR_76	Chr2	135084237	135092905	8669	-0.8874884	0.540554	1.07E-180	Loss	Gain
Chr2_CNVR_77	Chr2	135098109	135107645	9537	-0.9121965	0.531375	5.92E-208	Loss	Gain
Chr2_CNVR_78	Chr2	135127587	135137123	9537	-0.8698023	0.547222	6.68E-192	Loss	Gain
Chr2_CNVR_79	Chr2	135177005	135190009	13005	-0.8878754	0.540409	3.42E-270	Loss	Gain
Chr2_CNVR_80	Chr2	135262839	135274109	11271	-0.8716893	0.546507	3.01E-227	Loss	Gain
Chr2_CNVR_81	Chr2	135331331	135340001	8671	-0.9170852	0.529578	6.11E-191	Loss	Gain
Chr2_CNVR_82	Chr2	135389421	135404159	14739	-0.8245312	0.564666	1.60E-269	Loss	Gain
Chr2_CNVR_83	Chr2	135507333	135525539	18207	-0.8716393	0.546525	0	Loss	Gain
Chr2_CNVR_84	Chr2	135532475	135548081	15607	-0.9047061	0.534142	0	Loss	Gain
Chr2_CNVR_85	Chr2	135567155	135577559	10405	-0.9621407	0.513295	1.04E-247	Loss	Gain
Chr2_CNVR_86	Chr2	135578427	135596633	18207	-0.9496272	0.517766	0	Loss	Gain
Chr2_CNVR_87	Chr2	135598367	135624377	26011	-0.9860859	0.504846	0	Loss	Gain
Chr2_CNVR_88	Chr2	135634781	135643451	8671	-0.8926503	0.538624	1.79E-182	Loss	Gain
Chr2_CNVR_89	Chr2	135654723	135665993	11271	-0.9952762	0.50164	1.58E-283	Loss	Gain
Chr2_CNVR_90	Chr2	135681599	135690269	8671	-0.9520011	0.516915	3.44E-203	Loss	Gain
Chr2_CNVR_91	Chr2	135692003	135706741	14739	-0.9079903	0.532927	0	Loss	Gain
Chr2_CNVR_92	Chr2	135708477	135717145	8669	-0.9407942	0.520946	3.07E-199	Loss	Gain
Chr2_CNVR_93	Chr2	135721481	135741421	19941	-0.9553415	0.515719	0	Loss	Gain
Chr2_CNVR_94	Chr2	135742289	135764831	22543	-0.8942581	0.538024	0	Loss	Gain
Chr2_CNVR_95	Chr2	135786507	135802111	15605	-0.9653714	0.512147	0	Loss	Gain
Chr2_CNVR_96	Chr2	135809049	135821185	12137	-0.8615907	0.550345	9.32E-240	Loss	Gain
Chr2_CNVR_97	Chr2	135822921	135831589	8669	-0.8658554	0.548721	2.67E-173	Loss	Gain
Chr2_CNVR_98	Chr2	135841127	135849797	8671	-0.8235856	0.565036	4.33E-159	Loss	Gain

Chr2_CNVR_99	Chr2	135885345	135894013	8669	-0.837275	0.5597	1.18E-163	Loss	Gain
Chr2_CNVR_100	Chr2	135904419	135915689	11271	-0.899179	0.536192	1.63E-239	Loss	Gain
Chr2_CNVR_101	Chr2	136073483	136087355	13873	-0.8328441	0.561421	3.18E-258	Loss	Gain
Chr2_CNVR_102	Chr2	136098627	136108163	9537	-0.7917809	0.577631	2.75E-163	Loss	Gain
Chr2_CNVR_103	Chr2	136115099	136125503	10405	-0.8205636	0.566221	2.71E-189	Loss	Gain
Chr2_CNVR_104	Chr2	136129839	136139375	9537	-0.8436811	0.55722	3.53E-182	Loss	Gain
Chr2_CNVR_105	Chr2	136149779	136167985	18207	-0.8850956	0.541452	0	Loss	Gain
Chr2_CNVR_106	Chr2	136206135	136216537	10403	-0.8416053	0.558022	1.03E-197	Loss	Gain
Chr2_CNVR_107	Chr2	136222607	136233877	11271	-0.8813346	0.542865	1.56E-231	Loss	Gain
Chr2_CNVR_108	Chr2	136234745	136243415	8671	-0.8710752	0.546739	4.45E-175	Loss	Gain
Chr2_CNVR_109	Chr2	136244283	136253819	9537	-0.8256639	0.564222	1.49E-175	Loss	Gain
Chr2_CNVR_110	Chr2	136495713	136509583	13871	-0.8728442	0.546069	7.94E-280	Loss	Gain
Chr2_CNVR_111	Chr2	136529525	136541663	12139	-0.9861258	0.504832	1.38E-300	Loss	Gain
Chr2_CNVR_112	Chr2	136544265	136555535	11271	-0.8094659	0.570593	3.97E-200	Loss	Gain
Chr2_CNVR_113	Chr2	136567673	136579811	12139	-0.8302981	0.562413	5.05E-225	Loss	Gain
Chr2_CNVR_114	Chr2	136608423	136622293	13871	-0.8968549	0.537056	5.22E-293	Loss	Gain
Chr2_CNVR_115	Chr2	136901469	136916207	14739	-1.139294	0.453982	0	Loss	Gain
Chr2_CNVR_116	Chr2	136972563	136983833	11271	-2.280148	0.205877	0	Loss	Gain
Chr2_CNVR_6	Chr2	137002041	137015911	13871	3.314276	9.9471	0	Gain	Loss
Chr2_CNVR_117	Chr2	137030651	137043655	13005	-1.684422	0.311128	0	Loss	Gain
Chr20_CNVR_1	Chr20	16925635	16934394	8760	0.8717943	1.82994	1.21E-185	Gain	Loss
Chr20_CNVR_2	Chr20	44576575	44587086	10512	1.10961	2.15787	0	Gain	Loss
Chr20_CNVR_3	Chr20	45832759	45841518	8760	0.8691576	1.8266	1.14E-184	Gain	Loss
Chr20_CNVR_4	Chr20	62333095	62341854	8760	-0.8417989	0.557947	4.70E-165	Loss	Gain
Chr20_CNVR_5	Chr20	67241323	67252710	11388	-1.052111	0.482262	0	Loss	Gain
Chr20_CNVR_6	Chr20	70235491	70246878	11388	-0.7995439	0.574531	8.42E-196	Loss	Gain
Chr20_CNVR_7	Chr20	70250383	70260018	9636	-0.8427764	0.55757	1.03E-181	Loss	Gain
Chr20_CNVR_8	Chr20	70577131	70592898	15768	-0.8759571	0.544892	0	Loss	Gain
Chr20_CNVR_9	Chr20	70910887	70925778	14892	-0.8801676	0.543304	3.71E-301	Loss	Gain
Chr20_CNVR_10	Chr20	70949431	70964322	14892	-0.9511347	0.517225	0	Loss	Gain
Chr20_CNVR_11	Chr20	70965199	70979214	14016	-0.980629	0.506759	0	Loss	Gain
Chr20_CNVR_12	Chr20	71036155	71044914	8760	-0.8083996	0.571015	5.48E-154	Loss	Gain
Chr20_CNVR_13	Chr20	71051047	71060682	9636	-0.9433651	0.520019	1.13E-219	Loss	Gain
Chr20_CNVR_14	Chr20	71061559	71071194	9636	-0.9259915	0.526319	5.36E-213	Loss	Gain
Chr20_CNVR_15	Chr20	71085211	71105358	20148	-0.882633	0.542377	0	Loss	Gain
Chr20_CNVR_16	Chr20	71137771	71158794	21024	-0.9011631	0.535455	0	Loss	Gain
Chr20_CNVR_17	Chr20	71180695	71197338	16644	-0.8908223	0.539307	0	Loss	Gain
Chr20_CNVR_18	Chr20	71246395	71263038	16644	-0.894564	0.53791	0	Loss	Gain
Chr20_CNVR_19	Chr20	71292823	71303334	10512	-0.9875206	0.504344	3.58E-258	Loss	Gain
Chr20_CNVR_20	Chr20	71327863	71344506	16644	-0.9000765	0.535858	0	Loss	Gain
Chr20_CNVR_21	Chr20	71361151	71378670	17520	-0.9993785	0.500215	0	Loss	Gain
Chr20_CNVR_22	Chr20	71400571	71411958	11388	-0.9159423	0.529998	8.45E-247	Loss	Gain
Chr20_CNVR_23	Chr20	71419843	71441742	21900	-0.9958686	0.501434	0	Loss	Gain
Chr20_CNVR_24	Chr20	71467147	71480286	13140	-0.8120699	0.569564	9.41E-232	Loss	Gain
Chr20_CNVR_25	Chr20	71495179	71503938	8760	-0.8642075	0.549348	1.35E-172	Loss	Gain
Chr21_CNVR_7	Chr21	951775	969295	17521	-1.399186	0.379143	0	Loss	Gain
Chr21_CNVR_8	Chr21	1771711	1780471	8761	-1.429793	0.371184	0	Loss	Gain
Chr21_CNVR_9	Chr21	9609283	9641695	32413	-1.914598	0.265246	0	Loss	Gain
Chr21_CNVR_10	Chr21	20059087	20083615	24529	-1.38825	0.382028	0	Loss	Gain
Chr21_CNVR_11	Chr21	20087119	20109019	21901	-2.799482	0.143639	0	Loss	Gain
Chr21_CNVR_12	Chr21	20114275	20148439	34165	-2.194642	0.218447	0	Loss	Gain
Chr21_CNVR_1	Chr21	37111303	37125319	14017	1.03735	2.05245	0	Gain	Loss
Chr21_CNVR_2	Chr21	37127947	37141087	13141	1.014821	2.02065	0	Gain	Loss
Chr21_CNVR_13	Chr21	37431043	37442431	11389	-6.809451	0.00891561	0	Loss	Gain
Chr21_CNVR_3	Chr21	40147519	40157155	9637	0.9939048	1.99157	1.40E-266	Gain	Loss
Chr21_CNVR_4	Chr21	41978359	41987119	8761	1.03217	2.0451	1.69E-258	Gain	Loss

Chr21_CNVR_5	Chr21	53551195	53560831	9637	0.9225136	1.89541	1.49E-234	Gain	Loss
Chr21_CNVR_6	Chr21	54928267	54938779	10513	1.198148	2.29445	0	Gain	Loss
Chr21_CNVR_14	Chr21	62490775	62499535	8761	-0.9731018	0.50941	3.26E-209	Loss	Gain
Chr21_CNVR_15	Chr21	64675519	64686031	10513	-1.007876	0.497278	2.94E-265	Loss	Gain
Chr21_CNVR_16	Chr21	64687783	64698295	10513	-1.127332	0.457761	0	Loss	Gain
Chr21_CNVR_17	Chr21	65933455	65942215	8761	-0.9527933	0.516631	3.74E-202	Loss	Gain
Chr21_CNVR_18	Chr21	70409815	70420327	10513	-0.8290479	0.562901	5.44E-192	Loss	Gain
Chr21_CNVR_19	Chr21	70860955	70871467	10513	-0.7912129	0.577858	2.93E-177	Loss	Gain
Chr21_CNVR_20	Chr21	71129887	71139523	9637	-0.8307046	0.562255	9.22E-177	Loss	Gain
Chr21_CNVR_21	Chr21	71217487	71231503	14017	-0.8868519	0.540793	4.55E-286	Loss	Gain
Chr21_CNVR_22	Chr21	71505691	71533723	28033	-0.9655153	0.512095	0	Loss	Gain
Chr22_CNVR_1	Chr22	50690063	50700563	10501	-0.8605807	0.550731	1.84E-204	Loss	Gain
Chr22_CNVR_2	Chr22	58474939	58483687	8749	-0.82218	0.565587	4.46E-158	Loss	Gain
Chr22_CNVR_3	Chr22	58517813	58527437	9625	-0.8593122	0.551215	3.92E-187	Loss	Gain
Chr22_CNVR_4	Chr22	59469813	59479437	9625	-0.8485942	0.555326	3.18E-183	Loss	Gain
Chr22_CNVR_5	Chr22	60120813	60134813	14001	-0.9069695	0.533304	7.35E-297	Loss	Gain
Chr24_CNVR_4	Chr24	1009034	1023892	14859	-0.8739209	0.545662	8.66E-297	Loss	Gain
Chr24_CNVR_5	Chr24	1065844	1075458	9615	-0.8442996	0.556981	7.15E-182	Loss	Gain
Chr24_CNVR_6	Chr24	19922394	19932882	10489	-0.8627619	0.549899	1.28E-205	Loss	Gain
Chr24_CNVR_7	Chr24	19943370	19952984	9615	-0.8850084	0.541484	6.45E-197	Loss	Gain
Chr24_CNVR_8	Chr24	19968716	19977456	8741	-0.9120186	0.531441	2.00E-188	Loss	Gain
Chr24_CNVR_9	Chr24	24850880	24859620	8741	-1.210021	0.432262	5.83E-295	Loss	Gain
Chr24_CNVR_1	Chr24	44178516	44195122	16607	3.37139	10.3488	0	Gain	Loss
Chr24_CNVR_2	Chr24	56917066	56925806	8741	1.893909	3.71641	0	Gain	Loss
Chr24_CNVR_3	Chr24	56936294	56945908	9615	1.342933	2.53666	0	Gain	Loss
Chr25_CNVR_1	Chr25	2505536	2516599	11064	-1.138869	0.454115	0	Loss	Gain
Chr26_CNVR_1	Chr26	2950607	2965685	15079	0.9642435	1.95104	0	Gain	Loss
Chr26_CNVR_2	Chr26	3309841	3320485	10645	-3.443982	0.0918879	0	Loss	Gain
Chr26_CNVR_3	Chr26	25748281	25760697	12417	-0.8700115	0.547142	7.98E-243	Loss	Gain
Chr26_CNVR_4	Chr26	31579419	31598045	18627	-1.715489	0.304499	0	Loss	Gain
Chr26_CNVR_5	Chr26	51115593	51126237	10645	-0.9100368	0.532172	1.05E-224	Loss	Gain
Chr26_CNVR_6	Chr26	51175023	51183891	8869	-0.8337647	0.561063	5.52E-162	Loss	Gain
Chr26_CNVR_7	Chr26	51247757	51256625	8869	-0.878844	0.543803	5.15E-177	Loss	Gain
Chr26_CNVR_8	Chr26	51273479	51283235	9757	-0.8831413	0.542186	4.19E-196	Loss	Gain
Chr26_CNVR_9	Chr26	51495229	51504985	9757	-0.8468581	0.555994	9.69E-183	Loss	Gain
Chr26_CNVR_10	Chr26	51563529	51575059	11531	-0.867133	0.548235	2.24E-224	Loss	Gain
Chr26_CNVR_11	Chr26	51615861	51626505	10645	-0.8505102	0.554589	1.28E-200	Loss	Gain
Chr26_CNVR_12	Chr26	51657551	51667307	9757	-0.8276735	0.563437	8.97E-176	Loss	Gain
Chr27_CNVR_1	Chr27	1694754	1707388	12635	1.174462	2.25709	0	Gain	Loss
Chr27_CNVR_2	Chr27	3365898	3374542	8645	1.167616	2.2464	0	Gain	Loss
Chr27_CNVR_7	Chr27	5193318	5203292	9975	-1.342585	0.394313	0	Loss	Gain
Chr27_CNVR_8	Chr27	5203958	5212602	8645	-1.389825	0.381611	0	Loss	Gain
Chr27_CNVR_9	Chr27	5225904	5233218	7315	-1.25267	0.419671	0	Loss	Gain
Chr27_CNVR_3	Chr27	5424074	5434048	9975	2.258293	4.78425	0	Gain	Loss
Chr27_CNVR_4	Chr27	5458654	5467962	9309	1.381144	2.60475	0	Gain	Loss
Chr27_CNVR_10	Chr27	5571038	5605618	34581	-3.310061	0.100826	0	Loss	Gain
Chr27_CNVR_11	Chr27	5614928	5637538	22611	-3.592885	0.082877	0	Loss	Gain
Chr27_CNVR_12	Chr27	5641528	5664802	23275	-1.8566	0.276126	0	Loss	Gain
Chr27_CNVR_5	Chr27	5664804	5760562	95759	3.271079	9.65368	0	Gain	Loss
Chr27_CNVR_6	Chr27	5857654	5872282	14629	1.519316	2.86655	0	Gain	Loss
Chr27_CNVR_13	Chr27	5900214	5916838	16625	-1.383047	0.383408	0	Loss	Gain
Chr27_CNVR_14	Chr27	6468124	6492728	24605	-2.378992	0.192244	0	Loss	Gain
Chr27_CNVR_15	Chr27	28524178	28530828	6651	-1.866935	0.274155	0	Loss	Gain
Chr27_CNVR_16	Chr27	34199954	34208598	8645	-0.9139844	0.530717	4.88E-245	Loss	Gain
Chr28_CNVR_1	Chr28	2253257	2270707	17451	3.58101	11.9672	0	Gain	Loss
Chr28_CNVR_3	Chr28	2657955	2685377	27423	-1.107955	0.463951	0	Loss	Gain

Chr28_CNVR_4	Chr28	2708645	2721941	13297	-0.8565658	0.552266	3.49E-270	Loss	Gain
Chr28_CNVR_5	Chr28	2745209	2758505	13297	-0.8628691	0.549858	1.47E-273	Loss	Gain
Chr28_CNVR_6	Chr28	2819169	2827477	8309	-1.012474	0.495695	1.56E-223	Loss	Gain
Chr28_CNVR_7	Chr28	26557515	26569147	11633	-0.9845251	0.505392	2.90E-298	Loss	Gain
Chr28_CNVR_8	Chr28	29153559	29165191	11633	-3.384373	0.095764	0	Loss	Gain
Chr28_CNVR_2	Chr28	29617257	29626397	9141	0.8477106	1.79964	2.34E-199	Gain	Loss
Chr28_CNVR_9	Chr28	35014601	35022911	8311	-0.8647067	0.549158	2.22E-172	Loss	Gain
Chr28_CNVR_10	Chr28	35026235	35037037	10803	-0.8674315	0.548122	1.17E-224	Loss	Gain
Chr28_CNVR_11	Chr28	35042855	35051165	8311	-0.7934688	0.576955	6.53E-149	Loss	Gain
Chr29_CNVR_1	Chr29	4433563	4441123	7561	0.9309293	1.9065	2.13E-219	Gain	Loss
Chr29_CNVR_2	Chr29	9644671	9658279	13609	0.9935002	1.99101	0	Gain	Loss
Chr29_CNVR_3	Chr29	20004139	20012455	8317	1.009947	2.01384	2.53E-277	Gain	Loss
Chr29_CNVR_4	Chr29	22119427	22131523	12097	1.253479	2.38416	0	Gain	Loss
Chr29_CNVR_5	Chr29	26954803	26966899	12097	3.377507	10.3928	0	Gain	Loss
Chr29_CNVR_9	Chr29	27391015	27400843	9829	-2.129332	0.228564	0	Loss	Gain
Chr29_CNVR_6	Chr29	28026811	28040419	13609	1.753447	3.37163	0	Gain	Loss
Chr29_CNVR_7	Chr29	38780911	38788471	7561	1.014373	2.02002	3.49E-254	Gain	Loss
Chr29_CNVR_10	Chr29	39341107	39366811	25705	-4.4448	0.0459179	0	Loss	Gain
Chr29_CNVR_8	Chr29	42514795	42575275	60481	4.480784	22.328	0	Gain	Loss
Chr29_CNVR_11	Chr29	43527079	43534639	7561	-0.8418487	0.557928	2.28E-164	Loss	Gain
Chr29_CNVR_12	Chr29	46952515	46962343	9829	-0.8569274	0.552127	1.57E-219	Loss	Gain
Chr29_CNVR_13	Chr29	48365479	48377575	12097	-0.8521272	0.553967	3.71E-267	Loss	Gain
Chr29_CNVR_14	Chr29	48500047	48508363	8317	-0.8374784	0.559621	5.10E-179	Loss	Gain
Chr29_CNVR_15	Chr29	48518191	48526507	8317	-0.9067777	0.533375	1.80E-204	Loss	Gain
Chr29_CNVR_16	Chr29	50342419	50351491	9073	-0.9461608	0.519012	5.15E-239	Loss	Gain
Chr3_CNVR_1	Chr3	8518276	8539526	21251	4.621509	24.6157	0	Gain	Loss
Chr3_CNVR_2	Chr3	8612626	8670426	57801	4.194512	18.3094	0	Gain	Loss
Chr3_CNVR_3	Chr3	8732476	8752026	19551	5.215159	37.1466	0	Gain	Loss
Chr3_CNVR_12	Chr3	11866426	11887676	21251	-2.140229	0.226844	0	Loss	Gain
Chr3_CNVR_13	Chr3	11893626	11907226	13601	-2.480523	0.179179	0	Loss	Gain
Chr3_CNVR_14	Chr3	15578376	15587726	9351	-0.8034535	0.572976	3.53E-167	Loss	Gain
Chr3_CNVR_4	Chr3	21309076	21320126	11051	1.192929	2.28616	0	Gain	Loss
Chr3_CNVR_15	Chr3	28178776	28187276	8501	-0.9901801	0.503415	6.57E-216	Loss	Gain
Chr3_CNVR_5	Chr3	40003976	40013326	9351	1.030421	2.04262	5.76E-275	Gain	Loss
Chr3_CNVR_6	Chr3	46785276	46797176	11901	1.248826	2.37648	0	Gain	Loss
Chr3_CNVR_16	Chr3	47039426	47047926	8501	-4.240238	0.0529129	0	Loss	Gain
Chr3_CNVR_7	Chr3	52501526	52510026	8501	0.8366523	1.7859	5.43E-175	Gain	Loss
Chr3_CNVR_17	Chr3	54367276	54381726	14451	-2.825826	0.14104	0	Loss	Gain
Chr3_CNVR_18	Chr3	54394476	54447176	52701	-3.284923	0.102598	0	Loss	Gain
Chr3_CNVR_19	Chr3	54458226	54536426	78201	-2.983333	0.126452	0	Loss	Gain
Chr3_CNVR_8	Chr3	54765926	54799926	34001	3.165836	8.97453	0	Gain	Loss
Chr3_CNVR_9	Chr3	62693876	62710876	17001	1.330099	2.5142	0	Gain	Loss
Chr3_CNVR_10	Chr3	62821376	62829876	8501	1.051987	2.07338	6.65E-259	Gain	Loss
Chr3_CNVR_20	Chr3	99832926	99842276	9351	-0.866977	0.548295	2.37E-190	Loss	Gain
Chr3_CNVR_11	Chr3	105709826	105725126	15301	1.027767	2.03887	0	Gain	Loss
Chr3_CNVR_21	Chr3	115799326	115812076	12751	-0.833318	0.561237	6.32E-242	Loss	Gain
Chr3_CNVR_22	Chr3	119358276	119367626	9351	-0.8084347	0.571001	5.77E-169	Loss	Gain
Chr3_CNVR_23	Chr3	120278826	120295826	17001	-0.9226958	0.527522	0	Loss	Gain
Chr3_CNVR_24	Chr3	120364676	120379126	14451	-0.8929507	0.538512	5.28E-308	Loss	Gain
Chr3_CNVR_25	Chr3	120412276	120423326	11051	-0.9064694	0.533489	4.67E-242	Loss	Gain
Chr3_CNVR_26	Chr3	120492176	120500676	8501	-0.9271899	0.525882	7.84E-194	Loss	Gain
Chr3_CNVR_27	Chr3	120662176	120670676	8501	-0.909457	0.532385	1.04E-187	Loss	Gain
Chr3_CNVR_28	Chr3	120689376	120701276	11901	-0.909737	0.532282	4.39E-262	Loss	Gain
Chr3_CNVR_29	Chr3	120842376	120852576	10201	-0.8408668	0.558308	5.72E-197	Loss	Gain
Chr3_CNVR_30	Chr3	121089726	121099076	9351	-0.8197953	0.566522	4.63E-173	Loss	Gain
Chr3_CNVR_31	Chr3	121244426	121260576	16151	-0.8661557	0.548607	0	Loss	Gain

Chr3_CNVR_32	Chr3	121322626	121331126	8501	-0.8572809	0.551992	5.69E-170	Loss	Gain
Chr4_CNVR_1	Chr4	2846998	2855217	8220	1.118265	2.17086	2.57E-279	Gain	Loss
Chr4_CNVR_2	Chr4	19987342	19996383	9042	1.269025	2.40999	0	Gain	Loss
Chr4_CNVR_3	Chr4	28241866	28254195	12330	4.178314	18.105	0	Gain	Loss
Chr4_CNVR_7	Chr4	31884970	31895655	10686	-2.633829	0.161116	0	Loss	Gain
Chr4_CNVR_8	Chr4	43480924	43490787	9864	-1.188825	0.43866	0	Loss	Gain
Chr4_CNVR_9	Chr4	47480776	47498859	18084	-1.138972	0.454083	0	Loss	Gain
Chr4_CNVR_10	Chr4	50800012	50810697	10686	-1.067424	0.47717	0	Loss	Gain
Chr4_CNVR_11	Chr4	51982048	51990267	8220	-1.220362	0.429175	5.06E-301	Loss	Gain
Chr4_CNVR_4	Chr4	54383932	54396261	12330	0.9657789	1.95312	0	Gain	Loss
Chr4_CNVR_12	Chr4	69352552	69360771	8220	-0.8412765	0.558149	5.28E-165	Loss	Gain
Chr4_CNVR_5	Chr4	73982878	73996851	13974	1.168092	2.24714	0	Gain	Loss
Chr4_CNVR_13	Chr4	75413158	75423021	9864	-0.8581971	0.551641	1.97E-204	Loss	Gain
Chr4_CNVR_14	Chr4	77798602	77811753	13152	-0.8422229	0.557783	2.94E-263	Loss	Gain
Chr4_CNVR_15	Chr4	86733742	86745249	11508	-4.772185	0.0365956	0	Loss	Gain
Chr4_CNVR_16	Chr4	93070540	93079581	9042	-0.830251	0.562431	3.03E-177	Loss	Gain
Chr4_CNVR_17	Chr4	96123448	96132489	9042	-0.7980058	0.575144	1.54E-165	Loss	Gain
Chr4_CNVR_18	Chr4	98734120	98743983	9864	-1.105003	0.464902	0	Loss	Gain
Chr4_CNVR_19	Chr4	106513528	106526679	13152	-0.8450438	0.556694	8.97E-265	Loss	Gain
Chr4_CNVR_6	Chr4	109595206	109605891	10686	1.298159	2.45915	0	Gain	Loss
Chr4_CNVR_20	Chr4	114337324	114347187	9864	-0.8281328	0.563258	2.53E-192	Loss	Gain
Chr4_CNVR_21	Chr4	114361162	114376779	15618	-0.9638444	0.512689	0	Loss	Gain
Chr4_CNVR_22	Chr4	114378424	114388287	9864	-0.9010383	0.535501	5.13E-222	Loss	Gain
Chr4_CNVR_23	Chr4	114419524	114437607	18084	-0.9058075	0.533734	0	Loss	Gain
Chr4_CNVR_24	Chr4	114456514	114472953	16440	-0.9209224	0.528171	0	Loss	Gain
Chr4_CNVR_25	Chr4	114473776	114489393	15618	-0.9392124	0.521518	0	Loss	Gain
Chr4_CNVR_26	Chr4	114531316	114541179	9864	-0.9022565	0.535049	1.60E-222	Loss	Gain
Chr4_CNVR_27	Chr4	114566662	114577347	10686	-0.8184971	0.567032	5.09E-204	Loss	Gain
Chr4_CNVR_28	Chr4	115054930	115063971	9042	-0.7772301	0.583486	4.00E-158	Loss	Gain
Chr4_CNVR_29	Chr4	115096030	115122333	26304	-0.8863337	0.540987	0	Loss	Gain
Chr4_CNVR_30	Chr4	117887542	117899049	11508	-0.8410406	0.558241	4.61E-230	Loss	Gain
Chr4_CNVR_31	Chr4	117908092	117917955	9864	-0.9189949	0.528877	1.72E-229	Loss	Gain
Chr4_CNVR_32	Chr4	117932752	117942615	9864	-0.871016	0.546762	1.18E-209	Loss	Gain
Chr4_CNVR_33	Chr4	118342930	118353615	10686	-0.8467185	0.556048	2.93E-216	Loss	Gain
Chr4_CNVR_34	Chr4	118379920	118388961	9042	-0.865743	0.548764	2.20E-190	Loss	Gain
Chr4_CNVR_35	Chr4	118405402	118416909	11508	-0.8236242	0.565021	6.25E-222	Loss	Gain
Chr4_CNVR_36	Chr4	119114788	119123007	8220	-0.821381	0.5659	2.27E-158	Loss	Gain
Chr4_CNVR_37	Chr4	119151778	119164929	13152	-0.8711024	0.546729	7.07E-279	Loss	Gain
Chr4_CNVR_38	Chr4	119183014	119196165	13152	-0.839435	0.558862	9.18E-262	Loss	Gain
Chr4_CNVR_39	Chr4	119439478	119449341	9864	-0.8945197	0.537926	2.57E-219	Loss	Gain
Chr4_CNVR_40	Chr4	119492908	119507703	14796	-0.889772	0.539699	0	Loss	Gain
Chr4_CNVR_41	Chr4	119540584	119571819	31236	-0.9168608	0.52966	0	Loss	Gain
Chr4_CNVR_42	Chr4	119576752	119602233	25482	-0.8396278	0.558788	0	Loss	Gain
Chr4_CNVR_43	Chr4	119676214	119686899	10686	-0.8437295	0.557201	5.93E-215	Loss	Gain
Chr4_CNVR_44	Chr4	119698408	119709915	11508	-0.8900966	0.539578	2.19E-253	Loss	Gain
Chr4_CNVR_45	Chr4	119710738	119723067	12330	-1.532244	0.345739	0	Loss	Gain
Chr4_CNVR_46	Chr4	119728000	119736219	8220	-0.8273516	0.563563	2.36E-160	Loss	Gain
Chr4_CNVR_47	Chr4	119737864	119748549	10686	-0.886669	0.540861	6.59E-234	Loss	Gain
Chr5_CNVR_1	Chr5	4120849	4130353	9505	1.72396	3.30342	0	Gain	Loss
Chr5_CNVR_21	Chr5	23331889	23341393	9505	-0.8523353	0.553887	4.43E-185	Loss	Gain
Chr5_CNVR_22	Chr5	26205553	26215057	9505	-0.7897684	0.578437	2.15E-162	Loss	Gain
Chr5_CNVR_2	Chr5	27799633	27810865	11233	2.268466	4.81811	0	Gain	Loss
Chr5_CNVR_23	Chr5	27968977	27977617	8641	-0.8129275	0.569226	2.32E-155	Loss	Gain
Chr5_CNVR_24	Chr5	31504465	31515697	11233	-0.9289812	0.525229	2.10E-252	Loss	Gain
Chr5_CNVR_3	Chr5	44760817	44771185	10369	0.8394152	1.78932	1.36E-209	Gain	Loss
Chr5_CNVR_25	Chr5	56548369	56557009	8641	-0.9451679	0.519369	2.75E-200	Loss	Gain

Chr5_CNVR_26	Chr5	56603665	56617489	13825	-0.872269	0.546287	5.31E-279	Loss	Gain
Chr5_CNVR_27	Chr5	58031857	58040497	8641	-0.9298646	0.524908	5.97E-195	Loss	Gain
Chr5_CNVR_4	Chr5	58067281	58075921	8641	0.894571	1.85906	1.49E-195	Gain	Loss
Chr5_CNVR_5	Chr5	58084561	58095793	11233	0.9283803	1.90314	2.33E-270	Gain	Loss
Chr5_CNVR_6	Chr5	58141585	58152817	11233	2.663596	6.3361	0	Gain	Loss
Chr5_CNVR_28	Chr5	58230577	58251313	20737	-1.668321	0.314619	0	Loss	Gain
Chr5_CNVR_7	Chr5	58964977	59028913	63937	1.198455	2.29494	0	Gain	Loss
Chr5_CNVR_8	Chr5	59032369	59091985	59617	1.20798	2.31014	0	Gain	Loss
Chr5_CNVR_9	Chr5	59100625	59117905	17281	1.203126	2.30238	0	Gain	Loss
Chr5_CNVR_10	Chr5	59119633	59132593	12961	1.181467	2.26807	0	Gain	Loss
Chr5_CNVR_11	Chr5	59138641	59152465	13825	1.184674	2.27312	0	Gain	Loss
Chr5_CNVR_12	Chr5	59153329	59237137	83809	1.469787	2.76981	0	Gain	Loss
Chr5_CNVR_29	Chr5	59424625	59435857	11233	-0.8653897	0.548898	4.60E-224	Loss	Gain
Chr5_CNVR_30	Chr5	60128785	60147793	19009	-1.19689	0.436215	0	Loss	Gain
Chr5_CNVR_13	Chr5	63419761	63434449	14689	1.041008	2.05766	0	Gain	Loss
Chr5_CNVR_14	Chr5	68968369	68977009	8641	0.9771788	1.96861	1.90E-227	Gain	Loss
Chr5_CNVR_31	Chr5	74631889	74666449	34561	-1.584191	0.333512	0	Loss	Gain
Chr5_CNVR_32	Chr5	74819377	74831473	12097	-1.257185	0.418359	0	Loss	Gain
Chr5_CNVR_33	Chr5	76145617	76155121	9505	-0.7806467	0.582106	3.70E-159	Loss	Gain
Chr5_CNVR_15	Chr5	76417777	76429009	11233	3.317993	9.97276	0	Gain	Loss
Chr5_CNVR_34	Chr5	99607537	99619633	12097	-1.73826	0.299731	0	Loss	Gain
Chr5_CNVR_16	Chr5	99658513	99672337	13825	0.9756307	1.9665	0	Gain	Loss
Chr5_CNVR_17	Chr5	99674065	99687025	12961	1.048429	2.06828	0	Gain	Loss
Chr5_CNVR_18	Chr5	99727633	99741457	13825	1.119978	2.17344	0	Gain	Loss
Chr5_CNVR_35	Chr5	99808849	99820081	11233	-1.336424	0.396001	0	Loss	Gain
Chr5_CNVR_36	Chr5	103357297	103366801	9505	-3.433663	0.0925474	0	Loss	Gain
Chr5_CNVR_37	Chr5	103371121	103381489	10369	-0.9126557	0.531206	2.30E-226	Loss	Gain
Chr5_CNVR_38	Chr5	103398769	103427281	28513	-2.783438	0.145245	0	Loss	Gain
Chr5_CNVR_39	Chr5	104013937	104022577	8641	-0.7840878	0.580719	5.56E-146	Loss	Gain
Chr5_CNVR_40	Chr5	106852177	106864273	12097	-0.8744401	0.545466	2.00E-245	Loss	Gain
Chr5_CNVR_41	Chr5	107032753	107049169	16417	-0.8746888	0.545371	0	Loss	Gain
Chr5_CNVR_42	Chr5	107064721	107091505	26785	-0.8721358	0.546337	0	Loss	Gain
Chr5_CNVR_43	Chr5	107092369	107103601	11233	-0.8263748	0.563945	4.14E-207	Loss	Gain
Chr5_CNVR_44	Chr5	107110513	107128657	18145	-0.8897706	0.5397	0	Loss	Gain
Chr5_CNVR_45	Chr5	107131249	107141617	10369	-0.8968223	0.537068	7.91E-220	Loss	Gain
Chr5_CNVR_46	Chr5	107158897	107169265	10369	-0.8745099	0.545439	1.08E-210	Loss	Gain
Chr5_CNVR_47	Chr5	107615089	107628913	13825	-0.8290573	0.562897	7.79E-256	Loss	Gain
Chr5_CNVR_48	Chr5	107641873	107653969	12097	-0.8615157	0.550374	2.63E-239	Loss	Gain
Chr5_CNVR_49	Chr5	109243729	109256689	12961	-0.8825044	0.542425	6.49E-267	Loss	Gain
Chr5_CNVR_50	Chr5	109715473	109733617	18145	-0.8515883	0.554174	0	Loss	Gain
Chr5_CNVR_51	Chr5	109734481	109745713	11233	-0.8450662	0.556685	3.46E-215	Loss	Gain
Chr5_CNVR_52	Chr5	110009233	110019601	10369	-0.8812046	0.542914	2.00E-213	Loss	Gain
Chr5_CNVR_53	Chr5	111524689	111533329	8641	-0.8879612	0.540377	1.66E-180	Loss	Gain
Chr5_CNVR_54	Chr5	112210705	112219345	8641	-2.655855	0.158675	0	Loss	Gain
Chr5_CNVR_55	Chr5	112977937	112986577	8641	-0.8801711	0.543303	7.53E-178	Loss	Gain
Chr5_CNVR_56	Chr5	114602257	114614353	12097	-0.873544	0.545804	5.33E-245	Loss	Gain
Chr5_CNVR_57	Chr5	114698161	114708529	10369	-0.8787228	0.543849	2.06E-212	Loss	Gain
Chr5_CNVR_58	Chr5	114730993	114741361	10369	-0.8034671	0.572971	2.62E-182	Loss	Gain
Chr5_CNVR_59	Chr5	114812209	114821713	9505	-0.7816463	0.581703	1.64E-159	Loss	Gain
Chr5_CNVR_60	Chr5	115480945	115489585	8641	-0.8843441	0.541734	2.85E-179	Loss	Gain
Chr5_CNVR_61	Chr5	115514641	115523281	8641	-0.8854807	0.541307	1.17E-179	Loss	Gain
Chr5_CNVR_62	Chr5	115556977	115565617	8641	-1.001124	0.499611	4.78E-220	Loss	Gain
Chr5_CNVR_63	Chr5	115574257	115583761	9505	-0.904299	0.534292	1.65E-204	Loss	Gain
Chr5_CNVR_64	Chr5	115762609	115772977	10369	-0.8146558	0.568544	1.05E-186	Loss	Gain
Chr5_CNVR_65	Chr5	115929361	115940593	11233	-0.8930616	0.53847	2.69E-236	Loss	Gain
Chr5_CNVR_66	Chr5	115994161	116014033	19873	-0.8869888	0.540742	0	Loss	Gain

Chr5_CNVR_67	Chr5	116046865	116056369	9505	-0.8733495	0.545878	7.05E-193	Loss	Gain
Chr5_CNVR_68	Chr5	116058097	116066737	8641	-0.8646887	0.549165	1.34E-172	Loss	Gain
Chr5_CNVR_69	Chr5	116078833	116091793	12961	-0.9398126	0.521301	1.46E-296	Loss	Gain
Chr5_CNVR_70	Chr5	116098705	116107345	8641	-0.898617	0.536401	3.74E-184	Loss	Gain
Chr5_CNVR_71	Chr5	116122897	116131537	8641	-0.8737829	0.545714	1.12E-175	Loss	Gain
Chr5_CNVR_72	Chr5	116133265	116144497	11233	-0.8604738	0.550772	6.58E-222	Loss	Gain
Chr5_CNVR_73	Chr5	116170417	116181649	11233	-0.8900356	0.539601	5.97E-235	Loss	Gain
Chr5_CNVR_19	Chr5	117385201	117393841	8641	1.295167	2.45405	0	Gain	Loss
Chr5_CNVR_20	Chr5	117401617	117417169	15553	1.771659	3.41446	0	Gain	Loss
Chr5_CNVR_74	Chr5	117739441	117750673	11233	-0.8564388	0.552314	3.84E-220	Loss	Gain
Chr5_CNVR_75	Chr5	117765361	117774001	8641	-0.9109359	0.53184	2.15E-188	Loss	Gain
Chr5_CNVR_76	Chr5	117774865	117783505	8641	-0.9003211	0.535767	9.72E-185	Loss	Gain
Chr5_CNVR_77	Chr5	117805105	117823249	18145	-0.899316	0.536141	0	Loss	Gain
Chr5_CNVR_78	Chr5	117871633	117881137	9505	-0.9573163	0.515014	8.63E-225	Loss	Gain
Chr5_CNVR_79	Chr5	117952849	117968401	15553	-0.9247612	0.526768	0	Loss	Gain
Chr5_CNVR_80	Chr5	118011601	118021105	9505	-0.9485796	0.518142	2.03E-221	Loss	Gain
Chr5_CNVR_81	Chr5	118032337	118045297	12961	-0.8825621	0.542403	6.07E-267	Loss	Gain
Chr5_CNVR_82	Chr5	118060849	118072081	11233	-0.9144852	0.530533	7.13E-246	Loss	Gain
Chr5_CNVR_83	Chr5	118098865	118108369	9505	-0.8358498	0.560253	5.06E-179	Loss	Gain
Chr5_CNVR_84	Chr5	118117873	118129105	11233	-0.8376687	0.559547	5.58E-212	Loss	Gain
Chr5_CNVR_85	Chr5	118158481	118168849	10369	-0.8487559	0.555263	2.82E-200	Loss	Gain
Chr5_CNVR_86	Chr5	118177489	118190449	12961	-0.8539772	0.553257	1.92E-252	Loss	Gain
Chr5_CNVR_87	Chr5	119500273	119508913	8641	-0.9072507	0.5332	4.02E-187	Loss	Gain
Chr5_CNVR_88	Chr5	119543473	119562481	19009	-0.8757347	0.544976	0	Loss	Gain
Chr5_CNVR_89	Chr5	119570257	119585809	15553	-0.8807686	0.543078	0	Loss	Gain
Chr5_CNVR_90	Chr5	119887345	119899441	12097	-0.8193248	0.566707	1.27E-219	Loss	Gain
Chr5_CNVR_91	Chr5	120853297	120861937	8641	-0.9170867	0.529577	1.61E-190	Loss	Gain
Chr5_CNVR_92	Chr5	120865393	120877489	12097	-0.9088315	0.532616	6.85E-262	Loss	Gain
Chr5_CNVR_93	Chr5	120893041	120909457	16417	-0.8729798	0.546018	0	Loss	Gain
Chr5_CNVR_94	Chr5	120911185	120924145	12961	-0.8823533	0.542482	7.76E-267	Loss	Gain
Chr6_CNVR_1	Chr6	2667601	2675601	8001	6.081313	67.7108	0	Gain	Loss
Chr6_CNVR_9	Chr6	9093201	9104401	11201	-3.575161	0.0839014	0	Loss	Gain
Chr6_CNVR_10	Chr6	23130801	23141201	10401	-3.114597	0.115455	0	Loss	Gain
Chr6_CNVR_2	Chr6	36456401	36464401	8001	2.300488	4.92624	0	Gain	Loss
Chr6_CNVR_3	Chr6	38832401	38842001	9601	1.036748	2.0516	2.51E-291	Gain	Loss
Chr6_CNVR_4	Chr6	53174801	53185201	10401	3.63668	12.438	0	Gain	Loss
Chr6_CNVR_11	Chr6	56225201	56235601	10401	-1.086203	0.470999	0	Loss	Gain
Chr6_CNVR_5	Chr6	58298001	58308401	10401	0.9706519	1.95973	1.12E-282	Gain	Loss
Chr6_CNVR_6	Chr6	60766801	60775601	8801	0.9259921	1.89999	3.51E-221	Gain	Loss
Chr6_CNVR_7	Chr6	80735601	80757201	21601	1.227916	2.34228	0	Gain	Loss
Chr6_CNVR_8	Chr6	86131601	86150001	18401	1.184984	2.27361	0	Gain	Loss
Chr6_CNVR_12	Chr6	88681201	88694001	12801	-1.327714	0.398399	0	Loss	Gain
Chr6_CNVR_13	Chr6	104630801	104653201	22401	-0.8521651	0.553953	0	Loss	Gain
Chr6_CNVR_14	Chr6	104656401	104666001	9601	-0.8857057	0.541223	7.57E-216	Loss	Gain
Chr6_CNVR_15	Chr6	106075601	106088401	12801	-0.810446	0.570206	1.99E-246	Loss	Gain
Chr6_CNVR_16	Chr6	106767601	106775601	8001	-0.8547203	0.552973	1.26E-169	Loss	Gain
Chr6_CNVR_17	Chr6	106810801	106823601	12801	-0.9032777	0.534671	8.25E-297	Loss	Gain
Chr6_CNVR_18	Chr6	106824401	106858801	34401	-0.9437056	0.519896	0	Loss	Gain
Chr6_CNVR_19	Chr6	106861201	106870001	8801	-1.032519	0.488856	2.27E-255	Loss	Gain
Chr6_CNVR_20	Chr6	106897201	106906801	9601	-0.8981638	0.536569	5.16E-221	Loss	Gain
Chr6_CNVR_21	Chr6	106917201	106932401	15201	-0.9244814	0.52687	0	Loss	Gain
Chr6_CNVR_22	Chr6	106933201	106943601	10401	-0.8667445	0.548383	3.31E-225	Loss	Gain
Chr6_CNVR_23	Chr6	106944401	106960401	16001	-0.9343738	0.52327	0	Loss	Gain
Chr6_CNVR_24	Chr6	106962001	106971601	9601	-0.9209167	0.528173	1.59E-230	Loss	Gain
Chr6_CNVR_25	Chr6	106983601	106998001	14401	-0.9797077	0.507082	0	Loss	Gain
Chr6_CNVR_26	Chr6	107016401	107033201	16801	-1.074183	0.47494	0	Loss	Gain

Chr6_CNVR_27	Chr6	107034001	107050001	16001	-0.9368736	0.522364	0	Loss	Gain
Chr6_CNVR_28	Chr6	107059601	107076401	16801	-0.9080539	0.532903	0	Loss	Gain
Chr6_CNVR_29	Chr6	107086001	107108401	22401	-0.8919671	0.538879	0	Loss	Gain
Chr6_CNVR_30	Chr6	107155601	107177201	21601	-0.8729428	0.546032	0	Loss	Gain
Chr6_CNVR_31	Chr6	107187601	107208401	20801	-0.9336012	0.52355	0	Loss	Gain
Chr6_CNVR_32	Chr6	107238001	107249201	11201	-0.9767684	0.508117	2.86E-296	Loss	Gain
Chr6_CNVR_33	Chr6	107250001	107261201	11201	-0.9839123	0.505607	7.52E-300	Loss	Gain
Chr6_CNVR_34	Chr6	107262801	107275601	12801	-1.031831	0.489089	0	Loss	Gain
Chr6_CNVR_35	Chr6	107297201	107306001	8801	-0.9950992	0.501701	1.77E-240	Loss	Gain
Chr6_CNVR_36	Chr6	107314001	107326001	12001	-0.8954489	0.53758	2.53E-274	Loss	Gain
Chr6_CNVR_37	Chr6	107673201	107681201	8001	-0.9049568	0.534049	6.95E-187	Loss	Gain
Chr6_CNVR_38	Chr6	107687601	107702801	15201	-0.854921	0.552896	0	Loss	Gain
Chr6_CNVR_39	Chr6	107754801	107772401	17601	-0.9100095	0.532182	0	Loss	Gain
Chr6_CNVR_40	Chr6	107775601	107784401	8801	-0.9495748	0.517785	1.36E-222	Loss	Gain
Chr6_CNVR_41	Chr6	107786801	107795601	8801	-1.000432	0.49985	1.37E-242	Loss	Gain
Chr6_CNVR_42	Chr6	108086001	108094001	8001	-0.8611568	0.550511	8.21E-172	Loss	Gain
Chr6_CNVR_43	Chr6	108349201	108361201	12001	-0.8330558	0.561339	1.93E-242	Loss	Gain
Chr6_CNVR_44	Chr6	108366001	108376401	10401	-0.9416599	0.520634	4.86E-259	Loss	Gain
Chr6_CNVR_45	Chr6	108406001	108418001	12001	-0.8985475	0.536427	6.21E-276	Loss	Gain
Chr6_CNVR_46	Chr6	108426001	108437201	11201	-0.8562996	0.552368	2.17E-237	Loss	Gain
Chr6_CNVR_47	Chr6	108954001	108962001	8001	-0.8340875	0.560938	1.15E-162	Loss	Gain
Chr6_CNVR_48	Chr6	109002801	109010801	8001	-0.8601293	0.550903	1.84E-171	Loss	Gain
Chr6_CNVR_49	Chr6	109036401	109049201	12801	-0.9562099	0.515409	0	Loss	Gain
Chr6_CNVR_50	Chr6	109078001	109087601	9601	-0.9498578	0.517683	9.31E-243	Loss	Gain
Chr6_CNVR_51	Chr6	109106001	109118001	12001	-0.9178142	0.52931	5.41E-286	Loss	Gain
Chr6_CNVR_52	Chr6	109342001	109350001	8001	-0.9897437	0.503567	6.98E-217	Loss	Gain
Chr6_CNVR_53	Chr6	109371601	109382801	11201	-0.8614428	0.550402	7.74E-240	Loss	Gain
Chr6_CNVR_54	Chr6	109400401	109410001	9601	-0.8368791	0.559853	6.52E-196	Loss	Gain
Chr6_CNVR_55	Chr6	109445201	109454801	9601	-0.9764588	0.508226	4.04E-254	Loss	Gain
Chr6_CNVR_56	Chr6	109474001	109486001	12001	-0.9414152	0.520722	1.98E-298	Loss	Gain
Chr6_CNVR_57	Chr6	109502001	109510801	8801	-1.053811	0.481694	6.54E-264	Loss	Gain
Chr6_CNVR_58	Chr6	109520401	109535601	15201	-0.9301393	0.524808	0	Loss	Gain
Chr6_CNVR_59	Chr6	109546801	109562001	15201	-0.9118627	0.531498	0	Loss	Gain
Chr6_CNVR_60	Chr6	109567601	109578001	10401	-0.9623045	0.513236	1.49E-268	Loss	Gain
Chr6_CNVR_61	Chr6	109591601	109626001	34401	-0.8844689	0.541687	0	Loss	Gain
Chr6_CNVR_62	Chr6	109719601	109736401	16801	-0.9216883	0.527891	0	Loss	Gain
Chr6_CNVR_63	Chr6	109737201	109748401	11201	-0.8742183	0.545549	6.06E-246	Loss	Gain
Chr6_CNVR_64	Chr6	109764401	109773201	8801	-0.8695603	0.547314	6.12E-192	Loss	Gain
Chr6_CNVR_65	Chr6	109810801	109819601	8801	-0.8969519	0.53702	2.62E-202	Loss	Gain
Chr6_CNVR_66	Chr6	109850801	109864401	13601	-0.9210356	0.52813	0	Loss	Gain
Chr6_CNVR_67	Chr6	118199601	118207601	8001	-0.8993966	0.536111	5.93E-185	Loss	Gain
Chr6_CNVR_68	Chr6	118356401	118365201	8801	-0.8611542	0.550512	8.72E-189	Loss	Gain
Chr6_CNVR_69	Chr6	118398001	118410801	12801	-0.8865997	0.540887	1.41E-287	Loss	Gain
Chr6_CNVR_70	Chr6	118456401	118466001	9601	-0.8305872	0.5623	2.21E-193	Loss	Gain
Chr6_CNVR_71	Chr6	118511601	118520401	8801	-0.9305102	0.524673	3.41E-215	Loss	Gain
Chr6_CNVR_72	Chr6	118521201	118531601	10401	-1.001572	0.499455	7.69E-287	Loss	Gain
Chr6_CNVR_73	Chr6	118532401	118543601	11201	-0.9153789	0.530205	7.03E-266	Loss	Gain
Chr6_CNVR_74	Chr6	118544401	118570001	25601	-0.9274489	0.525787	0	Loss	Gain
Chr6_CNVR_75	Chr6	118580401	118601201	20801	-0.9050323	0.534021	0	Loss	Gain
Chr6_CNVR_76	Chr6	118609201	118617201	8001	-0.8895278	0.539791	1.54E-181	Loss	Gain
Chr6_CNVR_77	Chr6	118630001	118642001	12001	-0.9192379	0.528788	9.69E-287	Loss	Gain
Chr6_CNVR_78	Chr6	118666801	118679601	12801	-0.9130071	0.531077	3.15E-302	Loss	Gain
Chr6_CNVR_79	Chr6	118688401	118697201	8801	-0.8820745	0.542587	1.17E-196	Loss	Gain
Chr6_CNVR_80	Chr6	118730801	118747601	16801	-0.9207281	0.528242	0	Loss	Gain
Chr6_CNVR_81	Chr6	118770001	118784401	14401	-0.8731035	0.545971	0	Loss	Gain
Chr6_CNVR_82	Chr6	118795601	118810801	15201	-0.9041117	0.534362	0	Loss	Gain

Chr6_CNVR_83	Chr6	118815601	118824401	8801	-0.9863442	0.504755	5.12E-237	Loss	Gain
Chr6_CNVR_84	Chr6	118845201	118870801	25601	-0.8997159	0.535992	0	Loss	Gain
Chr6_CNVR_85	Chr6	118880401	118888401	8001	-0.9007505	0.535608	2.01E-185	Loss	Gain
Chr6_CNVR_86	Chr6	118891601	118900401	8801	-0.9460303	0.519059	3.25E-221	Loss	Gain
Chr6_CNVR_87	Chr6	118904401	118913201	8801	-0.8799199	0.543398	7.62E-196	Loss	Gain
Chr6_CNVR_88	Chr6	119055601	119066801	11201	-0.8636204	0.549572	7.10E-241	Loss	Gain
Chr6_CNVR_89	Chr6	119093201	119114001	20801	-0.8545753	0.553028	0	Loss	Gain
Chr6_CNVR_90	Chr6	119130801	119142001	11201	-0.8512799	0.554293	5.21E-235	Loss	Gain
Chr6_CNVR_91	Chr6	119143601	119157201	13601	-0.9549759	0.51585	0	Loss	Gain
Chr6_CNVR_92	Chr6	119198001	119241201	43201	-0.9967645	0.501123	0	Loss	Gain
Chr6_CNVR_93	Chr6	119242001	119254801	12801	-0.9638915	0.512672	0	Loss	Gain
Chr6_CNVR_94	Chr6	119270801	119278801	8001	-1.065768	0.477718	1.72E-244	Loss	Gain
Chr6_CNVR_95	Chr6	119279601	119304401	24801	-0.9136625	0.530836	0	Loss	Gain
Chr6_CNVR_96	Chr6	119305201	119314001	8801	-0.8684013	0.547753	1.67E-191	Loss	Gain
Chr6_CNVR_97	Chr6	119321201	119330001	8801	-0.9487063	0.518097	2.95E-222	Loss	Gain
Chr6_CNVR_98	Chr6	119338801	119346801	8001	-0.9268176	0.526017	1.60E-194	Loss	Gain
Chr6_CNVR_99	Chr6	119353201	119365201	12001	-1.006853	0.497631	0	Loss	Gain
Chr6_CNVR_100	Chr6	119369201	119379601	10401	-0.9056635	0.533787	1.22E-242	Loss	Gain
Chr6_CNVR_101	Chr6	119392401	119401201	8801	-0.9547122	0.515945	1.34E-224	Loss	Gain
Chr6_CNVR_102	Chr6	119408401	119431601	23201	-0.9027678	0.53486	0	Loss	Gain
Chr6_CNVR_103	Chr6	119434001	119442001	8001	-0.9873591	0.5044	5.01E-216	Loss	Gain
Chr8_CNVR_1	Chr8	14241753	14249593	7841	2.512492	5.70605	0	Gain	Loss
Chr8_CNVR_14	Chr8	15646681	15657657	10977	-1.357624	0.390224	0	Loss	Gain
Chr8_CNVR_2	Chr8	23173081	23185625	12545	1.319173	2.49523	0	Gain	Loss
Chr8_CNVR_3	Chr8	26915897	26926873	10977	3.305223	9.88488	0	Gain	Loss
Chr8_CNVR_4	Chr8	34624185	34638297	14113	1.386395	2.61425	0	Gain	Loss
Chr8_CNVR_5	Chr8	34639865	34669657	29793	1.296963	2.45711	0	Gain	Loss
Chr8_CNVR_6	Chr8	35137705	35146329	8625	0.8510869	1.80386	8.72E-198	Gain	Loss
Chr8_CNVR_7	Chr8	35660633	35668473	7841	0.9131651	1.88317	1.94E-203	Gain	Loss
Chr8_CNVR_8	Chr8	39006745	39014585	7841	1.134591	2.19556	3.38E-292	Gain	Loss
Chr8_CNVR_9	Chr8	44936921	44948681	11761	3.824135	14.1638	0	Gain	Loss
Chr8_CNVR_15	Chr8	60536169	60551849	15681	-1.188888	0.438641	0	Loss	Gain
Chr8_CNVR_10	Chr8	61629065	61636905	7841	0.8957664	1.8606	8.71E-197	Gain	Loss
Chr8_CNVR_11	Chr8	61640041	61651017	10977	0.9461727	1.92675	7.59E-302	Gain	Loss
Chr8_CNVR_12	Chr8	66197433	66208409	10977	3.287822	9.76637	0	Gain	Loss
Chr8_CNVR_16	Chr8	69977097	69984937	7841	-0.8813153	0.542872	3.79E-178	Loss	Gain
Chr8_CNVR_17	Chr8	70692889	70703081	10193	-0.9751869	0.508674	2.84E-273	Loss	Gain
Chr8_CNVR_18	Chr8	70812057	70821465	9409	-1.025654	0.491188	6.09E-274	Loss	Gain
Chr8_CNVR_19	Chr8	70829305	70840281	10977	-1.500493	0.353433	0	Loss	Gain
Chr8_CNVR_20	Chr8	70841849	70874777	32929	-2.140909	0.226737	0	Loss	Gain
Chr8_CNVR_21	Chr8	70887321	70901433	14113	-1.074851	0.47472	0	Loss	Gain
Chr8_CNVR_22	Chr8	70972777	71004137	31361	-1.177253	0.442193	0	Loss	Gain
Chr8_CNVR_23	Chr8	71015113	71049609	34497	-1.252025	0.419858	0	Loss	Gain
Chr8_CNVR_24	Chr8	71051961	71076265	24305	-1.204397	0.433951	0	Loss	Gain
Chr8_CNVR_25	Chr8	71086457	71104489	18033	-1.017629	0.493927	0	Loss	Gain
Chr8_CNVR_26	Chr8	71105273	71135065	29793	-1.033346	0.488576	0	Loss	Gain
Chr8_CNVR_27	Chr8	71150745	71169561	18817	-1.35212	0.391716	0	Loss	Gain
Chr8_CNVR_28	Chr8	71175049	71198569	23521	-1.650903	0.318441	0	Loss	Gain
Chr8_CNVR_29	Chr8	71231497	71242473	10977	-1.194546	0.436924	0	Loss	Gain
Chr8_CNVR_30	Chr8	77274569	77284761	10193	-0.8864884	0.540929	2.97E-233	Loss	Gain
Chr8_CNVR_31	Chr8	77946457	77958217	11761	-3.325323	0.099765	0	Loss	Gain
Chr8_CNVR_32	Chr8	83232969	83259625	26657	-1.307586	0.403996	0	Loss	Gain
Chr8_CNVR_33	Chr8	91971433	91979273	7841	-5.105314	0.0290501	0	Loss	Gain
Chr8_CNVR_13	Chr8	93817753	93825593	7841	1.065654	2.09312	6.54E-264	Gain	Loss
Chr8_CNVR_34	Chr8	105235929	105245337	9409	-0.7958024	0.576023	2.97E-179	Loss	Gain
Chr8_CNVR_35	Chr8	105261801	105271993	10193	-0.9301271	0.524812	9.06E-253	Loss	Gain

Chr8_CNVR_36	Chr8	110998329	111006169	7841	-1.290857	0.408708	0	Loss	Gain
Chr8_CNVR_37	Chr8	112999097	113010857	11761	-0.8679724	0.547916	2.17E-259	Loss	Gain
Chr8_CNVR_38	Chr8	113033593	113044569	10977	-0.8422149	0.557787	3.82E-230	Loss	Gain
Chr9_CNVR_5	Chr9	422	11367	10946	-1.032168	0.488975	2.56E-301	Loss	Gain
Chr9_CNVR_6	Chr9	42029694	42042323	12630	-1.710352	0.305586	0	Loss	Gain
Chr9_CNVR_1	Chr9	48624238	48633499	9262	0.9385995	1.91667	9.07E-227	Gain	Loss
Chr9_CNVR_2	Chr9	55697038	55705457	8420	0.949645	1.9314	1.80E-210	Gain	Loss
Chr9_CNVR_3	Chr9	56012788	56027101	14314	1.356452	2.56055	0	Gain	Loss
Chr9_CNVR_7	Chr9	79748768	79760555	11788	-1.037646	0.487122	0	Loss	Gain
Chr9_CNVR_4	Chr9	88307698	88324537	16840	1.017606	2.02456	0	Gain	Loss
Chr9_CNVR_8	Chr9	96037258	96045677	8420	-0.7809967	0.581965	3.23E-145	Loss	Gain
Chr9_CNVR_9	Chr9	97695156	97706101	10946	-0.8533063	0.553515	2.40E-219	Loss	Gain
Chr9_CNVR_10	Chr9	103139528	103149631	10104	-0.8738598	0.545685	4.88E-211	Loss	Gain
Chr9_CNVR_11	Chr9	103515060	103528531	13472	-0.8595145	0.551138	7.64E-273	Loss	Gain
Chr9_CNVR_12	Chr9	103544530	103558001	13472	-0.87015	0.54709	1.21E-278	Loss	Gain
Chr9_CNVR_13	Chr9	103614416	103622835	8420	-0.8727423	0.546108	7.91E-176	Loss	Gain
Chr9_CNVR_14	Chr9	103623678	103637991	14314	-0.9148877	0.530385	0	Loss	Gain
Chr9_CNVR_15	Chr9	103721350	103739031	17682	-0.9577819	0.514848	0	Loss	Gain
Chr9_CNVR_16	Chr9	103747452	103756713	9262	-0.8889754	0.539997	2.42E-199	Loss	Gain
Chr9_CNVR_17	Chr9	103936902	103950373	13472	-0.8718954	0.546428	1.34E-279	Loss	Gain
Chr9_CNVR_18	Chr9	103953742	103971423	17682	-0.9738691	0.509139	0	Loss	Gain
Chr9_CNVR_19	Chr9	104057308	104099407	42100	-0.9293687	0.525088	0	Loss	Gain
Chr9_CNVR_20	Chr9	104106986	104125509	18524	-0.9676093	0.511353	0	Loss	Gain
Chr9_CNVR_21	Chr9	104141508	104150769	9262	-0.901639	0.535278	3.65E-204	Loss	Gain
Chr9_CNVR_22	Chr9	104244232	104256861	12630	-0.8944198	0.537964	7.00E-274	Loss	Gain
Chr9_CNVR_23	Chr9	104340220	104350323	10104	-0.8326101	0.561512	2.87E-194	Loss	Gain
Chr9_CNVR_24	Chr9	104375584	104385687	10104	-0.8686882	0.547645	6.48E-209	Loss	Gain
Chr9_CNVR_25	Chr9	105238634	105247895	9262	-0.9287821	0.525302	1.36E-214	Loss	Gain
ChrX_CNVR_10	ChrX	39168644	39200522	31879	-1.197767	0.43595	0	Loss	Gain
ChrX_CNVR_11	ChrX	39209216	39233848	24633	-1.010648	0.496323	0	Loss	Gain
ChrX_CNVR_12	ChrX	39242544	39258482	15939	-1.020373	0.492989	5.94E-250	Loss	Gain
ChrX_CNVR_13	ChrX	39286014	39304850	18837	-1.822077	0.282814	0	Loss	Gain
ChrX_CNVR_14	ChrX	39309198	39325136	15939	-1.215155	0.430727	0	Loss	Gain
ChrX_CNVR_1	ChrX	43007046	43105576	98531	4.459977	22.0083	0	Gain	Loss
ChrX_CNVR_2	ChrX	44309696	44385044	75349	1.322158	2.5004	0	Gain	Loss
ChrX_CNVR_3	ChrX	48035076	48053912	18837	0.8536857	1.80711	3.33E-231	Gain	Loss
ChrX_CNVR_4	ChrX	94758080	94801550	43471	2.288026	4.88387	0	Gain	Loss
ChrX_CNVR_15	ChrX	95175392	95194228	18837	-1.096336	0.467703	0	Loss	Gain
ChrX_CNVR_5	ChrX	118705704	118753520	47817	1.112605	2.16236	0	Gain	Loss
ChrX_CNVR_6	ChrX	119065056	119088238	23183	1.804237	3.49244	0	Gain	Loss
ChrX_CNVR_7	ChrX	119137506	119163586	26081	1.209407	2.31243	0	Gain	Loss
ChrX_CNVR_8	ChrX	119218650	119234588	15939	1.838415	3.57617	0	Gain	Loss
ChrX_CNVR_9	ChrX	119498306	119546122	47817	1.168529	2.24782	0	Gain	Loss
ChrX_CNVR_16	ChrX	147140880	147155368	14489	-1.345295	0.393574	0	Loss	Gain
