# Supplementary material for: Whole-Genome Analyses of Korean Native and Holstein Cattle Breeds by Massively Parallel Sequencing
Source: PLoS One. 2014 Jul 3;9(7):e101127. doi: 10.1371/journal.pone.0101127 (PMC4081042; doi:10.1371/journal.pone.0101127)
Supplement: Table S2 — CNVRs detected from JJHvsHOL. (PDF) [file pone.0101127.s005.pdf]

Supplementary Table S2. CNVRs detected from JJHvsHOL

cnv_name	chr	start	end	size	log2	Ratio	pvalue	Jeju Heugu	Holstein
Chr1_CNVR_1	Chr1	12913071	12925837	12767	1.080364	2.11457	0	Gain	Loss
Chr1_CNVR_9	Chr1	12926589	12939355	12767	-2.872997	0.136503	0	Loss	Gain
Chr1_CNVR_10	Chr1	15060931	15068439	7509	-2.535501	0.17248	0	Loss	Gain
Chr1_CNVR_2	Chr1	24985395	24997411	12017	1.043944	2.06186	0	Gain	Loss
Chr1_CNVR_11	Chr1	26475379	26506921	31543	-1.058565	0.480109	0	Loss	Gain
Chr1_CNVR_12	Chr1	32135667	32143927	8261	-0.8536478	0.553384	2.75E-189	Loss	Gain
Chr1_CNVR_13	Chr1	42133729	42146495	12767	-1.376998	0.385019	0	Loss	Gain
Chr1_CNVR_14	Chr1	42154007	42161515	7509	-0.9686215	0.510994	1.40E-213	Loss	Gain
Chr1_CNVR_15	Chr1	42181043	42191555	10513	-0.886669	0.540861	1.30E-256	Loss	Gain
Chr1_CNVR_16	Chr1	42253139	42263651	10513	-0.9791619	0.507274	1.32E-303	Loss	Gain
Chr1_CNVR_3	Chr1	46184623	46192883	8261	1.998253	3.99516	0	Gain	Loss
Chr1_CNVR_4	Chr1	76009837	76018097	8261	1.105316	2.15146	8.43E-286	Gain	Loss
Chr1_CNVR_5	Chr1	93010975	93022239	11265	1.841483	3.58378	0	Gain	Loss
Chr1_CNVR_17	Chr1	97519979	97544761	24783	-1.05456	0.481444	0	Loss	Gain
Chr1_CNVR_6	Chr1	104071703	104082967	11265	2.571398	5.94385	0	Gain	Loss
Chr1_CNVR_7	Chr1	118624581	118635845	11265	0.990464	1.98682	0	Gain	Loss
Chr1_CNVR_18	Chr1	120969955	120986475	16521	-1.115061	0.461672	0	Loss	Gain
Chr1_CNVR_8	Chr1	124142179	124151189	9011	4.657252	25.2332	0	Gain	Loss
Chr1_CNVR_19	Chr1	134334751	134342259	7509	-0.8821252	0.542568	2.77E-182	Loss	Gain
Chr13_CNVR_1	Chr13	239650	263271	23622	2.405698	5.29892	0	Gain	Loss
Chr13_CNVR_2	Chr13	875920	885825	9906	1.199867	2.29718	0	Gain	Loss
Chr13_CNVR_3	Chr13	6369178	6376797	7620	3.856283	14.4829	0	Gain	Loss
Chr13_CNVR_4	Chr13	10997566	11007471	9906	1.400666	2.64023	0	Gain	Loss
Chr13_CNVR_5	Chr13	11010520	11034903	24384	1.01961	2.02737	0	Gain	Loss
Chr13_CNVR_6	Chr13	11066146	11081385	15240	1.113259	2.16334	0	Gain	Loss
Chr13_CNVR_7	Chr13	11092054	11101197	9144	1.526158	2.88018	0	Gain	Loss
Chr13_CNVR_8	Chr13	11106532	11117961	11430	1.109101	2.15711	0	Gain	Loss
Chr13_CNVR_9	Chr13	11119486	11137773	18288	1.047088	2.06635	0	Gain	Loss
Chr13_CNVR_10	Chr13	11340466	11364087	23622	1.121457	2.17567	0	Gain	Loss
Chr13_CNVR_11	Chr13	11372470	11384661	12192	0.9564194	1.94049	0	Gain	Loss
Chr13_CNVR_12	Chr13	11438764	11450193	11430	1.06985	2.09922	0	Gain	Loss
Chr13_CNVR_13	Chr13	11483722	11502009	18288	0.9600781	1.94542	0	Gain	Loss
Chr13_CNVR_14	Chr13	17829658	17844135	14478	0.99626	1.99482	0	Gain	Loss
Chr13_CNVR_15	Chr13	17844898	17863185	18288	1.026274	2.03676	0	Gain	Loss
Chr13_CNVR_16	Chr13	53856256	53863875	7620	0.9811437	1.97403	7.39E-220	Gain	Loss
Chr13_CNVR_17	Chr13	53934742	53962935	28194	-1.060212	0.479562	0	Loss	Gain
Chr13_CNVR_18	Chr13	53963698	53977413	13716	-0.9287517	0.525313	0	Loss	Gain
Chr13_CNVR_19	Chr13	63029212	63065787	36576	-1.444966	0.367301	0	Loss	Gain
Chr14_CNVR_7	Chr14	2731688	2741796	10109	-1.446607	0.366883	0	Loss	Gain
Chr14_CNVR_8	Chr14	14495956	14507508	11553	-1.083561	0.471863	0	Loss	Gain
Chr14_CNVR_9	Chr14	14534944	14545052	10109	-0.9789239	0.507358	4.03E-298	Loss	Gain
Chr14_CNVR_10	Chr14	14545774	14557326	11553	-1.031078	0.489344	0	Loss	Gain
Chr14_CNVR_11	Chr14	14563824	14573932	10109	-0.9161013	0.529939	9.27E-267	Loss	Gain
Chr14_CNVR_12	Chr14	14574654	14599202	24549	-1.141677	0.453232	0	Loss	Gain
Chr14_CNVR_13	Chr14	14600646	14619418	18773	-2.237362	0.212074	0	Loss	Gain
Chr14_CNVR_14	Chr14	14620140	14641078	20939	-0.9709451	0.510172	0	Loss	Gain
Chr14_CNVR_15	Chr14	14649020	14677900	28881	-1.24021	0.423311	0	Loss	Gain
Chr14_CNVR_16	Chr14	14678622	14690174	11553	-1.06416	0.478251	0	Loss	Gain
Chr14_CNVR_17	Chr14	14695228	14711112	15885	-1.197863	0.435921	0	Loss	Gain
Chr14_CNVR_18	Chr14	14724830	14747934	23105	-1.072533	0.475483	0	Loss	Gain
Chr14_CNVR_19	Chr14	14750822	14764540	13719	-1.100218	0.466446	0	Loss	Gain
Chr14_CNVR_20	Chr14	14765984	14801362	35379	-1.060632	0.479422	0	Loss	Gain

Chr14_CNVR_21	Chr14	14804250	14845404	41155	-1.054382	0.481503	0	Loss	Gain
Chr14_CNVR_22	Chr14	14846126	14860566	14441	-1.136499	0.454862	0	Loss	Gain
Chr14_CNVR_23	Chr14	14870674	14887280	16607	-1.555593	0.340189	0	Loss	Gain
Chr14_CNVR_24	Chr14	14890890	14942152	51263	-1.969719	0.255303	0	Loss	Gain
Chr14_CNVR_25	Chr14	15014352	15030958	16607	-1.278072	0.412346	0	Loss	Gain
Chr14_CNVR_26	Chr14	15040344	15049008	8665	-1.521674	0.348282	0	Loss	Gain
Chr14_CNVR_27	Chr14	15079332	15114710	35379	-1.291264	0.408593	0	Loss	Gain
Chr14_CNVR_28	Chr14	15116876	15186188	69313	-1.20558	0.433595	0	Loss	Gain
Chr14_CNVR_29	Chr14	15290156	15308206	18051	-1.080451	0.472881	0	Loss	Gain
Chr14_CNVR_30	Chr14	15308928	15381850	72923	-1.310389	0.403212	0	Loss	Gain
Chr14_CNVR_1	Chr14	32270152	32293256	23105	2.415641	5.33556	0	Gain	Loss
Chr14_CNVR_31	Chr14	32536570	32545234	8665	-4.804485	0.0357854	0	Loss	Gain
Chr14_CNVR_2	Chr14	32548844	32556064	7221	0.9689077	1.95736	2.34E-212	Gain	Loss
Chr14_CNVR_3	Chr14	32558230	32566894	8665	0.9931287	1.9905	4.43E-265	Gain	Loss
Chr14_CNVR_4	Chr14	44859666	44866886	7221	1.711766	3.27562	0	Gain	Loss
Chr14_CNVR_5	Chr14	49642194	49655190	12997	0.9808351	1.97361	0	Gain	Loss
Chr14_CNVR_6	Chr14	49657356	49667464	10109	0.9752733	1.96601	1.04E-299	Gain	Loss
Chr14_CNVR_32	Chr14	54821100	54828320	7221	-0.9409195	0.520901	6.24E-200	Loss	Gain
Chr15_CNVR_1	Chr15	11375395	11390670	15276	1.144337	2.21045	0	Gain	Loss
Chr15_CNVR_22	Chr15	17743879	17755938	12060	-4.65433	0.0397107	0	Loss	Gain
Chr15_CNVR_2	Chr15	31857295	31865334	8040	3.668969	12.7195	0	Gain	Loss
Chr15_CNVR_3	Chr15	46480447	46488486	8040	1.123909	2.17937	4.42E-268	Gain	Loss
Chr15_CNVR_23	Chr15	46553611	46564062	10452	-3.39043	0.0953628	0	Loss	Gain
Chr15_CNVR_24	Chr15	46597831	46610694	12864	-1.106843	0.464309	0	Loss	Gain
Chr15_CNVR_25	Chr15	46611499	46619538	8040	-1.206148	0.433424	7.82E-299	Loss	Gain
Chr15_CNVR_26	Chr15	46623559	46632402	8844	-0.9217167	0.527881	1.10E-212	Loss	Gain
Chr15_CNVR_4	Chr15	46827775	46837422	9648	1.00611	2.00849	1.19E-268	Gain	Loss
Chr15_CNVR_5	Chr15	46878427	46889682	11256	0.9611358	1.94684	3.67E-290	Gain	Loss
Chr15_CNVR_6	Chr15	46896115	46908174	12060	1.111922	2.16133	0	Gain	Loss
Chr15_CNVR_27	Chr15	46944355	46952394	8040	-3.980873	0.0633341	0	Loss	Gain
Chr15_CNVR_7	Chr15	49091035	49110330	19296	1.025985	2.03635	0	Gain	Loss
Chr15_CNVR_8	Chr15	49115155	49128822	13668	0.9879582	1.98338	0	Gain	Loss
Chr15_CNVR_28	Chr15	49569415	49579062	9648	-1.049194	0.483238	2.25E-287	Loss	Gain
Chr15_CNVR_29	Chr15	49871719	49880562	8844	-0.8625846	0.549966	5.96E-190	Loss	Gain
Chr15_CNVR_30	Chr15	49912723	49921566	8844	-0.8431652	0.557419	1.23E-182	Loss	Gain
Chr15_CNVR_31	Chr15	49944883	49956138	11256	-1.034818	0.488077	0	Loss	Gain
Chr15_CNVR_32	Chr15	50719939	50754510	34572	-4.933456	0.0327252	0	Loss	Gain
Chr15_CNVR_33	Chr15	50787475	50808378	20904	-2.042892	0.242677	0	Loss	Gain
Chr15_CNVR_9	Chr15	50814811	50827674	12864	1.036213	2.05084	0	Gain	Loss
Chr15_CNVR_34	Chr15	50831695	50844558	12864	-2.467536	0.1808	0	Loss	Gain
Chr15_CNVR_10	Chr15	51084955	51105054	20100	3.362279	10.2836	0	Gain	Loss
Chr15_CNVR_35	Chr15	51730567	51745842	15276	-0.9722381	0.509715	0	Loss	Gain
Chr15_CNVR_36	Chr15	52218595	52227438	8844	-1.152282	0.449913	4.62E-306	Loss	Gain
Chr15_CNVR_37	Chr15	52229047	52241910	12864	-1.147683	0.45135	0	Loss	Gain
Chr15_CNVR_38	Chr15	52263619	52282914	19296	-2.67764	0.156297	0	Loss	Gain
Chr15_CNVR_11	Chr15	64543915	64564818	20904	1.181043	2.26741	0	Gain	Loss
Chr15_CNVR_12	Chr15	69969307	69977346	8040	1.437695	2.70888	0	Gain	Loss
Chr15_CNVR_39	Chr15	78331711	78341358	9648	-3.861302	0.0688069	0	Loss	Gain
Chr15_CNVR_13	Chr15	79531279	79542534	11256	3.113712	8.65607	0	Gain	Loss
Chr15_CNVR_14	Chr15	79606051	79618110	12060	1.685117	3.21566	0	Gain	Loss
Chr15_CNVR_15	Chr15	79815895	79833582	17688	1.066921	2.09496	0	Gain	Loss
Chr15_CNVR_40	Chr15	80789539	80799186	9648	-0.9366617	0.52244	3.31E-238	Loss	Gain
Chr15_CNVR_16	Chr15	80812051	80842602	30552	1.227219	2.34115	0	Gain	Loss
Chr15_CNVR_17	Chr15	80996167	81028326	32160	1.270793	2.41294	0	Gain	Loss
Chr15_CNVR_18	Chr15	81036367	81054054	17688	0.9863841	1.98121	0	Gain	Loss
Chr15_CNVR_19	Chr15	81054859	81075762	20904	1.043392	2.06107	0	Gain	Loss

Chr15_CNVR_20	Chr15	81078175	81086214	8040	1.11673	2.16855	2.23E-265	Gain	Loss
Chr15_CNVR_21	Chr15	81300079	81308922	8844	1.050828	2.07172	1.37E-264	Gain	Loss
Chr15_CNVR_41	Chr15	81870115	81907902	37788	-2.438196	0.184514	0	Loss	Gain
Chr15_CNVR_42	Chr15	81908707	81928002	19296	-1.45873	0.363813	0	Loss	Gain
Chr15_CNVR_43	Chr15	83368771	83377614	8844	-3.015878	0.123632	0	Loss	Gain
Chr16_CNVR_1	Chr16	3502	16727	13226	1.79793	3.47721	0	Gain	Loss
Chr16_CNVR_8	Chr16	5584874	5592653	7780	-0.8375179	0.559606	3.18E-164	Loss	Gain
Chr16_CNVR_9	Chr16	5620662	5632331	11670	-1.020597	0.492912	0	Loss	Gain
Chr16_CNVR_10	Chr16	5633110	5643223	10114	-1.054761	0.481377	0	Loss	Gain
Chr16_CNVR_11	Chr16	5644780	5652559	7780	-0.9511125	0.517233	6.16E-204	Loss	Gain
Chr16_CNVR_12	Chr16	5681346	5689903	8558	-0.8276725	0.563438	8.87E-177	Loss	Gain
Chr16_CNVR_13	Chr16	5702352	5724135	21784	-0.9868274	0.504586	0	Loss	Gain
Chr16_CNVR_14	Chr16	5728804	5738917	10114	-0.928343	0.525462	6.50E-254	Loss	Gain
Chr16_CNVR_15	Chr16	5739696	5761479	21784	-1.077629	0.473807	0	Loss	Gain
Chr16_CNVR_16	Chr16	5762258	5773149	10892	-0.9210069	0.52814	1.23E-269	Loss	Gain
Chr16_CNVR_2	Chr16	7408506	7419397	10892	0.8977255	1.86313	2.54E-258	Gain	Loss
Chr16_CNVR_3	Chr16	8578618	8587175	8558	1.118221	2.17079	4.79E-292	Gain	Loss
Chr16_CNVR_4	Chr16	8601180	8638523	37344	1.065145	2.09238	0	Gain	Loss
Chr16_CNVR_5	Chr16	11524904	11533461	8558	3.986269	15.8484	0	Gain	Loss
Chr16_CNVR_6	Chr16	39448880	39476887	28008	3.098251	8.5638	0	Gain	Loss
Chr16_CNVR_7	Chr16	81517674	81526231	8558	1.069123	2.09816	7.89E-272	Gain	Loss
Chr17_CNVR_1	Chr17	15322753	15331969	9217	2.416955	5.34043	0	Gain	Loss
Chr17_CNVR_2	Chr17	21083521	21096577	13057	5.45096	43.7424	0	Gain	Loss
Chr17_CNVR_3	Chr17	21913729	21921409	7681	1.196942	2.29253	2.58E-295	Gain	Loss
Chr17_CNVR_4	Chr17	28278913	28287361	8449	1.646983	3.13178	0	Gain	Loss
Chr17_CNVR_5	Chr17	52439425	52450177	10753	5.490304	44.9517	0	Gain	Loss
Chr17_CNVR_6	Chr17	52468609	52480129	11521	3.013605	8.0758	0	Gain	Loss
Chr17_CNVR_7	Chr17	70111873	70120321	8449	1.021808	2.03046	1.96E-252	Gain	Loss
Chr17_CNVR_8	Chr17	72879745	72888961	9217	-0.9730686	0.509421	1.27E-253	Loss	Gain
Chr17_CNVR_9	Chr17	72895873	72906625	10753	-1.156036	0.448744	0	Loss	Gain
Chr17_CNVR_10	Chr17	72911233	72925057	13825	-0.8610917	0.550536	0	Loss	Gain
Chr17_CNVR_11	Chr17	72925825	72938881	13057	-2.44287	0.183917	0	Loss	Gain
Chr17_CNVR_12	Chr17	75136129	75146881	10753	-2.077406	0.23694	0	Loss	Gain
Chr18_CNVR_1	Chr18	669145	678552	9408	1.358974	2.56503	0	Gain	Loss
Chr18_CNVR_8	Chr18	57636153	57650264	14112	-2.007736	0.248663	0	Loss	Gain
Chr18_CNVR_2	Chr18	58473465	58486008	12544	1.056953	2.08053	0	Gain	Loss
Chr18_CNVR_3	Chr18	58569897	58580872	10976	1.196516	2.29186	0	Gain	Loss
Chr18_CNVR_4	Chr18	61447961	61463640	15680	5.820073	56.4959	0	Gain	Loss
Chr18_CNVR_9	Chr18	61661209	61679240	18032	-3.764001	0.0736076	0	Loss	Gain
Chr18_CNVR_5	Chr18	61680025	61689432	9408	0.9947895	1.99279	2.06E-271	Gain	Loss
Chr18_CNVR_6	Chr18	61757641	61770968	13328	3.495898	11.2816	0	Gain	Loss
Chr18_CNVR_7	Chr18	63207257	63216664	9408	1.925407	3.79844	0	Gain	Loss
Chr18_CNVR_10	Chr18	63255865	63266056	10192	-1.384692	0.382971	0	Loss	Gain
Chr18_CNVR_11	Chr18	63317017	63327208	10192	-1.937784	0.261017	0	Loss	Gain
Chr18_CNVR_12	Chr18	63527129	63571816	44688	-1.462397	0.36289	0	Loss	Gain
Chr18_CNVR_13	Chr18	63791337	63805448	14112	-1.133148	0.45592	0	Loss	Gain
Chr19_CNVR_1	Chr19	1867363	1875113	7751	1.187083	2.27692	2.34E-300	Gain	Loss
Chr19_CNVR_2	Chr19	19796213	19851237	55025	1.638088	3.11253	0	Gain	Loss
Chr19_CNVR_3	Chr19	19857439	19899287	41849	2.179538	4.53008	0	Gain	Loss
Chr19_CNVR_4	Chr19	19902389	19952763	50375	1.616617	3.06655	0	Gain	Loss
Chr19_CNVR_8	Chr19	24206739	24215263	8525	-1.299268	0.406332	0	Loss	Gain
Chr19_CNVR_5	Chr19	24621363	24633763	12401	1.008119	2.01129	0	Gain	Loss
Chr19_CNVR_6	Chr19	51058939	51067463	8525	1.062842	2.08904	7.20E-277	Gain	Loss
Chr19_CNVR_7	Chr19	57488339	57499187	10849	0.9214919	1.89407	3.16E-277	Gain	Loss
Chr2_CNVR_1	Chr2	57407997	57416884	8888	1.097461	2.13978	1.54E-282	Gain	Loss
Chr2_CNVR_2	Chr2	68404877	68416996	12120	3.232497	9.39893	0	Gain	Loss

Chr2_CNVR_3	Chr2	68451741	68460628	8888	2.526267	5.76079	0	Gain	Loss
Chr2_CNVR_11	Chr2	82047149	82055228	8080	-0.9085928	0.532704	1.62E-191	Loss	Gain
Chr2_CNVR_12	Chr2	83461149	83471652	10504	-4.140604	0.0566962	0	Loss	Gain
Chr2_CNVR_4	Chr2	124181925	124208588	26664	0.91149	1.88099	0	Gain	Loss
Chr2_CNVR_5	Chr2	124329789	124342716	12928	0.9365767	1.91398	0	Gain	Loss
Chr2_CNVR_6	Chr2	124343525	124355644	12120	1.030866	2.04325	0	Gain	Loss
Chr2_CNVR_7	Chr2	124363725	124373420	9696	0.9710943	1.96033	1.78E-252	Gain	Loss
Chr2_CNVR_8	Chr2	124389581	124400084	10504	0.9391009	1.91733	1.80E-258	Gain	Loss
Chr2_CNVR_13	Chr2	133284853	133296164	11312	-1.174817	0.44294	0	Loss	Gain
Chr2_CNVR_14	Chr2	136971757	136983068	11312	-2.100297	0.23321	0	Loss	Gain
Chr2_CNVR_9	Chr2	137001653	137016196	14544	3.056325	8.31851	0	Gain	Loss
Chr2_CNVR_15	Chr2	137031549	137042860	11312	-1.394807	0.380296	0	Loss	Gain
Chr2_CNVR_10	Chr2	137043669	137057404	13736	2.643075	6.24662	0	Gain	Loss
Chr20_CNVR_1	Chr20	72030206	72038265	8060	1.048121	2.06783	3.78E-239	Gain	Loss
Chr21_CNVR_3	Chr21	952106	969815	17710	-1.455812	0.36455	0	Loss	Gain
Chr21_CNVR_4	Chr21	1605836	1626625	20790	-2.267316	0.207716	0	Loss	Gain
Chr21_CNVR_5	Chr21	1627396	1638175	10780	-1.875796	0.272477	0	Loss	Gain
Chr21_CNVR_6	Chr21	1771386	1779855	8470	-2.179627	0.220733	0	Loss	Gain
Chr21_CNVR_7	Chr21	9609216	9641555	32340	-1.73975	0.299422	0	Loss	Gain
Chr21_CNVR_8	Chr21	20087376	20108935	21560	-2.70831	0.153009	0	Loss	Gain
Chr21_CNVR_9	Chr21	20115096	20142045	26950	-2.461191	0.181597	0	Loss	Gain
Chr21_CNVR_10	Chr21	20160526	20177465	16940	-1.056557	0.480778	0	Loss	Gain
Chr21_CNVR_11	Chr21	20327616	20337625	10010	-3.280783	0.102893	0	Loss	Gain
Chr21_CNVR_12	Chr21	52304176	52333435	29260	-6.649154	0.00996335	0	Loss	Gain
Chr21_CNVR_1	Chr21	54928336	54939115	10780	1.09573	2.13721	0	Gain	Loss
Chr21_CNVR_13	Chr21	62490506	62498975	8470	-1.07286	0.475376	9.43E-273	Loss	Gain
Chr21_CNVR_2	Chr21	70583206	70591675	8470	0.9508924	1.93307	2.99E-226	Gain	Loss
Chr23_CNVR_6	Chr23	7086731	7101911	15181	-1.138929	0.454097	0	Loss	Gain
Chr23_CNVR_1	Chr23	25332695	25346277	13583	4.326091	20.0578	0	Gain	Loss
Chr23_CNVR_2	Chr23	25348675	25362257	13583	5.374742	41.4914	0	Gain	Loss
Chr23_CNVR_7	Chr23	25424581	25442957	18377	-3.753756	0.0741322	0	Loss	Gain
Chr23_CNVR_3	Chr23	25458937	25466927	7991	2.933755	7.64097	0	Gain	Loss
Chr23_CNVR_8	Chr23	25705029	25713817	8789	-1.224542	0.427933	0	Loss	Gain
Chr23_CNVR_9	Chr23	25748975	25758561	9587	-3.351351	0.0979812	0	Loss	Gain
Chr23_CNVR_10	Chr23	25759361	25770547	11187	-2.84822	0.138867	0	Loss	Gain
Chr23_CNVR_11	Chr23	25780135	25816089	35955	-3.667312	0.0787098	0	Loss	Gain
Chr23_CNVR_12	Chr23	25824879	25838461	13583	-1.646455	0.319424	0	Loss	Gain
Chr23_CNVR_13	Chr23	25844855	25857637	12783	-3.466676	0.0904537	0	Loss	Gain
Chr23_CNVR_14	Chr23	25888799	25910371	21573	-1.127869	0.457591	0	Loss	Gain
Chr23_CNVR_15	Chr23	26005453	26027025	21573	-1.655067	0.317523	0	Loss	Gain
Chr23_CNVR_16	Chr23	26285903	26301881	15979	-3.331025	0.0993714	0	Loss	Gain
Chr23_CNVR_17	Chr23	26319461	26348223	28763	-3.495365	0.0886728	0	Loss	Gain
Chr23_CNVR_18	Chr23	26535191	26550371	15181	-1.225058	0.42778	0	Loss	Gain
Chr23_CNVR_19	Chr23	27737685	27756061	18377	-1.725542	0.302385	0	Loss	Gain
Chr23_CNVR_20	Chr23	27857535	27870319	12785	-2.42798	0.185825	0	Loss	Gain
Chr23_CNVR_21	Chr23	28820331	28833913	13583	-2.950463	0.129367	0	Loss	Gain
Chr23_CNVR_22	Chr23	28835511	28850691	15181	-3.478106	0.0897399	0	Loss	Gain
Chr23_CNVR_4	Chr23	29007297	29022477	15181	0.9268325	1.9011	0	Gain	Loss
Chr23_CNVR_23	Chr23	29702427	29735983	33557	-1.367512	0.387559	0	Loss	Gain
Chr23_CNVR_24	Chr23	29736783	29750365	13583	-1.669645	0.314331	0	Loss	Gain
Chr23_CNVR_5	Chr23	29782327	29812687	30361	4.32654	20.064	0	Gain	Loss
Chr24_CNVR_3	Chr24	23602713	23611337	8625	-2.039339	0.243275	0	Loss	Gain
Chr24_CNVR_1	Chr24	44178009	44194473	16465	3.486174	11.2058	0	Gain	Loss
Chr24_CNVR_2	Chr24	62404441	62427961	23521	1.479738	2.78898	0	Gain	Loss
Chr26_CNVR_1	Chr26	1941577	1967448	25872	-4.102209	0.0582253	0	Loss	Gain
Chr26_CNVR_2	Chr26	31584617	31598728	14112	-3.391722	0.0952774	0	Loss	Gain

Chr28_CNVR_5	Chr28	1805282	1812652	7371	-4.102082	0.0582305	0	Loss	Gain
Chr28_CNVR_1	Chr28	2254116	2271066	16951	3.786404	13.7982	0	Gain	Loss
Chr28_CNVR_2	Chr28	3150308	3158414	8107	2.441519	5.43213	0	Gain	Loss
Chr28_CNVR_3	Chr28	6792562	6799930	7369	0.8072882	1.74992	3.96E-154	Gain	Loss
Chr28_CNVR_4	Chr28	11353118	11361224	8107	1.452258	2.73636	0	Gain	Loss
Chr29_CNVR_10	Chr29	5550931	5568751	17821	-0.9169982	0.52961	0	Loss	Gain
Chr29_CNVR_11	Chr29	8559211	8571091	11881	-0.9144123	0.53056	0	Loss	Gain
Chr29_CNVR_12	Chr29	21858871	21865471	6601	-0.9011787	0.535449	7.51E-186	Loss	Gain
Chr29_CNVR_13	Chr29	21866791	21874051	7261	-0.822298	0.56554	1.45E-174	Loss	Gain
Chr29_CNVR_1	Chr29	22119571	22131451	11881	1.076897	2.10949	0	Gain	Loss
Chr29_CNVR_2	Chr29	23940511	23954371	13861	1.002909	2.00404	0	Gain	Loss
Chr29_CNVR_3	Chr29	26954731	26966611	11881	3.596618	12.0973	0	Gain	Loss
Chr29_CNVR_14	Chr29	27168571	27175171	6601	-5.256817	0.0261541	0	Loss	Gain
Chr29_CNVR_15	Chr29	27362611	27382411	19801	-3.426376	0.0930161	0	Loss	Gain
Chr29_CNVR_16	Chr29	27390331	27414091	23761	-5.063344	0.0299076	0	Loss	Gain
Chr29_CNVR_17	Chr29	27639151	27647731	8581	-0.9261466	0.526262	2.31E-252	Loss	Gain
Chr29_CNVR_18	Chr29	27649051	27659611	10561	-0.9429503	0.520168	0	Loss	Gain
Chr29_CNVR_19	Chr29	27734851	27744091	9241	-1.106756	0.464337	0	Loss	Gain
Chr29_CNVR_4	Chr29	27762571	27769171	6601	2.761281	6.77998	0	Gain	Loss
Chr29_CNVR_5	Chr29	28026571	28039771	13201	1.747951	3.35881	0	Gain	Loss
Chr29_CNVR_20	Chr29	49910851	49918111	7261	-0.9118571	0.5315	2.46E-208	Loss	Gain
Chr29_CNVR_6	Chr29	50246131	50253391	7261	0.9368792	1.91438	2.25E-222	Gain	Loss
Chr29_CNVR_7	Chr29	50352391	50359651	7261	1.043549	2.06129	4.90E-266	Gain	Loss
Chr29_CNVR_8	Chr29	50881711	50888311	6601	0.8066453	1.74914	2.25E-156	Gain	Loss
Chr29_CNVR_9	Chr29	51401131	51407731	6601	0.8912318	1.85476	7.18E-186	Gain	Loss
Chr3_CNVR_1	Chr3	8518304	8538986	20683	6.586098	96.0756	0	Gain	Loss
Chr3_CNVR_2	Chr3	8611756	8669972	58217	4.626146	24.695	0	Gain	Loss
Chr3_CNVR_3	Chr3	8732018	8751934	19917	6.697408	103.782	0	Gain	Loss
Chr3_CNVR_8	Chr3	47039678	47048104	8427	-8.469147	0.00282184	0	Loss	Gain
Chr3_CNVR_4	Chr3	54766320	54799258	32939	2.327264	5.01853	0	Gain	Loss
Chr3_CNVR_5	Chr3	62696718	62711272	14555	1.05596	2.0791	0	Gain	Loss
Chr3_CNVR_9	Chr3	91037952	91047144	9193	-1.165513	0.445806	0	Loss	Gain
Chr3_CNVR_6	Chr3	105709916	105725236	15321	0.9799279	1.97237	0	Gain	Loss
Chr3_CNVR_7	Chr3	119799720	119809678	9959	1.072006	2.10235	0	Gain	Loss
Chr4_CNVR_1	Chr4	1901	23941	22041	1.678213	3.20031	0	Gain	Loss
Chr4_CNVR_2	Chr4	24701	49781	25081	1.520857	2.86961	0	Gain	Loss
Chr4_CNVR_3	Chr4	54341	83221	28881	1.281727	2.4313	0	Gain	Loss
Chr4_CNVR_4	Chr4	84741	229141	144401	1.228818	2.34375	0	Gain	Loss
Chr4_CNVR_5	Chr4	2846581	2854181	7601	1.156496	2.22915	1.12E-278	Gain	Loss
Chr4_CNVR_9	Chr4	25537901	25546261	8361	-1.813233	0.284553	0	Loss	Gain
Chr4_CNVR_6	Chr4	28241981	28254141	12161	4.238072	18.8706	0	Gain	Loss
Chr4_CNVR_10	Chr4	44618461	44628341	9881	-1.058443	0.48015	0	Loss	Gain
Chr4_CNVR_11	Chr4	86733861	86745261	11401	-4.795322	0.0360134	0	Loss	Gain
Chr4_CNVR_12	Chr4	95976221	95983821	7601	-3.312301	0.10067	0	Loss	Gain
Chr4_CNVR_7	Chr4	99615861	99623461	7601	1.908553	3.75432	0	Gain	Loss
Chr4_CNVR_13	Chr4	106640541	106651941	11401	-1.213927	0.431094	0	Loss	Gain
Chr4_CNVR_14	Chr4	106690701	106705901	15201	-1.298412	0.406573	0	Loss	Gain
Chr4_CNVR_15	Chr4	106768221	106778861	10641	-2.316769	0.200716	0	Loss	Gain
Chr4_CNVR_16	Chr4	106861701	106869301	7601	-1.375021	0.385547	0	Loss	Gain
Chr4_CNVR_8	Chr4	109596181	109605301	9121	1.130522	2.18938	0	Gain	Loss
Chr4_CNVR_17	Chr4	119712541	119721661	9121	-3.408245	0.0941924	0	Loss	Gain
ChrX_CNVR_7	ChrX	34742030	34771378	29349	-1.483807	0.357544	0	Loss	Gain
ChrX_CNVR_1	ChrX	43007494	43104876	97383	4.55209	23.4593	0	Gain	Loss
ChrX_CNVR_2	ChrX	44310812	44384182	73371	1.154507	2.22608	0	Gain	Loss
ChrX_CNVR_8	ChrX	53878260	53898270	20011	-2.889551	0.134946	0	Loss	Gain
ChrX_CNVR_9	ChrX	54062352	54127718	65367	-1.184129	0.44009	0	Loss	Gain

ChrX_CNVR_3	ChrX	64062016	64078024	16009	0.9991386	1.99881	1.42E-264	Gain	Loss
ChrX_CNVR_4	ChrX	64163400	64176740	13341	0.8389113	1.7887	1.66E-164	Gain	Loss
ChrX_CNVR_10	ChrX	82480554	82499230	18677	-2.732634	0.150451	0	Loss	Gain
ChrX_CNVR_11	ChrX	92759024	92777700	18677	-1.029417	0.489908	0	Loss	Gain
ChrX_CNVR_5	ChrX	94758690	94801378	42689	2.563264	5.91043	0	Gain	Loss
ChrX_CNVR_12	ChrX	95176232	95194908	18677	-0.9378767	0.522001	3.45E-282	Loss	Gain
ChrX_CNVR_6	ChrX	118681312	118694652	13341	1.090867	2.13002	1.55E-254	Gain	Loss
ChrX_CNVR_13	ChrX	147142202	147155542	13341	-1.238529	0.423805	0	Loss	Gain
