# Supplementary material for: Whole-Genome Analyses of Korean Native and Holstein Cattle Breeds by Massively Parallel Sequencing
Source: PLoS One. 2014 Jul 3;9(7):e101127. doi: 10.1371/journal.pone.0101127 (PMC4081042; doi:10.1371/journal.pone.0101127)
Supplement: Table S3 — CNVRs detected from CHSvsHOL. (PDF) [file pone.0101127.s006.pdf]

Supplementary Table S3. CNVRs detected from CHSvsHOL

cnv_name	chr	start	end	size	log2	Ratio	pvalue	Chikso	Holstein
Chr1_CNVR_16	Chr1	2404591	2412581	7991	-0.8912844	0.539134	4.01E-182	Loss	Gain
Chr1_CNVR_17	Chr1	2414179	2432555	18377	-0.8198837	0.566488	0	Loss	Gain
Chr1_CNVR_18	Chr1	5237047	5246633	9587	-1.214756	0.430846	0	Loss	Gain
Chr1_CNVR_19	Chr1	5249031	5257021	7991	-1.133545	0.455794	2.55E-269	Loss	Gain
Chr1_CNVR_20	Chr1	5275399	5288181	12783	-1.045921	0.484336	0	Loss	Gain
Chr1_CNVR_21	Chr1	6314897	6322887	7991	-0.8893908	0.539842	1.80E-181	Loss	Gain
Chr1_CNVR_1	Chr1	12913039	12925823	12785	1.199741	2.29698	0	Gain	Loss
Chr1_CNVR_22	Chr1	12926623	12939405	12783	-2.79276	0.14431	0	Loss	Gain
Chr1_CNVR_2	Chr1	24985929	24997913	11985	1.054119	2.07645	0	Gain	Loss
Chr1_CNVR_3	Chr1	32403047	32411035	7989	0.873487	1.83209	4.35E-183	Gain	Loss
Chr1_CNVR_4	Chr1	40436193	40445779	9587	0.8453385	1.79669	3.83E-207	Gain	Loss
Chr1_CNVR_5	Chr1	40474545	40483333	8789	0.9745514	1.96503	1.64E-242	Gain	Loss
Chr1_CNVR_6	Chr1	40506505	40517689	11185	0.8792383	1.8394	2.10E-258	Gain	Loss
Chr1_CNVR_7	Chr1	40524881	40536067	11187	0.9193762	1.8913	4.76E-279	Gain	Loss
Chr1_CNVR_8	Chr1	40581611	40593595	11985	1.10928	2.15738	0	Gain	Loss
Chr1_CNVR_9	Chr1	42273893	42281881	7989	1.099276	2.14247	5.15E-269	Gain	Loss
Chr1_CNVR_23	Chr1	57474069	57483655	9587	-0.957673	0.514887	5.12E-246	Loss	Gain
Chr1_CNVR_24	Chr1	57647451	57659435	11985	-1.119635	0.46021	0	Loss	Gain
Chr1_CNVR_25	Chr1	68314101	68325287	11187	-0.9033481	0.534645	5.66E-260	Loss	Gain
Chr1_CNVR_26	Chr1	68343665	68359643	15979	-0.8677711	0.547993	0	Loss	Gain
Chr1_CNVR_27	Chr1	70006383	70014373	7991	-1.049355	0.483184	2.26E-238	Loss	Gain
Chr1_CNVR_10	Chr1	76010069	76018857	8789	1.208212	2.31051	0	Gain	Loss
Chr1_CNVR_28	Chr1	83146737	83158721	11985	-0.9109823	0.531823	2.28E-282	Loss	Gain
Chr1_CNVR_29	Chr1	83439971	83455151	15181	-0.8876318	0.540501	0	Loss	Gain
Chr1_CNVR_11	Chr1	88506429	88516017	9589	0.8655367	1.82202	7.74E-216	Gain	Loss
Chr1_CNVR_12	Chr1	92989619	93007195	17577	0.904218	1.87153	0	Gain	Loss
Chr1_CNVR_13	Chr1	93012789	93026371	13583	1.263202	2.40028	0	Gain	Loss
Chr1_CNVR_30	Chr1	93717507	93830965	113459	-5.493212	0.0222013	0	Loss	Gain
Chr1_CNVR_31	Chr1	97519949	97544717	24769	-3.483555	0.0894016	0	Loss	Gain
Chr1_CNVR_14	Chr1	104071749	104082933	11185	2.573177	5.95119	0	Gain	Loss
Chr1_CNVR_32	Chr1	111641475	111651861	10387	-0.896531	0.537177	1.73E-238	Loss	Gain
Chr1_CNVR_15	Chr1	118623935	118635919	11985	1.156044	2.22846	0	Gain	Loss
Chr1_CNVR_33	Chr1	128889487	128897477	7991	-0.8763933	0.544728	5.29E-177	Loss	Gain
Chr1_CNVR_34	Chr1	143506393	143514383	7991	-0.8006926	0.574074	1.34E-151	Loss	Gain
Chr1_CNVR_35	Chr1	143523173	143532759	9587	-0.9038	0.534477	2.51E-223	Loss	Gain
Chr1_CNVR_36	Chr1	143647017	143658203	11187	-0.8677336	0.548007	8.00E-243	Loss	Gain
Chr1_CNVR_37	Chr1	143839577	143853957	14381	-0.864625	0.549189	0	Loss	Gain
Chr1_CNVR_38	Chr1	144030537	144040923	10387	-0.8310464	0.562121	1.58E-209	Loss	Gain
Chr1_CNVR_39	Chr1	144422847	144431635	8789	-0.9686699	0.510977	5.42E-230	Loss	Gain
Chr1_CNVR_40	Chr1	144446017	144454007	7991	-0.9079452	0.532944	6.79E-188	Loss	Gain
Chr1_CNVR_41	Chr1	144467591	144475579	7989	-0.8086849	0.570902	3.15E-154	Loss	Gain
Chr1_CNVR_42	Chr1	144491561	144504343	12783	-0.8740252	0.545622	1.21E-280	Loss	Gain
Chr1_CNVR_43	Chr1	144514731	144524319	9589	-0.8816358	0.542752	3.81E-214	Loss	Gain
Chr1_CNVR_44	Chr1	144787191	144797577	10387	-0.9382373	0.52187	2.07E-257	Loss	Gain
Chr1_CNVR_45	Chr1	144928613	144939799	11187	-0.860793	0.55065	1.62E-239	Loss	Gain
Chr1_CNVR_46	Chr1	144972559	144982145	9587	-0.8627094	0.549919	2.20E-206	Loss	Gain
Chr1_CNVR_47	Chr1	145009313	145018101	8789	-1.004568	0.498419	3.87E-244	Loss	Gain
Chr1_CNVR_48	Chr1	145227439	145236227	8789	-1.019031	0.493448	6.99E-250	Loss	Gain
Chr1_CNVR_49	Chr1	145453557	145463143	9587	-0.8346671	0.560712	5.03E-195	Loss	Gain
Chr1_CNVR_50	Chr1	145472733	145490309	17577	-0.8929771	0.538502	0	Loss	Gain
Chr1_CNVR_51	Chr1	145593381	145604567	11187	-0.8654054	0.548892	1.03E-241	Loss	Gain
Chr1_CNVR_52	Chr1	145610161	145626939	16779	-0.8557484	0.552579	0	Loss	Gain

Chr1_CNVR_53	Chr1	145630935	145646115	15181	-0.954093	0.516166	0	Loss	Gain
Chr1_CNVR_54	Chr1	145646915	145656501	9587	-0.8310424	0.562123	1.43E-193	Loss	Gain
Chr1_CNVR_55	Chr1	145657301	145666889	9589	-0.8491554	0.55511	7.17E-201	Loss	Gain
Chr1_CNVR_56	Chr1	145678875	145694853	15979	-0.9925047	0.502604	0	Loss	Gain
Chr1_CNVR_57	Chr1	145698051	145709235	11185	-0.9334261	0.523613	1.12E-274	Loss	Gain
Chr1_CNVR_58	Chr1	145757177	145765165	7989	-0.9227436	0.527505	4.67E-193	Loss	Gain
Chr1_CNVR_59	Chr1	145766765	145780347	13583	-0.9270133	0.525946	0	Loss	Gain
Chr1_CNVR_60	Chr1	145783543	145791533	7991	-1.077409	0.473879	1.25E-248	Loss	Gain
Chr1_CNVR_61	Chr1	145792333	145802719	10387	-0.9289083	0.525256	3.78E-253	Loss	Gain
Chr1_CNVR_62	Chr1	145817901	145845065	27165	-0.930472	0.524687	0	Loss	Gain
Chr1_CNVR_63	Chr1	145853855	145862643	8789	-0.8923581	0.538733	1.55E-200	Loss	Gain
Chr1_CNVR_64	Chr1	146024841	146032831	7991	-0.8829867	0.542244	2.89E-179	Loss	Gain
Chr1_CNVR_65	Chr1	146041621	146051207	9587	-0.8541701	0.553183	6.62E-203	Loss	Gain
Chr1_CNVR_66	Chr1	146172657	146181445	8789	-0.8354356	0.560414	3.15E-179	Loss	Gain
Chr1_CNVR_67	Chr1	146260547	146270133	9587	-0.9082823	0.532819	3.40E-225	Loss	Gain
Chr1_CNVR_68	Chr1	146493055	146509833	16779	-0.9099926	0.532188	0	Loss	Gain
Chr1_CNVR_69	Chr1	146540995	146548985	7991	-0.9029167	0.534804	3.79E-186	Loss	Gain
Chr1_CNVR_70	Chr1	146572157	146581743	9587	-0.894119	0.538076	2.64E-219	Loss	Gain
Chr1_CNVR_71	Chr1	146588137	146606513	18377	-0.9346985	0.523152	0	Loss	Gain
Chr1_CNVR_72	Chr1	146627287	146638473	11187	-0.8655241	0.548847	9.06E-242	Loss	Gain
Chr1_CNVR_73	Chr1	146650459	146659247	8789	-0.7645483	0.588638	1.14E-153	Loss	Gain
Chr1_CNVR_74	Chr1	146683217	146696799	13583	-0.8635344	0.549604	5.77E-292	Loss	Gain
Chr1_CNVR_75	Chr1	146831033	146839821	8789	-0.8191956	0.566758	2.85E-173	Loss	Gain
Chr1_CNVR_76	Chr1	146853405	146861393	7989	-0.9195764	0.528664	5.99E-192	Loss	Gain
Chr1_CNVR_77	Chr1	146864591	146880569	15979	-0.9229275	0.527438	0	Loss	Gain
Chr1_CNVR_78	Chr1	146953279	146962067	8789	-0.943585	0.519939	3.29E-220	Loss	Gain
Chr1_CNVR_79	Chr1	146970059	146986037	15979	-0.9287916	0.525298	0	Loss	Gain
Chr1_CNVR_80	Chr1	146986837	146997223	10387	-0.891793	0.538944	2.33E-236	Loss	Gain
Chr1_CNVR_81	Chr1	147003617	147017997	14381	-0.9562752	0.515386	0	Loss	Gain
Chr1_CNVR_82	Chr1	147029185	147037173	7989	-1.015101	0.494794	6.15E-226	Loss	Gain
Chr1_CNVR_83	Chr1	147037973	147047561	9589	-0.9197924	0.528585	5.21E-230	Loss	Gain
Chr1_CNVR_84	Chr1	147050757	147059545	8789	-0.9559021	0.515519	5.31E-225	Loss	Gain
Chr1_CNVR_85	Chr1	147324015	147341593	17579	-0.8469772	0.555948	0	Loss	Gain
Chr1_CNVR_86	Chr1	147591681	147599669	7989	-0.821828	0.565725	1.40E-158	Loss	Gain
Chr1_CNVR_87	Chr1	154054791	154064379	9589	-0.8670213	0.548278	3.82E-208	Loss	Gain
Chr1_CNVR_88	Chr1	155073517	155088697	15181	-0.8427423	0.557583	0	Loss	Gain
Chr10_CNVR_26	Chr10	40735	49495	8761	-0.8420484	0.557851	5.65E-165	Loss	Gain
Chr10_CNVR_27	Chr10	71395	82783	11389	-0.949152	0.517937	1.78E-261	Loss	Gain
Chr10_CNVR_28	Chr10	20409487	20418247	8761	-0.8487664	0.555259	3.22E-167	Loss	Gain
Chr10_CNVR_29	Chr10	21345931	21355567	9637	-0.9501665	0.517573	5.76E-222	Loss	Gain
Chr10_CNVR_1	Chr10	22538167	22548679	10513	0.8714351	1.82948	1.76E-224	Gain	Loss
Chr10_CNVR_2	Chr10	22567951	22576711	8761	0.875389	1.8345	6.70E-189	Gain	Loss
Chr10_CNVR_3	Chr10	22580215	22608247	28033	1.195155	2.28969	0	Gain	Loss
Chr10_CNVR_4	Chr10	22620511	22645915	25405	1.159007	2.23304	0	Gain	Loss
Chr10_CNVR_5	Chr10	22715119	22738771	23653	2.311594	4.96431	0	Gain	Loss
Chr10_CNVR_6	Chr10	22766803	22777315	10513	0.866322	1.82301	3.49E-222	Gain	Loss
Chr10_CNVR_7	Chr10	22851775	22864915	13141	0.8311917	1.77915	1.03E-257	Gain	Loss
Chr10_CNVR_8	Chr10	22888567	22902583	14017	0.8533532	1.8067	8.31E-288	Gain	Loss
Chr10_CNVR_9	Chr10	22905211	22931491	26281	0.9732257	1.96323	0	Gain	Loss
Chr10_CNVR_10	Chr10	22942003	22952515	10513	1.062172	2.08807	0	Gain	Loss
Chr10_CNVR_11	Chr10	22959523	22968283	8761	0.9258542	1.89981	3.96E-208	Gain	Loss
Chr10_CNVR_12	Chr10	23055883	23064643	8761	0.8700124	1.82768	6.98E-187	Gain	Loss
Chr10_CNVR_13	Chr10	23529799	23543815	14017	0.93429	1.91095	0	Gain	Loss
Chr10_CNVR_14	Chr10	23582359	23634043	51685	1.439184	2.71167	0	Gain	Loss
Chr10_CNVR_15	Chr10	23669959	23683099	13141	4.353486	20.4423	0	Gain	Loss
Chr10_CNVR_16	Chr10	23686603	23701495	14893	2.958522	7.77327	0	Gain	Loss

Chr10_CNVR_30	Chr10	24075547	24109711	34165	-1.679103	0.312277	0	Loss	Gain
Chr10_CNVR_31	Chr10	24112339	24138619	26281	-2.759019	0.147724	0	Loss	Gain
Chr10_CNVR_32	Chr10	24154387	24164023	9637	-1.061456	0.479148	1.50E-265	Loss	Gain
Chr10_CNVR_33	Chr10	24168403	24185047	16645	-0.9471654	0.518651	0	Loss	Gain
Chr10_CNVR_34	Chr10	24192055	24206947	14893	-0.9011066	0.535476	0	Loss	Gain
Chr10_CNVR_35	Chr10	24230599	24239359	8761	-0.8765247	0.544678	1.42E-176	Loss	Gain
Chr10_CNVR_36	Chr10	24255127	24271771	16645	-0.9378067	0.522026	0	Loss	Gain
Chr10_CNVR_17	Chr10	24785107	24807007	21901	1.486987	2.80303	0	Gain	Loss
Chr10_CNVR_18	Chr10	24905995	24914755	8761	1.440201	2.71359	0	Gain	Loss
Chr10_CNVR_19	Chr10	25158283	25173175	14893	2.269064	4.8201	0	Gain	Loss
Chr10_CNVR_20	Chr10	27364051	27372811	8761	2.098832	4.28362	0	Gain	Loss
Chr10_CNVR_21	Chr10	27635611	27660139	24529	1.347704	2.54507	0	Gain	Loss
Chr10_CNVR_22	Chr10	33084331	33098347	14017	1.18737	2.27737	0	Gain	Loss
Chr10_CNVR_37	Chr10	36067987	36077623	9637	-0.8581268	0.551668	3.45E-187	Loss	Gain
Chr10_CNVR_38	Chr10	37351327	37362715	11389	-0.8261677	0.564026	5.40E-207	Loss	Gain
Chr10_CNVR_39	Chr10	37367095	37379359	12265	-0.8815607	0.54278	9.22E-249	Loss	Gain
Chr10_CNVR_23	Chr10	41589415	41599051	9637	1.426073	2.68714	0	Gain	Loss
Chr10_CNVR_24	Chr10	53139475	53155243	15769	1.038081	2.05349	0	Gain	Loss
Chr10_CNVR_40	Chr10	65506843	65517355	10513	-0.7962364	0.575849	1.81E-179	Loss	Gain
Chr10_CNVR_25	Chr10	79247779	79262671	14893	1.342099	2.5352	0	Gain	Loss
Chr10_CNVR_41	Chr10	80967367	80976127	8761	-0.8787095	0.543854	2.57E-177	Loss	Gain
Chr10_CNVR_42	Chr10	86256655	86268043	11389	-0.8233842	0.565115	8.46E-206	Loss	Gain
Chr10_CNVR_43	Chr10	86269795	86280307	10513	-0.8805188	0.543172	4.21E-213	Loss	Gain
Chr10_CNVR_44	Chr10	88671787	88681423	9637	-0.8606615	0.5507	3.98E-188	Loss	Gain
Chr10_CNVR_45	Chr10	89189503	89202643	13141	-0.8128222	0.569267	6.30E-232	Loss	Gain
Chr10_CNVR_46	Chr10	89252575	89261335	8761	-0.8217747	0.565746	2.98E-158	Loss	Gain
Chr10_CNVR_47	Chr10	96710839	96720475	9637	-2.076961	0.237013	0	Loss	Gain
Chr10_CNVR_48	Chr10	100027375	100044895	17521	-0.9185388	0.529045	0	Loss	Gain
Chr10_CNVR_49	Chr10	103120531	103131043	10513	-0.8004188	0.574182	4.23E-181	Loss	Gain
Chr12_CNVR_1	Chr12	6718843	6727683	8841	0.8479306	1.79992	2.56E-174	Gain	Loss
Chr12_CNVR_33	Chr12	15563263	15572103	8841	-1.062884	0.478674	3.85E-243	Loss	Gain
Chr12_CNVR_2	Chr12	24263591	24274199	10609	0.9887578	1.98448	1.41E-271	Gain	Loss
Chr12_CNVR_34	Chr12	31002323	31013815	11493	-0.8363831	0.560046	9.28E-212	Loss	Gain
Chr12_CNVR_35	Chr12	32023343	32070195	46853	-3.235371	0.106183	0	Loss	Gain
Chr12_CNVR_36	Chr12	32071079	32100251	29173	-4.124964	0.0573142	0	Loss	Gain
Chr12_CNVR_37	Chr12	32102019	32189535	87517	-3.015104	0.123698	0	Loss	Gain
Chr12_CNVR_38	Chr12	32191303	32201027	9725	-2.613202	0.163436	0	Loss	Gain
Chr12_CNVR_39	Chr12	32305339	32316831	11493	-0.8100415	0.570365	2.21E-200	Loss	Gain
Chr12_CNVR_40	Chr12	32995743	33007235	11493	-0.9350836	0.523012	7.95E-256	Loss	Gain
Chr12_CNVR_41	Chr12	34517107	34527715	10609	-0.8404806	0.558458	2.80E-197	Loss	Gain
Chr12_CNVR_42	Chr12	34540091	34548931	8841	-0.8727228	0.546115	1.16E-175	Loss	Gain
Chr12_CNVR_43	Chr12	35463871	35475363	11493	-0.8437966	0.557175	5.42E-215	Loss	Gain
Chr12_CNVR_3	Chr12	70639115	70657679	18565	2.694945	6.47529	0	Gain	Loss
Chr12_CNVR_4	Chr12	70725747	70769063	43317	1.04448	2.06262	0	Gain	Loss
Chr12_CNVR_5	Chr12	70784975	70815915	30941	1.260374	2.39558	0	Gain	Loss
Chr12_CNVR_6	Chr12	70817683	70998903	181221	4.524683	23.0179	0	Gain	Loss
Chr12_CNVR_7	Chr12	71000671	71010395	9725	3.847025	14.3903	0	Gain	Loss
Chr12_CNVR_8	Chr12	71021003	71033379	12377	1.091101	2.13037	0	Gain	Loss
Chr12_CNVR_9	Chr12	71041335	71215483	174149	4.787911	27.6252	0	Gain	Loss
Chr12_CNVR_44	Chr12	71281783	71304767	22985	-0.8983577	0.536497	0	Loss	Gain
Chr12_CNVR_45	Chr12	71305651	71346315	40665	-1.831264	0.281018	0	Loss	Gain
Chr12_CNVR_10	Chr12	71348967	71362227	13261	1.043265	2.06089	0	Gain	Loss
Chr12_CNVR_11	Chr12	71459467	71473611	14145	1.244673	2.36965	0	Gain	Loss
Chr12_CNVR_12	Chr12	71474495	71496595	22101	1.007643	2.01062	0	Gain	Loss
Chr12_CNVR_13	Chr12	71497479	71506319	8841	1.032018	2.04488	2.24E-243	Gain	Loss
Chr12_CNVR_14	Chr12	71508971	71518695	9725	0.9342099	1.91084	2.27E-226	Gain	Loss

Chr12_CNVR_15	Chr12	71549635	71574387	24753	1.122313	2.17696	0	Gain	Loss
Chr12_CNVR_46	Chr12	71653947	71665439	11493	-1.96233	0.256614	0	Loss	Gain
Chr12_CNVR_16	Chr12	71681351	71690191	8841	1.106602	2.15338	1.15E-272	Gain	Loss
Chr12_CNVR_47	Chr12	71811299	71828979	17681	-1.059056	0.479946	0	Loss	Gain
Chr12_CNVR_48	Chr12	71835167	71895279	60113	-1.780223	0.291138	0	Loss	Gain
Chr12_CNVR_17	Chr12	71954507	71964231	9725	0.8926469	1.85658	2.14E-209	Gain	Loss
Chr12_CNVR_18	Chr12	71996055	72005779	9725	0.8910481	1.85452	9.47E-209	Gain	Loss
Chr12_CNVR_49	Chr12	72033183	72050863	17681	-1.027679	0.490499	0	Loss	Gain
Chr12_CNVR_19	Chr12	72174623	72195839	21217	0.9689731	1.95745	0	Gain	Loss
Chr12_CNVR_50	Chr12	72206447	72243575	37129	-1.601569	0.329518	0	Loss	Gain
Chr12_CNVR_20	Chr12	72245343	72255951	10609	0.9562512	1.94026	1.02E-256	Gain	Loss
Chr12_CNVR_51	Chr12	72509659	72734195	224537	-5.092275	0.0293138	0	Loss	Gain
Chr12_CNVR_52	Chr12	72736847	72745687	8841	-4.688685	0.0387762	0	Loss	Gain
Chr12_CNVR_53	Chr12	72842043	72851767	9725	-1.190997	0.438	0	Loss	Gain
Chr12_CNVR_54	Chr12	72870331	72881823	11493	-1.731722	0.301092	0	Loss	Gain
Chr12_CNVR_55	Chr12	72911879	72933095	21217	-2.398548	0.189655	0	Loss	Gain
Chr12_CNVR_56	Chr12	72951659	72967571	15913	-2.044806	0.242355	0	Loss	Gain
Chr12_CNVR_57	Chr12	72988787	72998511	9725	-1.487658	0.356591	0	Loss	Gain
Chr12_CNVR_58	Chr12	73019727	73028567	8841	-1.123824	0.458876	1.61E-265	Loss	Gain
Chr12_CNVR_59	Chr12	73035639	73062159	26521	-1.155476	0.448918	0	Loss	Gain
Chr12_CNVR_60	Chr12	73141719	73162051	20333	-1.599626	0.329963	0	Loss	Gain
Chr12_CNVR_61	Chr12	73241611	73264595	22985	-2.790227	0.144563	0	Loss	Gain
Chr12_CNVR_21	Chr12	73306143	73323823	17681	1.107431	2.15462	0	Gain	Loss
Chr12_CNVR_62	Chr12	73379515	73391007	11493	-2.041026	0.242991	0	Loss	Gain
Chr12_CNVR_22	Chr12	73523607	73549243	25637	1.097092	2.13923	0	Gain	Loss
Chr12_CNVR_23	Chr12	73550127	73573111	22985	1.227336	2.34134	0	Gain	Loss
Chr12_CNVR_63	Chr12	73584603	73702175	117573	-2.594695	0.165546	0	Loss	Gain
Chr12_CNVR_24	Chr12	73755215	73764939	9725	2.186318	4.55142	0	Gain	Loss
Chr12_CNVR_64	Chr12	73815327	73846267	30941	-1.746737	0.297975	0	Loss	Gain
Chr12_CNVR_65	Chr12	73885163	73916987	31825	-2.437999	0.184539	0	Loss	Gain
Chr12_CNVR_66	Chr12	73920523	73957651	37129	-1.918563	0.264518	0	Loss	Gain
Chr12_CNVR_67	Chr12	73990359	73999199	8841	-1.366285	0.387889	0	Loss	Gain
Chr12_CNVR_68	Chr12	74001851	74011575	9725	-2.108477	0.231892	0	Loss	Gain
Chr12_CNVR_69	Chr12	74035443	74046051	10609	-1.714658	0.304675	0	Loss	Gain
Chr12_CNVR_70	Chr12	74046935	74075223	28289	-3.8385	0.0699031	0	Loss	Gain
Chr12_CNVR_71	Chr12	74090251	74099975	9725	-3.865365	0.0686134	0	Loss	Gain
Chr12_CNVR_72	Chr12	74118539	74137103	18565	-3.653343	0.0794757	0	Loss	Gain
Chr12_CNVR_73	Chr12	74137987	74170695	32709	-2.721402	0.151627	0	Loss	Gain
Chr12_CNVR_74	Chr12	74249371	74278543	29173	-2.117708	0.230413	0	Loss	Gain
Chr12_CNVR_75	Chr12	74288267	74304179	15913	-2.27897	0.206045	0	Loss	Gain
Chr12_CNVR_76	Chr12	74657779	74671039	13261	-1.332435	0.397097	0	Loss	Gain
Chr12_CNVR_77	Chr12	74680763	74713471	32709	-2.978169	0.126906	0	Loss	Gain
Chr12_CNVR_78	Chr12	74714355	74726731	12377	-1.920502	0.264163	0	Loss	Gain
Chr12_CNVR_79	Chr12	74770931	75041435	270505	-3.037057	0.12183	0	Loss	Gain
Chr12_CNVR_80	Chr12	75106851	75136023	29173	-3.796941	0.071946	0	Loss	Gain
Chr12_CNVR_81	Chr12	75136907	75146631	9725	-1.329325	0.397954	0	Loss	Gain
Chr12_CNVR_82	Chr12	75147515	75185527	38013	-2.082994	0.236024	0	Loss	Gain
Chr12_CNVR_83	Chr12	75186411	75217351	30941	-2.29162	0.204246	0	Loss	Gain
Chr12_CNVR_84	Chr12	75223539	75233263	9725	-1.278093	0.41234	0	Loss	Gain
Chr12_CNVR_85	Chr12	75427743	75444539	16797	-1.950985	0.25864	0	Loss	Gain
Chr12_CNVR_86	Chr12	75448075	75463103	15029	-0.9713492	0.510029	0	Loss	Gain
Chr12_CNVR_87	Chr12	75546199	75561227	15029	-1.292733	0.408177	0	Loss	Gain
Chr12_CNVR_88	Chr12	75577139	75607195	30057	-1.530388	0.346184	0	Loss	Gain
Chr12_CNVR_89	Chr12	75608963	75619571	10609	-0.9168475	0.529665	1.18E-228	Loss	Gain
Chr12_CNVR_25	Chr12	75957259	75967867	10609	3.255356	9.54904	0	Gain	Loss
Chr12_CNVR_26	Chr12	75972287	75990851	18565	1.882614	3.68743	0	Gain	Loss

Chr12_CNVR_27	Chr12	75997039	76012067	15029	1.643057	3.12327	0	Gain	Loss
Chr12_CNVR_90	Chr12	76077483	76086323	8841	-1.825933	0.282059	0	Loss	Gain
Chr12_CNVR_91	Chr12	76233951	76248979	15029	-2.132762	0.228021	0	Loss	Gain
Chr12_CNVR_28	Chr12	76312627	76322351	9725	1.219854	2.32923	0	Gain	Loss
Chr12_CNVR_29	Chr12	76346219	76406331	60113	1.377126	2.5975	0	Gain	Loss
Chr12_CNVR_30	Chr12	76407215	76419591	12377	1.537058	2.90202	0	Gain	Loss
Chr12_CNVR_92	Chr12	76460255	76471747	11493	-2.643267	0.160065	0	Loss	Gain
Chr12_CNVR_31	Chr12	76472631	76481471	8841	1.380438	2.60347	0	Gain	Loss
Chr12_CNVR_93	Chr12	76481471	76498267	16797	-3.29479	0.101899	0	Loss	Gain
Chr12_CNVR_32	Chr12	76500035	76514179	14145	1.559406	2.94732	0	Gain	Loss
Chr12_CNVR_94	Chr12	76514179	76523903	9725	-3.317125	0.100333	0	Loss	Gain
Chr12_CNVR_95	Chr12	79438451	79449059	10609	-0.8193592	0.566694	7.99E-189	Loss	Gain
Chr12_CNVR_96	Chr12	88587851	88601995	14145	-0.8588803	0.55138	2.91E-272	Loss	Gain
Chr12_CNVR_97	Chr12	88643543	88654151	10609	-0.9114201	0.531661	2.19E-226	Loss	Gain
Chr12_CNVR_98	Chr12	88868079	88876919	8841	-0.9900417	0.503463	8.84E-217	Loss	Gain
Chr12_CNVR_99	Chr12	88877803	88906975	29173	-0.8847348	0.541587	0	Loss	Gain
Chr12_CNVR_100	Chr12	88983883	89001563	17681	-0.8686466	0.54766	0	Loss	Gain
Chr12_CNVR_101	Chr12	89013939	89026315	12377	-0.8899538	0.539631	2.31E-253	Loss	Gain
Chr12_CNVR_102	Chr12	89036923	89055487	18565	-1.01676	0.494225	0	Loss	Gain
Chr12_CNVR_103	Chr12	89074051	89082891	8841	-0.8615003	0.55038	7.68E-172	Loss	Gain
Chr12_CNVR_104	Chr12	89092615	89156263	63649	-0.9765159	0.508206	0	Loss	Gain
Chr12_CNVR_105	Chr12	89157147	89165987	8841	-0.9026412	0.534907	5.88E-186	Loss	Gain
Chr12_CNVR_106	Chr12	89181015	89193391	12377	-0.8788498	0.543801	5.04E-248	Loss	Gain
Chr12_CNVR_107	Chr12	89199579	89215491	15913	-0.9056656	0.533786	0	Loss	Gain
Chr12_CNVR_108	Chr12	89219911	89234055	14145	-0.8205774	0.566215	1.04E-251	Loss	Gain
Chr12_CNVR_109	Chr12	89237591	89253503	15913	-0.9512941	0.517168	0	Loss	Gain
Chr12_CNVR_110	Chr12	89254387	89264111	9725	-0.8940952	0.538085	4.29E-201	Loss	Gain
Chr12_CNVR_111	Chr12	89264995	89275603	10609	-0.837701	0.559535	3.68E-196	Loss	Gain
Chr12_CNVR_112	Chr12	89341019	89357815	16797	-0.8703588	0.547011	0	Loss	Gain
Chr12_CNVR_113	Chr12	89388755	89407319	18565	-0.877302	0.544385	0	Loss	Gain
Chr12_CNVR_114	Chr12	89423231	89438259	15029	-0.8992918	0.53615	0	Loss	Gain
Chr12_CNVR_115	Chr12	89443563	89474503	30941	-0.9226352	0.527545	0	Loss	Gain
Chr12_CNVR_116	Chr12	89491299	89501023	9725	-0.8593222	0.551211	5.26E-188	Loss	Gain
Chr12_CNVR_117	Chr12	89501907	89518703	16797	-0.9659434	0.511944	0	Loss	Gain
Chr12_CNVR_118	Chr12	89532847	89554063	21217	-0.9090014	0.532554	0	Loss	Gain
Chr12_CNVR_119	Chr12	89556715	89576163	19449	-0.993534	0.502246	0	Loss	Gain
Chr12_CNVR_120	Chr12	89598263	89607987	9725	-0.9405017	0.521052	6.85E-219	Loss	Gain
Chr12_CNVR_121	Chr12	89657491	89670751	13261	-0.9613114	0.51359	0	Loss	Gain
Chr12_CNVR_122	Chr12	89680475	89692851	12377	-0.8223331	0.565527	2.51E-221	Loss	Gain
Chr12_CNVR_123	Chr12	89694619	89705227	10609	-0.9251181	0.526637	4.05E-232	Loss	Gain
Chr12_CNVR_124	Chr12	89731747	89741471	9725	-0.8685199	0.547708	1.91E-191	Loss	Gain
Chr12_CNVR_125	Chr12	89752079	89766223	14145	-0.934568	0.523199	0	Loss	Gain
Chr12_CNVR_126	Chr12	89830755	89839595	8841	-0.8992542	0.536164	8.76E-185	Loss	Gain
Chr12_CNVR_127	Chr12	89840479	89854623	14145	-0.9558545	0.515536	0	Loss	Gain
Chr12_CNVR_128	Chr12	89855507	89878491	22985	-0.9514122	0.517126	0	Loss	Gain
Chr12_CNVR_129	Chr12	89879375	89925343	45969	-0.9336105	0.523546	0	Loss	Gain
Chr12_CNVR_130	Chr12	89926227	89954515	28289	-0.9665901	0.511714	0	Loss	Gain
Chr12_CNVR_131	Chr12	89981035	89991643	10609	-0.9008876	0.535557	5.31E-222	Loss	Gain
Chr12_CNVR_132	Chr12	90049103	90066783	17681	-0.864925	0.549075	0	Loss	Gain
Chr12_CNVR_133	Chr12	90068551	90080043	11493	-0.8566489	0.552234	1.25E-220	Loss	Gain
Chr12_CNVR_134	Chr12	90081811	90102143	20333	-0.9124156	0.531295	0	Loss	Gain
Chr12_CNVR_135	Chr12	90103911	90124243	20333	-0.9525147	0.516731	0	Loss	Gain
Chr12_CNVR_136	Chr12	90135735	90147227	11493	-1.003495	0.49879	1.89E-287	Loss	Gain
Chr12_CNVR_137	Chr12	90151647	90174631	22985	-0.9259268	0.526342	0	Loss	Gain
Chr12_CNVR_138	Chr12	90178167	90199383	21217	-0.9607335	0.513796	0	Loss	Gain
Chr12_CNVR_139	Chr12	90200267	90209991	9725	-0.8394281	0.558865	1.27E-180	Loss	Gain

Chr12_CNVR_140	Chr12	90210875	90226787	15913	-0.9526287	0.51669	0	Loss	Gain
Chr12_CNVR_141	Chr12	90229439	90281595	52157	-0.8990082	0.536255	0	Loss	Gain
Chr12_CNVR_142	Chr12	90298391	90316955	18565	-0.9653455	0.512156	0	Loss	Gain
Chr12_CNVR_143	Chr12	90331099	90347895	16797	-1.06454	0.478125	0	Loss	Gain
Chr12_CNVR_144	Chr12	90348779	90438063	89285	-1.057071	0.480607	0	Loss	Gain
Chr12_CNVR_145	Chr12	90439831	90473423	33593	-1.202232	0.434602	0	Loss	Gain
Chr12_CNVR_146	Chr12	90475191	90490219	15029	-1.171118	0.444077	0	Loss	Gain
Chr12_CNVR_147	Chr12	90491103	90521159	30057	-1.023068	0.492069	0	Loss	Gain
Chr12_CNVR_148	Chr12	90530883	90543259	12377	-0.9780625	0.507661	1.22E-296	Loss	Gain
Chr12_CNVR_149	Chr12	90544143	90559171	15029	-0.9139449	0.530732	0	Loss	Gain
Chr12_CNVR_150	Chr12	90573315	90596299	22985	-0.9737382	0.509185	0	Loss	Gain
Chr12_CNVR_151	Chr12	90610443	90619283	8841	-1.010705	0.496304	3.32E-224	Loss	Gain
Chr12_CNVR_152	Chr12	90626355	90641383	15029	-0.9821688	0.506218	0	Loss	Gain
Chr12_CNVR_153	Chr12	90642267	90664367	22101	-1.010696	0.496307	0	Loss	Gain
Chr12_CNVR_154	Chr12	90665251	90689119	23869	-0.9007679	0.535602	0	Loss	Gain
Chr12_CNVR_155	Chr12	90699727	90710335	10609	-0.9320644	0.524108	4.86E-235	Loss	Gain
Chr12_CNVR_156	Chr12	90728899	90762491	33593	-0.9682916	0.511111	0	Loss	Gain
Chr12_CNVR_157	Chr12	90778403	90819951	41549	-1.009324	0.496779	0	Loss	Gain
Chr12_CNVR_158	Chr12	90820835	90871223	50389	-1.026666	0.490843	0	Loss	Gain
Chr12_CNVR_159	Chr12	90872991	90892439	19449	-1.026344	0.490953	0	Loss	Gain
Chr12_CNVR_160	Chr12	90893323	90949015	55693	-1.017825	0.49386	0	Loss	Gain
Chr12_CNVR_161	Chr12	90949899	90963159	13261	-0.9644494	0.512474	0	Loss	Gain
Chr12_CNVR_162	Chr12	90964043	90990563	26521	-0.9484701	0.518182	0	Loss	Gain
Chr12_CNVR_163	Chr12	90991447	91001171	9725	-0.8555054	0.552672	1.39E-186	Loss	Gain
Chr15_CNVR_20	Chr15	447	15610	15164	-1.311633	0.402865	0	Loss	Gain
Chr15_CNVR_1	Chr15	9736627	9745546	8920	1.192675	2.28576	0	Gain	Loss
Chr15_CNVR_2	Chr15	11375231	11391286	16056	1.222975	2.33428	0	Gain	Loss
Chr15_CNVR_21	Chr15	24284255	24294066	9812	-0.8297369	0.562632	8.64E-177	Loss	Gain
Chr15_CNVR_22	Chr15	28601535	28611346	9812	-0.8643919	0.549278	1.62E-189	Loss	Gain
Chr15_CNVR_23	Chr15	28612239	28622942	10704	-0.8528365	0.553695	6.74E-202	Loss	Gain
Chr15_CNVR_24	Chr15	30739659	30761066	21408	-0.8719273	0.546416	0	Loss	Gain
Chr15_CNVR_25	Chr15	30762851	30774446	11596	-0.8873179	0.540618	1.10E-233	Loss	Gain
Chr15_CNVR_26	Chr15	30778907	30790502	11596	-0.9350247	0.523034	5.02E-255	Loss	Gain
Chr15_CNVR_27	Chr15	30800315	30810126	9812	-0.8459116	0.556359	1.06E-182	Loss	Gain
Chr15_CNVR_3	Chr15	31856443	31865362	8920	3.579198	11.9521	0	Gain	Loss
Chr15_CNVR_28	Chr15	46553927	46563738	9812	-1.719836	0.303583	0	Loss	Gain
Chr15_CNVR_4	Chr15	46826879	46838474	11596	1.117368	2.16951	0	Gain	Loss
Chr15_CNVR_5	Chr15	46878615	46890210	11596	1.006086	2.00845	0	Gain	Loss
Chr15_CNVR_6	Chr15	46891103	46902698	11596	1.231811	2.34862	0	Gain	Loss
Chr15_CNVR_7	Chr15	46909835	46918754	8920	0.9420254	1.92122	7.13E-216	Gain	Loss
Chr15_CNVR_8	Chr15	47817891	47854462	36572	4.052086	16.5882	0	Gain	Loss
Chr15_CNVR_9	Chr15	48069435	48078354	8920	0.9986636	1.99815	2.33E-238	Gain	Loss
Chr15_CNVR_10	Chr15	49090775	49129130	38356	1.40026	2.63949	0	Gain	Loss
Chr15_CNVR_29	Chr15	49295043	49310206	15164	-0.9733781	0.509312	0	Loss	Gain
Chr15_CNVR_30	Chr15	49311991	49320910	8920	-0.9757269	0.508484	6.57E-211	Loss	Gain
Chr15_CNVR_31	Chr15	50719567	50754354	34788	-2.573361	0.168012	0	Loss	Gain
Chr15_CNVR_32	Chr15	50789143	50807874	18732	-1.950178	0.258784	0	Loss	Gain
Chr15_CNVR_11	Chr15	50811443	50831066	19624	0.964242	1.95104	0	Gain	Loss
Chr15_CNVR_12	Chr15	51085287	51104910	19624	3.517807	11.4542	0	Gain	Loss
Chr15_CNVR_33	Chr15	52215451	52227938	12488	-1.381368	0.383855	0	Loss	Gain
Chr15_CNVR_34	Chr15	52228831	52256482	27652	-1.116162	0.461319	0	Loss	Gain
Chr15_CNVR_35	Chr15	52263619	52283242	19624	-2.775187	0.146078	0	Loss	Gain
Chr15_CNVR_36	Chr15	52549059	52561546	12488	-1.560882	0.338944	0	Loss	Gain
Chr15_CNVR_37	Chr15	52954027	52962946	8920	-0.8539873	0.553254	5.59E-169	Loss	Gain
Chr15_CNVR_38	Chr15	53017359	53033414	16056	-0.81176	0.569686	2.61E-277	Loss	Gain
Chr15_CNVR_39	Chr15	53053931	53063742	9812	-0.8992931	0.536149	1.48E-202	Loss	Gain

Chr15_CNVR_40	Chr15	53610539	53621242	10704	-0.8979881	0.536635	3.03E-220	Loss	Gain
Chr15_CNVR_41	Chr15	55408811	55418622	9812	-0.8076439	0.571314	8.36E-169	Loss	Gain
Chr15_CNVR_42	Chr15	56385551	56395362	9812	-0.8712653	0.546667	4.57E-192	Loss	Gain
Chr15_CNVR_43	Chr15	56851175	56869014	17840	-0.8467826	0.556023	0	Loss	Gain
Chr15_CNVR_44	Chr15	57215111	57226706	11596	-1.988106	0.25207	0	Loss	Gain
Chr15_CNVR_45	Chr15	57289147	57298066	8920	-0.9336969	0.523515	3.37E-196	Loss	Gain
Chr15_CNVR_46	Chr15	57300743	57316798	16056	-0.8219158	0.56569	2.63E-283	Loss	Gain
Chr15_CNVR_13	Chr15	64541999	64566082	24084	1.207517	2.3094	0	Gain	Loss
Chr15_CNVR_47	Chr15	75503787	75514490	10704	-0.8403276	0.558517	6.89E-197	Loss	Gain
Chr15_CNVR_48	Chr15	75774063	75783874	9812	-0.8640889	0.549393	2.10E-189	Loss	Gain
Chr15_CNVR_49	Chr15	76438603	76447522	8920	-1.012829	0.495574	4.64E-224	Loss	Gain
Chr15_CNVR_50	Chr15	76765967	76786482	20516	-0.8625594	0.549976	0	Loss	Gain
Chr15_CNVR_51	Chr15	76797187	76807890	10704	-0.9210625	0.52812	9.12E-230	Loss	Gain
Chr15_CNVR_52	Chr15	77168259	77179854	11596	-0.8197826	0.566527	2.74E-204	Loss	Gain
Chr15_CNVR_53	Chr15	77185207	77195910	10704	-0.9518653	0.516964	1.29E-242	Loss	Gain
Chr15_CNVR_54	Chr15	77197695	77224454	26760	-0.9310219	0.524487	0	Loss	Gain
Chr15_CNVR_55	Chr15	77228915	77244078	15164	-0.8971435	0.536949	0	Loss	Gain
Chr15_CNVR_56	Chr15	78375135	78387622	12488	-0.9176837	0.529358	4.51E-266	Loss	Gain
Chr15_CNVR_57	Chr15	78393867	78408138	14272	-0.9449439	0.51945	0	Loss	Gain
Chr15_CNVR_58	Chr15	78429547	78444710	15164	-0.8948781	0.537793	0	Loss	Gain
Chr15_CNVR_59	Chr15	78931743	78942446	10704	-0.7955371	0.576129	3.07E-179	Loss	Gain
Chr15_CNVR_60	Chr15	78944231	78953150	8920	-0.8195688	0.566611	1.50E-157	Loss	Gain
Chr15_CNVR_14	Chr15	79379527	79389338	9812	1.493849	2.81639	0	Gain	Loss
Chr15_CNVR_15	Chr15	79524923	79541870	16948	1.619204	3.07205	0	Gain	Loss
Chr15_CNVR_16	Chr15	79816607	79833554	16948	1.270716	2.41281	0	Gain	Loss
Chr15_CNVR_17	Chr15	80070827	80079746	8920	1.422646	2.68077	0	Gain	Loss
Chr15_CNVR_18	Chr15	80808511	80842406	33896	1.412918	2.66275	0	Gain	Loss
Chr15_CNVR_19	Chr15	81300003	81308922	8920	1.065588	2.09302	1.56E-265	Gain	Loss
Chr15_CNVR_61	Chr15	81681779	81690698	8920	-0.822116	0.565612	2.17E-158	Loss	Gain
Chr15_CNVR_62	Chr15	81695159	81707646	12488	-0.8686893	0.547644	1.17E-242	Loss	Gain
Chr15_CNVR_63	Chr15	81729947	81739758	9812	-0.8115766	0.569759	3.22E-170	Loss	Gain
Chr15_CNVR_64	Chr15	81781683	81802198	20516	-0.9230323	0.527399	0	Loss	Gain
Chr15_CNVR_65	Chr15	81804875	81820930	16056	-0.9844355	0.505423	0	Loss	Gain
Chr15_CNVR_66	Chr15	81822715	81839662	16948	-1.029716	0.489807	0	Loss	Gain
Chr15_CNVR_67	Chr15	81849475	81868206	18732	-1.135332	0.45523	0	Loss	Gain
Chr15_CNVR_68	Chr15	81869099	81929754	60656	-2.465691	0.181031	0	Loss	Gain
Chr15_CNVR_69	Chr15	81934215	81944918	10704	-1.07041	0.476184	3.13E-293	Loss	Gain
Chr15_CNVR_70	Chr15	81966327	81993086	26760	-0.871585	0.546546	0	Loss	Gain
Chr15_CNVR_71	Chr15	81993979	82006466	12488	-0.9452458	0.519341	1.85E-279	Loss	Gain
Chr15_CNVR_72	Chr15	82030551	82046606	16056	-1.00167	0.499422	0	Loss	Gain
Chr15_CNVR_73	Chr15	82051959	82072474	20516	-0.9073208	0.533174	0	Loss	Gain
Chr15_CNVR_74	Chr15	82105479	82116182	10704	-0.8981054	0.536591	2.72E-220	Loss	Gain
Chr15_CNVR_75	Chr15	82159891	82168810	8920	-0.8183535	0.567089	3.76E-157	Loss	Gain
Chr15_CNVR_76	Chr15	82185759	82194678	8920	-0.9026429	0.534906	1.76E-185	Loss	Gain
Chr15_CNVR_77	Chr15	84955419	84965230	9812	-0.8640751	0.549399	2.13E-189	Loss	Gain
Chr15_CNVR_78	Chr15	84996451	85007154	10704	-0.9212239	0.528061	7.81E-230	Loss	Gain
Chr15_CNVR_79	Chr15	85024103	85066026	41924	-1.030663	0.489485	0	Loss	Gain
Chr15_CNVR_80	Chr15	85074055	85091002	16948	-0.9090599	0.532532	0	Loss	Gain
Chr16_CNVR_10	Chr16	39250	56890	17641	-0.8552381	0.552774	0	Loss	Gain
Chr16_CNVR_11	Chr16	93052	102754	9703	-0.8935805	0.538277	6.20E-200	Loss	Gain
Chr16_CNVR_12	Chr16	112456	128332	15877	-0.8841163	0.541819	0	Loss	Gain
Chr16_CNVR_13	Chr16	160084	168904	8821	-0.8485907	0.555327	7.96E-167	Loss	Gain
Chr16_CNVR_14	Chr16	1729162	1746802	17641	-0.9613389	0.51358	0	Loss	Gain
Chr16_CNVR_15	Chr16	2589994	2601460	11467	-0.8421563	0.557809	1.78E-213	Loss	Gain
Chr16_CNVR_16	Chr16	2603224	2612044	8821	-0.9061781	0.533597	3.08E-186	Loss	Gain
Chr16_CNVR_17	Chr16	2616454	2630566	14113	-0.9073381	0.533168	2.42E-297	Loss	Gain

Chr16_CNVR_18	Chr16	2634976	2644678	9703	-0.9026719	0.534895	2.45E-203	Loss	Gain
Chr16_CNVR_19	Chr16	3075976	3087442	11467	-0.8312549	0.56204	8.51E-209	Loss	Gain
Chr16_CNVR_20	Chr16	4144078	4154662	10585	-0.9238438	0.527103	2.49E-230	Loss	Gain
Chr16_CNVR_21	Chr16	4179358	4189060	9703	-0.8235384	0.565054	3.32E-174	Loss	Gain
Chr16_CNVR_22	Chr16	5628484	5638186	9703	-1.742263	0.2989	0	Loss	Gain
Chr16_CNVR_23	Chr16	5702572	5712274	9703	-0.9917078	0.502882	2.69E-237	Loss	Gain
Chr16_CNVR_24	Chr16	5740498	5750200	9703	-0.9398474	0.521288	2.20E-217	Loss	Gain
Chr16_CNVR_1	Chr16	6027148	6042142	14995	1.139009	2.2023	0	Gain	Loss
Chr16_CNVR_2	Chr16	6057136	6094180	37045	1.204919	2.30524	0	Gain	Loss
Chr16_CNVR_3	Chr16	6097708	6119758	22051	1.265284	2.40375	0	Gain	Loss
Chr16_CNVR_4	Chr16	11523772	11533474	9703	3.092778	8.53137	0	Gain	Loss
Chr16_CNVR_5	Chr16	17787736	17801848	14113	0.8949017	1.85948	0	Gain	Loss
Chr16_CNVR_6	Chr16	29366632	29380744	14113	1.17956	2.26508	0	Gain	Loss
Chr16_CNVR_7	Chr16	29383390	29392210	8821	0.9441906	1.92411	1.13E-219	Gain	Loss
Chr16_CNVR_8	Chr16	29397502	29410732	13231	1.038425	2.05398	0	Gain	Loss
Chr16_CNVR_25	Chr16	30402100	30411802	9703	-0.8362415	0.560101	8.39E-179	Loss	Gain
Chr16_CNVR_9	Chr16	39448774	39476998	28225	2.890846	7.41705	0	Gain	Loss
Chr16_CNVR_26	Chr16	43706188	43722946	16759	-0.7971817	0.575472	4.42E-283	Loss	Gain
Chr16_CNVR_27	Chr16	43730002	43739704	9703	-0.7822382	0.581464	1.71E-159	Loss	Gain
Chr16_CNVR_28	Chr16	43741468	43750288	8821	-0.8004639	0.574165	4.87E-151	Loss	Gain
Chr16_CNVR_29	Chr16	43756462	43767046	10585	-0.934006	0.523403	1.56E-234	Loss	Gain
Chr16_CNVR_30	Chr16	43770574	43784686	14113	-0.9939119	0.502114	0	Loss	Gain
Chr16_CNVR_31	Chr16	43792624	43806736	14113	-0.8619375	0.550213	8.78E-273	Loss	Gain
Chr16_CNVR_32	Chr16	43809382	43818202	8821	-0.9758516	0.50844	2.38E-210	Loss	Gain
Chr16_CNVR_33	Chr16	44991262	45000082	8821	-0.8536584	0.55338	1.64E-168	Loss	Gain
Chr16_CNVR_34	Chr16	47720170	47730754	10585	-0.8706017	0.546919	1.33E-208	Loss	Gain
Chr16_CNVR_35	Chr16	47733400	47745748	12349	-0.9627667	0.513072	3.10E-287	Loss	Gain
Chr16_CNVR_36	Chr16	47753686	47764270	10585	-0.9549567	0.515857	3.03E-243	Loss	Gain
Chr16_CNVR_37	Chr16	47765152	47775736	10585	-0.8580503	0.551698	1.47E-203	Loss	Gain
Chr16_CNVR_38	Chr16	47920384	47934496	14113	-0.9234465	0.527248	3.58E-306	Loss	Gain
Chr16_CNVR_39	Chr16	47938906	47951254	12349	-0.883666	0.541988	3.50E-249	Loss	Gain
Chr16_CNVR_40	Chr16	48082672	48091492	8821	-0.827972	0.563321	5.21E-160	Loss	Gain
Chr16_CNVR_41	Chr16	48120598	48130300	9703	-0.847943	0.555576	4.58E-183	Loss	Gain
Chr16_CNVR_42	Chr16	48131182	48142648	11467	-0.851361	0.554262	1.90E-217	Loss	Gain
Chr16_CNVR_43	Chr16	49315708	49325410	9703	-0.8918615	0.538918	2.72E-199	Loss	Gain
Chr16_CNVR_44	Chr16	49373920	49388032	14113	-1.076713	0.474108	0	Loss	Gain
Chr16_CNVR_45	Chr16	49389796	49403026	13231	-0.875908	0.544911	6.67E-263	Loss	Gain
Chr16_CNVR_46	Chr16	49416256	49425958	9703	-1.034518	0.488179	5.81E-254	Loss	Gain
Chr16_CNVR_47	Chr16	49449772	49485052	35281	-0.9846606	0.505345	0	Loss	Gain
Chr16_CNVR_48	Chr16	49485934	49511512	25579	-0.9361379	0.52263	0	Loss	Gain
Chr16_CNVR_49	Chr16	49514158	49537090	22933	-0.9315206	0.524305	0	Loss	Gain
Chr16_CNVR_50	Chr16	49539736	49555612	15877	-0.9692776	0.510762	0	Loss	Gain
Chr16_CNVR_51	Chr16	49564432	49578544	14113	-0.8779353	0.544146	2.23E-281	Loss	Gain
Chr16_CNVR_52	Chr16	49582072	49593538	11467	-0.9590127	0.514409	3.59E-265	Loss	Gain
Chr16_CNVR_53	Chr16	49626172	49643812	17641	-0.9967071	0.501143	0	Loss	Gain
Chr16_CNVR_54	Chr16	49661452	49670272	8821	-0.9419387	0.520533	1.55E-198	Loss	Gain
Chr16_CNVR_55	Chr16	50629006	50651056	22051	-0.9097732	0.532269	0	Loss	Gain
Chr16_CNVR_56	Chr16	50662522	50673106	10585	-0.9310791	0.524466	2.55E-233	Loss	Gain
Chr16_CNVR_57	Chr16	50676634	50685454	8821	-0.9868806	0.504568	3.22E-214	Loss	Gain
Chr16_CNVR_58	Chr16	50690746	50707504	16759	-0.9200611	0.528487	0	Loss	Gain
Chr16_CNVR_59	Chr16	50718970	50731318	12349	-0.9146762	0.530463	5.86E-264	Loss	Gain
Chr16_CNVR_60	Chr16	50732200	50746312	14113	-1.060417	0.479493	0	Loss	Gain
Chr16_CNVR_61	Chr16	50753368	50791294	37927	-0.9546438	0.515969	0	Loss	Gain
Chr16_CNVR_62	Chr16	50808934	50821282	12349	-0.8616335	0.550329	8.24E-239	Loss	Gain
Chr16_CNVR_63	Chr16	50822164	50840686	18523	-0.9702717	0.51041	0	Loss	Gain
Chr16_CNVR_64	Chr16	50841568	50853916	12349	-0.8639426	0.549449	6.82E-240	Loss	Gain

Chr16_CNVR_65	Chr16	50861854	50876848	14995	-0.9361516	0.522625	0	Loss	Gain
Chr16_CNVR_66	Chr16	50898016	50911246	13231	-0.9545645	0.515997	2.59E-303	Loss	Gain
Chr16_CNVR_67	Chr16	50913010	50926240	13231	-0.9838325	0.505635	0	Loss	Gain
Chr16_CNVR_68	Chr16	50931532	50943880	12349	-0.8379277	0.559447	8.90E-228	Loss	Gain
Chr16_CNVR_69	Chr16	50945644	50958874	13231	-0.9479504	0.518368	7.24E-300	Loss	Gain
Chr16_CNVR_70	Chr16	50995918	51006502	10585	-0.9500925	0.517599	3.24E-241	Loss	Gain
Chr16_CNVR_71	Chr16	51053248	51067360	14113	-0.9314637	0.524326	0	Loss	Gain
Chr16_CNVR_72	Chr16	51080590	51089410	8821	-1.013081	0.495487	1.85E-223	Loss	Gain
Chr16_CNVR_73	Chr16	51099112	51123808	24697	-0.9105121	0.531996	0	Loss	Gain
Chr16_CNVR_74	Chr16	51124690	51145858	21169	-0.9720162	0.509793	0	Loss	Gain
Chr16_CNVR_75	Chr16	51147622	51167908	20287	-0.9527107	0.516661	0	Loss	Gain
Chr16_CNVR_76	Chr16	51169672	51196132	26461	-0.8870614	0.540714	0	Loss	Gain
Chr16_CNVR_77	Chr16	51197896	51220828	22933	-0.9557865	0.51556	0	Loss	Gain
Chr16_CNVR_78	Chr16	51383116	51391936	8821	-0.8663514	0.548532	9.34E-173	Loss	Gain
Chr16_CNVR_79	Chr16	51393700	51402520	8821	-0.8601961	0.550878	1.08E-170	Loss	Gain
Chr16_CNVR_80	Chr16	51555106	51570100	14995	-0.8957381	0.537472	0	Loss	Gain
Chr16_CNVR_81	Chr16	51571864	51614200	42337	-0.9897015	0.503582	0	Loss	Gain
Chr16_CNVR_82	Chr16	51615082	51633604	18523	-0.9479731	0.51836	0	Loss	Gain
Chr16_CNVR_83	Chr16	51642424	51651244	8821	-0.9177663	0.529328	3.33E-190	Loss	Gain
Chr16_CNVR_84	Chr16	51662710	51688288	25579	-0.8830051	0.542237	0	Loss	Gain
Chr16_CNVR_85	Chr16	51697990	51711220	13231	-0.9055347	0.533835	5.70E-278	Loss	Gain
Chr16_CNVR_86	Chr16	51732388	51750910	18523	-0.9151354	0.530294	0	Loss	Gain
Chr16_CNVR_87	Chr16	51769432	51791482	22051	-0.932799	0.523841	0	Loss	Gain
Chr16_CNVR_88	Chr16	51793246	51818824	25579	-0.9619145	0.513375	0	Loss	Gain
Chr16_CNVR_89	Chr16	51822352	51847048	24697	-0.9337266	0.523504	0	Loss	Gain
Chr16_CNVR_90	Chr16	51863806	51873508	9703	-0.9405161	0.521046	1.22E-217	Loss	Gain
Chr16_CNVR_91	Chr16	52362136	52375366	13231	-0.8626962	0.549924	3.00E-256	Loss	Gain
Chr16_CNVR_92	Chr16	52432696	52455628	22933	-0.8897655	0.539702	0	Loss	Gain
Chr16_CNVR_93	Chr16	52477678	52486498	8821	-0.8751851	0.545184	1.00E-175	Loss	Gain
Chr16_CNVR_94	Chr16	52581754	52591456	9703	-0.8458681	0.556376	2.62E-182	Loss	Gain
Chr16_CNVR_95	Chr16	52608214	52619680	11467	-0.9093103	0.53244	7.18E-243	Loss	Gain
Chr16_CNVR_96	Chr16	52751980	52771384	19405	-0.870786	0.546849	0	Loss	Gain
Chr16_CNVR_97	Chr16	52787260	52796080	8821	-0.8722352	0.5463	9.87E-175	Loss	Gain
Chr16_CNVR_98	Chr16	53792740	53801560	8821	-0.8524284	0.553852	4.21E-168	Loss	Gain
Chr16_CNVR_99	Chr16	55315954	55324774	8821	-0.9874752	0.50436	1.99E-214	Loss	Gain
Chr16_CNVR_100	Chr16	55326538	55336240	9703	-0.8463797	0.556179	1.71E-182	Loss	Gain
Chr16_CNVR_101	Chr16	70827688	70838272	10585	-0.9733674	0.509316	5.95E-251	Loss	Gain
Chr16_CNVR_102	Chr16	70839154	70847974	8821	-1.0025	0.499134	1.01E-219	Loss	Gain
Chr16_CNVR_103	Chr16	70856794	70869142	12349	-0.971229	0.510071	2.26E-291	Loss	Gain
Chr16_CNVR_104	Chr16	70878844	70920298	41455	-0.9993285	0.500233	0	Loss	Gain
Chr16_CNVR_105	Chr16	70937938	70976746	38809	-0.9123963	0.531302	0	Loss	Gain
Chr16_CNVR_106	Chr16	70977628	70990858	13231	-0.9391596	0.521537	2.67E-295	Loss	Gain
Chr16_CNVR_107	Chr16	70992622	71002324	9703	-0.8807001	0.543104	3.89E-195	Loss	Gain
Chr16_CNVR_108	Chr16	71045542	71056126	10585	-0.8778801	0.544166	1.52E-211	Loss	Gain
Chr16_CNVR_109	Chr16	71089642	71098462	8821	-0.8918089	0.538938	2.40E-181	Loss	Gain
Chr16_CNVR_110	Chr16	71131096	71151382	20287	-0.9148928	0.530383	0	Loss	Gain
Chr16_CNVR_111	Chr16	71153146	71161966	8821	-0.9615148	0.513517	2.44E-205	Loss	Gain
Chr16_CNVR_112	Chr16	77075776	77084596	8821	-0.884583	0.541644	6.75E-179	Loss	Gain
Chr16_CNVR_113	Chr16	77123404	77138398	14995	-0.8451656	0.556647	3.35E-280	Loss	Gain
Chr16_CNVR_114	Chr16	77178088	77187790	9703	-0.9375068	0.522134	1.71E-216	Loss	Gain
Chr16_CNVR_115	Chr16	77238946	77248648	9703	-0.7841332	0.580701	3.69E-160	Loss	Gain
Chr16_CNVR_116	Chr16	81374644	81388756	14113	-0.932399	0.523986	0	Loss	Gain
Chr16_CNVR_117	Chr16	81395812	81408160	12349	-0.9959042	0.501422	1.70E-303	Loss	Gain
Chr16_CNVR_118	Chr16	81417862	81427564	9703	-0.8965487	0.53717	4.82E-201	Loss	Gain
Chr16_CNVR_119	Chr16	81484894	81506062	21169	-0.9808674	0.506675	0	Loss	Gain
Chr16_CNVR_120	Chr16	81528112	81554572	26461	-0.901549	0.535312	0	Loss	Gain

Chr16_CNVR_121	Chr16	81569566	81583678	14113	-1.001198	0.499585	0	Loss	Gain
Chr16_CNVR_122	Chr16	81587206	81602200	14995	-0.9688596	0.51091	0	Loss	Gain
Chr16_CNVR_123	Chr16	81618076	81628660	10585	-1.00514	0.498222	2.34E-264	Loss	Gain
Chr16_CNVR_124	Chr16	81629542	81651592	22051	-0.9284927	0.525407	0	Loss	Gain
Chr16_CNVR_125	Chr16	81655120	81667468	12349	-0.9502214	0.517553	4.00E-281	Loss	Gain
Chr16_CNVR_126	Chr16	81684226	81707158	22933	-0.9604751	0.513888	0	Loss	Gain
Chr17_CNVR_15	Chr17	1986474	1995122	8649	-0.9175202	0.529418	2.11E-190	Loss	Gain
Chr17_CNVR_16	Chr17	1996854	2005502	8649	-0.8493574	0.555032	2.66E-167	Loss	Gain
Chr17_CNVR_1	Chr17	8807864	8818242	10379	1.289323	2.44413	0	Gain	Loss
Chr17_CNVR_2	Chr17	8848518	8866682	18165	1.394362	2.62872	0	Gain	Loss
Chr17_CNVR_3	Chr17	8896094	8905608	9515	1.117604	2.16986	0	Gain	Loss
Chr17_CNVR_4	Chr17	8914258	8926368	12111	1.035596	2.04996	0	Gain	Loss
Chr17_CNVR_5	Chr17	8927234	8942802	15569	1.063756	2.09037	0	Gain	Loss
Chr17_CNVR_17	Chr17	14332618	14350782	18165	-1.793709	0.28843	0	Loss	Gain
Chr17_CNVR_6	Chr17	15323044	15332558	9515	2.948872	7.72145	0	Gain	Loss
Chr17_CNVR_7	Chr17	21002634	21013012	10379	0.9080636	1.87653	6.08E-245	Gain	Loss
Chr17_CNVR_8	Chr17	21083078	21096918	13841	3.894345	14.8701	0	Gain	Loss
Chr17_CNVR_9	Chr17	24686668	24696182	9515	0.8253167	1.77192	5.09E-190	Gain	Loss
Chr17_CNVR_18	Chr17	25030074	25044778	14705	-0.8125109	0.56939	2.79E-262	Loss	Gain
Chr17_CNVR_10	Chr17	28279014	28287662	8649	1.668107	3.17797	0	Gain	Loss
Chr17_CNVR_11	Chr17	30044478	30053128	8651	0.7962126	1.73654	3.10E-162	Gain	Loss
Chr17_CNVR_12	Chr17	31412908	31425018	12111	0.8796339	1.83991	5.33E-270	Gain	Loss
Chr17_CNVR_19	Chr17	45646484	45655998	9515	-0.9071081	0.533253	2.66E-205	Loss	Gain
Chr17_CNVR_20	Chr17	45662054	45681082	19029	-0.8762046	0.544799	0	Loss	Gain
Chr17_CNVR_21	Chr17	45682814	45695788	12975	-0.899235	0.536171	3.79E-275	Loss	Gain
Chr17_CNVR_22	Chr17	45696654	45711358	14705	-0.913564	0.530872	0	Loss	Gain
Chr17_CNVR_23	Chr17	45712224	45722602	10379	-0.8762576	0.544779	3.51E-211	Loss	Gain
Chr17_CNVR_24	Chr17	51371918	51397002	25085	-1.039289	0.486567	0	Loss	Gain
Chr17_CNVR_13	Chr17	52440194	52450572	10379	4.868356	29.2093	0	Gain	Loss
Chr17_CNVR_25	Chr17	53467814	53482518	14705	-0.8840186	0.541856	9.53E-303	Loss	Gain
Chr17_CNVR_26	Chr17	53533554	53543068	9515	-0.940884	0.520914	4.09E-218	Loss	Gain
Chr17_CNVR_27	Chr17	53562098	53575072	12975	-0.8858937	0.541152	2.44E-268	Loss	Gain
Chr17_CNVR_28	Chr17	53586318	53606212	19895	-0.8420397	0.557854	0	Loss	Gain
Chr17_CNVR_29	Chr17	53625244	53636488	11245	-1.033396	0.488559	9.55E-300	Loss	Gain
Chr17_CNVR_30	Chr17	53637354	53649462	12109	-0.9447719	0.519512	6.56E-279	Loss	Gain
Chr17_CNVR_31	Chr17	53666764	53715202	48439	-0.9259118	0.526348	0	Loss	Gain
Chr17_CNVR_32	Chr17	53718664	53727312	8649	-0.8770178	0.544492	1.38E-176	Loss	Gain
Chr17_CNVR_33	Chr17	62943888	62957728	13841	-0.9303643	0.524726	0	Loss	Gain
Chr17_CNVR_34	Chr17	62962918	62971568	8651	-0.9466213	0.518846	1.80E-200	Loss	Gain
Chr17_CNVR_35	Chr17	62975028	63006168	31141	-0.8843462	0.541733	0	Loss	Gain
Chr17_CNVR_36	Chr17	63013954	63024332	10379	-0.8732998	0.545897	5.54E-210	Loss	Gain
Chr17_CNVR_37	Chr17	63026064	63038172	12109	-0.7875656	0.579321	4.67E-205	Loss	Gain
Chr17_CNVR_38	Chr17	63122944	63131592	8649	-0.8086503	0.570916	6.99E-154	Loss	Gain
Chr17_CNVR_39	Chr17	65640094	65648742	8649	-0.9017593	0.535234	5.36E-185	Loss	Gain
Chr17_CNVR_40	Chr17	65691128	65703238	12111	-0.8826871	0.542356	4.53E-249	Loss	Gain
Chr17_CNVR_41	Chr17	65710158	65718808	8651	-0.8037478	0.572859	2.75E-152	Loss	Gain
Chr17_CNVR_42	Chr17	65840774	65854612	13839	-0.854771	0.552953	2.57E-269	Loss	Gain
Chr17_CNVR_43	Chr17	65857208	65867588	10381	-0.8698549	0.547202	1.37E-208	Loss	Gain
Chr17_CNVR_44	Chr17	65874508	65883158	8651	-0.7861686	0.579882	1.32E-146	Loss	Gain
Chr17_CNVR_45	Chr17	65903918	65914298	10381	-0.825772	0.56418	5.64E-191	Loss	Gain
Chr17_CNVR_46	Chr17	66812168	66820818	8651	-0.8529196	0.553663	1.72E-168	Loss	Gain
Chr17_CNVR_47	Chr17	66854554	66864932	10379	-0.856875	0.552147	2.32E-203	Loss	Gain
Chr17_CNVR_48	Chr17	67403828	67413342	9515	-0.9298157	0.524925	6.75E-214	Loss	Gain
Chr17_CNVR_49	Chr17	67414208	67428912	14705	-0.8713559	0.546633	1.79E-295	Loss	Gain
Chr17_CNVR_50	Chr17	67451404	67460052	8649	-0.8955291	0.53755	7.17E-183	Loss	Gain
Chr17_CNVR_51	Chr17	67506764	67517142	10379	-0.8563946	0.552331	3.61E-203	Loss	Gain

Chr17_CNVR_52	Chr17	69193514	69203028	9515	-0.8380699	0.559391	1.06E-179	Loss	Gain
Chr17_CNVR_53	Chr17	69234168	69242818	8651	-0.755927	0.592166	5.46E-137	Loss	Gain
Chr17_CNVR_54	Chr17	69356998	69369972	12975	-0.9326285	0.523903	2.32E-292	Loss	Gain
Chr17_CNVR_55	Chr17	69395058	69409762	14705	-0.908453	0.532756	0	Loss	Gain
Chr17_CNVR_56	Chr17	69414088	69423602	9515	-0.8480896	0.55552	2.32E-183	Loss	Gain
Chr17_CNVR_14	Chr17	70111278	70120792	9515	1.468803	2.76792	0	Gain	Loss
Chr17_CNVR_57	Chr17	70947734	70958978	11245	-0.876775	0.544583	7.86E-229	Loss	Gain
Chr17_CNVR_58	Chr17	70972818	70990118	17301	-0.8888202	0.540056	0	Loss	Gain
Chr17_CNVR_59	Chr17	71003094	71012608	9515	-1.10071	0.466287	1.46E-280	Loss	Gain
Chr17_CNVR_60	Chr17	71018664	71031638	12975	-0.8671664	0.548223	7.52E-259	Loss	Gain
Chr17_CNVR_61	Chr17	71426078	71436458	10381	-0.8500387	0.55477	1.27E-200	Loss	Gain
Chr17_CNVR_62	Chr17	71438188	71452028	13841	-0.9494617	0.517826	0	Loss	Gain
Chr17_CNVR_63	Chr17	71459814	71471922	12109	-0.9585612	0.51457	1.24E-285	Loss	Gain
Chr17_CNVR_64	Chr17	71477114	71488358	11245	-0.90643	0.533504	6.06E-242	Loss	Gain
Chr17_CNVR_65	Chr17	71497008	71507388	10381	-0.9026434	0.534906	6.09E-222	Loss	Gain
Chr17_CNVR_66	Chr17	71509984	71522958	12975	-0.9253389	0.526557	1.39E-288	Loss	Gain
Chr17_CNVR_67	Chr17	71524688	71533338	8651	-1.017434	0.493994	2.20E-225	Loss	Gain
Chr17_CNVR_68	Chr17	71569668	71580048	10381	-0.8305192	0.562327	7.49E-193	Loss	Gain
Chr17_CNVR_69	Chr17	71586104	71597348	11245	-0.858188	0.551645	1.05E-220	Loss	Gain
Chr17_CNVR_70	Chr17	72581718	72591232	9515	-0.8504717	0.554603	3.10E-184	Loss	Gain
Chr17_CNVR_71	Chr17	72818728	72830838	12111	-0.9097266	0.532286	5.68E-262	Loss	Gain
Chr17_CNVR_72	Chr17	72874088	72888792	14705	-1.160004	0.447511	0	Loss	Gain
Chr17_CNVR_73	Chr17	72899174	72932908	33735	-1.207194	0.43311	0	Loss	Gain
Chr17_CNVR_74	Chr17	72935504	72946748	11245	-1.479551	0.3586	0	Loss	Gain
Chr17_CNVR_75	Chr17	73143968	73152618	8651	-0.8629522	0.549826	7.53E-172	Loss	Gain
Chr17_CNVR_76	Chr17	73153484	73169052	15569	-0.9594266	0.514261	0	Loss	Gain
Chr17_CNVR_77	Chr17	73195868	73207112	11245	-0.9808083	0.506696	1.60E-275	Loss	Gain
Chr17_CNVR_78	Chr17	73207978	73222682	14705	-1.074201	0.474934	0	Loss	Gain
Chr17_CNVR_79	Chr17	73240848	73249498	8651	-1.023135	0.492046	2.06E-227	Loss	Gain
Chr17_CNVR_80	Chr17	73273718	73282368	8651	-1.011232	0.496122	3.50E-223	Loss	Gain
Chr17_CNVR_81	Chr17	73300534	73318698	18165	-0.8382185	0.559334	0	Loss	Gain
Chr17_CNVR_82	Chr17	73344648	73355892	11245	-0.9673165	0.511457	2.29E-269	Loss	Gain
Chr17_CNVR_83	Chr17	73380978	73390492	9515	-1.146575	0.451696	7.56E-299	Loss	Gain
Chr17_CNVR_84	Chr17	73393088	73410388	17301	-0.8394455	0.558858	0	Loss	Gain
Chr17_CNVR_85	Chr17	73429418	73438068	8651	-1.135747	0.455099	6.06E-268	Loss	Gain
Chr17_CNVR_86	Chr17	73440664	73453638	12975	-1.110232	0.46322	0	Loss	Gain
Chr17_CNVR_87	Chr17	73487374	73505538	18165	-0.9827197	0.506025	0	Loss	Gain
Chr17_CNVR_88	Chr17	73513324	73521972	8649	-0.9626464	0.513115	4.64E-206	Loss	Gain
Chr17_CNVR_89	Chr17	73573008	73586848	13841	-0.9844126	0.505431	0	Loss	Gain
Chr17_CNVR_90	Chr17	73588578	73614528	25951	-0.9607678	0.513783	0	Loss	Gain
Chr17_CNVR_91	Chr17	73615394	73624908	9515	-0.8788285	0.543809	1.03E-194	Loss	Gain
Chr17_CNVR_92	Chr17	73626638	73636152	9515	-0.9291954	0.525151	1.16E-213	Loss	Gain
Chr17_CNVR_93	Chr17	73645668	73660372	14705	-0.9407356	0.520967	0	Loss	Gain
Chr17_CNVR_94	Chr17	73661238	73669888	8651	-0.8718817	0.546434	7.45E-175	Loss	Gain
Chr17_CNVR_95	Chr17	73694974	73712272	17299	-1.036945	0.487358	0	Loss	Gain
Chr17_CNVR_96	Chr17	73713138	73753792	40655	-1.052682	0.482071	0	Loss	Gain
Chr17_CNVR_97	Chr17	73754658	73778878	24221	-1.169134	0.444688	0	Loss	Gain
Chr17_CNVR_98	Chr17	73779744	73822128	42385	-1.077676	0.473791	0	Loss	Gain
Chr17_CNVR_99	Chr17	73826454	73846348	19895	-0.9668344	0.511627	0	Loss	Gain
Chr17_CNVR_100	Chr17	73929388	73939768	10381	-0.9158292	0.530039	2.30E-227	Loss	Gain
Chr17_CNVR_101	Chr17	73970908	73982152	11245	-0.9165851	0.529761	1.79E-246	Loss	Gain
Chr17_CNVR_102	Chr17	74015024	74030592	15569	-0.8756263	0.545017	0	Loss	Gain
Chr17_CNVR_103	Chr17	74040974	74079898	38925	-0.9905754	0.503277	0	Loss	Gain
Chr17_CNVR_104	Chr17	74082494	74097198	14705	-0.9944519	0.501927	0	Loss	Gain
Chr17_CNVR_105	Chr17	74106714	74156882	50169	-1.012006	0.495856	0	Loss	Gain
Chr17_CNVR_106	Chr17	74159478	74170722	11245	-1.011871	0.495903	8.64E-290	Loss	Gain

Chr17_CNVR_107	Chr17	74184564	74200132	15569	-1.111961	0.462665	0	Loss	Gain
Chr17_CNVR_108	Chr17	74221758	74239922	18165	-1.057648	0.480415	0	Loss	Gain
Chr17_CNVR_109	Chr17	74241654	74260682	19029	-1.114601	0.461819	0	Loss	Gain
Chr17_CNVR_110	Chr17	74265008	74280578	15571	-1.001215	0.499579	0	Loss	Gain
Chr17_CNVR_111	Chr17	74282308	74309988	27681	-1.012612	0.495648	0	Loss	Gain
Chr17_CNVR_112	Chr17	74311718	74329018	17301	-1.044998	0.484646	0	Loss	Gain
Chr17_CNVR_113	Chr17	74329884	74341128	11245	-0.9313656	0.524362	4.24E-253	Loss	Gain
Chr17_CNVR_114	Chr17	74341994	74359292	17299	-0.8984435	0.536465	0	Loss	Gain
Chr17_CNVR_115	Chr17	74365348	74380052	14705	-0.9307367	0.52459	0	Loss	Gain
Chr17_CNVR_116	Chr17	74380918	74396488	15571	-0.904707	0.534141	0	Loss	Gain
Chr17_CNVR_117	Chr17	74445794	74457038	11245	-1.035144	0.487967	1.48E-300	Loss	Gain
Chr17_CNVR_118	Chr17	74457904	74523642	65739	-0.9753445	0.508618	0	Loss	Gain
Chr17_CNVR_119	Chr17	74525374	74538348	12975	-1.099439	0.466698	0	Loss	Gain
Chr17_CNVR_120	Chr17	74539214	74548728	9515	-0.9511371	0.517225	4.90E-222	Loss	Gain
Chr17_CNVR_121	Chr17	74553918	74577272	23355	-1.008794	0.496962	0	Loss	Gain
Chr17_CNVR_122	Chr17	74578138	74613602	35465	-1.00359	0.498757	0	Loss	Gain
Chr17_CNVR_123	Chr17	74616198	74628308	12111	-0.9843297	0.505461	2.76E-298	Loss	Gain
Chr17_CNVR_124	Chr17	74666368	74684532	18165	-0.9379539	0.521973	0	Loss	Gain
Chr17_CNVR_125	Chr17	74692318	74803038	110721	-0.9977546	0.500779	0	Loss	Gain
Chr17_CNVR_126	Chr17	74823798	74834178	10381	-1.126851	0.457914	0	Loss	Gain
Chr17_CNVR_127	Chr17	74835908	74872238	36331	-0.9606105	0.513839	0	Loss	Gain
Chr17_CNVR_128	Chr17	74873104	74894728	21625	-1.041699	0.485755	0	Loss	Gain
Chr17_CNVR_129	Chr17	74898188	74909432	11245	-1.058376	0.480172	0	Loss	Gain
Chr17_CNVR_130	Chr17	74910298	74944898	34601	-1.116752	0.461131	0	Loss	Gain
Chr17_CNVR_131	Chr17	74946628	75005448	58821	-1.083973	0.471728	0	Loss	Gain
Chr17_CNVR_132	Chr17	75046104	75054752	8649	-1.001121	0.499612	1.34E-219	Loss	Gain
Chr17_CNVR_133	Chr17	75056484	75078972	22489	-1.061435	0.479155	0	Loss	Gain
Chr17_CNVR_134	Chr17	75079838	75101462	21625	-1.023618	0.491881	0	Loss	Gain
Chr17_CNVR_135	Chr17	75102328	75110978	8651	-0.9609134	0.513732	1.87E-205	Loss	Gain
Chr17_CNVR_136	Chr17	75111844	75136928	25085	-0.9916427	0.502905	0	Loss	Gain
Chr18_CNVR_1	Chr18	669296	678755	9460	1.304962	2.47077	0	Gain	Loss
Chr18_CNVR_14	Chr18	11395990	11405449	9460	-0.8692609	0.547427	5.39E-173	Loss	Gain
Chr18_CNVR_15	Chr18	11409234	11422477	13244	-0.9127216	0.531182	1.39E-261	Loss	Gain
Chr18_CNVR_16	Chr18	11429100	11442343	13244	-1.954069	0.258087	0	Loss	Gain
Chr18_CNVR_17	Chr18	11477346	11488697	11352	-0.9171927	0.529538	2.56E-226	Loss	Gain
Chr18_CNVR_18	Chr18	11512348	11521807	9460	-0.8141443	0.568746	4.53E-155	Loss	Gain
Chr18_CNVR_19	Chr18	11558702	11569107	10406	-0.8759254	0.544904	1.66E-192	Loss	Gain
Chr18_CNVR_20	Chr18	13702338	13711797	9460	-0.7762247	0.583893	4.82E-143	Loss	Gain
Chr18_CNVR_21	Chr18	13978570	13988975	10406	-0.8203767	0.566294	1.42E-172	Loss	Gain
Chr18_CNVR_22	Chr18	14098712	14109117	10406	-0.8554788	0.552682	4.15E-185	Loss	Gain
Chr18_CNVR_23	Chr18	14137498	14146957	9460	-0.8365768	0.559971	2.63E-162	Loss	Gain
Chr18_CNVR_2	Chr18	16107070	16116529	9460	0.8025635	1.7442	4.12E-176	Gain	Loss
Chr18_CNVR_3	Chr18	21791584	21804827	13244	1.159011	2.23304	0	Gain	Loss
Chr18_CNVR_4	Chr18	31858916	31868375	9460	0.8414738	1.79188	7.45E-192	Gain	Loss
Chr18_CNVR_5	Chr18	36845282	36855687	10406	0.9250435	1.89874	1.35E-249	Gain	Loss
Chr18_CNVR_6	Chr18	36876500	36888797	12298	0.8462121	1.79777	2.61E-251	Gain	Loss
Chr18_CNVR_24	Chr18	43809734	43833383	23650	-1.916065	0.264976	0	Loss	Gain
Chr18_CNVR_25	Chr18	51751404	51781675	30272	-1.329342	0.39795	0	Loss	Gain
Chr18_CNVR_26	Chr18	56670604	56681955	11352	-0.8412989	0.558141	3.40E-196	Loss	Gain
Chr18_CNVR_7	Chr18	57604306	57707419	103114	1.221442	2.3318	0	Gain	Loss
Chr18_CNVR_8	Chr18	58468004	58485977	17974	1.427596	2.68998	0	Gain	Loss
Chr18_CNVR_9	Chr18	58488816	58499221	10406	0.8405824	1.79077	1.98E-210	Gain	Loss
Chr18_CNVR_10	Chr18	58565442	58581523	16082	1.404098	2.64652	0	Gain	Loss
Chr18_CNVR_11	Chr18	61447904	61463985	16082	4.522634	22.9852	0	Gain	Loss
Chr18_CNVR_27	Chr18	61543450	61552909	9460	-0.8527942	0.553711	1.37E-167	Loss	Gain
Chr18_CNVR_28	Chr18	61652240	61691971	39732	-4.241759	0.0528571	0	Loss	Gain

Chr18_CNVR_12	Chr18	61703324	61719405	16082	2.705465	6.52268	0	Gain	Loss
Chr18_CNVR_13	Chr18	61758192	61770489	12298	3.250822	9.51908	0	Gain	Loss
Chr18_CNVR_29	Chr18	61774274	61784679	10406	-0.8930742	0.538465	9.21E-199	Loss	Gain
Chr18_CNVR_30	Chr18	61791302	61814005	22704	-0.8824755	0.542436	0	Loss	Gain
Chr18_CNVR_31	Chr18	61872658	61884009	11352	-0.9890613	0.503805	1.25E-255	Loss	Gain
Chr18_CNVR_32	Chr18	61891578	61901037	9460	-0.9957791	0.501465	1.09E-215	Loss	Gain
Chr18_CNVR_33	Chr18	61902930	61914281	11352	-0.8937861	0.5382	6.33E-217	Loss	Gain
Chr18_CNVR_34	Chr18	62474314	62485665	11352	-0.8754723	0.545075	1.23E-209	Loss	Gain
Chr18_CNVR_35	Chr18	63215032	63229221	14190	-1.593185	0.331439	0	Loss	Gain
Chr18_CNVR_36	Chr18	63255710	63275575	19866	-1.7797	0.291244	0	Loss	Gain
Chr18_CNVR_37	Chr18	63303010	63329497	26488	-2.369172	0.193557	0	Loss	Gain
Chr18_CNVR_38	Chr18	63331390	63352201	20812	-2.027608	0.245261	0	Loss	Gain
Chr18_CNVR_39	Chr18	63776010	63786415	10406	-1.128933	0.457254	4.30E-288	Loss	Gain
Chr19_CNVR_1	Chr19	275426	285963	10538	0.8177798	1.76269	9.90E-203	Gain	Loss
Chr19_CNVR_2	Chr19	1594592	1604171	9580	0.8589534	1.81372	1.27E-201	Gain	Loss
Chr19_CNVR_3	Chr19	1866664	1876243	9580	1.396523	2.63266	0	Gain	Loss
Chr19_CNVR_4	Chr19	2388774	2399311	10538	0.9291733	1.90418	6.39E-255	Gain	Loss
Chr19_CNVR_5	Chr19	2545886	2558339	12454	0.871699	1.82982	1.69E-268	Gain	Loss
Chr19_CNVR_6	Chr19	2876396	2895555	19160	1.087815	2.12552	0	Gain	Loss
Chr19_CNVR_7	Chr19	18515746	18525325	9580	0.8427763	1.7935	7.87E-195	Gain	Loss
Chr19_CNVR_8	Chr19	18532990	18543527	10538	0.841087	1.7914	2.50E-213	Gain	Loss
Chr19_CNVR_9	Chr19	19074260	19085755	11496	1.066384	2.09418	0	Gain	Loss
Chr19_CNVR_10	Chr19	19123118	19132697	9580	0.977263	1.96873	2.10E-253	Gain	Loss
Chr19_CNVR_11	Chr19	19796592	19871315	74724	1.548484	2.9251	0	Gain	Loss
Chr19_CNVR_12	Chr19	19877064	19889517	12454	1.108945	2.15688	0	Gain	Loss
Chr19_CNVR_13	Chr19	19906762	19917299	10538	1.07448	2.10596	0	Gain	Loss
Chr19_CNVR_14	Chr19	24617248	24635449	18202	0.9671348	1.95495	0	Gain	Loss
Chr19_CNVR_15	Chr19	24769570	24784897	15328	1.2236	2.33529	0	Gain	Loss
Chr19_CNVR_19	Chr19	35274998	35292241	17244	-0.8518913	0.554058	3.09E-299	Loss	Gain
Chr19_CNVR_20	Chr19	35296074	35305653	9580	-0.826817	0.563772	4.64E-159	Loss	Gain
Chr19_CNVR_21	Chr19	35309486	35319065	9580	-0.8125132	0.569389	1.79E-154	Loss	Gain
Chr19_CNVR_16	Chr19	38845464	38855043	9580	0.9169749	1.88815	1.40E-226	Gain	Loss
Chr19_CNVR_22	Chr19	43915200	43927653	12454	-1.337475	0.395713	0	Loss	Gain
Chr19_CNVR_17	Chr19	51063796	51074333	10538	0.8524128	1.80552	1.52E-218	Gain	Loss
Chr19_CNVR_23	Chr19	51860852	51870431	9580	-0.7870094	0.579544	2.18E-146	Loss	Gain
Chr19_CNVR_18	Chr19	52133882	52146335	12454	1.295331	2.45433	0	Gain	Loss
Chr19_CNVR_24	Chr19	54383266	54392845	9580	-0.8239974	0.564875	3.75E-158	Loss	Gain
Chr19_CNVR_25	Chr19	57803326	57815779	12454	-0.8960083	0.537371	1.43E-235	Loss	Gain
Chr2_CNVR_7	Chr2	40270	64518	24249	-0.919146	0.528822	0	Loss	Gain
Chr2_CNVR_8	Chr2	85302	150252	64951	-1.587982	0.332636	0	Loss	Gain
Chr2_CNVR_9	Chr2	4807600	4817126	9527	-0.8431551	0.557423	6.08E-182	Loss	Gain
Chr2_CNVR_10	Chr2	5379160	5393016	13857	-0.9156801	0.530094	2.21E-303	Loss	Gain
Chr2_CNVR_1	Chr2	56706980	56716506	9527	0.9648717	1.95189	1.09E-239	Gain	Loss
Chr2_CNVR_2	Chr2	57408440	57417100	8661	1.211921	2.31646	0	Gain	Loss
Chr2_CNVR_11	Chr2	63493822	63505946	12125	-1.172078	0.443782	0	Loss	Gain
Chr2_CNVR_12	Chr2	72515810	72524470	8661	-0.8593083	0.551217	4.86E-171	Loss	Gain
Chr2_CNVR_13	Chr2	72966996	72975656	8661	-0.8318009	0.561827	8.69E-162	Loss	Gain
Chr2_CNVR_14	Chr2	72993842	73002502	8661	-0.7890473	0.578726	1.06E-147	Loss	Gain
Chr2_CNVR_15	Chr2	73112484	73132402	19919	-0.8058909	0.572009	0	Loss	Gain
Chr2_CNVR_16	Chr2	83461184	83471576	10393	-3.999869	0.0625057	0	Loss	Gain
Chr2_CNVR_17	Chr2	100640026	100653016	12991	-1.362976	0.388779	0	Loss	Gain
Chr2_CNVR_18	Chr2	104880828	104889488	8661	-0.8238842	0.564919	3.76E-159	Loss	Gain
Chr2_CNVR_19	Chr2	104901612	104911138	9527	-0.8495075	0.554974	2.71E-184	Loss	Gain
Chr2_CNVR_20	Chr2	104945778	104957036	11259	-0.9365625	0.522476	2.16E-256	Loss	Gain
Chr2_CNVR_21	Chr2	104970026	104981284	11259	-1.023977	0.491759	6.73E-297	Loss	Gain
Chr2_CNVR_22	Chr2	104983016	104994274	11259	-0.9826678	0.506043	1.30E-277	Loss	Gain

Chr2_CNVR_23	Chr2	104995140	105006398	11259	-0.9180807	0.529213	5.46E-248	Loss	Gain
Chr2_CNVR_24	Chr2	105374448	105385706	11259	-0.8721367	0.546337	2.14E-227	Loss	Gain
Chr2_CNVR_25	Chr2	106624086	106633612	9527	-0.8348086	0.560657	7.25E-179	Loss	Gain
Chr2_CNVR_26	Chr2	106659592	106668252	8661	-1.030414	0.48957	3.31E-231	Loss	Gain
Chr2_CNVR_27	Chr2	106674314	106694232	19919	-0.9266033	0.526096	0	Loss	Gain
Chr2_CNVR_28	Chr2	106704624	106715016	10393	-0.9189943	0.528878	1.85E-229	Loss	Gain
Chr2_CNVR_29	Chr2	106716748	106759182	42435	-0.9406257	0.521007	0	Loss	Gain
Chr2_CNVR_30	Chr2	106815472	106837988	22517	-0.8444351	0.556929	0	Loss	Gain
Chr2_CNVR_31	Chr2	106838854	106848380	9527	-0.9129895	0.531083	3.26E-208	Loss	Gain
Chr2_CNVR_32	Chr2	106857040	106870896	13857	-0.8607851	0.550653	3.33E-273	Loss	Gain
Chr2_CNVR_33	Chr2	106871762	106881288	9527	-0.8316433	0.561889	1.05E-177	Loss	Gain
Chr2_CNVR_34	Chr2	107114242	107126366	12125	-0.808305	0.571052	8.95E-215	Loss	Gain
Chr2_CNVR_35	Chr2	107542912	107551572	8661	-0.8413603	0.558117	5.49E-165	Loss	Gain
Chr2_CNVR_36	Chr2	107650296	107671946	21651	-0.8624715	0.55001	0	Loss	Gain
Chr2_CNVR_37	Chr2	107947334	107955994	8661	-0.7892028	0.578664	9.40E-148	Loss	Gain
Chr2_CNVR_38	Chr2	108072904	108086760	13857	-0.8460695	0.556298	2.97E-265	Loss	Gain
Chr2_CNVR_39	Chr2	108096286	108112740	16455	-0.911194	0.531745	0	Loss	Gain
Chr2_CNVR_40	Chr2	108113606	108143050	29445	-0.9103062	0.532072	0	Loss	Gain
Chr2_CNVR_41	Chr2	108143916	108191546	47631	-1.028389	0.490257	0	Loss	Gain
Chr2_CNVR_42	Chr2	108201938	108217526	15589	-0.9139419	0.530733	0	Loss	Gain
Chr2_CNVR_43	Chr2	108220124	108233114	12991	-0.8711858	0.546697	1.45E-261	Loss	Gain
Chr2_CNVR_44	Chr2	108281610	108290270	8661	-0.7742297	0.584701	6.53E-143	Loss	Gain
Chr2_CNVR_45	Chr2	110788680	110797340	8661	-0.9809441	0.506648	2.01E-213	Loss	Gain
Chr2_CNVR_46	Chr2	120171790	120181316	9527	-0.8494849	0.554983	2.76E-184	Loss	Gain
Chr2_CNVR_47	Chr2	120197770	120209028	11259	-0.9682888	0.511112	5.88E-271	Loss	Gain
Chr2_CNVR_48	Chr2	120210760	120219420	8661	-0.8757517	0.54497	1.23E-176	Loss	Gain
Chr2_CNVR_49	Chr2	120780588	120789248	8661	-0.8872602	0.54064	1.40E-180	Loss	Gain
Chr2_CNVR_50	Chr2	120855930	120873250	17321	-0.8905882	0.539394	0	Loss	Gain
Chr2_CNVR_51	Chr2	120904426	120924344	19919	-0.9311306	0.524447	0	Loss	Gain
Chr2_CNVR_52	Chr2	120934736	120949458	14723	-0.9127735	0.531163	0	Loss	Gain
Chr2_CNVR_53	Chr2	120960716	120978036	17321	-0.9404063	0.521086	0	Loss	Gain
Chr2_CNVR_54	Chr2	120978902	120988428	9527	-0.8606162	0.550717	2.00E-188	Loss	Gain
Chr2_CNVR_55	Chr2	122486608	122498732	12125	-0.8143211	0.568676	1.50E-217	Loss	Gain
Chr2_CNVR_56	Chr2	122500464	122517784	17321	-0.9735952	0.509235	0	Loss	Gain
Chr2_CNVR_57	Chr2	122523846	122544630	20785	-0.8852842	0.541381	0	Loss	Gain
Chr2_CNVR_58	Chr2	122545496	122555022	9527	-0.8917096	0.538975	4.10E-200	Loss	Gain
Chr2_CNVR_59	Chr2	122555888	122574940	19053	-0.9215351	0.527947	0	Loss	Gain
Chr2_CNVR_60	Chr2	122584466	122593992	9527	-0.8361172	0.560149	2.39E-179	Loss	Gain
Chr2_CNVR_61	Chr2	122598322	122612178	13857	-0.8599549	0.55097	9.40E-273	Loss	Gain
Chr2_CNVR_62	Chr2	123255616	123275534	19919	-0.8128634	0.569251	0	Loss	Gain
Chr2_CNVR_63	Chr2	123285060	123298050	12991	-0.8942229	0.538037	2.07E-273	Loss	Gain
Chr2_CNVR_64	Chr2	123299782	123310174	10393	-0.8036972	0.572879	1.42E-182	Loss	Gain
Chr2_CNVR_65	Chr2	123344814	123363000	18187	-0.9019152	0.535176	0	Loss	Gain
Chr2_CNVR_66	Chr2	123399372	123410630	11259	-0.8426114	0.557633	2.09E-214	Loss	Gain
Chr2_CNVR_3	Chr2	124333786	124344178	10393	0.9023204	1.86907	4.52E-233	Gain	Loss
Chr2_CNVR_4	Chr2	124371024	124381416	10393	0.8633748	1.81929	6.11E-216	Gain	Loss
Chr2_CNVR_67	Chr2	124816148	124827406	11259	-0.944862	0.519479	3.48E-260	Loss	Gain
Chr2_CNVR_68	Chr2	126269296	126280554	11259	-0.8198041	0.566519	1.62E-204	Loss	Gain
Chr2_CNVR_69	Chr2	126281420	126290946	9527	-0.8941446	0.538066	4.90E-201	Loss	Gain
Chr2_CNVR_70	Chr2	126291812	126301338	9527	-0.8782499	0.544027	4.95E-195	Loss	Gain
Chr2_CNVR_71	Chr2	126303936	126313462	9527	-1.052172	0.482242	8.47E-263	Loss	Gain
Chr2_CNVR_72	Chr2	126314328	126326452	12125	-0.9258214	0.526381	1.11E-270	Loss	Gain
Chr2_CNVR_73	Chr2	126327318	126335978	8661	-0.9145696	0.530502	5.04E-190	Loss	Gain
Chr2_CNVR_74	Chr2	126346370	126355896	9527	-0.9658794	0.511966	1.17E-228	Loss	Gain
Chr2_CNVR_75	Chr2	126510910	126527364	16455	-0.916385	0.529835	0	Loss	Gain
Chr2_CNVR_76	Chr2	126529962	126542952	12991	-0.8835612	0.542028	6.53E-268	Loss	Gain

Chr2_CNVR_77	Chr2	126802752	126814010	11259	-0.8815684	0.542777	1.38E-231	Loss	Gain
Chr2_CNVR_78	Chr2	126930054	126938714	8661	-0.857548	0.55189	1.92E-170	Loss	Gain
Chr2_CNVR_79	Chr2	127849746	127858406	8661	-0.7869706	0.57956	4.99E-147	Loss	Gain
Chr2_CNVR_80	Chr2	127880056	127897376	17321	-0.9384793	0.521783	0	Loss	Gain
Chr2_CNVR_81	Chr2	127899108	127913830	14723	-0.9381529	0.521901	0	Loss	Gain
Chr2_CNVR_82	Chr2	127914696	127925954	11259	-0.8562351	0.552392	2.25E-220	Loss	Gain
Chr2_CNVR_83	Chr2	128057586	128067112	9527	-0.8710276	0.546757	2.56E-192	Loss	Gain
Chr2_CNVR_84	Chr2	128078370	128093958	15589	-0.8941711	0.538056	0	Loss	Gain
Chr2_CNVR_85	Chr2	128493184	128502710	9527	-0.821384	0.565899	5.99E-174	Loss	Gain
Chr2_CNVR_86	Chr2	128550340	128560732	10393	-0.9014716	0.53534	3.71E-222	Loss	Gain
Chr2_CNVR_87	Chr2	128566794	128577186	10393	-0.8550821	0.552834	4.07E-203	Loss	Gain
Chr2_CNVR_88	Chr2	128584980	128601434	16455	-0.9576196	0.514906	0	Loss	Gain
Chr2_CNVR_89	Chr2	128611826	128621352	9527	-0.8914478	0.539073	5.15E-200	Loss	Gain
Chr2_CNVR_90	Chr2	128639538	128649930	10393	-0.9631388	0.51294	4.37E-248	Loss	Gain
Chr2_CNVR_91	Chr2	128651662	128661188	9527	-0.8909067	0.539275	8.26E-200	Loss	Gain
Chr2_CNVR_92	Chr2	129169530	129179056	9527	-0.8562515	0.552386	8.47E-187	Loss	Gain
Chr2_CNVR_93	Chr2	129392958	129401618	8661	-0.8979567	0.536646	2.89E-184	Loss	Gain
Chr2_CNVR_94	Chr2	129887444	129897836	10393	-0.8621382	0.550137	5.52E-206	Loss	Gain
Chr2_CNVR_95	Chr2	129991364	130000890	9527	-0.8211101	0.566006	7.54E-174	Loss	Gain
Chr2_CNVR_96	Chr2	130524820	130533480	8661	-0.7868486	0.579609	5.47E-147	Loss	Gain
Chr2_CNVR_97	Chr2	130538676	130555996	17321	-0.8709632	0.546782	0	Loss	Gain
Chr2_CNVR_98	Chr2	130556862	130577646	20785	-0.9243776	0.526908	0	Loss	Gain
Chr2_CNVR_99	Chr2	130606224	130634802	28579	-1.030385	0.489579	0	Loss	Gain
Chr2_CNVR_100	Chr2	130636534	130651256	14723	-0.9472943	0.518604	0	Loss	Gain
Chr2_CNVR_101	Chr2	130652122	130686762	34641	-0.9257171	0.526419	0	Loss	Gain
Chr2_CNVR_102	Chr2	130719670	130732660	12991	-0.8976439	0.536763	3.52E-275	Loss	Gain
Chr2_CNVR_103	Chr2	130769898	130784620	14723	-0.8365502	0.559981	2.86E-276	Loss	Gain
Chr2_CNVR_104	Chr2	130785486	130797610	12125	-0.9133619	0.530946	1.33E-264	Loss	Gain
Chr2_CNVR_105	Chr2	130806270	130817528	11259	-0.929957	0.524874	2.21E-253	Loss	Gain
Chr2_CNVR_106	Chr2	130818394	130862560	44167	-0.9763084	0.508279	0	Loss	Gain
Chr2_CNVR_107	Chr2	130863426	130873818	10393	-1.038834	0.486721	1.11E-280	Loss	Gain
Chr2_CNVR_108	Chr2	130883344	130902396	19053	-0.9592242	0.514333	0	Loss	Gain
Chr2_CNVR_109	Chr2	131273910	131286900	12991	-0.8931238	0.538447	7.67E-273	Loss	Gain
Chr2_CNVR_110	Chr2	131291230	131300756	9527	-0.9582936	0.514665	1.06E-225	Loss	Gain
Chr2_CNVR_111	Chr2	131467894	131486946	19053	-0.8833014	0.542125	0	Loss	Gain
Chr2_CNVR_112	Chr2	131490410	131517256	26847	-0.8986904	0.536373	0	Loss	Gain
Chr2_CNVR_113	Chr2	131518122	131550164	32043	-0.9877914	0.504249	0	Loss	Gain
Chr2_CNVR_114	Chr2	131570082	131578742	8661	-1.029643	0.489831	6.30E-231	Loss	Gain
Chr2_CNVR_115	Chr2	131723364	131767530	44167	-0.9716813	0.509911	0	Loss	Gain
Chr2_CNVR_116	Chr2	131768396	131777056	8661	-0.9644132	0.512487	1.53E-207	Loss	Gain
Chr2_CNVR_117	Chr2	131778788	131788314	9527	-1.063129	0.478593	3.45E-267	Loss	Gain
Chr2_CNVR_118	Chr2	131789180	131808232	19053	-0.9390025	0.521593	0	Loss	Gain
Chr2_CNVR_119	Chr2	131814294	131824686	10393	-0.8571882	0.552027	5.69E-204	Loss	Gain
Chr2_CNVR_120	Chr2	131932936	131945926	12991	-0.8492644	0.555068	2.01E-250	Loss	Gain
Chr2_CNVR_121	Chr2	131966710	131979700	12991	-0.9325941	0.523915	2.04E-293	Loss	Gain
Chr2_CNVR_122	Chr2	132038588	132048980	10393	-0.8170663	0.567595	7.38E-188	Loss	Gain
Chr2_CNVR_123	Chr2	132551260	132567714	16455	-0.9298366	0.524918	0	Loss	Gain
Chr2_CNVR_124	Chr2	132578106	132588498	10393	-0.8793811	0.543601	4.90E-213	Loss	Gain
Chr2_CNVR_125	Chr2	132589364	132598024	8661	-1.009417	0.496747	1.26E-223	Loss	Gain
Chr2_CNVR_126	Chr2	132600622	132617076	16455	-0.8748833	0.545298	0	Loss	Gain
Chr2_CNVR_127	Chr2	132740914	132753038	12125	-0.9017348	0.535243	5.88E-259	Loss	Gain
Chr2_CNVR_128	Chr2	132766028	132777286	11259	-0.8717766	0.546473	3.09E-227	Loss	Gain
Chr2_CNVR_129	Chr2	132781616	132796338	14723	-0.887011	0.540733	2.34E-305	Loss	Gain
Chr2_CNVR_130	Chr2	132817988	132827514	9527	-0.8165668	0.567792	3.41E-172	Loss	Gain
Chr2_CNVR_131	Chr2	132846566	132855226	8661	-0.9190009	0.528875	1.44E-191	Loss	Gain
Chr2_CNVR_132	Chr2	132876010	132887268	11259	-0.810495	0.570186	1.61E-200	Loss	Gain

Chr2_CNVR_133	Chr2	132914980	132938362	23383	-0.9500735	0.517606	0	Loss	Gain
Chr2_CNVR_134	Chr2	133283896	133296886	12991	-1.361209	0.389256	0	Loss	Gain
Chr2_CNVR_135	Chr2	133530706	133567944	37239	-0.9642155	0.512557	0	Loss	Gain
Chr2_CNVR_136	Chr2	133568810	133586130	17321	-0.8750274	0.545244	0	Loss	Gain
Chr2_CNVR_137	Chr2	133612976	133628564	15589	-0.9177116	0.529348	0	Loss	Gain
Chr2_CNVR_138	Chr2	133638956	133655410	16455	-0.8451338	0.556659	0	Loss	Gain
Chr2_CNVR_139	Chr2	133803496	133820816	17321	-0.9452072	0.519355	0	Loss	Gain
Chr2_CNVR_140	Chr2	133833806	133869312	35507	-1.040674	0.4861	0	Loss	Gain
Chr2_CNVR_141	Chr2	133871044	133903086	32043	-1.040826	0.486049	0	Loss	Gain
Chr2_CNVR_142	Chr2	133903952	133918674	14723	-0.9891435	0.503777	0	Loss	Gain
Chr2_CNVR_143	Chr2	133919540	133928200	8661	-0.9477128	0.518454	1.23E-201	Loss	Gain
Chr2_CNVR_144	Chr2	133929932	133946386	16455	-0.9757564	0.508473	0	Loss	Gain
Chr2_CNVR_145	Chr2	133947252	133959376	12125	-0.9421479	0.520457	1.07E-278	Loss	Gain
Chr2_CNVR_146	Chr2	133975830	133985356	9527	-0.844041	0.557081	2.86E-182	Loss	Gain
Chr2_CNVR_147	Chr2	133986222	133996614	10393	-0.9435454	0.519954	8.86E-240	Loss	Gain
Chr2_CNVR_148	Chr2	134134308	134153360	19053	-0.849693	0.554903	0	Loss	Gain
Chr2_CNVR_149	Chr2	134155092	134168082	12991	-1.003845	0.498669	0	Loss	Gain
Chr2_CNVR_150	Chr2	134171546	134184536	12991	-0.9867667	0.504607	0	Loss	Gain
Chr2_CNVR_151	Chr2	134196660	134205320	8661	-0.8603955	0.550802	2.08E-171	Loss	Gain
Chr2_CNVR_152	Chr2	134274600	134289322	14723	-0.8845078	0.541672	6.76E-304	Loss	Gain
Chr2_CNVR_153	Chr2	134304044	134336086	32043	-1.074341	0.474888	0	Loss	Gain
Chr2_CNVR_154	Chr2	134337818	134360334	22517	-0.9605091	0.513876	0	Loss	Gain
Chr2_CNVR_155	Chr2	134361200	134375922	14723	-1.09268	0.46889	0	Loss	Gain
Chr2_CNVR_156	Chr2	134380252	134391510	11259	-0.9686485	0.510985	4.01E-271	Loss	Gain
Chr2_CNVR_157	Chr2	134401902	134433944	32043	-0.9669809	0.511576	0	Loss	Gain
Chr2_CNVR_158	Chr2	134436542	134450398	13857	-0.9705059	0.510327	0	Loss	Gain
Chr2_CNVR_159	Chr2	134472048	134487636	15589	-0.8206115	0.566202	7.66E-283	Loss	Gain
Chr2_CNVR_160	Chr2	134505822	134517080	11259	-0.8742986	0.545519	2.35E-228	Loss	Gain
Chr2_CNVR_161	Chr2	134517946	134536998	19053	-0.9360705	0.522655	0	Loss	Gain
Chr2_CNVR_162	Chr2	134537864	134547390	9527	-1.062231	0.478891	7.90E-267	Loss	Gain
Chr2_CNVR_163	Chr2	134548256	134589824	41569	-1.03927	0.486574	0	Loss	Gain
Chr2_CNVR_164	Chr2	134590690	134603680	12991	-0.9492841	0.517889	3.22E-302	Loss	Gain
Chr2_CNVR_165	Chr2	134617536	134656506	38971	-0.9706062	0.510292	0	Loss	Gain
Chr2_CNVR_166	Chr2	134673826	134683352	9527	-0.8682628	0.547806	2.78E-191	Loss	Gain
Chr2_CNVR_167	Chr2	134684218	134696342	12125	-0.870303	0.547032	7.54E-244	Loss	Gain
Chr2_CNVR_168	Chr2	134704136	134718858	14723	-0.8002325	0.574257	7.68E-256	Loss	Gain
Chr2_CNVR_169	Chr2	134804592	134817582	12991	-0.879864	0.543419	5.19E-266	Loss	Gain
Chr2_CNVR_170	Chr2	134821046	134833170	12125	-0.9947347	0.501828	7.69E-305	Loss	Gain
Chr2_CNVR_171	Chr2	134834902	134859150	24249	-0.9424085	0.520363	0	Loss	Gain
Chr2_CNVR_172	Chr2	134863480	134932760	69281	-0.9737226	0.50919	0	Loss	Gain
Chr2_CNVR_173	Chr2	134945750	134992514	46765	-0.9474179	0.51856	0	Loss	Gain
Chr2_CNVR_174	Chr2	134995112	135035814	40703	-0.8859416	0.541134	0	Loss	Gain
Chr2_CNVR_175	Chr2	135042742	135053134	10393	-0.8629615	0.549823	2.55E-206	Loss	Gain
Chr2_CNVR_176	Chr2	135054000	135062660	8661	-0.9348741	0.523088	4.05E-197	Loss	Gain
Chr2_CNVR_177	Chr2	135075650	135092970	17321	-0.9421779	0.520447	0	Loss	Gain
Chr2_CNVR_178	Chr2	135093836	135103362	9527	-0.9150825	0.530314	5.16E-209	Loss	Gain
Chr2_CNVR_179	Chr2	135107692	135126744	19053	-0.943729	0.519887	0	Loss	Gain
Chr2_CNVR_180	Chr2	135162250	135175240	12991	-0.8722603	0.54629	4.09E-262	Loss	Gain
Chr2_CNVR_181	Chr2	135176972	135186498	9527	-0.8870005	0.540737	2.48E-198	Loss	Gain
Chr2_CNVR_182	Chr2	135204684	135215076	10393	-0.8638022	0.549502	1.16E-206	Loss	Gain
Chr2_CNVR_183	Chr2	135229798	135249716	19919	-0.9584535	0.514608	0	Loss	Gain
Chr2_CNVR_184	Chr2	135250582	135279160	28579	-0.9364596	0.522514	0	Loss	Gain
Chr2_CNVR_185	Chr2	135280026	135289552	9527	-0.9071424	0.53324	5.58E-206	Loss	Gain
Chr2_CNVR_186	Chr2	135294748	135303408	8661	-0.9202391	0.528421	5.34E-192	Loss	Gain
Chr2_CNVR_187	Chr2	135314666	135334584	19919	-0.9323536	0.524003	0	Loss	Gain
Chr2_CNVR_188	Chr2	135335450	135344110	8661	-0.9411223	0.520828	2.58E-199	Loss	Gain

Chr2_CNVR_189	Chr2	135344976	135354502	9527	-0.9401207	0.521189	1.20E-218	Loss	Gain
Chr2_CNVR_190	Chr2	135357100	135370956	13857	-0.9028403	0.534833	2.97E-296	Loss	Gain
Chr2_CNVR_191	Chr2	135371822	135386544	14723	-0.8890546	0.539968	1.50E-306	Loss	Gain
Chr2_CNVR_192	Chr2	135394338	135414256	19919	-0.9493626	0.517861	0	Loss	Gain
Chr2_CNVR_193	Chr2	135467082	135497392	30311	-0.9969194	0.501069	0	Loss	Gain
Chr2_CNVR_194	Chr2	135506052	135517310	11259	-0.9155325	0.530148	7.77E-247	Loss	Gain
Chr2_CNVR_195	Chr2	135518176	135602178	84003	-1.061159	0.479247	0	Loss	Gain
Chr2_CNVR_196	Chr2	135603910	135644612	40703	-1.104122	0.465185	0	Loss	Gain
Chr2_CNVR_197	Chr2	135645478	135761522	116045	-1.071063	0.475968	0	Loss	Gain
Chr2_CNVR_198	Chr2	135786636	135838596	51961	-1.055325	0.481189	0	Loss	Gain
Chr2_CNVR_199	Chr2	135839462	135851586	12125	-1.003407	0.498821	0	Loss	Gain
Chr2_CNVR_200	Chr2	135852452	135867174	14723	-0.9049428	0.534054	0	Loss	Gain
Chr2_CNVR_201	Chr2	135881030	135900948	19919	-0.9589607	0.514427	0	Loss	Gain
Chr2_CNVR_202	Chr2	135906144	135924330	18187	-0.9814787	0.50646	0	Loss	Gain
Chr2_CNVR_203	Chr2	135926928	135953774	26847	-0.9723377	0.50968	0	Loss	Gain
Chr2_CNVR_204	Chr2	135962434	135972826	10393	-0.9790952	0.507298	6.81E-255	Loss	Gain
Chr2_CNVR_205	Chr2	136000538	136018724	18187	-0.8712052	0.54669	0	Loss	Gain
Chr2_CNVR_206	Chr2	136042972	136067220	24249	-0.8663285	0.548541	0	Loss	Gain
Chr2_CNVR_207	Chr2	136073282	136103592	30311	-0.9723485	0.509676	0	Loss	Gain
Chr2_CNVR_208	Chr2	136104458	136113984	9527	-0.8555016	0.552673	1.61E-186	Loss	Gain
Chr2_CNVR_209	Chr2	136115716	136125242	9527	-0.9547582	0.515928	2.52E-224	Loss	Gain
Chr2_CNVR_210	Chr2	136129572	136142562	12991	-0.8559159	0.552514	8.64E-254	Loss	Gain
Chr2_CNVR_211	Chr2	136149490	136190192	40703	-1.04657	0.484118	0	Loss	Gain
Chr2_CNVR_212	Chr2	136196254	136238688	42435	-0.9873883	0.50439	0	Loss	Gain
Chr2_CNVR_213	Chr2	136239554	136259472	19919	-0.9135779	0.530867	0	Loss	Gain
Chr2_CNVR_214	Chr2	136312298	136321824	9527	-0.8363113	0.560074	2.03E-179	Loss	Gain
Chr2_CNVR_215	Chr2	136439600	136449126	9527	-0.8903892	0.539469	1.30E-199	Loss	Gain
Chr2_CNVR_216	Chr2	136451724	136467312	15589	-0.9083912	0.532779	0	Loss	Gain
Chr2_CNVR_217	Chr2	136468178	136482034	13857	-0.9120741	0.531421	2.24E-301	Loss	Gain
Chr2_CNVR_218	Chr2	136482900	136515808	32909	-0.9734175	0.509298	0	Loss	Gain
Chr2_CNVR_219	Chr2	136517540	136554778	37239	-1.005292	0.498169	0	Loss	Gain
Chr2_CNVR_220	Chr2	136556510	136572098	15589	-0.9301081	0.524819	0	Loss	Gain
Chr2_CNVR_221	Chr2	136573830	136611934	38105	-0.9827903	0.506	0	Loss	Gain
Chr2_CNVR_222	Chr2	136612800	136626656	13857	-1.006375	0.497795	0	Loss	Gain
Chr2_CNVR_223	Chr2	136637048	136656966	19919	-0.8953256	0.537626	0	Loss	Gain
Chr2_CNVR_224	Chr2	136658698	136669090	10393	-0.8938956	0.538159	5.10E-219	Loss	Gain
Chr2_CNVR_225	Chr2	136726246	136735772	9527	-0.8590684	0.551308	7.57E-188	Loss	Gain
Chr2_CNVR_226	Chr2	136811980	136827568	15589	-1.362143	0.389004	0	Loss	Gain
Chr2_CNVR_227	Chr2	136910704	136921096	10393	-1.435321	0.369765	0	Loss	Gain
Chr2_CNVR_228	Chr2	136972190	136982582	10393	-1.272312	0.413996	0	Loss	Gain
Chr2_CNVR_5	Chr2	136994706	137020686	25981	2.851376	7.21688	0	Gain	Loss
Chr2_CNVR_6	Chr2	137043202	137057058	13857	2.907879	7.50514	0	Gain	Loss
Chr20_CNVR_3	Chr20	429292	438938	9647	-0.8194912	0.566642	3.63E-173	Loss	Gain
Chr20_CNVR_4	Chr20	465250	474018	8769	-0.8570541	0.552079	3.80E-170	Loss	Gain
Chr20_CNVR_1	Chr20	16924786	16934432	9647	0.8854109	1.84729	1.03E-209	Gain	Loss
Chr20_CNVR_2	Chr20	44576596	44586242	9647	1.170145	2.25034	0	Gain	Loss
Chr20_CNVR_5	Chr20	58788380	58797150	8771	-0.8066519	0.571707	2.18E-153	Loss	Gain
Chr20_CNVR_6	Chr20	62334968	62350754	15787	-0.8557299	0.552586	9.00E-304	Loss	Gain
Chr20_CNVR_7	Chr20	66577018	66597188	20171	-0.915351	0.530215	0	Loss	Gain
Chr20_CNVR_8	Chr20	66769958	66778726	8769	-0.7954935	0.576146	9.58E-150	Loss	Gain
Chr20_CNVR_9	Chr20	66956758	66968158	11401	-0.8147743	0.568497	2.97E-202	Loss	Gain
Chr20_CNVR_10	Chr20	69434284	69443052	8769	-0.865393	0.548897	5.79E-173	Loss	Gain
Chr20_CNVR_11	Chr20	69455332	69472870	17539	-0.8748678	0.545304	0	Loss	Gain
Chr20_CNVR_12	Chr20	69505320	69518474	13155	-0.8373795	0.559659	2.80E-244	Loss	Gain
Chr20_CNVR_13	Chr20	70253402	70263924	10523	-0.9529045	0.516591	2.04E-243	Loss	Gain
Chr20_CNVR_14	Chr20	70584030	70599816	15787	-0.9422982	0.520403	0	Loss	Gain

Chr20_CNVR_15	Chr20	70600694	70609462	8769	-0.8761929	0.544803	1.25E-176	Loss	Gain
Chr20_CNVR_16	Chr20	70869932	70878702	8771	-0.9388824	0.521637	2.80E-198	Loss	Gain
Chr20_CNVR_17	Chr20	70897996	70942722	44727	-0.9521621	0.516857	0	Loss	Gain
Chr20_CNVR_18	Chr20	70944478	70979556	35079	-1.097531	0.467316	0	Loss	Gain
Chr20_CNVR_19	Chr20	70980434	71012006	31573	-1.012463	0.495699	0	Loss	Gain
Chr20_CNVR_20	Chr20	71013760	71033930	20171	-0.9413561	0.520743	0	Loss	Gain
Chr20_CNVR_21	Chr20	71044456	71081288	36833	-0.9579725	0.51478	0	Loss	Gain
Chr20_CNVR_22	Chr20	71090936	71143556	52621	-1.035859	0.487725	0	Loss	Gain
Chr20_CNVR_23	Chr20	71144434	71179512	35079	-1.054925	0.481322	0	Loss	Gain
Chr20_CNVR_24	Chr20	71180390	71201438	21049	-1.088889	0.470123	0	Loss	Gain
Chr20_CNVR_25	Chr20	71202316	71262828	60513	-1.024912	0.49144	0	Loss	Gain
Chr20_CNVR_26	Chr20	71276860	71308432	31573	-1.074525	0.474827	0	Loss	Gain
Chr20_CNVR_27	Chr20	71318080	71354912	36833	-1.025295	0.49131	0	Loss	Gain
Chr20_CNVR_28	Chr20	71355790	71379468	23679	-1.004687	0.498378	0	Loss	Gain
Chr20_CNVR_29	Chr20	71381224	71422442	41219	-1.038383	0.486873	0	Loss	Gain
Chr20_CNVR_30	Chr20	71423320	71446998	23679	-1.192259	0.437617	0	Loss	Gain
Chr20_CNVR_31	Chr20	71448752	71472430	23679	-1.045977	0.484317	0	Loss	Gain
Chr20_CNVR_32	Chr20	71474186	71486462	12277	-0.9729819	0.509452	1.52E-293	Loss	Gain
Chr20_CNVR_33	Chr20	71488218	71518034	29817	-0.8860998	0.541075	0	Loss	Gain
Chr20_CNVR_34	Chr20	71557500	71567146	9647	-0.8903617	0.539479	2.07E-199	Loss	Gain
Chr20_CNVR_35	Chr20	71612752	71622398	9647	-0.8836483	0.541995	6.96E-197	Loss	Gain
Chr20_CNVR_36	Chr20	71687296	71699574	12279	-0.8408878	0.5583	8.95E-230	Loss	Gain
Chr20_CNVR_37	Chr20	71703082	71712728	9647	-0.8873555	0.540604	2.81E-198	Loss	Gain
Chr20_CNVR_38	Chr20	71759210	71768856	9647	-0.807646	0.571313	7.01E-169	Loss	Gain
Chr21_CNVR_7	Chr21	5769965	5783209	13245	-0.9201439	0.528456	2.71E-285	Loss	Gain
Chr21_CNVR_8	Chr21	9610131	9641035	30905	-1.586218	0.333043	0	Loss	Gain
Chr21_CNVR_9	Chr21	20088693	20109883	21191	-1.236107	0.424517	0	Loss	Gain
Chr21_CNVR_10	Chr21	20125779	20142555	16777	-1.180888	0.44108	0	Loss	Gain
Chr21_CNVR_11	Chr21	20564629	20574341	9713	-1.224455	0.427959	0	Loss	Gain
Chr21_CNVR_12	Chr21	33295723	33305435	9713	-0.794853	0.576402	5.50E-164	Loss	Gain
Chr21_CNVR_13	Chr21	33308085	33316915	8831	-0.9205317	0.528314	4.92E-191	Loss	Gain
Chr21_CNVR_14	Chr21	33518239	33529717	11479	-0.8381637	0.559355	9.59E-212	Loss	Gain
Chr21_CNVR_15	Chr21	33549145	33557973	8829	-0.8071323	0.571517	3.18E-153	Loss	Gain
Chr21_CNVR_16	Chr21	33595943	33604773	8831	-0.9697801	0.510584	4.97E-208	Loss	Gain
Chr21_CNVR_17	Chr21	34266141	34275853	9713	-0.7937792	0.576831	1.32E-163	Loss	Gain
Chr21_CNVR_1	Chr21	37111167	37124411	13245	1.088522	2.12656	0	Gain	Loss
Chr21_CNVR_2	Chr21	37127061	37141187	14127	1.040653	2.05716	0	Gain	Loss
Chr21_CNVR_18	Chr21	37430813	37442291	11479	-6.637386	0.0100449	0	Loss	Gain
Chr21_CNVR_3	Chr21	40146037	40156633	10597	0.9657535	1.95308	3.01E-278	Gain	Loss
Chr21_CNVR_4	Chr21	49704513	49717757	13245	0.8573897	1.81176	4.56E-282	Gain	Loss
Chr21_CNVR_5	Chr21	53532317	53545561	13245	0.9650992	1.9522	0	Gain	Loss
Chr21_CNVR_6	Chr21	53550861	53560573	9713	0.8754435	1.83457	3.46E-215	Gain	Loss
Chr21_CNVR_19	Chr21	62490353	62499181	8829	-2.64866	0.159468	0	Loss	Gain
Chr21_CNVR_20	Chr21	65884605	65893433	8829	-0.8137901	0.568885	2.21E-155	Loss	Gain
Chr21_CNVR_21	Chr21	66181293	66193653	12361	-0.8492353	0.555079	5.46E-233	Loss	Gain
Chr21_CNVR_22	Chr21	66219261	66231623	12363	-0.8803349	0.543241	1.61E-247	Loss	Gain
Chr21_CNVR_23	Chr21	66266061	66280187	14127	-0.8806814	0.543111	9.29E-283	Loss	Gain
Chr21_CNVR_24	Chr21	66319041	66328753	9713	-0.8712668	0.546667	1.39E-191	Loss	Gain
Chr21_CNVR_25	Chr21	66703145	66712857	9713	-0.8575129	0.551903	1.56E-186	Loss	Gain
Chr21_CNVR_26	Chr21	66727869	66740231	12363	-0.9254372	0.526521	5.92E-269	Loss	Gain
Chr21_CNVR_27	Chr21	66958333	66968045	9713	-1.000734	0.499746	1.60E-240	Loss	Gain
Chr21_CNVR_28	Chr21	67088133	67098729	10597	-0.8838704	0.541912	6.87E-214	Loss	Gain
Chr21_CNVR_29	Chr21	67232063	67240891	8829	-0.8406673	0.558385	3.49E-164	Loss	Gain
Chr21_CNVR_30	Chr21	68884155	68898283	14129	-0.8345479	0.560759	3.24E-258	Loss	Gain
Chr21_CNVR_31	Chr21	70336691	70353467	16777	-1.00895	0.496908	0	Loss	Gain
Chr21_CNVR_32	Chr21	70362297	70376425	14129	-0.9709414	0.510173	0	Loss	Gain

Chr21_CNVR_33	Chr21	70396735	70407329	10595	-0.9919658	0.502792	1.66E-258	Loss	Gain
Chr21_CNVR_34	Chr21	70440885	70452363	11479	-0.9103965	0.532039	3.23E-243	Loss	Gain
Chr21_CNVR_35	Chr21	70817043	70834701	17659	-0.9893341	0.50371	0	Loss	Gain
Chr21_CNVR_36	Chr21	70861193	70884149	22957	-0.9253614	0.526549	0	Loss	Gain
Chr21_CNVR_37	Chr21	70923003	70936247	13245	-0.862745	0.549905	3.26E-256	Loss	Gain
Chr21_CNVR_38	Chr21	70954791	70983045	28255	-0.8909734	0.53925	0	Loss	Gain
Chr21_CNVR_39	Chr21	71121677	71141985	20309	-0.8730821	0.545979	0	Loss	Gain
Chr21_CNVR_40	Chr21	71148167	71162295	14129	-0.9694612	0.510697	0	Loss	Gain
Chr21_CNVR_41	Chr21	71185253	71194083	8831	-0.94639	0.518929	6.37E-200	Loss	Gain
Chr21_CNVR_42	Chr21	71194967	71207327	12361	-0.8976403	0.536764	1.07E-255	Loss	Gain
Chr21_CNVR_43	Chr21	71221457	71232935	11479	-0.955182	0.515777	3.24E-263	Loss	Gain
Chr21_CNVR_44	Chr21	71233819	71245297	11479	-0.961491	0.513526	4.66E-266	Loss	Gain
Chr21_CNVR_45	Chr21	71268255	71277085	8831	-0.934494	0.523226	8.01E-196	Loss	Gain
Chr21_CNVR_46	Chr21	71312405	71334479	22075	-0.9614165	0.513552	0	Loss	Gain
Chr21_CNVR_47	Chr21	71341545	71356555	15011	-1.040217	0.486254	0	Loss	Gain
Chr21_CNVR_48	Chr21	71397173	71407769	10597	-0.8636828	0.549548	9.07E-206	Loss	Gain
Chr21_CNVR_49	Chr21	71408653	71421013	12361	-0.8496181	0.554932	3.63E-233	Loss	Gain
Chr21_CNVR_50	Chr21	71442207	71455451	13245	-1.102038	0.465858	0	Loss	Gain
Chr21_CNVR_51	Chr21	71505783	71517261	11479	-0.8740536	0.545612	3.00E-227	Loss	Gain
Chr22_CNVR_1	Chr22	22883536	22894988	11453	-3.414036	0.0938151	0	Loss	Gain
Chr22_CNVR_2	Chr22	50407738	50420070	12333	-0.8455369	0.556504	4.10E-231	Loss	Gain
Chr22_CNVR_3	Chr22	50428882	50441214	12333	-0.9973062	0.500934	1.83E-303	Loss	Gain
Chr22_CNVR_4	Chr22	50448264	50457072	8809	-0.8774003	0.544347	2.88E-176	Loss	Gain
Chr22_CNVR_5	Chr22	50513458	50525790	12333	-0.9271119	0.52591	1.75E-269	Loss	Gain
Chr22_CNVR_6	Chr22	50534602	50548696	14095	-0.8566599	0.55223	1.05E-269	Loss	Gain
Chr22_CNVR_7	Chr22	50559270	50568960	9691	-0.8089038	0.570815	7.04E-169	Loss	Gain
Chr22_CNVR_8	Chr22	50569842	50578650	8809	-0.84938	0.555023	6.06E-167	Loss	Gain
Chr22_CNVR_9	Chr22	50583056	50591866	8811	-0.8588145	0.551405	4.55E-170	Loss	Gain
Chr22_CNVR_10	Chr22	50682610	50700228	17619	-0.8877567	0.540454	0	Loss	Gain
Chr22_CNVR_11	Chr22	50711682	50729302	17621	-0.9485457	0.518155	0	Loss	Gain
Chr22_CNVR_12	Chr22	51808528	51819980	11453	-0.9009267	0.535543	8.19E-239	Loss	Gain
Chr22_CNVR_13	Chr22	56790582	56801154	10573	-0.8838448	0.541921	1.07E-213	Loss	Gain
Chr22_CNVR_14	Chr22	58338500	58347308	8809	-0.8375321	0.5596	4.85E-163	Loss	Gain
Chr22_CNVR_15	Chr22	58367572	58379906	12335	-0.9108278	0.53188	1.02E-261	Loss	Gain
Chr22_CNVR_16	Chr22	58523510	58538486	14977	-0.9580097	0.514767	0	Loss	Gain
Chr22_CNVR_17	Chr22	58553464	58579012	25549	-0.8585403	0.55151	0	Loss	Gain
Chr22_CNVR_18	Chr22	58581656	58598394	16739	-0.9497685	0.517716	0	Loss	Gain
Chr22_CNVR_19	Chr22	58891768	58903220	11453	-0.8007113	0.574066	8.94E-196	Loss	Gain
Chr22_CNVR_20	Chr22	58948152	58964008	15857	-0.8405728	0.558422	1.37E-293	Loss	Gain
Chr22_CNVR_21	Chr22	58984272	58997486	13215	-0.9398876	0.521273	3.84E-295	Loss	Gain
Chr22_CNVR_22	Chr22	59016870	59032726	15857	-0.9132609	0.530984	0	Loss	Gain
Chr22_CNVR_23	Chr22	59039776	59064442	24667	-0.9461296	0.519023	0	Loss	Gain
Chr22_CNVR_24	Chr22	59160472	59173686	13215	-0.8494063	0.555013	2.20E-249	Loss	Gain
Chr22_CNVR_25	Chr22	59174568	59195712	21145	-0.874901	0.545291	0	Loss	Gain
Chr22_CNVR_26	Chr22	59197474	59206284	8811	-0.808249	0.571075	1.68E-153	Loss	Gain
Chr22_CNVR_27	Chr22	59207166	59223904	16739	-0.917605	0.529387	0	Loss	Gain
Chr22_CNVR_28	Chr22	59225666	59251214	25549	-0.9724213	0.50965	0	Loss	Gain
Chr22_CNVR_29	Chr22	59264430	59290860	26431	-0.9583141	0.514658	0	Loss	Gain
Chr22_CNVR_30	Chr22	59313766	59324338	10573	-0.9235018	0.527228	8.22E-230	Loss	Gain
Chr22_CNVR_31	Chr22	59354292	59364864	10573	-0.9219092	0.52781	3.69E-229	Loss	Gain
Chr22_CNVR_32	Chr22	59375436	59398342	22907	-0.9096485	0.532315	0	Loss	Gain
Chr22_CNVR_33	Chr22	59400104	59414200	14097	-0.9181478	0.529188	8.86E-303	Loss	Gain
Chr22_CNVR_34	Chr22	59424772	59452082	27311	-1.016015	0.49448	0	Loss	Gain
Chr22_CNVR_35	Chr22	59465298	59480274	14977	-1.002246	0.499222	0	Loss	Gain
Chr22_CNVR_36	Chr22	59481156	59496132	14977	-0.9785504	0.507489	0	Loss	Gain
Chr22_CNVR_37	Chr22	59497014	59505824	8811	-0.8253635	0.56434	4.67E-159	Loss	Gain

Chr22_CNVR_38	Chr22	59507586	59516396	8811	-0.9707687	0.510234	3.95E-208	Loss	Gain
Chr22_CNVR_39	Chr22	59526088	59548112	22025	-0.9500625	0.51761	0	Loss	Gain
Chr22_CNVR_40	Chr22	59697002	59709334	12333	-0.948763	0.518076	6.99E-280	Loss	Gain
Chr22_CNVR_41	Chr22	59963944	59972754	8811	-0.9295465	0.525023	6.33E-194	Loss	Gain
Chr22_CNVR_42	Chr22	60019448	60033542	14095	-0.9979392	0.500715	0	Loss	Gain
Chr22_CNVR_43	Chr22	60042354	60059972	17619	-1.034365	0.488231	0	Loss	Gain
Chr22_CNVR_44	Chr22	60061736	60072306	10571	-1.026214	0.490997	1.37E-272	Loss	Gain
Chr22_CNVR_45	Chr22	60083760	60106666	22907	-0.9415738	0.520665	0	Loss	Gain
Chr22_CNVR_46	Chr22	60107548	60132214	24667	-0.9629121	0.51302	0	Loss	Gain
Chr22_CNVR_47	Chr22	60139264	60165692	26429	-0.8744016	0.54548	0	Loss	Gain
Chr22_CNVR_48	Chr22	60426470	60435278	8809	-0.8850295	0.541476	7.95E-179	Loss	Gain
Chr22_CNVR_49	Chr22	60445852	60466114	20263	-0.9051313	0.533984	0	Loss	Gain
Chr22_CNVR_50	Chr22	60466996	60489020	22025	-0.9449308	0.519454	0	Loss	Gain
Chr22_CNVR_51	Chr22	60599146	60608836	9691	-0.9391642	0.521535	9.83E-217	Loss	Gain
Chr22_CNVR_52	Chr22	60636148	60655528	19381	-0.9222524	0.527685	0	Loss	Gain
Chr22_CNVR_53	Chr22	60666102	60681078	14977	-0.9006723	0.535637	0	Loss	Gain
Chr22_CNVR_54	Chr22	60681960	60690768	8809	-0.9083484	0.532795	1.06E-186	Loss	Gain
Chr22_CNVR_55	Chr22	61119816	61130388	10573	-0.8679854	0.547911	2.50E-207	Loss	Gain
Chr22_CNVR_56	Chr22	61138318	61147126	8809	-0.8620056	0.550187	3.96E-171	Loss	Gain
Chr22_CNVR_57	Chr22	61154176	61163866	9691	-0.8366299	0.55995	8.18E-179	Loss	Gain
Chr22_CNVR_58	Chr22	61173558	61198224	24667	-0.9565455	0.515289	0	Loss	Gain
Chr22_CNVR_59	Chr22	61213202	61222012	8811	-0.9800737	0.506954	2.31E-211	Loss	Gain
Chr22_CNVR_60	Chr22	61223774	61236108	12335	-1.00298	0.498968	3.05E-306	Loss	Gain
Chr22_CNVR_61	Chr22	61244918	61253728	8811	-0.879339	0.543616	6.46E-177	Loss	Gain
Chr22_CNVR_62	Chr22	61254610	61274872	20263	-0.9449723	0.51944	0	Loss	Gain
Chr23_CNVR_19	Chr23	7080994	7103943	22950	-1.046376	0.484183	0	Loss	Gain
Chr23_CNVR_20	Chr23	7831918	7842015	10098	-0.8418735	0.557919	1.05E-180	Loss	Gain
Chr23_CNVR_21	Chr23	7866802	7875981	9180	-0.8133906	0.569043	3.64E-155	Loss	Gain
Chr23_CNVR_22	Chr23	8132104	8142201	10098	-0.9076548	0.533051	6.86E-205	Loss	Gain
Chr23_CNVR_23	Chr23	8143120	8161479	18360	-0.9006749	0.535636	0	Loss	Gain
Chr23_CNVR_24	Chr23	17875756	17886771	11016	-0.897773	0.536715	2.59E-219	Loss	Gain
Chr23_CNVR_1	Chr23	18577108	18586287	9180	1.092774	2.13284	2.98E-289	Gain	Loss
Chr23_CNVR_2	Chr23	25332670	25346439	13770	3.649269	12.547	0	Gain	Loss
Chr23_CNVR_3	Chr23	25348276	25362045	13770	3.097601	8.55994	0	Gain	Loss
Chr23_CNVR_4	Chr23	25717312	25742097	24786	1.200541	2.29826	0	Gain	Loss
Chr23_CNVR_25	Chr23	25748524	25758621	10098	-2.415758	0.187406	0	Loss	Gain
Chr23_CNVR_26	Chr23	25779736	25797177	17442	-1.656836	0.317134	0	Loss	Gain
Chr23_CNVR_27	Chr23	25799014	25849503	50490	-2.05886	0.240006	0	Loss	Gain
Chr23_CNVR_28	Chr23	25917436	25996383	78948	-2.937735	0.130513	0	Loss	Gain
Chr23_CNVR_29	Chr23	25997302	26008317	11016	-1.795427	0.288086	0	Loss	Gain
Chr23_CNVR_30	Chr23	26010154	26042283	32130	-1.679408	0.312211	0	Loss	Gain
Chr23_CNVR_31	Chr23	26047792	26127657	79866	-3.229999	0.106579	0	Loss	Gain
Chr23_CNVR_32	Chr23	26133166	26144181	11016	-1.287947	0.409533	0	Loss	Gain
Chr23_CNVR_33	Chr23	26149690	26178147	28458	-2.811865	0.142411	0	Loss	Gain
Chr23_CNVR_34	Chr23	26191000	26208441	17442	-2.672903	0.156811	0	Loss	Gain
Chr23_CNVR_35	Chr23	26211196	26231391	20196	-3.13605	0.113751	0	Loss	Gain
Chr23_CNVR_36	Chr23	26275456	26286471	11016	-1.813892	0.284423	0	Loss	Gain
Chr23_CNVR_37	Chr23	26305750	26316765	11016	-1.777829	0.291622	0	Loss	Gain
Chr23_CNVR_5	Chr23	26364502	26373681	9180	0.9772837	1.96876	1.15E-239	Gain	Loss
Chr23_CNVR_6	Chr23	26493022	26519643	26622	1.122037	2.17654	0	Gain	Loss
Chr23_CNVR_7	Chr23	26520562	26535249	14688	1.569586	2.9682	0	Gain	Loss
Chr23_CNVR_8	Chr23	26549938	26559117	9180	0.8291975	1.7767	9.92E-180	Gain	Loss
Chr23_CNVR_9	Chr23	26568298	26578395	10098	0.9355486	1.91262	2.62E-244	Gain	Loss
Chr23_CNVR_10	Chr23	26589412	26616033	26622	1.087525	2.12509	0	Gain	Loss
Chr23_CNVR_11	Chr23	26889598	26903367	13770	0.9677863	1.95584	0	Gain	Loss
Chr23_CNVR_38	Chr23	27653374	27662553	9180	-1.077739	0.473771	1.05E-245	Loss	Gain

Chr23_CNVR_39	Chr23	27663472	27687339	23868	-1.787172	0.289739	0	Loss	Gain
Chr23_CNVR_12	Chr23	27845236	27856251	11016	0.8096814	1.75282	2.92E-206	Gain	Loss
Chr23_CNVR_13	Chr23	28455706	28473147	17442	1.028115	2.03936	0	Gain	Loss
Chr23_CNVR_40	Chr23	28821070	28850445	29376	-1.765859	0.294052	0	Loss	Gain
Chr23_CNVR_14	Chr23	29009260	29018439	9180	0.8486732	1.80084	2.56E-187	Gain	Loss
Chr23_CNVR_15	Chr23	29779462	29812509	33048	3.512487	11.4121	0	Gain	Loss
Chr23_CNVR_16	Chr23	30394522	30404619	10098	0.9875075	1.98276	3.72E-268	Gain	Loss
Chr23_CNVR_17	Chr23	43417270	43430121	12852	1.141618	2.20628	0	Gain	Loss
Chr23_CNVR_18	Chr23	43472350	43483365	11016	1.007447	2.01035	1.78E-302	Gain	Loss
Chr23_CNVR_41	Chr23	48973924	48988611	14688	-0.8675383	0.548081	1.76E-275	Loss	Gain
Chr23_CNVR_42	Chr23	50253616	50262795	9180	-0.8350035	0.560582	3.29E-162	Loss	Gain
Chr23_CNVR_43	Chr23	50272894	50283909	11016	-0.9227378	0.527507	1.76E-229	Loss	Gain
Chr23_CNVR_44	Chr23	50286664	50300433	13770	-0.8533202	0.553509	2.56E-251	Loss	Gain
Chr23_CNVR_45	Chr23	50311450	50321547	10098	-0.909491	0.532373	1.42E-205	Loss	Gain
Chr23_CNVR_46	Chr23	51407542	51418557	11016	-0.9261569	0.526258	7.02E-231	Loss	Gain
Chr23_CNVR_47	Chr23	51427738	51438753	11016	-0.9116682	0.53157	5.85E-225	Loss	Gain
Chr23_CNVR_48	Chr23	51442426	51461703	19278	-0.9265762	0.526105	0	Loss	Gain
Chr23_CNVR_49	Chr23	51486490	51497505	11016	-0.8367676	0.559897	6.22E-195	Loss	Gain
Chr24_CNVR_4	Chr24	907975	916734	8760	-1.025664	0.491184	1.90E-228	Loss	Gain
Chr24_CNVR_5	Chr24	929875	939510	9636	-0.8583989	0.551564	3.17E-187	Loss	Gain
Chr24_CNVR_6	Chr24	964915	978054	13140	-0.8167712	0.567711	7.55E-234	Loss	Gain
Chr24_CNVR_7	Chr24	1015723	1025358	9636	-0.9628816	0.513031	1.15E-226	Loss	Gain
Chr24_CNVR_8	Chr24	1049011	1077042	28032	-0.9749847	0.508745	0	Loss	Gain
Chr24_CNVR_9	Chr24	1080547	1093686	13140	-0.9857988	0.504946	0	Loss	Gain
Chr24_CNVR_10	Chr24	1393279	1402038	8760	-0.8164886	0.567822	1.69E-156	Loss	Gain
Chr24_CNVR_1	Chr24	10925035	10933794	8760	0.9933903	1.99086	7.27E-238	Gain	Loss
Chr24_CNVR_11	Chr24	19857607	19881258	23652	-0.8359903	0.560198	0	Loss	Gain
Chr24_CNVR_12	Chr24	19885639	19909290	23652	-0.8634002	0.549656	0	Loss	Gain
Chr24_CNVR_13	Chr24	19933819	19953090	19272	-1.041877	0.485695	0	Loss	Gain
Chr24_CNVR_14	Chr24	19953967	19985502	31536	-0.9677172	0.511314	0	Loss	Gain
Chr24_CNVR_15	Chr24	19987255	20002146	14892	-0.9445247	0.519601	0	Loss	Gain
Chr24_CNVR_16	Chr24	20003023	20011782	8760	-0.8445799	0.556873	8.91E-166	Loss	Gain
Chr24_CNVR_17	Chr24	20027551	20041566	14016	-1.040475	0.486167	0	Loss	Gain
Chr24_CNVR_18	Chr24	20051203	20062590	11388	-0.999403	0.500207	3.29E-284	Loss	Gain
Chr24_CNVR_19	Chr24	20070475	20080986	10512	-0.8695337	0.547324	1.51E-208	Loss	Gain
Chr24_CNVR_20	Chr24	24850807	24866574	15768	-1.352463	0.391623	0	Loss	Gain
Chr24_CNVR_21	Chr24	43198627	43207386	8760	-0.8545958	0.55302	4.05E-169	Loss	Gain
Chr24_CNVR_2	Chr24	44177995	44194638	16644	2.616607	6.13306	0	Gain	Loss
Chr24_CNVR_22	Chr24	48995119	49010010	14892	-0.8132517	0.569098	8.72E-263	Loss	Gain
Chr24_CNVR_23	Chr24	58117783	58126542	8760	-0.8499222	0.554815	1.48E-167	Loss	Gain
Chr24_CNVR_24	Chr24	58137055	58145814	8760	-0.8338108	0.561045	3.34E-162	Loss	Gain
Chr24_CNVR_3	Chr24	62414563	62423322	8760	1.070633	2.10035	1.67E-269	Gain	Loss
Chr25_CNVR_4	Chr25	501967	513343	11377	-0.8507302	0.554504	2.85E-199	Loss	Gain
Chr25_CNVR_5	Chr25	2490871	2507935	17065	-2.260729	0.208667	0	Loss	Gain
Chr25_CNVR_1	Chr25	19439215	19451539	12325	0.8482411	1.8003	1.82E-262	Gain	Loss
Chr25_CNVR_2	Chr25	19512211	19524535	12325	0.9633621	1.94985	0	Gain	Loss
Chr25_CNVR_3	Chr25	19556767	19566247	9481	0.8791736	1.83932	5.98E-216	Gain	Loss
Chr25_CNVR_6	Chr25	27264955	27281071	16117	-0.9450192	0.519423	0	Loss	Gain
Chr25_CNVR_7	Chr25	41934307	41947579	13273	-0.936665	0.522439	1.17E-271	Loss	Gain
Chr25_CNVR_8	Chr25	41983603	41993083	9481	-0.9824496	0.50612	4.95E-210	Loss	Gain
Chr29_CNVR_1	Chr29	5650561	5660544	9984	0.9470678	1.92795	2.41E-296	Gain	Loss
Chr29_CNVR_2	Chr29	5748865	5756544	7680	0.927826	1.90241	1.89E-220	Gain	Loss
Chr29_CNVR_3	Chr29	8469121	8476800	7680	0.9147687	1.88527	4.59E-215	Gain	Loss
Chr29_CNVR_4	Chr29	9646465	9657216	10752	1.099957	2.14348	0	Gain	Loss
Chr29_CNVR_5	Chr29	19843201	19850880	7680	0.8661984	1.82285	2.26E-195	Gain	Loss
Chr29_CNVR_6	Chr29	20002177	20009856	7680	1.074075	2.10537	6.06E-283	Gain	Loss

Chr29_CNVR_7	Chr29	22119553	22131840	12288	1.256838	2.38971	0	Gain	Loss
Chr29_CNVR_10	Chr29	27060865	27068544	7680	-4.117788	0.0576	0	Loss	Gain
Chr29_CNVR_11	Chr29	27391105	27404160	13056	-1.678487	0.31241	0	Loss	Gain
Chr29_CNVR_8	Chr29	28027009	28040064	13056	2.600228	6.06382	0	Gain	Loss
Chr29_CNVR_12	Chr29	40712065	40721280	9216	-0.9736189	0.509227	2.92E-250	Loss	Gain
Chr29_CNVR_13	Chr29	40725889	40735104	9216	-0.8435396	0.557275	1.73E-197	Loss	Gain
Chr29_CNVR_14	Chr29	40779649	40789632	9984	-0.8102339	0.570289	1.14E-199	Loss	Gain
Chr29_CNVR_15	Chr29	40888705	40897920	9216	-0.9599311	0.514081	1.38E-244	Loss	Gain
Chr29_CNVR_9	Chr29	42514561	42576000	61440	4.582856	23.965	0	Gain	Loss
Chr29_CNVR_16	Chr29	42916225	42958464	42240	-0.9365013	0.522498	0	Loss	Gain
Chr29_CNVR_17	Chr29	42966913	42974592	7680	-0.8131219	0.569149	5.32E-155	Loss	Gain
Chr29_CNVR_18	Chr29	42977665	42986112	8448	-0.9196855	0.528624	3.18E-209	Loss	Gain
Chr29_CNVR_19	Chr29	42992257	43001472	9216	-0.8565381	0.552276	1.25E-202	Loss	Gain
Chr29_CNVR_20	Chr29	43069057	43076736	7680	-0.8730868	0.545977	1.06E-174	Loss	Gain
Chr29_CNVR_21	Chr29	43142785	43153536	10752	-0.9601211	0.514014	4.37E-285	Loss	Gain
Chr29_CNVR_22	Chr29	43181185	43189632	8448	-0.9323878	0.52399	5.51E-214	Loss	Gain
Chr29_CNVR_23	Chr29	43524481	43532928	8448	-0.8551373	0.552813	2.08E-185	Loss	Gain
Chr29_CNVR_24	Chr29	44432257	44444544	12288	-0.8778833	0.544165	8.10E-281	Loss	Gain
Chr29_CNVR_25	Chr29	44956033	44966784	10752	-0.8252734	0.564375	1.08E-221	Loss	Gain
Chr29_CNVR_26	Chr29	45924481	45932160	7680	-0.8947633	0.537835	5.81E-182	Loss	Gain
Chr29_CNVR_27	Chr29	46203265	46211712	8448	-0.9276545	0.525712	3.29E-212	Loss	Gain
Chr29_CNVR_28	Chr29	46944385	46955136	10752	-0.9240514	0.527027	8.73E-268	Loss	Gain
Chr29_CNVR_29	Chr29	47932801	47941248	8448	-0.9673908	0.51143	3.25E-227	Loss	Gain
Chr29_CNVR_30	Chr29	47952001	47961216	9216	-0.8266205	0.563849	7.59E-191	Loss	Gain
Chr29_CNVR_31	Chr29	48027265	48036480	9216	-0.8803187	0.543247	4.02E-212	Loss	Gain
Chr29_CNVR_32	Chr29	48285313	48295296	9984	-0.8764368	0.544711	6.15E-228	Loss	Gain
Chr29_CNVR_33	Chr29	48355969	48364416	8448	-0.8326619	0.561492	2.78E-177	Loss	Gain
Chr29_CNVR_34	Chr29	48391297	48402048	10752	-0.8790127	0.543739	1.64E-246	Loss	Gain
Chr29_CNVR_35	Chr29	48513409	48533376	19968	-0.9524072	0.516769	0	Loss	Gain
Chr29_CNVR_36	Chr29	48561793	48575616	13824	-0.8619445	0.55021	4.21E-306	Loss	Gain
Chr29_CNVR_37	Chr29	48641665	48650880	9216	-0.9397886	0.521309	2.77E-236	Loss	Gain
Chr29_CNVR_38	Chr29	48664705	48673920	9216	-0.8958047	0.537447	2.33E-218	Loss	Gain
Chr29_CNVR_39	Chr29	49099393	49111680	12288	-0.8481074	0.555513	5.12E-265	Loss	Gain
Chr29_CNVR_40	Chr29	49129345	49137024	7680	-0.985344	0.505105	5.71E-213	Loss	Gain
Chr29_CNVR_41	Chr29	49353601	49361280	7680	-0.9015995	0.535293	2.88E-184	Loss	Gain
Chr29_CNVR_42	Chr29	49368193	49392768	24576	-0.9429569	0.520166	0	Loss	Gain
Chr29_CNVR_43	Chr29	49405825	49413504	7680	-0.9342487	0.523315	2.34E-195	Loss	Gain
Chr29_CNVR_44	Chr29	49984129	49993344	9216	-0.8588135	0.551406	1.56E-203	Loss	Gain
Chr29_CNVR_45	Chr29	50022529	50033280	10752	-0.9704375	0.510351	4.52E-290	Loss	Gain
Chr29_CNVR_46	Chr29	50044033	50057088	13056	-0.9424763	0.520339	0	Loss	Gain
Chr29_CNVR_47	Chr29	50057857	50075520	17664	-0.9083853	0.532781	0	Loss	Gain
Chr29_CNVR_48	Chr29	50083969	50093184	9216	-1.117694	0.46083	0	Loss	Gain
Chr29_CNVR_49	Chr29	50093953	50105472	11520	-0.9270417	0.525936	2.75E-288	Loss	Gain
Chr29_CNVR_50	Chr29	50106241	50116992	10752	-0.9270178	0.525944	3.36E-269	Loss	Gain
Chr29_CNVR_51	Chr29	50126977	50147712	20736	-0.9915296	0.502944	0	Loss	Gain
Chr29_CNVR_52	Chr29	50160769	50173056	12288	-1.176949	0.442286	0	Loss	Gain
Chr29_CNVR_53	Chr29	50186113	50193792	7680	-0.9682762	0.511116	4.69E-207	Loss	Gain
Chr29_CNVR_54	Chr29	50200705	50209920	9216	-0.8241376	0.56482	7.08E-190	Loss	Gain
Chr29_CNVR_55	Chr29	50229121	50239104	9984	-1.024054	0.491733	8.68E-294	Loss	Gain
Chr29_CNVR_56	Chr29	50340481	50351232	10752	-0.8749458	0.545274	1.30E-244	Loss	Gain
Chr29_CNVR_57	Chr29	50389633	50397312	7680	-0.787874	0.579197	6.75E-147	Loss	Gain
Chr29_CNVR_58	Chr29	50434945	50446464	11520	-0.8288907	0.562962	3.84E-239	Loss	Gain
Chr29_CNVR_59	Chr29	50523265	50536320	13056	-0.9980256	0.500685	0	Loss	Gain
Chr29_CNVR_60	Chr29	50689921	50701440	11520	-1.089745	0.469844	0	Loss	Gain
Chr29_CNVR_61	Chr29	50732929	50742912	9984	-0.8794482	0.543575	3.03E-229	Loss	Gain
Chr29_CNVR_62	Chr29	50838145	50850432	12288	-0.9595438	0.514219	0	Loss	Gain

Chr29_CNVR_63	Chr29	50912641	50931072	18432	-0.9377545	0.522045	0	Loss	Gain
Chr29_CNVR_64	Chr29	50961793	50970240	8448	-1.014871	0.494873	2.21E-245	Loss	Gain
Chr29_CNVR_65	Chr29	50977153	50989440	12288	-0.968806	0.510929	0	Loss	Gain
Chr29_CNVR_66	Chr29	51021697	51029376	7680	-0.8322967	0.561634	3.11E-161	Loss	Gain
Chr29_CNVR_67	Chr29	51050113	51058560	8448	-1.013151	0.495463	1.02E-244	Loss	Gain
Chr29_CNVR_68	Chr29	51084673	51095424	10752	-0.8608245	0.550638	4.88E-238	Loss	Gain
Chr29_CNVR_69	Chr29	51100801	51110016	9216	-0.878635	0.543882	1.90E-211	Loss	Gain
Chr29_CNVR_70	Chr29	51124609	51135360	10752	-0.8825986	0.54239	3.43E-248	Loss	Gain
Chr29_CNVR_71	Chr29	51145345	51160704	15360	-0.817415	0.567458	0	Loss	Gain
Chr29_CNVR_72	Chr29	51172225	51179904	7680	-0.8188181	0.566906	7.61E-157	Loss	Gain
Chr29_CNVR_73	Chr29	51374209	51395712	21504	-1.160891	0.447236	0	Loss	Gain
Chr29_CNVR_74	Chr29	51488641	51497856	9216	-0.8872296	0.540651	6.70E-215	Loss	Gain
Chr3_CNVR_1	Chr3	8517629	8539028	21400	4.529865	23.1007	0	Gain	Loss
Chr3_CNVR_2	Chr3	8612645	8670852	58208	4.17149	18.0195	0	Gain	Loss
Chr3_CNVR_3	Chr3	8732485	8752172	19688	4.773873	27.3577	0	Gain	Loss
Chr3_CNVR_9	Chr3	11867157	11887700	20544	-2.250975	0.210082	0	Loss	Gain
Chr3_CNVR_10	Chr3	11893693	11907388	13696	-2.414851	0.187524	0	Loss	Gain
Chr3_CNVR_11	Chr3	13957509	13966068	8560	-0.8083878	0.57102	7.58E-154	Loss	Gain
Chr3_CNVR_12	Chr3	13984901	14004588	19688	-0.9374669	0.522149	0	Loss	Gain
Chr3_CNVR_13	Chr3	14010581	14040540	29960	-0.8845957	0.541639	0	Loss	Gain
Chr3_CNVR_14	Chr3	14048245	14056804	8560	-0.8487094	0.555281	3.64E-167	Loss	Gain
Chr3_CNVR_15	Chr3	14214309	14229716	15408	-0.8972868	0.536895	0	Loss	Gain
Chr3_CNVR_16	Chr3	14692813	14701372	8560	-0.9490073	0.517989	1.83E-201	Loss	Gain
Chr3_CNVR_17	Chr3	14702229	14714212	11984	-0.862002	0.550189	1.97E-239	Loss	Gain
Chr3_CNVR_18	Chr3	15465781	15475196	9416	-0.9424051	0.520365	7.21E-219	Loss	Gain
Chr3_CNVR_19	Chr3	15579629	15593324	13696	-0.9181943	0.529171	5.89E-304	Loss	Gain
Chr3_CNVR_20	Chr3	15670365	15680636	10272	-0.8826574	0.542367	6.62E-214	Loss	Gain
Chr3_CNVR_21	Chr3	33419525	33428084	8560	-0.8652257	0.548961	1.05E-172	Loss	Gain
Chr3_CNVR_4	Chr3	40003877	40014148	10272	0.9294124	1.9045	5.69E-253	Gain	Loss
Chr3_CNVR_22	Chr3	47039341	47048756	9416	-3.509299	0.0878205	0	Loss	Gain
Chr3_CNVR_5	Chr3	50102965	50111524	8560	0.9166606	1.88774	3.37E-206	Gain	Loss
Chr3_CNVR_6	Chr3	54780149	54799836	19688	3.057275	8.32399	0	Gain	Loss
Chr3_CNVR_7	Chr3	62694725	62711844	17120	1.306325	2.47311	0	Gain	Loss
Chr3_CNVR_8	Chr3	62821413	62831684	10272	1.098377	2.14114	0	Gain	Loss
Chr3_CNVR_23	Chr3	82711429	82720844	9416	-0.8485322	0.555349	1.31E-183	Loss	Gain
Chr3_CNVR_24	Chr3	92434733	92444148	9416	-0.8542697	0.553145	1.01E-185	Loss	Gain
Chr3_CNVR_25	Chr3	92549437	92559708	10272	-0.9072378	0.533205	5.59E-224	Loss	Gain
Chr3_CNVR_26	Chr3	93517573	93526132	8560	-0.8389813	0.559038	6.34E-164	Loss	Gain
Chr3_CNVR_27	Chr3	98987413	98997684	10272	-0.8275737	0.563476	9.19E-192	Loss	Gain
Chr3_CNVR_28	Chr3	99013949	99022508	8560	-0.8050774	0.572331	9.10E-153	Loss	Gain
Chr3_CNVR_29	Chr3	99810029	99822012	11984	-0.8993992	0.53611	3.42E-257	Loss	Gain
Chr3_CNVR_30	Chr3	100214061	100226044	11984	-0.859924	0.550982	1.87E-238	Loss	Gain
Chr3_CNVR_31	Chr3	100229469	100238884	9416	-0.9129292	0.531106	1.21E-207	Loss	Gain
Chr3_CNVR_32	Chr3	102657085	102665644	8560	-0.8060215	0.571957	4.48E-153	Loss	Gain
Chr3_CNVR_33	Chr3	103041429	103050844	9416	-0.8789466	0.543764	7.20E-195	Loss	Gain
Chr3_CNVR_34	Chr3	103053413	103064540	11128	-0.8586717	0.55146	5.00E-221	Loss	Gain
Chr3_CNVR_35	Chr3	103065397	103073956	8560	-0.8200981	0.566403	1.11E-157	Loss	Gain
Chr3_CNVR_36	Chr3	105261893	105270452	8560	-0.93944	0.521435	3.95E-198	Loss	Gain
Chr3_CNVR_37	Chr3	105304693	105314108	9416	-0.8157694	0.568105	1.10E-171	Loss	Gain
Chr3_CNVR_38	Chr3	105710437	105725844	15408	-3.746248	0.074519	0	Loss	Gain
Chr3_CNVR_39	Chr3	109475125	109485396	10272	-0.9620159	0.513339	8.52E-247	Loss	Gain
Chr3_CNVR_40	Chr3	115180365	115188924	8560	-0.8808246	0.543057	5.59E-178	Loss	Gain
Chr3_CNVR_41	Chr3	115205189	115213748	8560	-0.9131846	0.531012	4.83E-189	Loss	Gain
Chr3_CNVR_42	Chr3	115685405	115693964	8560	-0.776842	0.583643	1.19E-143	Loss	Gain
Chr3_CNVR_43	Chr3	115698245	115711084	12840	-1.013572	0.495318	0	Loss	Gain
Chr3_CNVR_44	Chr3	115773573	115788980	15408	-0.9146781	0.530462	0	Loss	Gain

Chr3_CNVR_45	Chr3	115792405	115807812	15408	-0.8316783	0.561875	5.02E-289	Loss	Gain
Chr3_CNVR_46	Chr3	115836917	115846332	9416	-0.8835316	0.542039	1.40E-196	Loss	Gain
Chr3_CNVR_47	Chr3	115892557	115901116	8560	-0.8468774	0.555987	1.49E-166	Loss	Gain
Chr3_CNVR_48	Chr3	116201573	116215268	13696	-0.8489118	0.555203	2.58E-266	Loss	Gain
Chr3_CNVR_49	Chr3	116678365	116686924	8560	-0.8444081	0.556939	9.92E-166	Loss	Gain
Chr3_CNVR_50	Chr3	117601989	117611404	9416	-0.8753457	0.545123	1.58E-193	Loss	Gain
Chr3_CNVR_51	Chr3	117624245	117644788	20544	-0.8855694	0.541274	0	Loss	Gain
Chr3_CNVR_52	Chr3	118095901	118104460	8560	-0.8471432	0.555884	1.21E-166	Loss	Gain
Chr3_CNVR_53	Chr3	118135277	118147260	11984	-0.8209273	0.566078	2.61E-220	Loss	Gain
Chr3_CNVR_54	Chr3	118205469	118214884	9416	-0.975148	0.508688	1.75E-231	Loss	Gain
Chr3_CNVR_55	Chr3	118239709	118248268	8560	-0.875848	0.544933	2.72E-176	Loss	Gain
Chr3_CNVR_56	Chr3	118681405	118695100	13696	-0.9423208	0.520395	0	Loss	Gain
Chr3_CNVR_57	Chr3	118927077	118978436	51360	-0.9330865	0.523737	0	Loss	Gain
Chr3_CNVR_58	Chr3	118987853	119003260	15408	-0.8990391	0.536244	0	Loss	Gain
Chr3_CNVR_59	Chr3	119184733	119198428	13696	-0.9585214	0.514584	0	Loss	Gain
Chr3_CNVR_60	Chr3	119220685	119231812	11128	-0.8521243	0.553968	3.56E-218	Loss	Gain
Chr3_CNVR_61	Chr3	119357645	119367916	10272	-0.8961033	0.537336	2.11E-219	Loss	Gain
Chr3_CNVR_62	Chr3	119395309	119405580	10272	-0.8769722	0.544509	1.36E-211	Loss	Gain
Chr3_CNVR_63	Chr3	119763389	119771948	8560	-0.8678922	0.547947	1.33E-173	Loss	Gain
Chr3_CNVR_64	Chr3	120239325	120251308	11984	-0.8736723	0.545756	6.10E-245	Loss	Gain
Chr3_CNVR_65	Chr3	120266717	120276132	9416	-0.9849371	0.505248	2.79E-235	Loss	Gain
Chr3_CNVR_66	Chr3	120276989	120295820	18832	-0.9284439	0.525425	0	Loss	Gain
Chr3_CNVR_67	Chr3	120297533	120318076	20544	-0.9478824	0.518393	0	Loss	Gain
Chr3_CNVR_68	Chr3	120342045	120356596	14552	-0.9521161	0.516874	0	Loss	Gain
Chr3_CNVR_69	Chr3	120374573	120383132	8560	-1.03366	0.488469	2.06E-231	Loss	Gain
Chr3_CNVR_70	Chr3	120390837	120401964	11128	-0.9841655	0.505518	2.58E-277	Loss	Gain
Chr3_CNVR_71	Chr3	120411381	120431924	20544	-0.936118	0.522637	0	Loss	Gain
Chr3_CNVR_72	Chr3	120445621	120455036	9416	-0.9736296	0.509223	6.79E-231	Loss	Gain
Chr3_CNVR_73	Chr3	120486709	120508108	21400	-0.9892523	0.503739	0	Loss	Gain
Chr3_CNVR_74	Chr3	120508965	120524372	15408	-0.9947338	0.501828	0	Loss	Gain
Chr3_CNVR_75	Chr3	120548341	120557756	9416	-1.095437	0.467994	8.08E-279	Loss	Gain
Chr3_CNVR_76	Chr3	120565461	120576588	11128	-0.9437252	0.519889	7.12E-259	Loss	Gain
Chr3_CNVR_77	Chr3	120587717	120597988	10272	-0.9073432	0.533166	5.06E-224	Loss	Gain
Chr3_CNVR_78	Chr3	120598845	120611684	12840	-0.8808454	0.543049	6.29E-266	Loss	Gain
Chr3_CNVR_79	Chr3	120614253	120630516	16264	-0.8969401	0.537025	0	Loss	Gain
Chr3_CNVR_80	Chr3	120648493	120659620	11128	-0.9938859	0.502123	8.77E-282	Loss	Gain
Chr3_CNVR_81	Chr3	120679309	120704988	25680	-1.008627	0.497019	0	Loss	Gain
Chr3_CNVR_82	Chr3	120709269	120717828	8560	-0.884771	0.541573	2.55E-179	Loss	Gain
Chr3_CNVR_83	Chr3	120724677	120734092	9416	-0.9073388	0.533168	1.58E-205	Loss	Gain
Chr3_CNVR_84	Chr3	120808565	120846228	37664	-0.9620358	0.513332	0	Loss	Gain
Chr3_CNVR_85	Chr3	120847941	120881324	33384	-0.9481395	0.5183	0	Loss	Gain
Chr3_CNVR_86	Chr3	120887317	120899300	11984	-0.939067	0.52157	2.37E-276	Loss	Gain
Chr3_CNVR_87	Chr3	120900157	120924124	23968	-1.027808	0.490455	0	Loss	Gain
Chr3_CNVR_88	Chr3	120971205	120983188	11984	-0.8590023	0.551334	5.07E-238	Loss	Gain
Chr3_CNVR_89	Chr3	121064509	121073068	8560	-0.9610933	0.513667	1.08E-205	Loss	Gain
Chr3_CNVR_90	Chr3	121080773	121094468	13696	-0.8817866	0.542695	5.04E-284	Loss	Gain
Chr3_CNVR_91	Chr3	121114157	121126996	12840	-0.9279018	0.525622	3.98E-290	Loss	Gain
Chr3_CNVR_92	Chr3	121141549	121150108	8560	-0.8623958	0.550038	9.42E-172	Loss	Gain
Chr3_CNVR_93	Chr3	121151821	121173220	21400	-0.9071399	0.533241	0	Loss	Gain
Chr3_CNVR_94	Chr3	121212597	121245980	33384	-0.9674843	0.511397	0	Loss	Gain
Chr3_CNVR_95	Chr3	121246837	121269948	23112	-0.9508793	0.517317	0	Loss	Gain
Chr3_CNVR_96	Chr3	121286213	121299908	13696	-1.083189	0.471984	0	Loss	Gain
Chr3_CNVR_97	Chr3	121303333	121317884	14552	-0.9298446	0.524915	0	Loss	Gain
Chr3_CNVR_98	Chr3	121319597	121328156	8560	-0.9385738	0.521748	7.90E-198	Loss	Gain
Chr3_CNVR_99	Chr3	121356405	121364964	8560	-0.8184707	0.567043	3.79E-157	Loss	Gain
Chr3_CNVR_100	Chr3	121411189	121430876	19688	-1.462052	0.362976	0	Loss	Gain

Chr4_CNVR_1	Chr4	2846949	2855217	8269	1.154998	2.22684	2.11E-295	Gain	Loss
Chr4_CNVR_2	Chr4	19987351	19997273	9923	1.210833	2.31471	0	Gain	Loss
Chr4_CNVR_3	Chr4	28239983	28254041	14059	2.502788	5.6678	0	Gain	Loss
Chr4_CNVR_9	Chr4	30104869	30113965	9097	-1.010064	0.496524	8.15E-246	Loss	Gain
Chr4_CNVR_10	Chr4	43480767	43490689	9923	-1.203574	0.434198	0	Loss	Gain
Chr4_CNVR_11	Chr4	47480965	47497505	16541	-1.127554	0.457691	0	Loss	Gain
Chr4_CNVR_4	Chr4	54383935	54395511	11577	1.037386	2.05251	0	Gain	Loss
Chr4_CNVR_5	Chr4	59801611	59810707	9097	1.092141	2.1319	7.65E-297	Gain	Loss
Chr4_CNVR_12	Chr4	65845327	65857731	12405	-0.9575436	0.514933	2.24E-306	Loss	Gain
Chr4_CNVR_13	Chr4	65878407	65901563	23157	-0.9125036	0.531262	0	Loss	Gain
Chr4_CNVR_14	Chr4	66061175	66071097	9923	-0.8756015	0.545027	2.14E-211	Loss	Gain
Chr4_CNVR_6	Chr4	73983007	73997065	14059	1.165734	2.24347	0	Gain	Loss
Chr4_CNVR_15	Chr4	75484839	75493109	8271	-0.8335548	0.561145	2.49E-162	Loss	Gain
Chr4_CNVR_16	Chr4	77775629	77790515	14887	-0.912932	0.531105	0	Loss	Gain
Chr4_CNVR_17	Chr4	77792169	77802919	10751	-0.9906343	0.503256	4.43E-281	Loss	Gain
Chr4_CNVR_18	Chr4	77817807	77829383	11577	-0.8361495	0.560137	1.21E-227	Loss	Gain
Chr4_CNVR_19	Chr4	77830211	77838481	8271	-0.9559645	0.515497	2.10E-204	Loss	Gain
Chr4_CNVR_20	Chr4	86737001	86745271	8271	-3.909588	0.0665421	0	Loss	Gain
Chr4_CNVR_7	Chr4	90632999	90642095	9097	1.25533	2.38722	0	Gain	Loss
Chr4_CNVR_21	Chr4	91970257	91982661	12405	-0.9726345	0.509575	0	Loss	Gain
Chr4_CNVR_22	Chr4	91990105	92020703	30599	-0.9319844	0.524137	0	Loss	Gain
Chr4_CNVR_23	Chr4	92027321	92036417	9097	-0.8705717	0.54693	4.53E-192	Loss	Gain
Chr4_CNVR_24	Chr4	92043033	92053783	10751	-0.8880494	0.540344	2.28E-234	Loss	Gain
Chr4_CNVR_25	Chr4	92071151	92085209	14059	-0.8822772	0.54251	1.84E-302	Loss	Gain
Chr4_CNVR_26	Chr4	92091827	92104231	12405	-0.8920409	0.538851	3.76E-272	Loss	Gain
Chr4_CNVR_27	Chr4	92111675	92148889	37215	-1.01051	0.496371	0	Loss	Gain
Chr4_CNVR_28	Chr4	92149717	92172045	22329	-1.023114	0.492053	0	Loss	Gain
Chr4_CNVR_29	Chr4	92172873	92186931	14059	-1.081905	0.472405	0	Loss	Gain
Chr4_CNVR_30	Chr4	92191067	92199335	8269	-0.9867505	0.504613	2.53E-215	Loss	Gain
Chr4_CNVR_31	Chr4	92205953	92257225	51273	-1.019173	0.493399	0	Loss	Gain
Chr4_CNVR_32	Chr4	92258053	92274593	16541	-0.9965615	0.501193	0	Loss	Gain
Chr4_CNVR_33	Chr4	93616815	93636661	19847	-0.9181754	0.529178	0	Loss	Gain
Chr4_CNVR_34	Chr4	96588225	96597321	9097	-0.8194139	0.566672	3.41E-173	Loss	Gain
Chr4_CNVR_35	Chr4	96622959	96635363	12405	-0.8523508	0.553881	6.73E-252	Loss	Gain
Chr4_CNVR_36	Chr4	98732637	98747521	14885	-1.21676	0.430248	0	Loss	Gain
Chr4_CNVR_37	Chr4	106471703	106482453	10751	-0.9093475	0.532426	6.58E-244	Loss	Gain
Chr4_CNVR_38	Chr4	106506437	106521321	14885	-0.8778092	0.544193	0	Loss	Gain
Chr4_CNVR_39	Chr4	106638757	106660257	21501	-1.506265	0.352021	0	Loss	Gain
Chr4_CNVR_40	Chr4	106690031	106705743	15713	-1.585597	0.333187	0	Loss	Gain
Chr4_CNVR_41	Chr4	106708225	106716493	8269	-1.206437	0.433338	1.68E-295	Loss	Gain
Chr4_CNVR_42	Chr4	106753709	106765287	11579	-1.25567	0.418799	0	Loss	Gain
Chr4_CNVR_43	Chr4	106767769	106779345	11577	-2.105664	0.232344	0	Loss	Gain
Chr4_CNVR_44	Chr4	106857911	106870315	12405	-1.546059	0.342444	0	Loss	Gain
Chr4_CNVR_8	Chr4	109595281	109606031	10751	1.231035	2.34735	0	Gain	Loss
Chr4_CNVR_45	Chr4	113443313	113452409	9097	-0.9050216	0.534025	4.61E-205	Loss	Gain
Chr4_CNVR_46	Chr4	113453237	113473911	20675	-0.9870072	0.504523	0	Loss	Gain
Chr4_CNVR_47	Chr4	113645927	113655023	9097	-1.230598	0.426141	0	Loss	Gain
Chr4_CNVR_48	Chr4	113656679	113669909	13231	-1.503431	0.352714	0	Loss	Gain
Chr4_CNVR_49	Chr4	113670737	113683141	12405	-0.9922454	0.502695	0	Loss	Gain
Chr4_CNVR_50	Chr4	113692239	113702163	9925	-1.007277	0.497484	9.09E-267	Loss	Gain
Chr4_CNVR_51	Chr4	113950263	113961013	10751	-1.279362	0.411978	0	Loss	Gain
Chr4_CNVR_52	Chr4	114337299	114350531	13233	-0.8039956	0.572761	7.48E-243	Loss	Gain
Chr4_CNVR_53	Chr4	114358801	114415037	56237	-1.081713	0.472468	0	Loss	Gain
Chr4_CNVR_54	Chr4	114415865	114429923	14059	-1.102349	0.465758	0	Loss	Gain
Chr4_CNVR_55	Chr4	114439021	114448117	9097	-1.057594	0.480433	1.08E-264	Loss	Gain
Chr4_CNVR_56	Chr4	114458869	114500217	41349	-1.078966	0.473368	0	Loss	Gain

Chr4_CNVR_57	Chr4	114503527	114515931	12405	-1.031602	0.489167	0	Loss	Gain
Chr4_CNVR_58	Chr4	114525029	114559761	34733	-0.9155917	0.530126	0	Loss	Gain
Chr4_CNVR_59	Chr4	114565551	114593669	28119	-0.9633724	0.512857	0	Loss	Gain
Chr4_CNVR_60	Chr4	114657349	114667271	9923	-0.9146494	0.530473	1.63E-227	Loss	Gain
Chr4_CNVR_61	Chr4	114764031	114776435	12405	-0.8858553	0.541167	5.73E-269	Loss	Gain
Chr4_CNVR_62	Chr4	114990629	115003033	12405	-0.8668504	0.548343	2.99E-259	Loss	Gain
Chr4_CNVR_63	Chr4	115003861	115013785	9925	-0.9620483	0.513328	1.93E-247	Loss	Gain
Chr4_CNVR_64	Chr4	115014613	115023709	9097	-0.8678976	0.547945	4.53E-191	Loss	Gain
Chr4_CNVR_65	Chr4	115051827	115071675	19849	-0.8971311	0.536953	0	Loss	Gain
Chr4_CNVR_66	Chr4	115073329	115089041	15713	-0.9343813	0.523267	0	Loss	Gain
Chr4_CNVR_67	Chr4	115098967	115125429	26463	-0.9066255	0.533431	0	Loss	Gain
Chr4_CNVR_68	Chr4	115126257	115135353	9097	-0.8474752	0.555756	1.75E-183	Loss	Gain
Chr4_CNVR_69	Chr4	115150241	115166779	16539	-0.8764297	0.544714	0	Loss	Gain
Chr4_CNVR_70	Chr4	117543579	117551847	8269	-0.8092347	0.570685	2.80E-154	Loss	Gain
Chr4_CNVR_71	Chr4	117555157	117565079	9923	-0.9563138	0.515372	5.19E-245	Loss	Gain
Chr4_CNVR_72	Chr4	117598987	117608911	9925	-0.8431693	0.557418	3.06E-198	Loss	Gain
Chr4_CNVR_73	Chr4	117651915	117661011	9097	-0.8285967	0.563077	1.53E-176	Loss	Gain
Chr4_CNVR_74	Chr4	117663493	117675897	12405	-1.140034	0.453749	0	Loss	Gain
Chr4_CNVR_75	Chr4	117782581	117794985	12405	-0.8782515	0.544026	4.54E-265	Loss	Gain
Chr4_CNVR_76	Chr4	117829721	117840471	10751	-0.7727345	0.585307	1.53E-184	Loss	Gain
Chr4_CNVR_77	Chr4	117855357	117899187	43831	-0.9218567	0.527829	0	Loss	Gain
Chr4_CNVR_78	Chr4	117909113	117971963	62851	-0.9524096	0.516769	0	Loss	Gain
Chr4_CNVR_79	Chr4	117975273	117986849	11577	-1.019785	0.49319	0	Loss	Gain
Chr4_CNVR_80	Chr4	117990159	118006697	16539	-0.9265838	0.526103	0	Loss	Gain
Chr4_CNVR_81	Chr4	118007525	118028199	20675	-0.8703538	0.547013	0	Loss	Gain
Chr4_CNVR_82	Chr4	118102631	118110899	8269	-0.8258646	0.564144	9.00E-160	Loss	Gain
Chr4_CNVR_83	Chr4	118247355	118258105	10751	-0.9178183	0.529309	9.97E-248	Loss	Gain
Chr4_CNVR_84	Chr4	118267203	118277127	9925	-1.029894	0.489746	1.58E-276	Loss	Gain
Chr4_CNVR_85	Chr4	118292841	118304417	11577	-0.8800515	0.543348	2.11E-248	Loss	Gain
Chr4_CNVR_86	Chr4	118317651	118329227	11577	-0.9802799	0.506881	2.26E-297	Loss	Gain
Chr4_CNVR_87	Chr4	118330883	118349075	18193	-0.8945417	0.537918	0	Loss	Gain
Chr4_CNVR_88	Chr4	118349903	118363961	14059	-0.9402874	0.521129	0	Loss	Gain
Chr4_CNVR_89	Chr4	118369751	118394561	24811	-0.9240529	0.527026	0	Loss	Gain
Chr4_CNVR_90	Chr4	118398697	118408619	9923	-0.8651933	0.548973	3.77E-207	Loss	Gain
Chr4_CNVR_91	Chr4	118409447	118419371	9925	-0.8656828	0.548787	2.39E-207	Loss	Gain
Chr4_CNVR_92	Chr4	118421025	118431775	10751	-0.8829516	0.542257	4.24E-232	Loss	Gain
Chr4_CNVR_93	Chr4	118432603	118445007	12405	-0.9098764	0.532231	2.23E-281	Loss	Gain
Chr4_CNVR_94	Chr4	118800619	118819639	19021	-1.264707	0.416184	0	Loss	Gain
Chr4_CNVR_95	Chr4	119139689	119157055	17367	-0.9081512	0.532868	0	Loss	Gain
Chr4_CNVR_96	Chr4	119161191	119188481	27291	-0.8932426	0.538403	0	Loss	Gain
Chr4_CNVR_97	Chr4	119190135	119199231	9097	-0.9221639	0.527717	1.33E-211	Loss	Gain
Chr4_CNVR_98	Chr4	119385307	119396885	11579	-0.8794539	0.543573	4.08E-248	Loss	Gain
Chr4_CNVR_99	Chr4	119425831	119444023	18193	-0.8933938	0.538346	0	Loss	Gain
Chr4_CNVR_100	Chr4	119450641	119459737	9097	-1.096164	0.467759	3.70E-280	Loss	Gain
Chr4_CNVR_101	Chr4	119474623	119482893	8271	-1.039345	0.486548	3.28E-234	Loss	Gain
Chr4_CNVR_102	Chr4	119485375	119555669	70295	-1.078725	0.473447	0	Loss	Gain
Chr4_CNVR_103	Chr4	119557323	119601981	44659	-0.9438403	0.519847	0	Loss	Gain
Chr4_CNVR_104	Chr4	119605289	119614385	9097	-0.9184056	0.529093	3.63E-210	Loss	Gain
Chr4_CNVR_105	Chr4	119615213	119629271	14059	-0.9543434	0.516076	0	Loss	Gain
Chr4_CNVR_106	Chr4	119643331	119651601	8271	-0.9762292	0.508307	1.40E-211	Loss	Gain
Chr4_CNVR_107	Chr4	119661525	119683027	21503	-1.032318	0.488924	0	Loss	Gain
Chr4_CNVR_108	Chr4	119684681	119703701	19021	-0.9352937	0.522936	0	Loss	Gain
Chr4_CNVR_109	Chr4	119704529	119725203	20675	-1.36017	0.389536	0	Loss	Gain
Chr4_CNVR_110	Chr4	119726031	119745051	19021	-0.975856	0.508438	0	Loss	Gain
Chr4_CNVR_111	Chr4	119746707	119756629	9923	-0.9968436	0.501095	2.76E-262	Loss	Gain
Chr4_CNVR_112	Chr4	119767381	119781439	14059	-0.8997859	0.535966	0	Loss	Gain

Chr4_CNVR_113	Chr4	119823617	119833541	9925	-0.9136158	0.530853	4.38E-227	Loss	Gain
Chr4_CNVR_114	Chr4	119868275	119907143	38869	-0.9061146	0.53362	0	Loss	Gain
Chr4_CNVR_115	Chr4	119910453	119931127	20675	-0.9276752	0.525705	0	Loss	Gain
Chr4_CNVR_116	Chr4	119943533	119961725	18193	-0.9888373	0.503884	0	Loss	Gain
Chr4_CNVR_117	Chr4	119974131	119985709	11579	-0.8730997	0.545973	4.40E-245	Loss	Gain
Chr4_CNVR_118	Chr4	119995633	120036983	41351	-0.981172	0.506568	0	Loss	Gain
Chr4_CNVR_119	Chr4	120043599	120070889	27291	-0.897393	0.536856	0	Loss	Gain
Chr4_CNVR_120	Chr4	120128781	120137877	9097	-0.96389	0.512673	1.03E-227	Loss	Gain
Chr5_CNVR_1	Chr5	4120364	4130792	10429	1.6969	3.24204	0	Gain	Loss
Chr5_CNVR_14	Chr5	26095636	26108670	13035	-0.84499	0.556715	5.29E-248	Loss	Gain
Chr5_CNVR_15	Chr5	26186882	26196440	9559	-0.9525808	0.516707	5.42E-223	Loss	Gain
Chr5_CNVR_16	Chr5	27891860	27900548	8689	-0.8447789	0.556796	5.83E-166	Loss	Gain
Chr5_CNVR_17	Chr5	27917930	27929226	11297	-0.950885	0.517315	2.24E-262	Loss	Gain
Chr5_CNVR_18	Chr5	27955296	27963986	8691	-0.7812846	0.581848	3.88E-145	Loss	Gain
Chr5_CNVR_19	Chr5	27999616	28008304	8689	-0.7770218	0.58357	9.07E-144	Loss	Gain
Chr5_CNVR_20	Chr5	32603578	32613136	9559	-0.7975027	0.575344	3.26E-165	Loss	Gain
Chr5_CNVR_21	Chr5	32620088	32646158	26071	-0.8741217	0.545586	0	Loss	Gain
Chr5_CNVR_22	Chr5	32648766	32658324	9559	-0.8760664	0.544851	6.10E-194	Loss	Gain
Chr5_CNVR_2	Chr5	34572732	34581420	8689	0.8228503	1.7689	1.26E-169	Gain	Loss
Chr5_CNVR_3	Chr5	42119128	42128686	9559	0.8671578	1.82407	2.20E-204	Gain	Loss
Chr5_CNVR_23	Chr5	50326832	50337260	10429	-0.9169219	0.529638	3.54E-228	Loss	Gain
Chr5_CNVR_4	Chr5	51419166	51427854	8689	0.959039	1.94401	2.98E-221	Gain	Loss
Chr5_CNVR_24	Chr5	56529754	56541920	12167	-0.8747273	0.545357	1.27E-245	Loss	Gain
Chr5_CNVR_25	Chr5	56561908	56574942	13035	-1.000873	0.499698	0	Loss	Gain
Chr5_CNVR_26	Chr5	56575812	56594928	19117	-1.013708	0.495272	0	Loss	Gain
Chr5_CNVR_27	Chr5	56597536	56607094	9559	-0.9355333	0.522849	1.93E-216	Loss	Gain
Chr5_CNVR_28	Chr5	56607964	56620130	12167	-0.9406132	0.521011	2.28E-277	Loss	Gain
Chr5_CNVR_29	Chr5	56621000	56629688	8689	-0.9232545	0.527318	1.09E-192	Loss	Gain
Chr5_CNVR_5	Chr5	58130452	58139142	8691	1.089848	2.12852	8.81E-274	Gain	Loss
Chr5_CNVR_6	Chr5	58141750	58153046	11297	2.687238	6.44079	0	Gain	Loss
Chr5_CNVR_30	Chr5	58161736	58170426	8691	-0.8834265	0.542078	5.31E-179	Loss	Gain
Chr5_CNVR_31	Chr5	59112422	59127194	14773	-3.060816	0.11984	0	Loss	Gain
Chr5_CNVR_7	Chr5	68968620	68977310	8691	1.053965	2.07623	3.70E-259	Gain	Loss
Chr5_CNVR_32	Chr5	73932348	73947990	15643	-0.8716238	0.546531	0	Loss	Gain
Chr5_CNVR_33	Chr5	74062698	74073126	10429	-0.8933845	0.53835	1.83E-218	Loss	Gain
Chr5_CNVR_34	Chr5	74632762	74666652	33891	-1.658504	0.316767	0	Loss	Gain
Chr5_CNVR_35	Chr5	74820466	74834370	13905	-0.9339997	0.523405	0	Loss	Gain
Chr5_CNVR_36	Chr5	75911930	75920620	8691	-0.814412	0.56864	6.67E-156	Loss	Gain
Chr5_CNVR_37	Chr5	76186534	76210866	24333	-0.9327747	0.52385	0	Loss	Gain
Chr5_CNVR_38	Chr5	76211736	76225638	13903	-0.8933843	0.53835	1.43E-290	Loss	Gain
Chr5_CNVR_8	Chr5	76418558	76428984	10427	2.999126	7.99515	0	Gain	Loss
Chr5_CNVR_39	Chr5	103676480	103716454	39975	-1.272412	0.413967	0	Loss	Gain
Chr5_CNVR_40	Chr5	104225688	104235246	9559	-0.8441761	0.557029	3.95E-182	Loss	Gain
Chr5_CNVR_41	Chr5	104628904	104637594	8691	-0.8466742	0.556065	1.36E-166	Loss	Gain
Chr5_CNVR_42	Chr5	106850938	106861364	10427	-0.8921065	0.538827	6.13E-218	Loss	Gain
Chr5_CNVR_43	Chr5	107010834	107021260	10427	-0.8717269	0.546492	1.29E-209	Loss	Gain
Chr5_CNVR_44	Chr5	107028214	107055152	26939	-0.9963613	0.501263	0	Loss	Gain
Chr5_CNVR_45	Chr5	107056022	107122064	66043	-1.015627	0.494613	0	Loss	Gain
Chr5_CNVR_46	Chr5	107122934	107151610	28677	-0.9842102	0.505502	0	Loss	Gain
Chr5_CNVR_47	Chr5	107159432	107168990	9559	-0.9815379	0.50644	3.29E-234	Loss	Gain
Chr5_CNVR_48	Chr5	107191586	107202012	10427	-0.8606384	0.550709	4.01E-205	Loss	Gain
Chr5_CNVR_49	Chr5	107257630	107266318	8689	-0.893597	0.53827	1.79E-182	Loss	Gain
Chr5_CNVR_50	Chr5	107589588	107600014	10427	-0.9954012	0.501596	3.31E-261	Loss	Gain
Chr5_CNVR_51	Chr5	107600884	107613050	12167	-0.7861248	0.5799	1.49E-204	Loss	Gain
Chr5_CNVR_52	Chr5	107622610	107632168	9559	-0.8814412	0.542825	5.97E-196	Loss	Gain
Chr5_CNVR_53	Chr5	107637382	107646072	8691	-0.8854457	0.54132	1.09E-179	Loss	Gain

Chr5_CNVR_54	Chr5	107646942	107658238	11297	-0.885681	0.541232	4.51E-233	Loss	Gain
Chr5_CNVR_55	Chr5	107918938	107927628	8691	-0.8935854	0.538275	1.81E-182	Loss	Gain
Chr5_CNVR_56	Chr5	108542880	108554176	11297	-0.835988	0.560199	2.55E-211	Loss	Gain
Chr5_CNVR_57	Chr5	108561998	108576770	14773	-0.8479554	0.555572	1.81E-282	Loss	Gain
Chr5_CNVR_58	Chr5	108941752	108951310	9559	-0.8153906	0.568255	1.21E-171	Loss	Gain
Chr5_CNVR_59	Chr5	109006058	109014746	8689	-0.8508002	0.554477	5.63E-168	Loss	Gain
Chr5_CNVR_60	Chr5	109027782	109037340	9559	-0.9459695	0.519081	1.90E-220	Loss	Gain
Chr5_CNVR_61	Chr5	109038210	109047768	9559	-0.96612	0.511881	3.18E-228	Loss	Gain
Chr5_CNVR_62	Chr5	109056460	109067756	11297	-0.8465513	0.556113	6.75E-216	Loss	Gain
Chr5_CNVR_63	Chr5	109127718	109137276	9559	-0.8749496	0.545273	1.59E-193	Loss	Gain
Chr5_CNVR_64	Chr5	109206796	109217224	10429	-0.8970367	0.536989	5.78E-220	Loss	Gain
Chr5_CNVR_65	Chr5	109242426	109262412	19987	-0.9052661	0.533934	0	Loss	Gain
Chr5_CNVR_66	Chr5	109264150	109278922	14773	-0.9089898	0.532558	0	Loss	Gain
Chr5_CNVR_67	Chr5	109288482	109302386	13905	-0.8401856	0.558572	7.87E-262	Loss	Gain
Chr5_CNVR_68	Chr5	109384942	109396238	11297	-0.9766796	0.508148	3.59E-274	Loss	Gain
Chr5_CNVR_69	Chr5	109397108	109407534	10427	-0.8720535	0.546369	9.49E-210	Loss	Gain
Chr5_CNVR_70	Chr5	109419702	109428390	8689	-1.078196	0.473621	9.26E-248	Loss	Gain
Chr5_CNVR_71	Chr5	109432736	109444032	11297	-0.9000491	0.535868	1.84E-239	Loss	Gain
Chr5_CNVR_72	Chr5	109674318	109685614	11297	-0.8289667	0.562932	2.72E-208	Loss	Gain
Chr5_CNVR_73	Chr5	109689960	109733410	43451	-0.9102138	0.532106	0	Loss	Gain
Chr5_CNVR_74	Chr5	109734280	109750790	16511	-0.9194974	0.528693	0	Loss	Gain
Chr5_CNVR_75	Chr5	109907210	109915900	8691	-0.9029787	0.534781	1.09E-185	Loss	Gain
Chr5_CNVR_76	Chr5	109997586	110006276	8691	-0.925118	0.526637	2.46E-193	Loss	Gain
Chr5_CNVR_77	Chr5	110012360	110022786	10427	-0.9140869	0.53068	5.30E-227	Loss	Gain
Chr5_CNVR_78	Chr5	110023656	110036690	13035	-0.9579228	0.514798	4.13E-306	Loss	Gain
Chr5_CNVR_79	Chr5	110241776	110261762	19987	-0.850109	0.554743	0	Loss	Gain
Chr5_CNVR_80	Chr5	110315640	110326068	10429	-0.8670844	0.548254	9.87E-208	Loss	Gain
Chr5_CNVR_81	Chr5	110351270	110359958	8689	-0.8540951	0.553212	4.42E-169	Loss	Gain
Chr5_CNVR_82	Chr5	110478144	110491178	13035	-0.8976118	0.536775	1.02E-274	Loss	Gain
Chr5_CNVR_83	Chr5	110557222	110569388	12167	-0.8875472	0.540532	9.95E-252	Loss	Gain
Chr5_CNVR_84	Chr5	110866586	110877882	11297	-0.9502489	0.517543	4.36E-262	Loss	Gain
Chr5_CNVR_85	Chr5	110890050	110899608	9559	-0.8937613	0.538209	1.42E-200	Loss	Gain
Chr5_CNVR_86	Chr5	110907430	110917856	10427	-0.8790482	0.543726	1.35E-212	Loss	Gain
Chr5_CNVR_87	Chr5	111361048	111370606	9559	-0.9803257	0.506865	9.73E-234	Loss	Gain
Chr5_CNVR_88	Chr5	111380166	111389724	9559	-0.8415211	0.558055	3.72E-181	Loss	Gain
Chr5_CNVR_89	Chr5	111398414	111410580	12167	-0.9657609	0.512008	1.05E-289	Loss	Gain
Chr5_CNVR_90	Chr5	111425354	111434042	8689	-0.8234455	0.565091	7.08E-159	Loss	Gain
Chr5_CNVR_91	Chr5	111475756	111487920	12165	-0.8362768	0.560087	1.51E-227	Loss	Gain
Chr5_CNVR_92	Chr5	111506170	111522680	16511	-0.8841971	0.541789	0	Loss	Gain
Chr5_CNVR_93	Chr5	111582642	111593938	11297	-0.8664416	0.548498	1.38E-224	Loss	Gain
Chr5_CNVR_94	Chr5	111597416	111613926	16511	-0.8863395	0.540985	0	Loss	Gain
Chr5_CNVR_95	Chr5	111616534	111627830	11297	-0.9651721	0.512217	6.75E-269	Loss	Gain
Chr5_CNVR_96	Chr5	112933068	112945234	12167	-0.8861074	0.541072	4.85E-251	Loss	Gain
Chr5_CNVR_97	Chr5	112993898	113008670	14773	-0.824865	0.564535	2.01E-269	Loss	Gain
Chr5_CNVR_98	Chr5	113012148	113024312	12165	-0.8231595	0.565203	1.82E-221	Loss	Gain
Chr5_CNVR_99	Chr5	113040824	113054728	13905	-0.8409813	0.558264	2.96E-262	Loss	Gain
Chr5_CNVR_100	Chr5	113386686	113395376	8691	-0.7905681	0.578116	3.94E-148	Loss	Gain
Chr5_CNVR_101	Chr5	113406674	113417100	10427	-0.9815877	0.506422	2.50E-255	Loss	Gain
Chr5_CNVR_102	Chr5	113462290	113471848	9559	-0.8594214	0.551174	9.42E-188	Loss	Gain
Chr5_CNVR_103	Chr5	113623924	113632612	8689	-0.8303313	0.5624	3.74E-161	Loss	Gain
Chr5_CNVR_104	Chr5	113780344	113805544	25201	-0.8918597	0.538919	0	Loss	Gain
Chr5_CNVR_105	Chr5	113816842	113826400	9559	-0.9575818	0.514919	6.38E-225	Loss	Gain
Chr5_CNVR_106	Chr5	113835090	113843780	8691	-0.8607337	0.550672	2.59E-171	Loss	Gain
Chr5_CNVR_107	Chr5	113854208	113862898	8691	-0.8720367	0.546375	3.93E-175	Loss	Gain
Chr5_CNVR_108	Chr5	114021056	114031484	10429	-0.9498149	0.517699	6.57E-242	Loss	Gain
Chr5_CNVR_109	Chr5	114509434	114532028	22595	-0.8771037	0.544459	0	Loss	Gain

Chr5_CNVR_110	Chr5	114561574	114578954	17381	-0.9568604	0.515177	0	Loss	Gain
Chr5_CNVR_111	Chr5	114603286	114625880	22595	-1.03562	0.487806	0	Loss	Gain
Chr5_CNVR_112	Chr5	114631094	114662378	31285	-0.9479593	0.518365	0	Loss	Gain
Chr5_CNVR_113	Chr5	114663248	114735374	72127	-0.9877655	0.504258	0	Loss	Gain
Chr5_CNVR_114	Chr5	114763182	114781430	18249	-0.8608928	0.550612	0	Loss	Gain
Chr5_CNVR_115	Chr5	114812716	114825750	13035	-0.8070646	0.571544	3.30E-229	Loss	Gain
Chr5_CNVR_116	Chr5	114850082	114863116	13035	-0.8648034	0.549121	5.58E-258	Loss	Gain
Chr5_CNVR_117	Chr5	115064726	115074284	9559	-0.9566369	0.515257	1.48E-224	Loss	Gain
Chr5_CNVR_118	Chr5	115403636	115413194	9559	-0.9660672	0.5119	3.34E-228	Loss	Gain
Chr5_CNVR_119	Chr5	115460120	115468810	8691	-0.8860205	0.541105	6.94E-180	Loss	Gain
Chr5_CNVR_120	Chr5	115480976	115493142	12167	-0.9331741	0.523705	9.69E-274	Loss	Gain
Chr5_CNVR_121	Chr5	115500964	115516604	15641	-1.041999	0.485654	0	Loss	Gain
Chr5_CNVR_122	Chr5	115517474	115546150	28677	-0.9525494	0.516719	0	Loss	Gain
Chr5_CNVR_123	Chr5	115547020	115565268	18249	-0.9620237	0.513336	0	Loss	Gain
Chr5_CNVR_124	Chr5	115567876	115579172	11297	-0.9906734	0.503243	1.30E-280	Loss	Gain
Chr5_CNVR_125	Chr5	115581780	115590470	8691	-0.9335843	0.523556	2.82E-196	Loss	Gain
Chr5_CNVR_126	Chr5	115667812	115679108	11297	-0.9107055	0.531925	3.17E-244	Loss	Gain
Chr5_CNVR_127	Chr5	115712130	115720820	8691	-0.9424949	0.520332	2.21E-199	Loss	Gain
Chr5_CNVR_128	Chr5	115739938	115756448	16511	-0.9071783	0.533227	0	Loss	Gain
Chr5_CNVR_129	Chr5	115769484	115778174	8691	-0.9062219	0.533581	8.32E-187	Loss	Gain
Chr5_CNVR_130	Chr5	115929380	115942414	13035	-0.9099678	0.532197	4.36E-281	Loss	Gain
Chr5_CNVR_131	Chr5	115945022	115962402	17381	-0.8571915	0.552026	0	Loss	Gain
Chr5_CNVR_132	Chr5	115963272	115975436	12165	-0.9480041	0.518349	5.55E-281	Loss	Gain
Chr5_CNVR_133	Chr5	115983258	115991948	8691	-0.9121142	0.531406	7.76E-189	Loss	Gain
Chr5_CNVR_134	Chr5	115994556	116013672	19117	-0.9975036	0.500866	0	Loss	Gain
Chr5_CNVR_135	Chr5	116015412	116033660	18249	-1.001118	0.499613	0	Loss	Gain
Chr5_CNVR_136	Chr5	116040612	116076240	35629	-0.9496929	0.517743	0	Loss	Gain
Chr5_CNVR_137	Chr5	116080586	116113608	33023	-1.022646	0.492213	0	Loss	Gain
Chr5_CNVR_138	Chr5	116130988	116163140	32153	-0.956358	0.515356	0	Loss	Gain
Chr5_CNVR_139	Chr5	116164010	116187472	23463	-1.005617	0.498057	0	Loss	Gain
Chr5_CNVR_140	Chr5	116189212	116207460	18249	-0.9505755	0.517426	0	Loss	Gain
Chr5_CNVR_141	Chr5	116608070	116616758	8689	-0.78069	0.582088	6.03E-145	Loss	Gain
Chr5_CNVR_142	Chr5	116647174	116662816	15643	-0.9039186	0.534433	0	Loss	Gain
Chr5_CNVR_143	Chr5	116923516	116935682	12167	-0.9653338	0.51216	1.70E-289	Loss	Gain
Chr5_CNVR_144	Chr5	116954800	116968704	13905	-0.9793114	0.507222	0	Loss	Gain
Chr5_CNVR_145	Chr5	116979132	116997380	18249	-0.9572137	0.515051	0	Loss	Gain
Chr5_CNVR_146	Chr5	117018238	117039092	20855	-0.9191872	0.528807	0	Loss	Gain
Chr5_CNVR_147	Chr5	117094710	117105136	10427	-0.9149999	0.530344	2.22E-227	Loss	Gain
Chr5_CNVR_148	Chr5	117120780	117139896	19117	-0.9198027	0.528581	0	Loss	Gain
Chr5_CNVR_9	Chr5	117265034	117283282	18249	1.230747	2.34688	0	Gain	Loss
Chr5_CNVR_10	Chr5	117309352	117320648	11297	1.109775	2.15812	0	Gain	Loss
Chr5_CNVR_11	Chr5	117331946	117475330	143385	1.507757	2.84368	0	Gain	Loss
Chr5_CNVR_12	Chr5	117484022	117502270	18249	1.553651	2.93559	0	Gain	Loss
Chr5_CNVR_13	Chr5	117504878	117514436	9559	1.339571	2.53076	0	Gain	Loss
Chr5_CNVR_149	Chr5	117585694	117637834	52141	-4.290611	0.0510972	0	Loss	Gain
Chr5_CNVR_150	Chr5	117726472	117767314	40843	-0.9423994	0.520367	0	Loss	Gain
Chr5_CNVR_151	Chr5	117775136	117824668	49533	-1.020927	0.4928	0	Loss	Gain
Chr5_CNVR_152	Chr5	117825538	117853346	27809	-0.9922325	0.502699	0	Loss	Gain
Chr5_CNVR_153	Chr5	117855084	117881154	26071	-0.8907373	0.539338	0	Loss	Gain
Chr5_CNVR_154	Chr5	117882892	117906354	23463	-1.002964	0.498974	0	Loss	Gain
Chr5_CNVR_155	Chr5	117908962	117928948	19987	-1.002198	0.499239	0	Loss	Gain
Chr5_CNVR_156	Chr5	117936770	117947198	10429	-0.9175915	0.529392	1.87E-228	Loss	Gain
Chr5_CNVR_157	Chr5	117957626	118001944	44319	-1.03407	0.488331	0	Loss	Gain
Chr5_CNVR_158	Chr5	118002814	118021062	18249	-1.111267	0.462887	0	Loss	Gain
Chr5_CNVR_159	Chr5	118021932	118047132	25201	-1.040815	0.486053	0	Loss	Gain
Chr5_CNVR_160	Chr5	118055824	118067988	12165	-0.9409442	0.520892	1.57E-277	Loss	Gain

Chr5_CNVR_161	Chr5	118075810	118085368	9559	-0.9751923	0.508672	9.64E-232	Loss	Gain
Chr5_CNVR_162	Chr5	118086238	118126212	39975	-0.9747562	0.508826	0	Loss	Gain
Chr5_CNVR_163	Chr5	118174008	118183566	9559	-0.9336761	0.523523	9.92E-216	Loss	Gain
Chr5_CNVR_164	Chr5	118193126	118204422	11297	-0.8944797	0.537941	5.58E-237	Loss	Gain
Chr5_CNVR_165	Chr5	118210506	118222670	12165	-0.8641518	0.549369	1.29E-240	Loss	Gain
Chr5_CNVR_166	Chr5	118246134	118255692	9559	-0.8780486	0.544103	1.11E-194	Loss	Gain
Chr5_CNVR_167	Chr5	118271336	118286108	14773	-0.9547966	0.515914	0	Loss	Gain
Chr5_CNVR_168	Chr5	118303488	118322606	19119	-0.8732302	0.545923	0	Loss	Gain
Chr5_CNVR_169	Chr5	118331296	118345200	13905	-0.9451572	0.519373	0	Loss	Gain
Chr5_CNVR_170	Chr5	118346070	118362580	16511	-0.9708476	0.510206	0	Loss	Gain
Chr5_CNVR_171	Chr5	118406900	118416458	9559	-0.8819723	0.542625	3.78E-196	Loss	Gain
Chr5_CNVR_172	Chr5	119430582	119443616	13035	-0.9754266	0.508589	0	Loss	Gain
Chr5_CNVR_173	Chr5	119465342	119476638	11297	-1.090538	0.469586	0	Loss	Gain
Chr5_CNVR_174	Chr5	119495756	119508790	13035	-1.058814	0.480027	0	Loss	Gain
Chr5_CNVR_175	Chr5	119509660	119543550	33891	-1.046359	0.484189	0	Loss	Gain
Chr5_CNVR_176	Chr5	119544420	119575704	31285	-1.055389	0.481167	0	Loss	Gain
Chr5_CNVR_177	Chr5	119635666	119657390	21725	-0.8679873	0.547911	0	Loss	Gain
Chr5_CNVR_178	Chr5	119673032	119682590	9559	-0.8227184	0.565376	2.70E-174	Loss	Gain
Chr5_CNVR_179	Chr5	119781658	119791216	9559	-0.9125763	0.531236	1.09E-207	Loss	Gain
Chr5_CNVR_180	Chr5	119792954	119802512	9559	-0.973376	0.509313	4.89E-231	Loss	Gain
Chr5_CNVR_181	Chr5	119804252	119816416	12165	-0.8898575	0.539667	7.83E-253	Loss	Gain
Chr5_CNVR_182	Chr5	119822500	119834666	12167	-0.9715207	0.509968	1.51E-292	Loss	Gain
Chr5_CNVR_183	Chr5	119836404	119857260	20857	-1.002348	0.499187	0	Loss	Gain
Chr5_CNVR_184	Chr5	119871164	119890282	19119	-0.9847396	0.505317	0	Loss	Gain
Chr5_CNVR_185	Chr5	119941554	119951980	10427	-0.9664127	0.511777	6.68E-249	Loss	Gain
Chr5_CNVR_186	Chr5	119971100	119982396	11297	-0.9491347	0.517943	1.40E-261	Loss	Gain
Chr5_CNVR_187	Chr5	119987610	120004120	16511	-0.9502336	0.517549	0	Loss	Gain
Chr5_CNVR_188	Chr5	120103188	120112746	9559	-0.820737	0.566153	1.41E-173	Loss	Gain
Chr5_CNVR_189	Chr5	120303058	120318698	15641	-0.8631588	0.549748	0	Loss	Gain
Chr5_CNVR_190	Chr5	120323914	120336078	12165	-0.839148	0.558974	6.95E-229	Loss	Gain
Chr5_CNVR_191	Chr5	120349114	120358672	9559	-0.8837854	0.541944	7.91E-197	Loss	Gain
Chr5_CNVR_192	Chr5	120455132	120463822	8691	-0.922859	0.527463	1.49E-192	Loss	Gain
Chr5_CNVR_193	Chr5	120499452	120508140	8689	-0.9441043	0.519752	6.05E-200	Loss	Gain
Chr5_CNVR_194	Chr5	120520308	120528996	8689	-0.8455223	0.556509	3.29E-166	Loss	Gain
Chr5_CNVR_195	Chr5	120655872	120665430	9559	-0.8445212	0.556896	2.95E-182	Loss	Gain
Chr5_CNVR_196	Chr5	120737558	120746246	8689	-0.9338821	0.523448	2.22E-196	Loss	Gain
Chr5_CNVR_197	Chr5	120747116	120774054	26939	-0.9994171	0.500202	0	Loss	Gain
Chr5_CNVR_198	Chr5	120779270	120787958	8689	-0.9801383	0.506931	1.29E-212	Loss	Gain
Chr5_CNVR_199	Chr5	120791436	120815766	24331	-0.9367925	0.522393	0	Loss	Gain
Chr5_CNVR_200	Chr5	120821850	120861824	39975	-0.9986293	0.500475	0	Loss	Gain
Chr5_CNVR_201	Chr5	120862694	120877466	14773	-0.9025262	0.534949	0	Loss	Gain
Chr5_CNVR_202	Chr5	120888764	120913094	24331	-0.9530911	0.516525	0	Loss	Gain
Chr5_CNVR_203	Chr5	120917440	120926130	8691	-0.9384813	0.521782	5.55E-198	Loss	Gain
Chr5_CNVR_204	Chr5	120927000	120950462	23463	-0.9902443	0.503393	0	Loss	Gain
Chr5_CNVR_205	Chr5	120953938	120973056	19119	-1.122006	0.459455	0	Loss	Gain
Chr5_CNVR_206	Chr5	120985222	120999126	13905	-0.9537553	0.516287	0	Loss	Gain
Chr5_CNVR_207	Chr5	121006078	121015636	9559	-0.9250865	0.526649	1.90E-212	Loss	Gain
Chr7_CNVR_19	Chr7	2637705	2647881	10177	-0.8848338	0.54155	1.32E-214	Loss	Gain
Chr7_CNVR_20	Chr7	3699401	3708729	9329	-0.9281157	0.525544	3.23E-213	Loss	Gain
Chr7_CNVR_21	Chr7	3709577	3719753	10177	-0.9969008	0.501075	2.62E-261	Loss	Gain
Chr7_CNVR_22	Chr7	3730777	3739257	8481	-0.9037846	0.534483	1.20E-185	Loss	Gain
Chr7_CNVR_23	Chr7	3979241	3987721	8481	-0.8655561	0.548835	1.14E-172	Loss	Gain
Chr7_CNVR_24	Chr7	4211593	4221769	10177	-0.9114485	0.531651	1.63E-225	Loss	Gain
Chr7_CNVR_25	Chr7	4237033	4248905	11873	-0.8605449	0.550745	1.53E-238	Loss	Gain
Chr7_CNVR_26	Chr7	4268409	4286217	17809	-0.8545302	0.553045	0	Loss	Gain
Chr7_CNVR_27	Chr7	4397305	4410025	12721	-0.9512502	0.517184	5.00E-302	Loss	Gain

Chr7_CNVR_28	Chr7	4420201	4429529	9329	-0.8866289	0.540876	1.44E-197	Loss	Gain
Chr7_CNVR_29	Chr7	4439705	4448185	8481	-0.9164573	0.529808	5.32E-190	Loss	Gain
Chr7_CNVR_30	Chr7	4450729	4472777	22049	-0.8539823	0.553255	0	Loss	Gain
Chr7_CNVR_31	Chr7	4538073	4548249	10177	-0.895148	0.537692	8.10E-219	Loss	Gain
Chr7_CNVR_32	Chr7	4583865	4592345	8481	-0.7758083	0.584061	3.32E-143	Loss	Gain
Chr7_CNVR_33	Chr7	4606761	4618633	11873	-0.850156	0.554725	1.13E-233	Loss	Gain
Chr7_CNVR_34	Chr7	4619481	4628809	9329	-0.9860972	0.504842	1.65E-235	Loss	Gain
Chr7_CNVR_35	Chr7	4630505	4655097	24593	-0.9783485	0.50756	0	Loss	Gain
Chr7_CNVR_36	Chr7	4685625	4698345	12721	-0.9267733	0.526034	2.80E-289	Loss	Gain
Chr7_CNVR_37	Chr7	4980729	4989209	8481	-0.8442704	0.556992	1.52E-165	Loss	Gain
Chr7_CNVR_38	Chr7	4992601	5001929	9329	-0.9020381	0.53513	2.39E-203	Loss	Gain
Chr7_CNVR_39	Chr7	5006169	5016345	10177	-0.8309648	0.562153	6.03E-193	Loss	Gain
Chr7_CNVR_40	Chr7	5019737	5029065	9329	-0.9751324	0.508693	2.92E-231	Loss	Gain
Chr7_CNVR_41	Chr7	5096057	5109625	13569	-0.8538912	0.55329	9.51E-269	Loss	Gain
Chr7_CNVR_42	Chr7	5289401	5304665	15265	-0.9287181	0.525325	0	Loss	Gain
Chr7_CNVR_43	Chr7	5716793	5728665	11873	-0.9414651	0.520704	2.88E-277	Loss	Gain
Chr7_CNVR_44	Chr7	5741385	5762585	21201	-0.8682119	0.547825	0	Loss	Gain
Chr7_CNVR_45	Chr7	5783785	5795657	11873	-0.8870273	0.540727	4.61E-251	Loss	Gain
Chr7_CNVR_46	Chr7	5813465	5824489	11025	-0.867088	0.548252	1.63E-224	Loss	Gain
Chr7_CNVR_47	Chr7	5899961	5909289	9329	-0.8589527	0.551353	2.74E-187	Loss	Gain
Chr7_CNVR_48	Chr7	6568185	6580905	12721	-0.9096322	0.532321	2.01E-280	Loss	Gain
Chr7_CNVR_49	Chr7	8891705	8904425	12721	-0.9011943	0.535443	4.36E-276	Loss	Gain
Chr7_CNVR_50	Chr7	8911209	8922233	11025	-0.8609741	0.550581	7.66E-222	Loss	Gain
Chr7_CNVR_1	Chr7	11813065	11823241	10177	1.109804	2.15816	0	Gain	Loss
Chr7_CNVR_2	Chr7	11924153	11934329	10177	0.9536195	1.93673	1.37E-264	Gain	Loss
Chr7_CNVR_3	Chr7	11956377	11966553	10177	0.9904091	1.98675	3.55E-282	Gain	Loss
Chr7_CNVR_4	Chr7	12005561	12023369	17809	1.06448	2.09142	0	Gain	Loss
Chr7_CNVR_51	Chr7	12427865	12436345	8481	-0.7978547	0.575204	2.68E-150	Loss	Gain
Chr7_CNVR_52	Chr7	12443977	12452457	8481	-0.8373087	0.559687	3.12E-163	Loss	Gain
Chr7_CNVR_53	Chr7	12595769	12610185	14417	-0.8608794	0.550617	2.17E-289	Loss	Gain
Chr7_CNVR_54	Chr7	12741625	12750953	9329	-0.805075	0.572332	1.06E-167	Loss	Gain
Chr7_CNVR_55	Chr7	12835753	12845929	10177	-0.8570515	0.55208	2.32E-203	Loss	Gain
Chr7_CNVR_56	Chr7	12914617	12926489	11873	-0.8964139	0.53722	1.54E-255	Loss	Gain
Chr7_CNVR_57	Chr7	12945145	12953625	8481	-0.8062063	0.571884	5.20E-153	Loss	Gain
Chr7_CNVR_58	Chr7	12970585	12985849	15265	-0.8729692	0.546022	0	Loss	Gain
Chr7_CNVR_59	Chr7	13524329	13533657	9329	-0.8017285	0.573661	1.66E-166	Loss	Gain
Chr7_CNVR_60	Chr7	13542985	13552313	9329	-0.8834791	0.542059	2.17E-196	Loss	Gain
Chr7_CNVR_61	Chr7	13572665	13587929	15265	-0.9289857	0.525227	0	Loss	Gain
Chr7_CNVR_62	Chr7	13598105	13606585	8481	-1.0724	0.475527	4.28E-245	Loss	Gain
Chr7_CNVR_63	Chr7	13614217	13640505	26289	-0.9732038	0.509374	0	Loss	Gain
Chr7_CNVR_64	Chr7	13650681	13659161	8481	-0.967335	0.51145	1.09E-207	Loss	Gain
Chr7_CNVR_65	Chr7	13667641	13676969	9329	-0.9621955	0.513275	2.88E-226	Loss	Gain
Chr7_CNVR_66	Chr7	13827065	13837241	10177	-0.8414184	0.558095	4.21E-197	Loss	Gain
Chr7_CNVR_67	Chr7	13845721	13854201	8481	-0.9060797	0.533633	1.96E-186	Loss	Gain
Chr7_CNVR_68	Chr7	14577545	14589417	11873	-1.363039	0.388763	0	Loss	Gain
Chr7_CNVR_5	Chr7	15391625	15400953	9329	2.481663	5.58541	0	Gain	Loss
Chr7_CNVR_69	Chr7	15707929	15721497	13569	-1.658069	0.316863	0	Loss	Gain
Chr7_CNVR_70	Chr7	15793577	15805449	11873	-0.836604	0.55996	2.29E-227	Loss	Gain
Chr7_CNVR_71	Chr7	15858873	15867353	8481	-0.7726593	0.585338	3.35E-142	Loss	Gain
Chr7_CNVR_72	Chr7	16056457	16075961	19505	-0.8816124	0.54276	0	Loss	Gain
Chr7_CNVR_73	Chr7	16170937	16181113	10177	-0.7909297	0.577972	2.91E-177	Loss	Gain
Chr7_CNVR_74	Chr7	16182809	16192985	10177	-0.86688	0.548331	2.54E-207	Loss	Gain
Chr7_CNVR_75	Chr7	16547449	16560169	12721	-0.8893788	0.539847	4.83E-270	Loss	Gain
Chr7_CNVR_76	Chr7	17620169	17629497	9329	-0.8668309	0.54835	3.36E-190	Loss	Gain
Chr7_CNVR_77	Chr7	17685465	17694793	9329	-0.8393934	0.558879	4.13E-180	Loss	Gain
Chr7_CNVR_78	Chr7	17866089	17877113	11025	-0.9788533	0.507383	1.27E-274	Loss	Gain

Chr7_CNVR_79	Chr7	17877961	17891529	13569	-0.9744012	0.508951	0	Loss	Gain
Chr7_CNVR_80	Chr7	17894073	17905945	11873	-1.109583	0.463428	0	Loss	Gain
Chr7_CNVR_81	Chr7	17947497	17955977	8481	-0.8507821	0.554484	1.03E-167	Loss	Gain
Chr7_CNVR_82	Chr7	18376585	18399481	22897	-0.9025287	0.534948	0	Loss	Gain
Chr7_CNVR_6	Chr7	18525833	18544489	18657	3.713679	13.1198	0	Gain	Loss
Chr7_CNVR_7	Chr7	18547033	18569081	22049	3.612927	12.2349	0	Gain	Loss
Chr7_CNVR_8	Chr7	18624201	18635225	11025	2.667259	6.35221	0	Gain	Loss
Chr7_CNVR_83	Chr7	19661305	19674025	12721	-0.9059695	0.533674	1.54E-278	Loss	Gain
Chr7_CNVR_84	Chr7	19674873	19694377	19505	-0.8479106	0.555589	0	Loss	Gain
Chr7_CNVR_85	Chr7	19713881	19725753	11873	-0.9153811	0.530204	1.23E-264	Loss	Gain
Chr7_CNVR_86	Chr7	19738473	19752041	13569	-0.8514	0.554247	2.04E-267	Loss	Gain
Chr7_CNVR_87	Chr7	19759673	19776633	16961	-0.8947406	0.537844	0	Loss	Gain
Chr7_CNVR_88	Chr7	19777481	19785961	8481	-0.9492316	0.517908	2.34E-201	Loss	Gain
Chr7_CNVR_89	Chr7	19964889	19975065	10177	-0.7884176	0.578979	2.72E-176	Loss	Gain
Chr7_CNVR_90	Chr7	20168409	20179433	11025	-0.9187464	0.528968	2.15E-247	Loss	Gain
Chr7_CNVR_91	Chr7	20188761	20197241	8481	-0.9951103	0.501698	1.78E-217	Loss	Gain
Chr7_CNVR_92	Chr7	20324441	20334617	10177	-0.9331746	0.523705	1.64E-234	Loss	Gain
Chr7_CNVR_93	Chr7	20336313	20344793	8481	-0.8528688	0.553683	2.06E-168	Loss	Gain
Chr7_CNVR_94	Chr7	20349881	20371081	21201	-0.8890683	0.539963	0	Loss	Gain
Chr7_CNVR_95	Chr7	20437225	20445705	8481	-0.8579091	0.551752	4.23E-170	Loss	Gain
Chr7_CNVR_96	Chr7	20651769	20661945	10177	-0.9276451	0.525716	3.26E-232	Loss	Gain
Chr7_CNVR_97	Chr7	20814585	20823065	8481	-0.9010024	0.535515	1.07E-184	Loss	Gain
Chr7_CNVR_98	Chr7	20829849	20838329	8481	-0.9600384	0.514043	3.92E-205	Loss	Gain
Chr7_CNVR_99	Chr7	20889209	20901081	11873	-0.8397523	0.558739	7.92E-229	Loss	Gain
Chr7_CNVR_100	Chr7	21007929	21018953	11025	-0.9356622	0.522802	5.37E-255	Loss	Gain
Chr7_CNVR_101	Chr7	21023193	21035913	12721	-0.7839547	0.580773	1.08E-217	Loss	Gain
Chr7_CNVR_102	Chr7	21151241	21160569	9329	-0.8598513	0.551009	1.28E-187	Loss	Gain
Chr7_CNVR_103	Chr7	21187705	21196185	8481	-0.8729954	0.546012	3.55E-175	Loss	Gain
Chr7_CNVR_104	Chr7	21268265	21276745	8481	-0.898094	0.536595	1.06E-183	Loss	Gain
Chr7_CNVR_105	Chr7	21278441	21286921	8481	-0.9454428	0.51927	4.88E-200	Loss	Gain
Chr7_CNVR_106	Chr7	21396313	21404793	8481	-0.8154454	0.568233	5.00E-156	Loss	Gain
Chr7_CNVR_107	Chr7	21432777	21442105	9329	-0.9937027	0.502187	1.82E-238	Loss	Gain
Chr7_CNVR_108	Chr7	21470937	21479417	8481	-0.834437	0.560802	2.79E-162	Loss	Gain
Chr7_CNVR_109	Chr7	21520121	21541321	21201	-0.9452347	0.519345	0	Loss	Gain
Chr7_CNVR_110	Chr7	21576089	21584569	8481	-1.262632	0.416783	0	Loss	Gain
Chr7_CNVR_111	Chr7	21589657	21603225	13569	-0.9847453	0.505315	0	Loss	Gain
Chr7_CNVR_112	Chr7	21608313	21624425	16113	-0.9253225	0.526563	0	Loss	Gain
Chr7_CNVR_113	Chr7	21649017	21665977	16961	-0.9409191	0.520901	0	Loss	Gain
Chr7_CNVR_114	Chr7	21678697	21689721	11025	-0.7978434	0.575208	9.12E-195	Loss	Gain
Chr7_CNVR_115	Chr7	21737209	21746537	9329	-0.919754	0.528599	4.84E-210	Loss	Gain
Chr7_CNVR_116	Chr7	21888153	21898329	10177	-0.8629877	0.549813	9.46E-206	Loss	Gain
Chr7_CNVR_117	Chr7	21907657	21916137	8481	-0.8377627	0.559511	2.21E-163	Loss	Gain
Chr7_CNVR_118	Chr7	21943273	21958537	15265	-0.8805926	0.543144	0	Loss	Gain
Chr7_CNVR_119	Chr7	21989913	22000089	10177	-0.8442757	0.55699	3.05E-198	Loss	Gain
Chr7_CNVR_120	Chr7	22006873	22015353	8481	-0.8496017	0.554938	2.54E-167	Loss	Gain
Chr7_CNVR_121	Chr7	22190041	22201913	11873	-0.8630595	0.549785	1.01E-239	Loss	Gain
Chr7_CNVR_122	Chr7	22398649	22407129	8481	-0.8904902	0.539431	4.15E-181	Loss	Gain
Chr7_CNVR_123	Chr7	22430873	22441049	10177	-0.8821333	0.542565	1.66E-213	Loss	Gain
Chr7_CNVR_124	Chr7	22515673	22524153	8481	-0.8503328	0.554657	1.45E-167	Loss	Gain
Chr7_CNVR_125	Chr7	22649657	22662377	12721	-0.8550088	0.552862	1.20E-252	Loss	Gain
Chr7_CNVR_126	Chr7	22673401	22684425	11025	-0.9354394	0.522883	6.76E-255	Loss	Gain
Chr7_CNVR_127	Chr7	22689513	22697993	8481	-0.8448039	0.556786	1.01E-165	Loss	Gain
Chr7_CNVR_128	Chr7	22701385	22713257	11873	-0.825188	0.564409	4.35E-222	Loss	Gain
Chr7_CNVR_129	Chr7	22750569	22760745	10177	-0.9282699	0.525488	1.79E-232	Loss	Gain
Chr7_CNVR_130	Chr7	23829225	23837705	8481	-0.8593308	0.551208	1.41E-170	Loss	Gain
Chr7_CNVR_9	Chr7	27005833	27014313	8481	0.9125426	1.88236	9.23E-205	Gain	Loss

Chr7_CNVR_131	Chr7	28899417	28912985	13569	-0.9006355	0.535651	4.82E-294	Loss	Gain
Chr7_CNVR_10	Chr7	31292473	31308585	16113	3.300652	9.85361	0	Gain	Loss
Chr7_CNVR_11	Chr7	34943961	34952441	8481	0.9133394	1.8834	4.53E-205	Gain	Loss
Chr7_CNVR_132	Chr7	39648665	39660537	11873	-0.8189086	0.566871	3.37E-219	Loss	Gain
Chr7_CNVR_12	Chr7	41842441	41850921	8481	3.79149	13.8469	0	Gain	Loss
Chr7_CNVR_133	Chr7	42482681	42497945	15265	-3.912015	0.0664303	0	Loss	Gain
Chr7_CNVR_13	Chr7	42738777	42794745	55969	1.356263	2.56021	0	Gain	Loss
Chr7_CNVR_134	Chr7	43490953	43501129	10177	-4.333777	0.049591	0	Loss	Gain
Chr7_CNVR_14	Chr7	44003145	44020105	16961	3.393465	10.5084	0	Gain	Loss
Chr7_CNVR_15	Chr7	44434777	44450041	15265	3.110389	8.63615	0	Gain	Loss
Chr7_CNVR_135	Chr7	44723945	44736665	12721	-0.9415839	0.520661	5.59E-297	Loss	Gain
Chr7_CNVR_136	Chr7	44790089	44810441	20353	-0.906984	0.533299	0	Loss	Gain
Chr7_CNVR_137	Chr7	44817225	44828249	11025	-0.9527852	0.516634	9.65E-263	Loss	Gain
Chr7_CNVR_138	Chr7	44836729	44854537	17809	-0.9504073	0.517486	0	Loss	Gain
Chr7_CNVR_139	Chr7	44855385	44879129	23745	-0.9365015	0.522498	0	Loss	Gain
Chr7_CNVR_140	Chr7	44879977	44901177	21201	-0.9821765	0.506215	0	Loss	Gain
Chr7_CNVR_141	Chr7	44913897	44927465	13569	-0.895148	0.537692	4.80E-291	Loss	Gain
Chr7_CNVR_142	Chr7	44944425	44952905	8481	-0.9055869	0.533815	2.89E-186	Loss	Gain
Chr7_CNVR_143	Chr7	44991913	45002937	11025	-0.7893767	0.578594	3.27E-191	Loss	Gain
Chr7_CNVR_144	Chr7	45010569	45028377	17809	-1.008656	0.497009	0	Loss	Gain
Chr7_CNVR_145	Chr7	45031769	45040249	8481	-0.9524929	0.516739	1.70E-202	Loss	Gain
Chr7_CNVR_146	Chr7	45041097	45069929	28833	-0.9195513	0.528673	0	Loss	Gain
Chr7_CNVR_147	Chr7	45083497	45100457	16961	-1.013476	0.495351	0	Loss	Gain
Chr7_CNVR_148	Chr7	45102153	45113177	11025	-0.9403106	0.521121	4.28E-257	Loss	Gain
Chr7_CNVR_149	Chr7	45116569	45130137	13569	-0.8989914	0.536262	3.82E-293	Loss	Gain
Chr7_CNVR_150	Chr7	45139465	45148793	9329	-0.9329464	0.523788	4.67E-215	Loss	Gain
Chr7_CNVR_151	Chr7	45149641	45163209	13569	-0.9430152	0.520145	0	Loss	Gain
Chr7_CNVR_152	Chr7	45164057	45181017	16961	-0.9538648	0.516248	0	Loss	Gain
Chr7_CNVR_153	Chr7	45189497	45208153	18657	-1.012847	0.495567	0	Loss	Gain
Chr7_CNVR_154	Chr7	45209849	45218329	8481	-0.8601952	0.550878	7.24E-171	Loss	Gain
Chr7_CNVR_155	Chr7	45225961	45242073	16113	-0.9654296	0.512126	0	Loss	Gain
Chr7_CNVR_156	Chr7	45242921	45259033	16113	-0.8796704	0.543492	0	Loss	Gain
Chr7_CNVR_157	Chr7	45269209	45280233	11025	-0.9172623	0.529513	9.92E-247	Loss	Gain
Chr7_CNVR_158	Chr7	45293801	45307369	13569	-0.893772	0.538205	2.70E-290	Loss	Gain
Chr7_CNVR_159	Chr7	45308217	45318393	10177	-1.137652	0.454499	0	Loss	Gain
Chr7_CNVR_160	Chr7	45328569	45349769	21201	-0.9777315	0.507778	0	Loss	Gain
Chr7_CNVR_161	Chr7	45366729	45376057	9329	-0.8478615	0.555608	3.29E-183	Loss	Gain
Chr7_CNVR_162	Chr7	45424393	45440505	16113	-0.9332416	0.52368	0	Loss	Gain
Chr7_CNVR_163	Chr7	45443897	45464249	20353	-0.8633738	0.549666	0	Loss	Gain
Chr7_CNVR_164	Chr7	45465945	45483753	17809	-0.9264571	0.526149	0	Loss	Gain
Chr7_CNVR_165	Chr7	45568553	45579577	11025	-0.9315236	0.524304	3.93E-253	Loss	Gain
Chr7_CNVR_166	Chr7	45592297	45600777	8481	-0.9311265	0.524449	4.57E-195	Loss	Gain
Chr7_CNVR_167	Chr7	45607561	45618585	11025	-0.8976518	0.53676	5.48E-238	Loss	Gain
Chr7_CNVR_168	Chr7	45661833	45671161	9329	-0.9234455	0.527248	1.92E-211	Loss	Gain
Chr7_CNVR_169	Chr7	45699993	45708473	8481	-0.8392388	0.558938	7.15E-164	Loss	Gain
Chr7_CNVR_170	Chr7	45769529	45791577	22049	-0.995998	0.501389	0	Loss	Gain
Chr7_CNVR_171	Chr7	45800057	45818713	18657	-0.9897615	0.503561	0	Loss	Gain
Chr7_CNVR_172	Chr7	45848393	45857721	9329	-0.9863973	0.504737	1.26E-235	Loss	Gain
Chr7_CNVR_173	Chr7	52530633	52541657	11025	-0.8652689	0.548944	1.02E-223	Loss	Gain
Chr7_CNVR_174	Chr7	52561161	52570489	9329	-0.9038578	0.534456	4.94E-204	Loss	Gain
Chr7_CNVR_16	Chr7	52606105	52654441	48337	2.651962	6.28521	0	Gain	Loss
Chr7_CNVR_175	Chr7	54598905	54608233	9329	-0.7899993	0.578344	2.48E-162	Loss	Gain
Chr7_CNVR_176	Chr7	63468985	63477465	8481	-0.8258047	0.564167	1.98E-159	Loss	Gain
Chr7_CNVR_177	Chr7	63524105	63532585	8481	-0.8276982	0.563427	4.71E-160	Loss	Gain
Chr7_CNVR_178	Chr7	63533433	63545305	11873	-0.8224392	0.565485	8.02E-221	Loss	Gain
Chr7_CNVR_179	Chr7	63547001	63557177	10177	-0.8438291	0.557163	4.60E-198	Loss	Gain

Chr7_CNVR_180	Chr7	63558873	63568201	9329	-0.8744612	0.545458	4.96E-193	Loss	Gain
Chr7_CNVR_181	Chr7	63621625	63631801	10177	-0.8892942	0.539878	2.00E-216	Loss	Gain
Chr7_CNVR_182	Chr7	63736953	63746281	9329	-0.843745	0.557195	1.06E-181	Loss	Gain
Chr7_CNVR_183	Chr7	63972697	63981177	8481	-0.79985	0.574409	6.05E-151	Loss	Gain
Chr7_CNVR_184	Chr7	70676137	70684617	8481	-2.752578	0.148385	0	Loss	Gain
Chr7_CNVR_17	Chr7	89345705	89355033	9329	1.113673	2.16396	0	Gain	Loss
Chr7_CNVR_18	Chr7	89625545	89634025	8481	0.965839	1.9532	1.09E-225	Gain	Loss
ChrX_CNVR_4	ChrX	37932775	37948747	15973	-0.8446666	0.556839	2.22E-182	Loss	Gain
ChrX_CNVR_5	ChrX	39207631	39229411	21781	-0.9445155	0.519604	2.32E-299	Loss	Gain
ChrX_CNVR_6	ChrX	39602575	39618547	15973	-0.9553198	0.515727	2.79E-224	Loss	Gain
ChrX_CNVR_7	ChrX	39635971	39654847	18877	-1.00188	0.499349	3.69E-286	Loss	Gain
ChrX_CNVR_8	ChrX	39660655	39704215	43561	-1.003309	0.498855	0	Loss	Gain
ChrX_CNVR_9	ChrX	39725995	39746323	20329	-0.828949	0.562939	3.30E-224	Loss	Gain
ChrX_CNVR_10	ChrX	39898783	39923467	24685	-0.9851114	0.505187	0	Loss	Gain
ChrX_CNVR_11	ChrX	40068667	40106419	37753	-0.8956359	0.53751	0	Loss	Gain
ChrX_CNVR_12	ChrX	40430215	40444735	14521	-0.8986455	0.53639	2.47E-184	Loss	Gain
ChrX_CNVR_1	ChrX	43006063	43104799	98737	4.457939	21.9773	0	Gain	Loss
ChrX_CNVR_2	ChrX	44309959	44385463	75505	1.355785	2.55936	0	Gain	Loss
ChrX_CNVR_13	ChrX	56760859	56776831	15973	-1.006269	0.497832	3.41E-244	Loss	Gain
ChrX_CNVR_3	ChrX	94758247	94801807	43561	2.240984	4.72719	0	Gain	Loss
ChrX_CNVR_14	ChrX	95176423	95195299	18877	-1.141328	0.453342	0	Loss	Gain
ChrX_CNVR_15	ChrX	108813607	108828127	14521	-0.8848823	0.541532	1.31E-179	Loss	Gain
ChrX_CNVR_16	ChrX	109027051	109044475	17425	-0.8485017	0.555361	2.58E-200	Loss	Gain
ChrX_CNVR_17	ChrX	118639291	118678495	39205	-2.882401	0.135616	0	Loss	Gain
ChrX_CNVR_18	ChrX	118851283	118871611	20329	-1.101298	0.466097	0	Loss	Gain
ChrX_CNVR_19	ChrX	118925335	118958731	33397	-1.255289	0.41891	0	Loss	Gain
ChrX_CNVR_20	ChrX	118963087	119003743	40657	-1.761754	0.294889	0	Loss	Gain
ChrX_CNVR_21	ChrX	119352223	119368195	15973	-2.423496	0.186404	0	Loss	Gain
ChrX_CNVR_22	ChrX	119382715	119398687	15973	-1.342959	0.394211	0	Loss	Gain
ChrX_CNVR_23	ChrX	128131015	128161507	30493	-0.8955695	0.537535	0	Loss	Gain
ChrX_CNVR_24	ChrX	128215231	128239915	24685	-1.351032	0.392012	0	Loss	Gain
ChrX_CNVR_25	ChrX	140201491	140223271	21781	-0.8342016	0.560893	1.08E-242	Loss	Gain
ChrX_CNVR_26	ChrX	140224723	140243599	18877	-0.854879	0.552912	1.27E-219	Loss	Gain
ChrX_CNVR_27	ChrX	140275543	140291515	15973	-0.8826487	0.542371	1.60E-196	Loss	Gain
ChrX_CNVR_28	ChrX	144438427	144455851	17425	-0.8080748	0.571144	3.29E-184	Loss	Gain
ChrX_CNVR_29	ChrX	144646063	144660583	14521	-0.8829477	0.542258	6.01E-179	Loss	Gain
ChrX_CNVR_30	ChrX	148804591	148824919	20329	-0.9403816	0.521095	1.61E-277	Loss	Gain
