# Supplementary material for: Whole-Genome Analyses of Korean Native and Holstein Cattle Breeds by Massively Parallel Sequencing
Source: PLoS One. 2014 Jul 3;9(7):e101127. doi: 10.1371/journal.pone.0101127 (PMC4081042; doi:10.1371/journal.pone.0101127)
Supplement: Table S7 — Gene Ontology terms enriched among the top 100 genes containing the highest number of nsSNPs in each of breed-specific nsSNPs. HAN, JJH, CHS, and HOL indicate Hanwoo, Jeju Heugu, Chikso, and Korean Holstein respectively. (PDF) [file pone.0101127.s010.pdf]

Supplementary Table S7. Gene Ontology terms enriched among the top 100 genes containing the highest number of nsSNPs in each of breed-specific nsSNPs. HAN, JJH, CHS, and HOL indicate Hanwoo, Jeju Heugu, Chikso, and Korean Holstein respectively.

GO term	Breed				Description	P-value			
						P-HAN	P-JJH	P-CHS	P-HOL
GO:0032502	HAN	JJH	CHS	HOL	developmental process	4.50E-53	4.20E-29	1.40E-19	3.20E-22
GO:0032501	HAN	JJH	CHS	HOL	multicellular organismal process	9.50E-69	1.20E-48	6.70E-61	1.80E-59
GO:0002376	HAN	JJH	CHS	HOL	immune system process	3.00E-25	1.20E-14	4.00E-06	1.70E-14
GO:0000003	HAN	JJH	CHS	HOL	reproduction	8.70E-14	4.00E-09	5.20E-09	1.30E-10
GO:0016043	HAN	JJH	CHS	HOL	cellular component organization	3.70E-13	9.60E-07	6.20E-08	1.30E-06
GO:0048518	HAN	JJH	CHS	HOL	positive regulation of biological process	1.50E-35	4.90E-15	5.20E-11	5.40E-17
GO:0048519	HAN	JJH	CHS	HOL	negative regulation of biological process	8.90E-26	3.80E-24	5.00E-11	1.60E-14
GO:0022610	HAN	JJH	CHS	HOL	biological adhesion	4.70E-08	2.40E-12	2.30E-06	2.10E-06
GO:0022414	HAN	JJH	CHS	HOL	reproductive process	8.70E-14	4.00E-09	5.20E-09	1.50E-07
GO:0051179	HAN	JJH	CHS	HOL	localization	9.70E-11	1.80E-05	7.80E-05	8.50E-06
GO:0040011	HAN	JJH	CHS	HOL	locomotion	2.00E-23	5.10E-09	4.70E-11	2.50E-13
GO:0050896	HAN	JJH	CHS	HOL	response to stimulus	5.90E-35	1.30E-32	5.60E-30	8.50E-39
GO:0016265	HAN	JJH	-	HOL	death	1.00E-06	2.10E-09	-	4.80E-08
GO:0051234	HAN	JJH	-	HOL	establishment of localization	1.20E-08	0.00078	-	0.001
GO:0040007	HAN	-	-	HOL	growth	1.80E-22	-	-	4.60E-12
GO:0050789	HAN	-	-	-	regulation of biological process	1.30E-05	-	-	-
GO:0065007	HAN	-	-	-	biological regulation	7.40E-06	-	-	-
GO:0009987	HAN	-	-	-	cellular process	0.0074	-	-	-
GO:0008152	HAN	-	-	-	metabolic process	0.0011	-	-	-
GO:0044085	HAN	-	-	-	cellular component biogenesis	0.006	-	-	-
