# Supplementary material for: Whole-Genome Analyses of Korean Native and Holstein Cattle Breeds by Massively Parallel Sequencing
Source: PLoS One. 2014 Jul 3;9(7):e101127. doi: 10.1371/journal.pone.0101127 (PMC4081042; doi:10.1371/journal.pone.0101127)
Supplement: Table S8 — Gene Ontology terms enriched among the genic-CNVRs from HANvsHOL. (PDF) [file pone.0101127.s011.pdf]

Supplementary Table S8. Gene Ontology terms enriched among the genic-CNVRs from HANvsHOL.

Go term	Ontology	Breed of Gain	Description	P-HAN	P-HOL
**Biological Process**					
GO:0032502	P	HAN/HOL	developmental process	1.10E-22	3.10E-191
GO:0032501	P	HAN/HOL	multicellular organismal process	3.60E-53	2.80E-259
GO:0002376	P	HAN/HOL	immune system process	6.20E-21	6.30E-30
GO:0016043	P	HAN/HOL	cellular component organization	3.70E-08	1.80E-71
GO:0065007	P	HAN/HOL	biological regulation	5.30E-03	8.50E-16
GO:0016265	P	HAN/HOL	death	2.30E-04	4.10E-42
GO:0051234	P	HAN/HOL	establishment of localization	1.10E-06	1.30E-21
GO:0051179	P	HAN/HOL	localization	1.40E-07	6.10E-27
GO:0040007	P	HAN/HOL	growth	8.40E-06	9.80E-61
GO:0050896	P	HAN/HOL	response to stimulus	9.80E-51	2.10E-79
GO:0044085	P	HAN/HOL	cellular component biogenesis	5.50E-04	3.90E-18
GO:0000003	P	HAN	reproduction	6.70E-12	-
GO:0048518	P	HAN	positive regulation of biological process	1.60E-40	-
GO:0048519	P	HAN	negative regulation of biological process	6.80E-16	-
GO:0022414	P	HAN	reproductive process	9.00E-09	-
GO:0040011	P	HAN	locomotion	2.30E-15	-
GO:0050789	P	HOL	regulation of biological process	-	2.90E-13
GO:0022610	P	HOL	biological adhesion	-	3.10E-14
GO:0009987	P	HOL	cellular process	-	1.30E-06
GO:0008152	P	HOL	metabolic process	-	5.00E-08
**Molecular Function**					
GO:0060089	F	HAN/HOL	molecular transducer activity	1.80E-05	6.40E-05
GO:0005215	F	HOL	transporter activity	-	2.50E-05
GO:0030528	F	HOL	transcription regulator activity	-	3.10E-08
GO:0003824	F	HOL	catalytic activity	-	0.0009
GO:0005488	F	HOL	binding	-	2.30E-10
GO:0030234	F	HOL	enzyme regulator activity	-	4.40E-06
**Cellular Component**					
GO:0032991	C	HAN/HOL	macromolecular complex	0.00061	2.10E-09
GO:0005623	C	HAN/HOL	cell	0.0014	2.00E-11
GO:0044464	C	HAN/HOL	cell part	1.40E-03	2.00E-11
GO:0031974	C	HAN/HOL	membrane-enclosed lumen	1.20E-07	3.30E-47
GO:0044421	C	HAN/HOL	extracellular region part	2.40E-12	6.70E-59
GO:0005576	C	HAN/HOL	extracellular region	6.90E-09	1.50E-26
GO:0045202	C	HAN/HOL	synapse	8.80E-10	3.30E-08
GO:0044456	C	HAN/HOL	synapse part	1.40E-08	3.10E-03
GO:0043226	C	HAN/HOL	organelle	2.60E-04	3.00E-39
GO:0044422	C	HAN/HOL	organelle part	4.30E-04	8.90E-46
