# Supplementary material for: Whole-Genome Analyses of Korean Native and Holstein Cattle Breeds by Massively Parallel Sequencing
Source: PLoS One. 2014 Jul 3;9(7):e101127. doi: 10.1371/journal.pone.0101127 (PMC4081042; doi:10.1371/journal.pone.0101127)
Supplement: Table S9 — Gene Ontology terms enriched among the genic-CNVRs from JJHvsHOL. (PDF) [file pone.0101127.s012.pdf]

Supplementary Table S9. Gene Ontology terms enriched among the genic-CNVRs from JJHvsHOL.

Go term	Ontology	Breed of Gain	Description	P-JJB	P-HOL
**Biological Process**					
GO:0032502	P	JJH/HOL	developmental process	7.10E-08	1.10E-06
GO:0032501	P	JJH/HOL	multicellular organismal process	3.10E-30	1.20E-15
GO:0002376	P	JJH/HOL	immune system process	1.20E-18	4.20E-17
GO:0016043	P	JJH/HOL	cellular component organization	4.10E-03	3.00E-04
GO:0048518	P	JJH/HOL	positive regulation of biological process	1.80E-23	3.00E-10
GO:0048519	P	JJH/HOL	negative regulation of biological process	2.70E-07	2.00E-12
GO:0051179	P	JJH/HOL	localization	1.10E-04	3.40E-03
GO:0050896	P	JJH/HOL	response to stimulus	2.60E-31	4.10E-23
GO:0065007	P	JJH	biological regulation	1.80E-03	-
GO:0016265	P	JJH	death	1.80E-05	-
GO:0051234	P	JJH	establishment of localization	9.20E-05	-
GO:0022610	P	HOL	biological adhesion	-	0.00037
**Molecular Function**					
GO:0060089	F	JJB	molecular transducer activity	9.90E-05	-
**Cellular Component**					
GO:0005623	C	JJH/HOL	cell	0.0018	3.10E-03
GO:0044464	C	JJH/HOL	cell part	0.0018	3.10E-03
GO:0044421	C	JJH/HOL	extracellular region part	1.40E-09	2.40E-16
GO:0005576	C	JJH/HOL	extracellular region	1.70E-06	3.00E-11
GO:0031974	C	HOL	membrane-enclosed lumen	-	1.30E-07
GO:0043226	C	HOL	organelle	-	6.30E-03
GO:0044422	C	HOL	organelle part	-	5.50E-03
