# Supplementary material for: Whole-Genome Analyses of Korean Native and Holstein Cattle Breeds by Massively Parallel Sequencing
Source: PLoS One. 2014 Jul 3;9(7):e101127. doi: 10.1371/journal.pone.0101127 (PMC4081042; doi:10.1371/journal.pone.0101127)
Supplement: Table S10 — Gene Ontology terms enriched among the genic-CNVRs from CHSvsHOL. (PDF) [file pone.0101127.s013.pdf]

Supplementary Table S10. Gene Ontology terms enriched among the genic-CNVRs from CHSvsHOL.

Go term	Ontology	Breed of Gain	Description	P-CHS	P-HOL
**Biological Process**					
GO:0002376	P	CHS/HOL	immune system process	1.20E-15	6.60E-86
GO:0016043	P	CHS/HOL	cellular component organization	5.10E-11	2.10E-263
GO:0065007	P	CHS/HOL	biological regulation	2.20E-03	1.00E-46
GO:0051234	P	CHS/HOL	establishment of localization	7.60E-07	3.90E-75
GO:0051179	P	CHS/HOL	localization	9.10E-08	2.00E-104
GO:0050896	P	CHS/HOL	response to stimulus	2.80E-43	8.90E-291
GO:0044085	P	CHS/HOL	cellular component biogenesis	2.90E-04	2.80E-56
GO:0032502	P	CHS	developmental process	1.70E-27	-
GO:0032501	P	CHS	multicellular organismal process	1.90E-51	-
GO:0000003	P	CHS	reproduction	1.90E-17	-
GO:0048518	P	CHS	positive regulation of biological process	1.70E-38	-
GO:0048519	P	CHS	negative regulation of biological process	5.20E-22	-
GO:0016265	P	CHS	death	1.50E-04	-
GO:0022414	P	CHS	reproductive process	1.30E-10	-
GO:0040007	P	CHS	growth	1.90E-10	-
GO:0040011	P	CHS	locomotion	2.50E-13	-
GO:0050789	P	HOL	regulation of biological process	-	1.50E-42
GO:0022610	P	HOL	biological adhesion	-	4.90E-31
GO:0009987	P	HOL	cellular process	-	1.30E-20
GO:0008152	P	HOL	metabolic process	-	1.00E-29
**Molecular Function**					
GO:0060089	F	CHS/HOL	molecular transducer activity	0.00043	4.70E-04
GO:0005215	F	HOL	transporter activity	-	4.30E-09
GO:0016209	F	HOL	antioxidant activity	-	1.30E-03
GO:0030528	F	HOL	transcription regulator activity	-	2.20E-15
GO:0005198	F	HOL	structural molecule activity	-	2.30E-08
GO:0003824	F	HOL	catalytic activity	-	3.50E-10
GO:0005488	F	HOL	binding	-	4.10E-29
GO:0030234	F	HOL	enzyme regulator activity	-	3.90E-19
GO:0009055	F	HOL	electron carrier activity	-	1.80E-04
**Cellular Component**					
GO:0032991	C	CHS/HOL	macromolecular complex	0.0002	3.70E-40
GO:0005623	C	CHS/HOL	cell	0.00042	6.80E-30
GO:0044464	C	CHS/HOL	cell part	4.20E-04	6.80E-30
GO:0005576	C	CHS/HOL	extracellular region	1.00E-04	6.00E-76
GO:0045202	C	CHS/HOL	synapse	3.50E-06	1.80E-36
GO:0043226	C	CHS/HOL	organelle	6.40E-06	9.00E-119
GO:0044422	C	CHS/HOL	organelle part	8.60E-08	5.40E-139
GO:0031974	C	CHS	membrane-enclosed lumen	2.10E-15	-
GO:0044421	C	CHS	extracellular region part	5.50E-08	-
GO:0044456	C	HOL	synapse part	-	5.90E-20
