# Supplementary material for: Whole-Genome Analyses of Korean Native and Holstein Cattle Breeds by Massively Parallel Sequencing
Source: PLoS One. 2014 Jul 3;9(7):e101127. doi: 10.1371/journal.pone.0101127 (PMC4081042; doi:10.1371/journal.pone.0101127)
Supplement: Table S11 — The nsSNPs identified in Hanwoo, Jeju Heugu, Chikso, and Korean Holstein that overlap with pigmentation-related genes. TYR, TYRP1, DCT, and MC1R indicate tyrosinase, tyrosinase-related protein 1, dopachrome tautomerase, and melanocortin 1 receptor respectively. (PDF) [file pone.0101127.s014.pdf]

Supplementary Table S11. The nsSNPs identified in the pigmentation-related genes from total nsSNP sets from Hanwoo, Jeju Heugu, Chikso, and Korean Holstein. The TYR, TYRP1, DCT, and MC1R indicate tyrosinase, tyrosinase-related protein 1, dopachrome tautomerase, and melanocortin 1 receptor respectively.

Breed	TYR	TYRP1	DCT	MC1R
**Hanwoo**		Chr8:31717680 (T>C: Heterozygous)	Chr12:69507694 (T>C: Heterozygous)<br>Chr12:69532190 (T>C: Heterozygous)<br>Chr12:69534625 (A>G: Heterozygous)	
**Jeju Heugu**			Chr12:69534625 (A>G: Heterozygous)	Chr18:14758485 (G>A: Heterozygous)
**Korean Holstein**			Chr12:69527675 (C>T: Heterozygous)<br>Chr12:69534625 (A>G: Heterozygous)<br>Chr12:69544526 (A>G: Heterozygous)	Chr18:14757910 (T>C: Homozygous)
**Chikso**	Chr29:6461851 (G>A: Heterozygous)	Chr8:31717680 (T>C: Heterozygous)	Chr12:69544299 (G>C: Heterozygous)	
