# Supplementary material for: Whole-Genome Analyses of Korean Native and Holstein Cattle Breeds by Massively Parallel Sequencing
Source: PLoS One. 2014 Jul 3;9(7):e101127. doi: 10.1371/journal.pone.0101127 (PMC4081042; doi:10.1371/journal.pone.0101127)
Supplement: Table S12 — Regions of homozygosity (ROHs) detected from Hanwoo, Jeju Heugu, Chikso, and Korean Holstein in this study. (PDF) [file pone.0101127.s015.pdf]

Supplementary Table S12. Regions of homozygosity (ROHs) detected from Hanwoo, Jeju Heugu, Chikso, and Korean Holstein in this study.

Hanwoo

Region_Name	Chr	Bin_Start	Bin_End	Size
HAN_ROH_1	Chr1	52,800,001	53,200,000	400,000
HAN_ROH_2	Chr2	12,400,001	19,200,000	6,800,000
HAN_ROH_3	Chr2	19,600,001	32,000,000	12,400,000
HAN_ROH_4	Chr2	48,000,001	48,800,000	800,000
HAN_ROH_5	Chr2	62,000,001	62,400,000	400,000
HAN_ROH_6	Chr2	64,400,001	100,000,000	35,600,000
HAN_ROH_7	Chr2	106,000,001	109,200,000	3,200,000
HAN_ROH_8	Chr3	20,400,001	20,800,000	400,000
HAN_ROH_9	Chr5	21,600,001	28,400,000	6,800,000
HAN_ROH_10	Chr5	75,600,001	76,000,000	400,000
HAN_ROH_11	Chr5	92,400,001	92,800,000	400,000
HAN_ROH_12	Chr6	1	1,200,000	1,200,000
HAN_ROH_13	Chr6	16,800,001	18,800,000	2,000,000
HAN_ROH_14	Chr6	64,000,001	64,400,000	400,000
HAN_ROH_15	Chr6	116,800,001	117,200,000	400,000
HAN_ROH_16	Chr7	86,800,001	87,600,000	800,000
HAN_ROH_17	Chr8	103,600,001	104,400,000	800,000
HAN_ROH_18	Chr9	20,000,001	20,400,000	400,000
HAN_ROH_19	Chr9	63,600,001	64,800,000	1,200,000
HAN_ROH_20	Chr10	12,000,001	12,400,000	400,000
HAN_ROH_21	Chr10	35,600,001	36,400,000	800,000
HAN_ROH_22	Chr10	38,800,001	39,200,000	400,000
HAN_ROH_23	Chr10	49,600,001	50,400,000	800,000
HAN_ROH_24	Chr10	58,400,001	58,800,000	400,000
HAN_ROH_25	Chr10	71,600,001	72,400,000	800,000
HAN_ROH_26	Chr11	17,600,001	18,000,000	400,000
HAN_ROH_27	Chr11	61,200,001	62,000,000	800,000
HAN_ROH_28	Chr11	71,600,001	72,000,000	400,000
HAN_ROH_29	Chr12	400,001	2,800,000	2,400,000
HAN_ROH_30	Chr12	40,800,001	42,000,000	1,200,000
HAN_ROH_31	Chr12	44,000,001	44,800,000	800,000
HAN_ROH_32	Chr12	56,800,001	58,000,000	1,200,000
HAN_ROH_33	Chr13	38,400,001	53,600,000	15,200,000
HAN_ROH_34	Chr13	54,000,001	58,000,000	4,000,000
HAN_ROH_35	Chr14	46,800,001	48,000,000	1,200,000
HAN_ROH_36	Chr15	18,000,001	18,400,000	400,000
HAN_ROH_37	Chr15	35,600,001	36,000,000	400,000
HAN_ROH_38	Chr16	72,000,001	76,800,000	4,800,000
HAN_ROH_39	Chr17	6,000,001	6,800,000	800,000
HAN_ROH_40	Chr17	54,400,001	54,800,000	400,000
HAN_ROH_41	Chr19	1,600,001	2,000,000	400,000

HAN_ROH_42	Chr19	2,800,001	3,200,000	400,000
HAN_ROH_43	Chr19	44,800,001	45,200,000	400,000
HAN_ROH_44	Chr20	47,200,001	47,600,000	400,000
HAN_ROH_45	Chr21	400,001	800,000	400,000
HAN_ROH_46	Chr21	2,000,001	10,400,000	8,400,000
HAN_ROH_47	Chr22	16,400,001	16,800,000	400,000
HAN_ROH_48	Chr22	60,800,001	61,200,000	400,000
HAN_ROH_49	Chr23	1	1,600,000	1,600,000
HAN_ROH_50	Chr24	28,800,001	29,200,000	400,000
HAN_ROH_51	Chr24	40,800,001	41,600,000	800,000
HAN_ROH_52	Chr24	46,400,001	56,800,000	10,400,000
HAN_ROH_53	Chr28	18,000,001	26,000,000	8,000,000

Jeju Heugu

Region_Name	Chr	Bin_Start	Bin_End	Size
JJH_ROH_1	Chr1	54,800,001	55,200,000	400,000
JJH_ROH_2	Chr1	70,400,001	70,800,000	400,000
JJH_ROH_3	Chr1	102,800,001	103,600,000	800,000
JJH_ROH_4	Chr2	24,400,001	38,000,000	13,600,000
JJH_ROH_5	Chr2	48,000,001	48,400,000	400,000
JJH_ROH_6	Chr2	89,200,001	89,600,000	400,000
JJH_ROH_7	Chr2	91,200,001	92,000,000	800,000
JJH_ROH_8	Chr2	96,400,001	100,400,000	4,000,000
JJH_ROH_9	Chr2	111,600,001	112,000,000	400,000
JJH_ROH_10	Chr3	94,800,001	95,200,000	400,000
JJH_ROH_11	Chr3	102,800,001	109,200,000	6,400,000
JJH_ROH_12	Chr4	78,400,001	78,800,000	400,000
JJH_ROH_13	Chr4	95,600,001	96,000,000	400,000
JJH_ROH_14	Chr5	13,600,001	21,600,000	8,000,000
JJH_ROH_15	Chr5	70,000,001	70,800,000	800,000
JJH_ROH_16	Chr5	80,800,001	81,600,000	800,000
JJH_ROH_17	Chr5	94,800,001	99,200,000	4,400,000
JJH_ROH_18	Chr5	100,000,001	102,000,000	2,000,000
JJH_ROH_19	Chr5	103,600,001	114,000,000	10,400,000
JJH_ROH_20	Chr6	51,200,001	51,600,000	400,000
JJH_ROH_21	Chr7	47,200,001	48,000,000	800,000
JJH_ROH_22	Chr7	52,800,001	53,600,000	800,000
JJH_ROH_23	Chr7	66,000,001	66,400,000	400,000
JJH_ROH_24	Chr8	8,800,001	22,000,000	13,200,000
JJH_ROH_25	Chr8	23,600,001	37,200,000	13,600,000
JJH_ROH_26	Chr8	38,000,001	42,400,000	4,400,000
JJH_ROH_27	Chr8	92,400,001	94,400,000	2,000,000
JJH_ROH_28	Chr9	2,400,001	4,000,000	1,600,000
JJH_ROH_29	Chr9	10,400,001	19,200,000	8,800,000
JJH_ROH_30	Chr9	26,400,001	34,800,000	8,400,000
JJH_ROH_31	Chr9	35,200,001	42,000,000	6,800,000
JJH_ROH_32	Chr9	42,400,001	62,000,000	19,600,000
JJH_ROH_33	Chr9	77,600,001	78,000,000	400,000
JJH_ROH_34	Chr10	44,800,001	58,800,000	14,000,000
JJH_ROH_35	Chr11	14,000,001	46,800,000	32,800,000

JJH_ROH_36	Chr11	47,200,001	48,400,000	1,200,000
JJH_ROH_37	Chr11	50,000,001	50,400,000	400,000
JJH_ROH_38	Chr11	57,600,001	58,000,000	400,000
JJH_ROH_39	Chr11	76,400,001	91,200,000	14,800,000
JJH_ROH_40	Chr12	43,200,001	44,000,000	800,000
JJH_ROH_41	Chr12	60,800,001	61,200,000	400,000
JJH_ROH_42	Chr13	83,600,001	84,240,350	640,350
JJH_ROH_43	Chr14	16,400,001	16,800,000	400,000
JJH_ROH_44	Chr14	52,000,001	52,400,000	400,000
JJH_ROH_45	Chr15	1,600,001	2,400,000	800,000
JJH_ROH_46	Chr17	800,001	1,200,000	400,000
JJH_ROH_47	Chr17	6,400,001	6,800,000	400,000
JJH_ROH_48	Chr17	29,600,001	30,000,000	400,000
JJH_ROH_49	Chr20	23,200,001	23,600,000	400,000
JJH_ROH_50	Chr20	38,000,001	38,800,000	800,000
JJH_ROH_51	Chr20	50,800,001	51,200,000	400,000
JJH_ROH_52	Chr21	1	400,000	400,000
JJH_ROH_53	Chr21	2,400,001	6,000,000	3,600,000
JJH_ROH_54	Chr21	6,400,001	10,400,000	4,000,000
JJH_ROH_55	Chr22	42,400,001	43,200,000	800,000
JJH_ROH_56	Chr23	34,400,001	34,800,000	400,000
JJH_ROH_57	Chr24	50,000,001	50,400,000	400,000
JJH_ROH_58	Chr26	2,000,001	2,800,000	800,000
JJH_ROH_59	Chr26	17,600,001	18,400,000	800,000
JJH_ROH_60	Chr26	22,000,001	22,400,000	400,000
JJH_ROH_61	Chr26	44,800,001	47,600,000	2,800,000
JJH_ROH_62	Chr27	42,800,001	45,407,902	2,607,902
JJH_ROH_63	Chr28	1	4,000,000	4,000,000
JJH_ROH_64	Chr28	4,400,001	18,000,000	13,600,000
JJH_ROH_65	Chr29	11,200,001	16,000,000	4,800,000

Chikso

**Region_Name**	**Chr**	**Bin_Start**	**Bin_End**	**Size**
CHS_ROH_1	Chr1	37,600,001	38,000,000	400,000
CHS_ROH_2	Chr1	54,400,001	54,800,000	400,000
CHS_ROH_3	Chr1	60,000,001	60,400,000	400,000
CHS_ROH_4	Chr1	71,600,001	72,400,000	800,000
CHS_ROH_5	Chr1	103,600,001	104,400,000	800,000
CHS_ROH_6	Chr2	42,000,001	42,400,000	400,000
CHS_ROH_7	Chr2	44,800,001	45,200,000	400,000
CHS_ROH_8	Chr2	62,000,001	62,400,000	400,000
CHS_ROH_9	Chr3	50,400,001	51,600,000	1,200,000
CHS_ROH_10	Chr4	2,400,001	2,800,000	400,000
CHS_ROH_11	Chr4	4,400,001	4,800,000	400,000
CHS_ROH_12	Chr4	34,800,001	35,200,000	400,000
CHS_ROH_13	Chr4	53,600,001	54,400,000	800,000
CHS_ROH_14	Chr5	17,200,001	17,600,000	400,000
CHS_ROH_15	Chr5	43,200,001	43,600,000	400,000
CHS_ROH_16	Chr5	45,600,001	46,400,000	800,000
CHS_ROH_17	Chr5	80,800,001	81,200,000	400,000
CHS_ROH_18	Chr6	78,400,001	78,800,000	400,000

CHS_ROH_19	Chr6	92,000,001	92,800,000	800,000
CHS_ROH_20	Chr7	21,200,001	21,600,000	400,000
CHS_ROH_21	Chr7	34,800,001	35,200,000	400,000
CHS_ROH_22	Chr7	56,800,001	57,600,000	800,000
CHS_ROH_23	Chr7	78,800,001	81,200,000	2,400,000
CHS_ROH_24	Chr7	82,400,001	83,200,000	800,000
CHS_ROH_25	Chr8	57,200,001	57,600,000	400,000
CHS_ROH_26	Chr9	19,600,001	20,000,000	400,000
CHS_ROH_27	Chr10	52,400,001	53,200,000	800,000
CHS_ROH_28	Chr10	104,000,001	104,305,016	305,016
CHS_ROH_29	Chr11	56,800,001	58,400,000	1,600,000
CHS_ROH_30	Chr12	38,800,001	39,200,000	400,000
CHS_ROH_31	Chr12	42,000,001	42,400,000	400,000
CHS_ROH_32	Chr12	43,600,001	44,000,000	400,000
CHS_ROH_33	Chr13	84,000,001	84,240,350	240,350
CHS_ROH_34	Chr14	71,600,001	72,000,000	400,000
CHS_ROH_35	Chr15	2,400,001	2,800,000	400,000
CHS_ROH_36	Chr17	44,800,001	45,200,000	400,000
CHS_ROH_37	Chr18	52,800,001	53,200,000	400,000
CHS_ROH_38	Chr19	800,001	1,600,000	800,000
CHS_ROH_39	Chr20	39,200,001	39,600,000	400,000
CHS_ROH_40	Chr20	46,800,001	48,000,000	1,200,000
CHS_ROH_41	Chr20	65,200,001	66,000,000	800,000
CHS_ROH_42	Chr21	32,400,001	32,800,000	400,000
CHS_ROH_43	Chr21	34,400,001	34,800,000	400,000
CHS_ROH_44	Chr23	400,001	1,200,000	800,000
CHS_ROH_45	Chr27	1	400,000	400,000

Korean Holstein

Region_Name	Chr	Bin_Start	Bin_End	Size
HOL_ROH_1	Chr1	47,600,001	50,400,000	2,800,000
HOL_ROH_2	Chr1	70,400,001	70,800,000	400,000
HOL_ROH_3	Chr1	82,400,001	82,800,000	400,000
HOL_ROH_4	Chr1	103,600,001	104,000,000	400,000
HOL_ROH_5	Chr1	131,200,001	131,600,000	400,000
HOL_ROH_6	Chr2	89,200,001	89,600,000	400,000
HOL_ROH_7	Chr3	8,000,001	8,400,000	400,000
HOL_ROH_8	Chr3	54,800,001	55,200,000	400,000
HOL_ROH_9	Chr3	65,600,001	71,600,000	6,000,000
HOL_ROH_10	Chr3	110,400,001	110,800,000	400,000
HOL_ROH_11	Chr4	22,800,001	29,600,000	6,800,000
HOL_ROH_12	Chr4	33,200,001	35,600,000	2,400,000
HOL_ROH_13	Chr4	65,200,001	65,600,000	400,000
HOL_ROH_14	Chr5	8,800,001	9,200,000	400,000
HOL_ROH_15	Chr5	11,200,001	11,600,000	400,000
HOL_ROH_16	Chr5	19,200,001	34,000,000	14,800,000
HOL_ROH_17	Chr5	39,600,001	40,400,000	800,000
HOL_ROH_18	Chr5	60,400,001	67,200,000	6,800,000
HOL_ROH_19	Chr5	73,600,001	74,000,000	400,000
HOL_ROH_20	Chr5	75,200,001	75,600,000	400,000
HOL_ROH_21	Chr5	84,400,001	98,400,000	14,000,000

HOL_ROH_22	Chr6	56,000,001	56,400,000	400,000
HOL_ROH_23	Chr6	71,200,001	71,600,000	400,000
HOL_ROH_24	Chr6	116,000,001	116,400,000	400,000
HOL_ROH_25	Chr7	24,800,001	25,200,000	400,000
HOL_ROH_26	Chr7	48,000,001	49,600,000	1,600,000
HOL_ROH_27	Chr7	88,400,001	89,600,000	1,200,000
HOL_ROH_28	Chr8	61,600,001	62,000,000	400,000
HOL_ROH_29	Chr8	64,800,001	65,600,000	800,000
HOL_ROH_30	Chr8	107,600,001	108,400,000	800,000
HOL_ROH_31	Chr9	17,600,001	18,000,000	400,000
HOL_ROH_32	Chr9	24,400,001	25,200,000	800,000
HOL_ROH_33	Chr9	43,200,001	43,600,000	400,000
HOL_ROH_34	Chr9	67,600,001	85,600,000	18,000,000
HOL_ROH_35	Chr9	86,000,001	88,000,000	2,000,000
HOL_ROH_36	Chr9	88,800,001	105,600,000	16,800,000
HOL_ROH_37	Chr10	400,001	5,600,000	5,200,000
HOL_ROH_38	Chr10	30,400,001	30,800,000	400,000
HOL_ROH_39	Chr10	34,800,001	35,200,000	400,000
HOL_ROH_40	Chr10	36,400,001	38,000,000	1,600,000
HOL_ROH_41	Chr10	42,000,001	48,800,000	6,800,000
HOL_ROH_42	Chr10	104,000,001	104,305,016	305,016
HOL_ROH_43	Chr11	34,000,001	34,400,000	400,000
HOL_ROH_44	Chr11	81,200,001	86,400,000	5,200,000
HOL_ROH_45	Chr12	16,800,001	23,200,000	6,400,000
HOL_ROH_46	Chr12	42,800,001	64,400,000	21,600,000
HOL_ROH_47	Chr13	400,001	5,200,000	4,800,000
HOL_ROH_48	Chr13	5,600,001	6,400,000	800,000
HOL_ROH_49	Chr13	30,400,001	34,800,000	4,400,000
HOL_ROH_50	Chr13	36,800,001	37,600,000	800,000
HOL_ROH_51	Chr13	43,600,001	44,000,000	400,000
HOL_ROH_52	Chr13	46,000,001	58,400,000	12,400,000
HOL_ROH_53	Chr13	61,200,001	62,400,000	1,200,000
HOL_ROH_54	Chr13	62,800,001	64,400,000	1,600,000
HOL_ROH_55	Chr13	65,200,001	71,600,000	6,400,000
HOL_ROH_56	Chr13	84,000,001	84,240,350	240,350
HOL_ROH_57	Chr14	6,000,001	6,800,000	800,000
HOL_ROH_58	Chr14	11,600,001	12,400,000	800,000
HOL_ROH_59	Chr14	25,200,001	26,000,000	800,000
HOL_ROH_60	Chr14	32,400,001	32,800,000	400,000
HOL_ROH_61	Chr14	44,000,001	44,400,000	400,000
HOL_ROH_62	Chr15	74,800,001	75,200,000	400,000
HOL_ROH_63	Chr16	45,200,001	54,800,000	9,600,000
HOL_ROH_64	Chr16	55,600,001	59,600,000	4,000,000
HOL_ROH_65	Chr18	3,600,001	4,000,000	400,000
HOL_ROH_66	Chr18	12,000,001	15,600,000	3,600,000
HOL_ROH_67	Chr18	34,800,001	35,600,000	800,000
HOL_ROH_68	Chr18	44,800,001	45,600,000	800,000
HOL_ROH_69	Chr20	1	10,000,000	10,000,000
HOL_ROH_70	Chr21	12,800,001	17,200,000	4,400,000
HOL_ROH_71	Chr22	1	400,000	400,000
HOL_ROH_72	Chr22	13,200,001	13,600,000	400,000
HOL_ROH_73	Chr22	20,800,001	27,200,000	6,400,000
HOL_ROH_74	Chr22	33,600,001	34,000,000	400,000
HOL_ROH_75	Chr23	1	400,000	400,000

HOL_ROH_76	Chr23	45,600,001	50,000,000	4,400,000
HOL_ROH_77	Chr23	50,800,001	52,000,000	1,200,000
HOL_ROH_78	Chr24	32,000,001	32,800,000	800,000
HOL_ROH_79	Chr25	37,200,001	39,200,000	2,000,000
HOL_ROH_80	Chr26	19,200,001	20,800,000	1,600,000
HOL_ROH_81	Chr29	26,400,001	30,400,000	4,000,000
HOL_ROH_82	Chr29	41,200,001	48,400,000	7,200,000
